# Lineage-Specific Gene Duplication and Loss in Human and Great Ape Evolution

**DOI:** 10.1371/journal.pbio.0020207

**Published:** 2004-07-13

**Authors:** Andrew Fortna, Young Kim, Erik MacLaren, Kriste Marshall, Gretchen Hahn, Lynne Meltesen, Matthew Brenton, Raquel Hink, Sonya Burgers, Tina Hernandez-Boussard, Anis Karimpour-Fard, Deborah Glueck, Loris McGavran, Rebecca Berry, Jonathan Pollack, James M Sikela

**Affiliations:** **1**Department of Pharmacology and Human Medical Genetics Program, University of Colorado Health Sciences CenterDenver, Colorado, United States of America; **2**Department of Pathology, Stanford UniversityStanford, California, United States of America; **3**Colorado Genetics Laboratory, University of Colorado Health Sciences CenterDenver, Colorado, United States of America; **4**Department of Genetics, Stanford UniversityStanford, California, United States of America; **5**Department of Preventive Medicine and Biometrics, University of Colorado Health Sciences CenterDenver, ColoradoUnited States of America

## Abstract

Given that gene duplication is a major driving force of evolutionary change and the key mechanism underlying the emergence of new genes and biological processes, this study sought to use a novel genome-wide approach to identify genes that have undergone lineage-specific duplications or contractions among several hominoid lineages. Interspecies cDNA array-based comparative genomic hybridization was used to individually compare copy number variation for 39,711 cDNAs, representing 29,619 human genes, across five hominoid species, including human. We identified 1,005 genes, either as isolated genes or in clusters positionally biased toward rearrangement-prone genomic regions, that produced relative hybridization signals unique to one or more of the hominoid lineages. Measured as a function of the evolutionary age of each lineage, genes showing copy number expansions were most pronounced in human (134) and include a number of genes thought to be involved in the structure and function of the brain. This work represents, to our knowledge, the first genome-wide gene-based survey of gene duplication across hominoid species. The genes identified here likely represent a significant majority of the major gene copy number changes that have occurred over the past 15 million years of human and great ape evolution and are likely to underlie some of the key phenotypic characteristics that distinguish these species.

## Abstract 2

This genome-wide analysis reports the major lineage-specific gene copy number changes that have occurred over the past 15 million years of human and great ape evolution

## **Introduction**

### Gene and Genome Evolution

The evolution of genomes has been primarily driven by single basepair mutation, chromosomal rearrangement, and gene duplication (Ohno 1970; Samonte and Eichler 2002), with the latter being the key mechanism for generating new genes and biological processes that facilitated the evolution of complex organisms from primitive ones (Li 1997). These factors are thought to also be important in hominoid evolution and speciation, although a systematic assessment of the relative contribution of each has not yet been possible.

Over the past few years, as the human genome sequence has become available, it has become apparent that recent segmental duplications in the human genome are far more frequent than originally believed, comprising approximately 5% of the available sequence (Bailey et al. 2001). Duplicated regions can range from one to several hundred kilobases in size and show very high sequence similarity (90%–100%) (Bailey et al. 2001; Stankiewicz and Lupski 2002). While such regions can pose unusually difficult challenges for accurate genome assembly (Cheung et al. 2003), they are also likely to be among the most evolutionarily recent duplications and thus are among the most important to human speciation and evolution.

### Interspecies cDNA Array-Based Comparative Genomic Hybridization

The assessment of DNA copy number changes between different human genomes has been aided by the development of comparative genomic hybridization (CGH), which originally involved cohybridizing differentially labeled test and reference genomic DNAs to normal metaphase chromosomes (Kallioniemi et al. 1992). A cytogenetic representation of copy number variation was obtained by scoring the resulting fluorescence ratios along the length of the chromosome. Increased resolution was obtained through the subsequent use of arrayed sets of either large genomic DNA clones or cDNA clones (array CGH [aCGH]) (Pinkel et al. 1998; Pollack et al. 1999), with the latter having the advantage of permitting the analysis of individual genes.

While cDNA microarrays, containing sequences derived from tens of thousands of genes, have been used extensively to profile mRNA expression levels (Schena et al. 1995), their use in aCGH is technically more challenging. Human genomic DNA represents at least a 20-fold increase in complexity compared to human cellular mRNA, and the cDNA array elements represent a smaller (e.g., less than 2 kb), generally more discontinuous hybridization target for a genomic DNA sample. These technical issues notwithstanding, highly reproducible aCGH signals can be obtained using human genomic DNA against high-density human cDNA microarrays, and gene changes as small as an increase or decrease of a single copy can be detected (Pollack et al. 1999).

Until now, cDNA aCGH studies have been limited to only within-species comparisons, partly due to concerns related to the increased sequence divergence that would come into play with interspecies applications. Such sequence divergence may produce differential hybridization signals that would be difficult to distinguish from those that arose from copy number changes. Fortunately, despite their significant anatomical and physical differences, hominoid species show a strikingly high degree of similarity at the genome sequence level, with the average sequence divergence values estimated as 1.24%, 1.62%, and 1.63% for human–chimp, human–gorilla, and chimp–gorilla, respectively, and orangutan showing approximately 3.1% sequence divergence when compared to human, chimp, or gorilla (Chen and Li 2001).

Because of this close sequence conservation, we reasoned that it may be possible to use cDNA aCGH to directly compare the cross-species hybridization signatures of human genes to those of the great apes and to identify genes that have alterations in copy number and/or significant changes in exonic sequence between human and other hominoid species. After we initiated such a cDNA aCGH study, two interhominoid aCGH reports appeared that used arrays containing either cloned or amplified genomic DNAs (Frazer et al. 2003; Locke et al. 2003). While these studies provided useful insights into hominoid DNA copy variations, they afforded little direct knowledge of changes in individual gene copy number and covered only limited sections of the genome. In contrast, interhominoid aCGH using human cDNA microarrays, representing more than 29,000 different genes, would allow a level of genomic resolution not previously obtainable and also provide direct data regarding the recent evolutionary history of a significant majority of human and great ape genes.

## **Results/Discussion**

### Identification of Lineage-Specific Gene Duplication and Contraction

**Figure 1:**
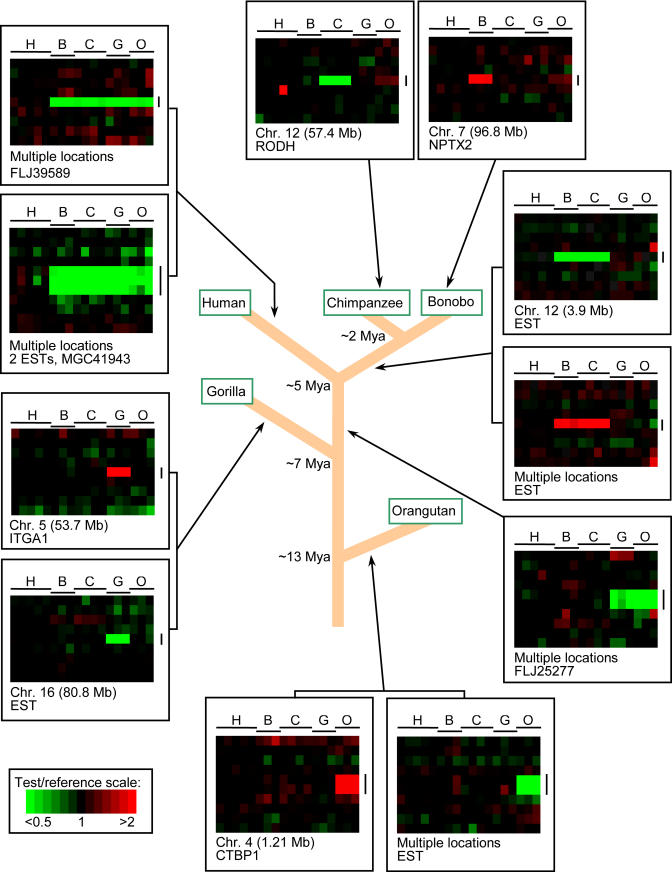
TreeView Images of Examples of Great Ape and HLS Gene Copy Number Increases and Decreases Interhominoid cDNA aCGH was carried out as described in the text and Materials and Methods. Specific test DNAs were, left to right, human (H) (*n* = 5), bonobo (B) (*n* = 3), chimpanzee (C) (*n* = 4), gorilla (G) (*n* = 3), and orangutan (O) (*n* = 3). Each horizontal row represents aCGH data for one cDNA clone on the microarray, while each vertical column represents data from one microarray experiment. Regions shown contain LS genes (vertical black lines) and adjacent flanking genes ordered by chromosome map position using the UCSC Golden Path genome assembly (http://genome.ucsc.edu), November 2002 sequence freeze. Arrows denote for which hominoid lineage the copy number change is unique. Note that fluorescence ratios (pseudocolor scale indicated) reflect copy number changes relative to the human genome. For great ape LS changes, red signal is interpreted according to parsimony as increased gene copy number, and green signal as decreased gene copy number in the specific ape lineage, while increased or decreased gene copy number specific to the human lineage is represented by green or red signal, respectively, in all the great ape lineages. Gray signal indicates cDNA comparisons scored as absent. Estimates of the time at which indicated branch points occurred during hominoid evolution are derived from Chen and Li (2001).

Interhominoid cDNA aCGH was carried out in a series of pairwise comparisons using microarrays containing 39,711 human cDNAs, representing the majority of all human genes (Table S1). The pairwise comparisons involved using a great ape (or human control) as the test genomic DNA sample (Cy5 red dye) and a sex-matched human as the reference genomic DNA sample (Cy3 green dye) in all comparisons. In each experiment, a test and a reference genomic DNA were simultaneously hybridized to a human cDNA microarray under standard cDNA aCGH conditions (Pollack et al. 1999, 2002). Specific test/reference DNAs were bonobo/human, chimp/human, gorilla/human, orangutan/human, and, as a control, human/human. After background was subtracted and data normalized, hybridization signals were scored and fluorescence ratios of the test/reference genomic DNAs determined. Using relatively conservative cutoff values (see Materials and Methods), cDNAs were identified that gave aCGH signatures unique to one or more of the hominoid lineages, permitting such gene changes to be placed within specific evolutionary time frames (Figure 1). The TreeView program (http://rana.lbl.gov/EisenSoftware.htm) was used for visualization of aCGH data for each gene as it occurred in the genome, permitting a “gene-by-gene” survey of the data and allowing for easy detection of interspecies copy number variations, whether they occur as single isolated genes or as multigene blocks.

**Figure 2:**
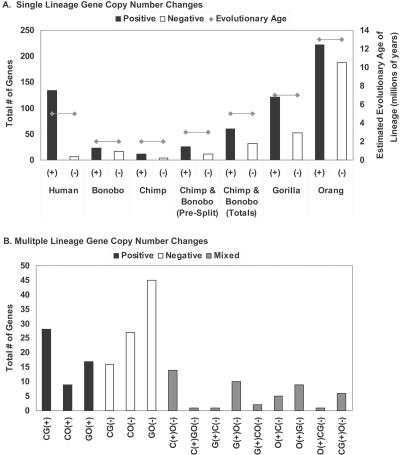
Number of LS Genes for Indicated Hominoid Lineages Totals of aCGH-identified LS genes are indicated for single lineages (A) and multiple (B) lineages, showing both increases (+) and decreases (–) for each. The numbers reflect totals after collapsing the dataset by UniGene cluster to remove redundant cDNAs corresponding to the same gene. Bonobo represents genes unique to this species; likewise with chimpanzee. “Bonobo and chimpanzee (pre-split)” refers to genes that were changed in both species and therefore likely occurred before these species diverged, and “bonobo and chimpanzee (total)” refers to the sum of the previous three categories, which was chosen to represent the period since the *Homo/Pan* split. Estimated evolutionary age of each lineage is also plotted for comparison. Letters denoting different great ape species are as in Figure 1. For (B), bonobo and chimpanzee were grouped together as one lineage (C), but selection criteria had to first be met by both species independently. In (B), no LS genes were identified for the following cases: C(+)G(–); CG(–)O(+); C(–)GO(+); and CO(+)G(–).

Results of the distribution of lineage-specific (LS) aCGH signatures for different individual hominoid species are presented in Figure 2A. Several lines of evidence indicate that the aCGH signature variations that were obtained are primarily due to gene copy number changes and not to interspecies sequence divergence or highly repetitive sequences (Figure S1; see also Materials and Methods). Because bonobos and chimpanzees diverged relatively recently and show a striking degree of sequence similarity (Kaessmann et al. 1999; Wildman et al. 2003), they were dealt with both as individual lineages as well as a single clade. After collapsing the LS dataset by UniGene cluster to remove redundant cDNAs corresponding to the same gene, 815 different genes were identified that gave aCGH signatures unique to a specific hominoid lineage. Each respective lineage and the numbers of genes identified that showed LS copy number change (increases/decreases) are as follows: human: 134/6; bonobo: 23/17; chimpanzee: 11/4; bonobo/chimpanzee pre-split: 26/11; gorilla: 121/52; and orangutan: 222/188.

Several interesting features were evident from these data. First, when increases and decreases were scored separately or combined, the number of LS signatures was generally in proportion to the evolutionary age of that lineage, although not in all cases. Bonobo and chimpanzee, from the time since the *Homo/Pan* split, showed fewer LS signatures (92) than did human (140), even though they represent the same evolutionary age. As mentioned below, this is due in large part to the significant number of LS gene copy number increases found in human.

Second, while all lineages showed more gene copy number increases than decreases, this was most pronounced in humans, with 134 cDNAs representing increases and only six representing decreases. This increase-to-decrease ratio (22.3:1) was significantly greater than that of any of the great apes, which showed ratios ranging from 2.75:1 (chimpanzee) to 1.18:1 (orangutan). It is worth noting that only genes found in the human genome are represented on the cDNA arrays, and if there are genes that are absent in human but present in the great apes, e.g., genes that were lost as the human lineage emerged, those genes would not be part of this analysis. So, while it is likely that the complete loss of both copies of a gene in an LS manner is a rare event, the number of genes identified here as having a reduced copy number specifically in the human lineage may be an underestimate of the true total.

Third, as mentioned above, for all lineages tested, the number of genes showing LS increases was greater than those showing LS decreases. Determination as to whether this is due to some, as yet unknown, ascertainment bias of the method or whether this is a real evolutionary tendency favoring gene duplication over gene loss will require further investigation. The favoring of gains over losses is even more striking when two additional factors are considered. (1) The fact that the cDNAs were only from human, while likely to be important to the low number of genes showing human lineage-specific (HLS) losses previously mentioned, does not help explain why, for all lineages tested, the number of LS genes showing increases was greater than the number showing decreases. To the contrary, if there were genes not on the microarray because they were only found in one or more of the great ape lineages, inclusion of such genes would be expected to add to the total number of LS increases, making the disparity between increased and decreased LS genes even greater. (2) If human/great ape sequence divergence was responsible for some of the LS aCGH signals that were obtained, it would, if anything, produce a falsely elevated number of LS decreases.

Fourth, while only orangutan had more LS gene copy number increases (222) than did human (134), when the number of genes showing copy number increases was measured as a function of the evolutionary age of the lineage, human showed the greatest number of expansions of any hominoid. When measured as copy number increases per million years of age, the following values were obtained: human, 26.8; bonobo and chimpanzee since the *Homo/Pan* split, 12; gorilla, 17.3; and orangutan, 17.1.

We also identified genes that gave aCGH signatures indicative of great ape gene copy number changes, relative to human, that were present in more than one great ape lineage (Figure 2B). For situations in which two great ape lineages showed copy number losses relative to human, there was a general trend that correlated with evolutionary age of the represented species: *Pan*/gorilla, 16 genes; *Pan*/orangutan, 27, and gorilla/orangutan, 45. For gene increases, this trend continued, with gorilla/orangutan (17) showing more changes than *Pan*/orangutan (nine). Interestingly, *Pan*/gorilla showed a departure from this trend with 28 increased genes, suggesting that gene expansion may have been particularly active in the African great apes as a group. There were also a number of more complex gene copy number changes in the five hominoid lineages, with some species showing an increase relative to human for a particular gene and others showing a decrease. These changes are likely due to more than one event, which may be indicative of a genomic region that is relatively unstable and/or of genes whose copy numbers have been influenced by different selection pressures. We identified 190 genes that showed copy number changes in multiple lineages, bringing the total number of LS genes identified to 1,005, which represents 3.4% of the total number of genes tested on the microarrays. Given the relatively conservative selection criteria used (see Materials and Methods), this likely reflects an underestimate of the true total. To visualize the effects of relaxing the selection criteria below a log_2_ fluorescence ratio of 0.5, a series of HLS datasets were generated using progressively reduced thresholds. Using values of 0.45, 0.4, 0.35, and 0.3 added 27, 31, 31, and 22 cDNAs, respectively, as the cutoff was progressively lowered. As seen in the TreeView image of these data (Figure S2), while some of the additional cDNAs could plausibly be scored HLS, several appeared to give marginal HLS signals.

### Independent Confirmation of Interspecies cDNA aCGH Data: Fluorescence In Situ Hybridization Analysis

**Figure 3:**
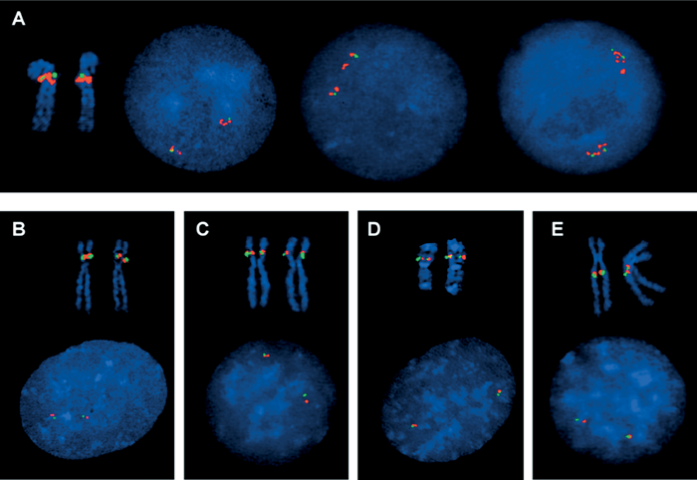
FISH Confirmation of a Human-Specific Duplication of a Gene Cluster on Chromosome 5q13.3 Detected by Interspecies cDNA aCGH (A) Human duplication of a cluster of genes at Chromosome 5q13.3. is shown by two separate, and sometimes multiple, red BAC probe (CTD-2288G5) signals in interphase cells, with only one green BAC probe signal (RP11-1077O1) for a flanking region. Metaphase FISH shows both probes at band 5q13. The third nucleus in (A) shows four signals of the control probe (green) and eight copies of the BAC probe duplicated in the aCGH assay, consistent with the pattern expected in an S/G_2_ nucleus. (B–E) Bonobo (B), chimpanzee (C), gorilla (D), and orangutan (E) interphase FISH studies all show no increased signal for the human duplicated gene cluster, with signals of comparable size for the CTD-2288G5 (red) and the flanking RP11-107701 (green) probes. Metaphase FISH analyses show the gene cluster to be in the p arm of Chromosomes 4 (corresponding to the human Chromosome 5) in both the bonobo and chimpanzee, in the q arm of Chromosome 4 (corresponding to the human Chromosome 5) in the orangutan, and in the p arm of the gorilla Chromosome 19 (syntenic regions to human Chromosomes 5 and 17).

A cluster of several genes located around map position 70 Mb in human Chromosome 5q13.3 showed one of the stronger HLS aCGH signatures. Several of these genes (test probe), as well as a set of flanking genes not shown to be increased in human (control probe), were evaluated by interphase and metaphase fluorescence in situ hybridization (FISH) using bacterial artificial chromosome (BAC) probes (see Materials and Methods). The FISH studies confirmed a duplication of the gene region in human, while the control probe containing a flanking region showed no duplication (Figure 3A). Two separate probe signals (and sometimes multiple probe signals) for the test probe could be seen in interphase nuclei with only one signal for the flanking probe; metaphase chromosomes showed a larger signal for the test probe than for the flanking probe. In all of the four great ape species, on the other hand, the FISH analyses showed no duplication of the gene region; all of these experiments showed a single signal for the test probe and a single signal of comparable size for the flanking probe (Figure 3B–3E). The Golden Path (http://genome.ucsc.edu) genome assembly lists multiple Chromosome 5 locations for some of the HLS cDNAs contained on the positive BAC (e.g., *BIRC1*) and therefore it is likely that the multiple, closely spaced signals seen in some of the human interphase spreads (Figure 3A) reflect additional copies of these genes.

Metaphase FISH showed both the test probe and the flanking probe to be located in the human 5q13 band. Both probes were located in the proximal q arm of the orangutan (PPY) Chromosome 4 and in the p arms of the bonobo (PPA) and chimpanzee (PTR) Chromosomes 4. In the gorilla (GGO), both probes were located on the gorilla Chromosome 19. All of these primate locations are consistent with described evolutionary chromosomal rearrangements, with the orangutan Chromosome 4 considered to be the ancestral Chromosome V (Stanyon et al. 1992). These rearrangements include a pericentric inversion of the ancestral Chromosome V (Chromosome 5 in human, Chromosome 4 in the great apes), in the bonobo and chimpanzee, and a translocation between the ancestral chromosome for human Chromosome 5 and the ancestral chromosome for human Chromosome 17 to form the gorilla Chromosomes 4 and 19.

It is of interest that, considering the orangutan Chromosome 4 as the ancestral Chromosome V, rearrangements at this site have occurred in all of the other three great ape species (pericentric inversion in bonobo and chimpanzee, translocation in gorilla) and in the human (gene duplication). This region is also involved in spinal muscular atrophy (SMA), which is characterized by deletions of one or more genes in this region (Lefebvre et al. 1995). Taken together these data suggest this region is one of high genomic instability that is relevant to both disease and evolutionary processes.

### Independent Confirmation of Interspecies cDNA aCGH Data: Literature-Based Validation

#### 

**Figure 4:**
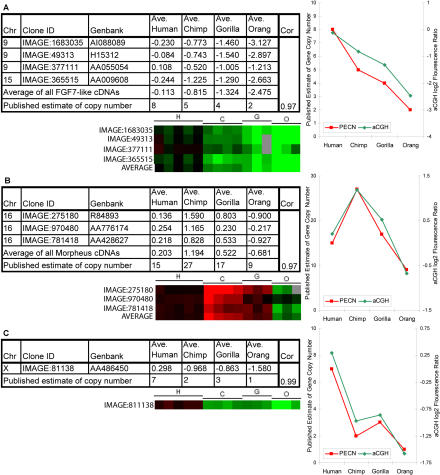
Independent Confirmation of Interspecies cDNA aCGH Data for Three Gene Families with Known Species Differences in Copy Number The chromosomal location, IMAGE clone ID, and GenBank accession are provided for each cDNA. The species average log_2_ ratios for each cDNA clone and the previously published estimate of gene copy number are shown for the indicated species. Also shown are TreeView images of interhominoid aCGH results for the indicated cDNAs, and a graphical depiction of the correlation between aCGH signal and published estimate of gene copy number (PECN). (A) *FGF7* cDNA clone located on human Chromosome 15 was identified using the UCSC November 2002 human genome assembly and *FGF7*-like cDNA clones located on human Chromosome 9 were identified based on UniGene cluster sequence similarity to *FGF7* reference sequence NM_002009. The correlation between published and aCGH-based copy number estimates is 0.97. (B) *morpheus* family cDNA clones were identified based on sequence similarity to one *morpheus* family member (Johnson et al. 2001). As in (A), except data relate to the *morpheus* genes and published data are from Johnson et al. (2001). Correlation = 0.97. (C) As in (A), except data relate to the *CXYorf1* genes and published data are from Ciccodicola et al. (2000). Correlation = 0.99.

***FGF7*-like genes.** Some genes we identified as having LS aCGH signatures have been previously studied by others using different methods, which provides a means of independently checking the accuracy of the cDNA aCGH data presented here. One such gene, the *FGF7* gene on Chromosome 15, was studied by Zimonjic et al. (1997) using FISH analysis of the same hominoids used in this study. The FISH analysis showed an interhominoid variation in gene copy number with eight copies in human, five in chimp, four in gorilla, and two in orangutan. Interspecies aCGH data presented here mirrored these results (correlation = 0.97), showing an elevation of the human gene number with respect to the chimp, gorilla, and orangutan, with the most pronounced difference being between human and orangutan (Figure 4A).

#### ***Morpheus* genes**

Recently the identification of a multimember gene family named *morpheus* on Chromosome 16 was reported and shown to exhibit gene copy number variation between several hominoid species (Johnson et al. 2001). Using a combination of approaches, the investigators estimated copy numbers for the *morpheus* genes to be 15, 25–30, 17, and nine for human, chimp, gorilla, and orangutan, respectively. In order to provide an independent test of the accuracy of the interspecies cDNA aCGH data we generated, the aCGH signatures of *morpheus*-like cDNAs were assembled for the same hominoids (Figure 4B). The average test/reference log_2_ ratios for these cDNAs indicated that chimpanzee had the most copies, gorilla was slightly higher than human, and orangutan clearly had the fewest, results that are in very good agreement (correlation = 0.96) with the copy number estimates reported independently by Johnson et al. (2001).

#### ***CXYorf1* genes**

Ciccodicola et al. (2000) used cross-species FISH to estimate the hominoid gene copy numbers for the *CXYorf1* gene family. They found values of seven, two, three, and one for human, chimpanzee, gorilla, and orangutan, respectively. These values closely mirrored the aCGH values that were obtained (Figure 4C) (correlation = 0.99).

Based on aCGH data, the *FLJ22004* gene shows the greatest gorilla-specific copy number increase (average log_2_ ratio = 3.94). This gene resides near the fusion region on Chromosome 2q14.1 (see below) and is contained within BAC RP11-432G15. Consistent with the aCGH data, two independent interhominoid FISH studies, by our lab (Figure S3) and by Fan et al. (2002), using this BAC showed that the copy number was highly elevated (more than 30 signals) in gorilla relative to all other hominoids tested (fewer than or equal to three signals).

Further independent support for the accuracy of the aCGH data comes from a comparison of the HLS gene dataset to the segmental duplication dataset generated by Bailey et al. (2002a), who used whole genome shotgun data to generate a genome-wide database (the Whole Genome Shotgun Segmental Duplication [WSSD] database) of recent (less than 40 million years ago [MYA]) segmental duplications for the human genome (see Table S2). The majority of changes in copy number of the HLS gene set we identified are likely to have occurred since the *Homo/Pan* split (less than 5–6 MYA) and therefore should represent a subset of the segmental duplications found in the WSSD dataset. Results of this analysis confirmed this expectation (Table 1): 80% of HLS genes gave significant basic local alignment search tool (BLAST) scores with the WSSD dataset (as a control, only 13% of a randomly selected set of cDNAs were positive for the WSSD dataset), and 57% (5414/9461) of the segments in the WSSD were positive with the HLS gene list.

**Table 1:**
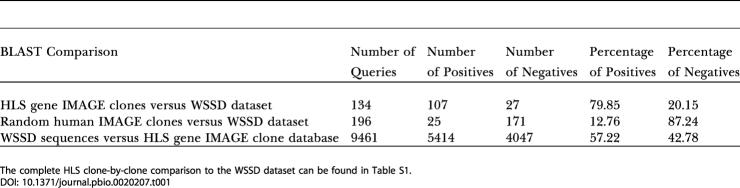
Comparison of HLS Gene and WSSD Datasets

The complete HLS clone-by-clone comparison to the WSSD dataset can be found in Table S1

### Non-Random Distribution of LS Genes

**Figure 5:**
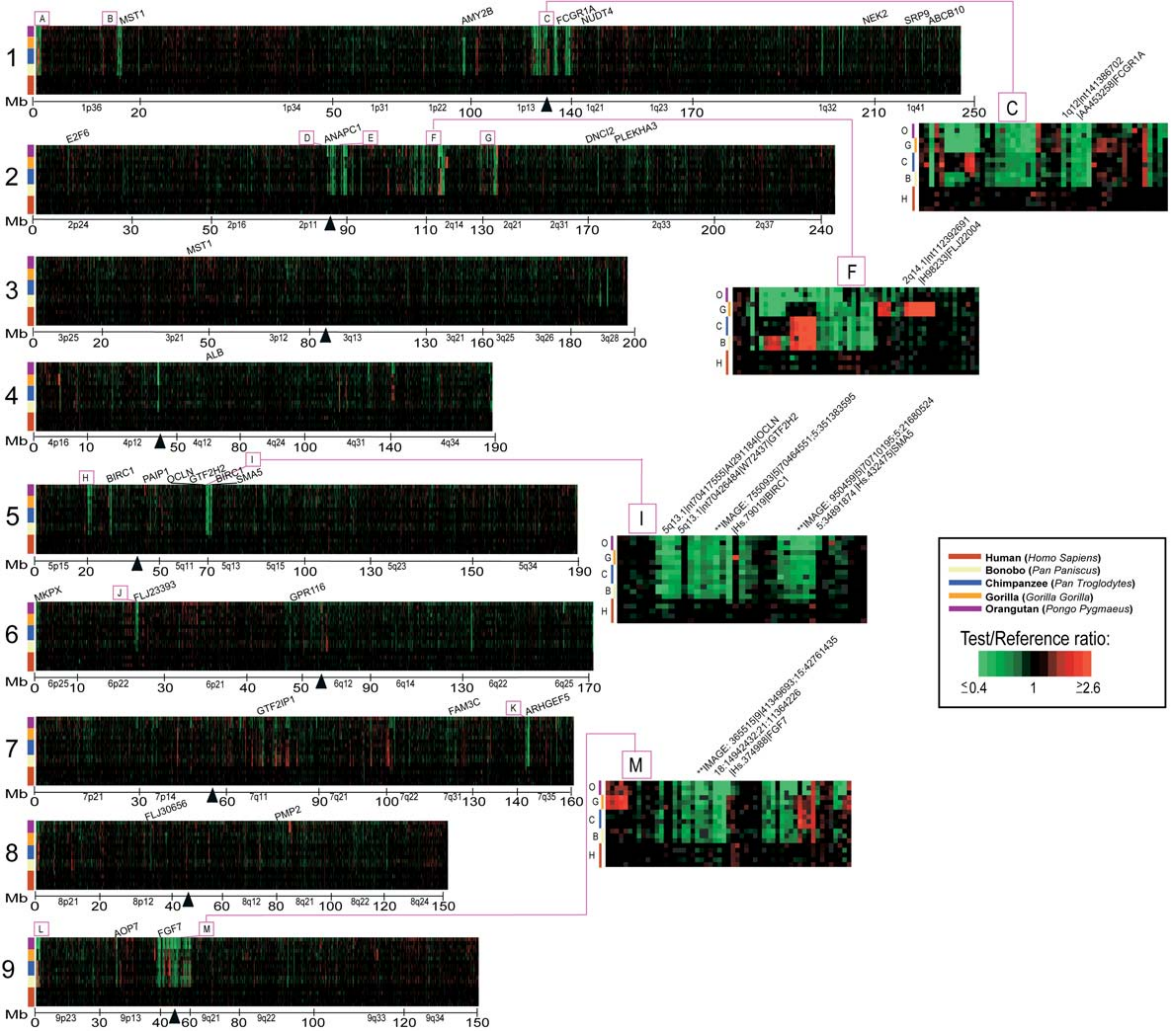
Whole Genome TreeView Representation of Interhominoid cDNA aCGH Data for Five Hominoid Species for Human Chromosomes 1–9 Hominoid species are identified by color bar (see key). Genes along each chromosome are ordered by map position. cDNAs mapping to multiple genome locations (more than 1 Mb apart) are shown at each of the multiple genomic locations. Fluorescence ratios are depicted using a pseudocolor scale (indicated). Megabase positions, cytobands, centromeres (black vertical triangles), and selected genes are indicated. Boxed and lettered regions (A–M) identify clusters of LS genes (greater than or equal to eight per cluster); insets show detailed views of clusters C, F, I, and M. The complete annotated interhomioid aCGH dataset depicted here is available in Table S1 and can be viewed either as a TreeView image (see Protocol S1) or as a tab-delimited text file that can be opened in Excel.

**Figure 6:**
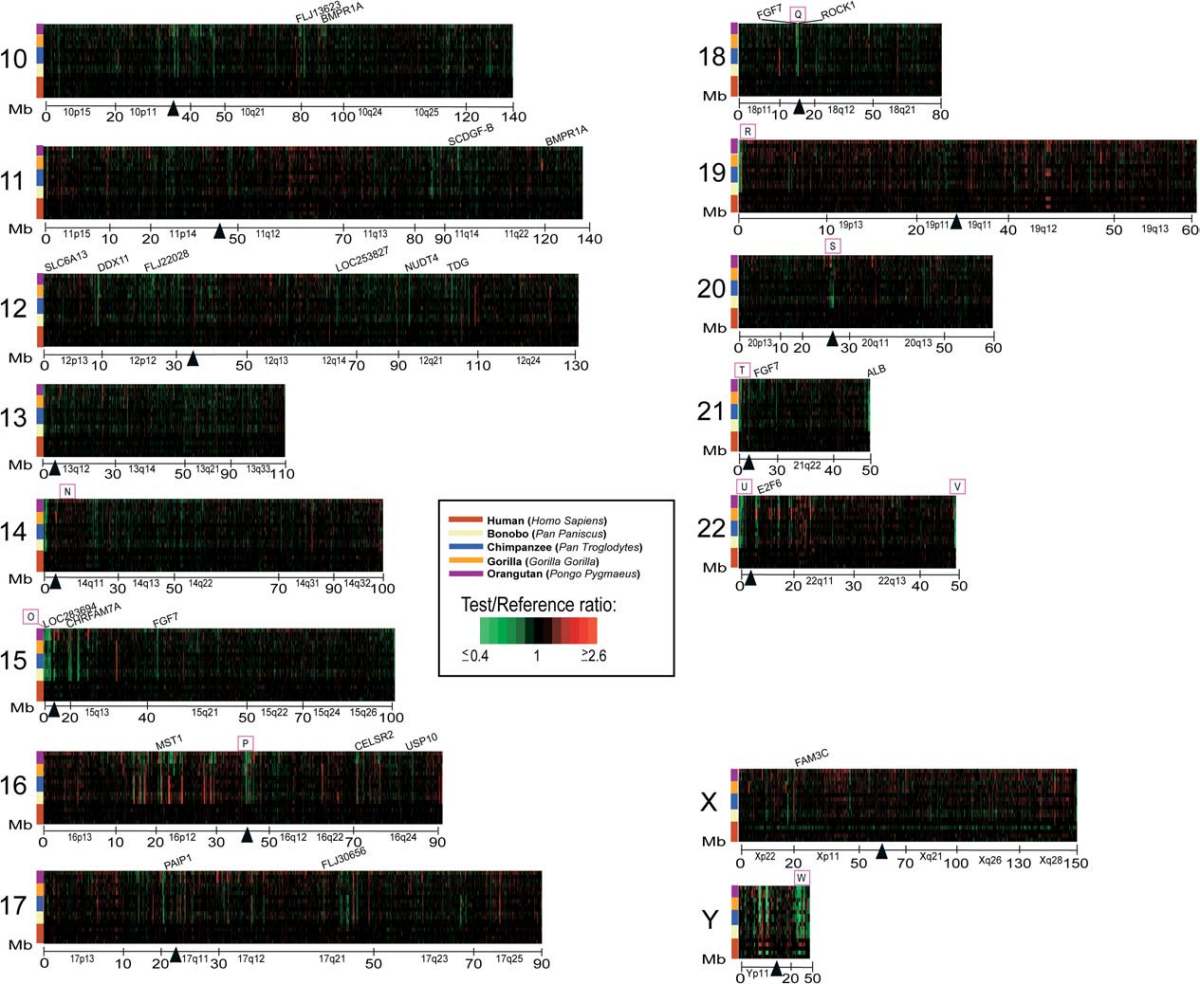
Whole Genome TreeView Representation of Interhominoid cDNA aCGH Data for Five Hominoid Species for Human Chromosomes 10–22, X, and Y Data are as described for Figure 5, except boxed and lettered regions denoting clusters of LS genes are N–W. The complete annotated interhomioid aCGH dataset depicted here is available in Table S1 and can be viewed either as a TreeView image (see Protocol S1) or as a tab-delimited text file that can be opened in Excel.

Genes identified as having a variation in copy number specific for one or more hominoid lineages occurred either as single isolated genes or as clusters of genes. This latter category likely reflects LS copy number changes that involved blocks of contiguous genes. In addition, certain specific regions of the genome, while not necessarily composed of contiguously positioned LS genes, showed a marked enrichment for LS genes. Surveying the genome for regions containing contiguous gene clusters of LS genes or for regions highly enriched in LS genes (greater than or equal to eight contiguous or nearly contiguous LS cDNAs) identified 23 prominent sites (Figures 5 and 6; Table 2). Most (18) of these are not randomly distributed in the genome, but instead are found near regions thought to be more genomically and evolutionarily dynamic. Among these are heterochromatic C-band regions, pericentromeric and subtelomeric regions, breakpoints of recent pericentromeric inversions, and sites of recent chromosomal fusions. For example, the two cytogenetic regions with the most LS genes represented were 1p13.2–1q21.2 (66 cDNAs) and 9p13.3–9q21.12 (77 cDNAs) (see insets in Figure 5, regions C and M). Interestingly, these regions are also known to contain C-band regions of heterochromatin which, along with C-band regions at pericentromeric 16 and at the distal end of Yq, are found at these chromosomal locations only in human and are known to be highly polymorphic. (While C-band chromosomal regions contain the alphoid class of repetitive DNA, there are several reasons that argue that the LS signals in these regions are not due to human-specific repetitive DNA. First, several HLS cDNAs were checked and found to contain no repetitive sequences in them. Second, *Cot-1* analyses, described earlier, indicated that HLS signals did not correspond to repetitive DNA regions. Third, the genes in these regions showed LS signals for other hominoid lineages in addition to human.). The regions near the C-band regions on 16 (15 cDNAs) and Y (14 cDNAs) also showed an enrichment of LS genes, although to a lesser extent. These regions, as well as the pericentromeric regions of the acrocentric chromosomes, which showed enrichment for LS genes, are known to contain highly repetitive DNA, which may make them especially prone to recombination and duplication.

**Table 2:**
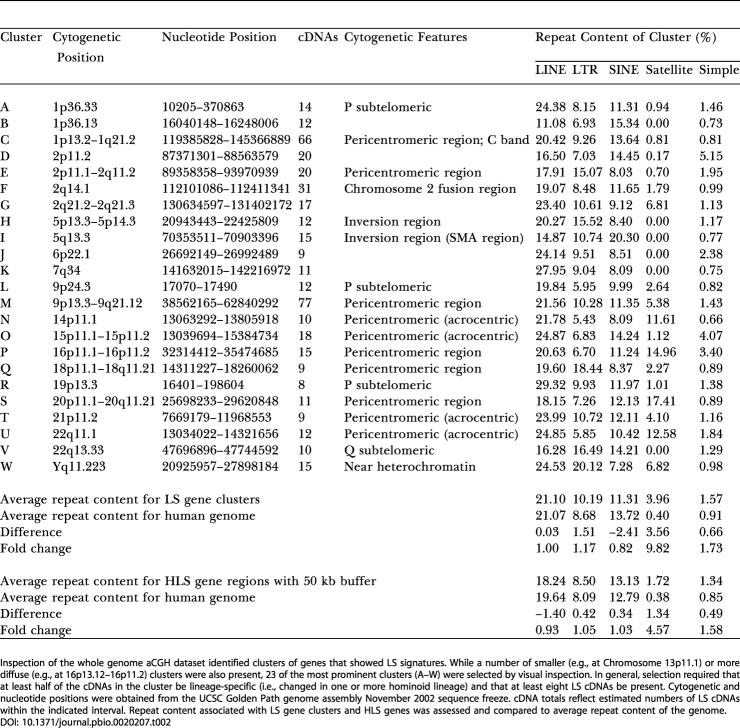
Genome Distribution and Repeat Content of Clusters of LS Genes

Inspection of the whole genome aCGH dataset identified clusters of genes that showed LS signatures. While a number of smaller (e.g., at Chromosome 13p11.1) or more diffuse (e.g., at 16p13.12–16p11.2) clusters were also present, 23 of the most prominent clusters (A–W) were selected by visual inspection. In general, selection required that at least half of the cDNAs in the cluster be lineage-specific (i.e., changed in one or more hominoid lineage) and that at least eight LS cDNAs be present. Cytogenetic and nucleotide positions were obtained from the UCSC Golden Path genome assembly November 2002 sequence freeze. cDNA totals reflect estimated numbers of LS cDNAs within the indicated interval. Repeat content associated with LS gene clusters and HLS genes was assessed and compared to average repeat content of the genome

Previous reports have shown that recent (less than 40 MYA) segmental duplications in the human genome are positionally biased and found more frequently in pericentromeric and subtelomeric regions (Bailey et al. 2001; Mefford and Trask 2002; Samonte and Eichler 2002). Consistent with this, most of the LS clusters we identified mapped to either pericentromeric (10/23) or subtelomeric (4/23) regions (Table 2). Also, a recent report by Bailey et al. (2002b) showed that a 400 kb HLS duplication transposed from Chromosome 14 to the most proximal pericentromeric region of Chromosome 22 (at approximately 13–14 Mb) and suggested that a pericentromeric gradient of duplications exists in which the most recent duplications transpose nearest to the centromere. Data presented here, showing a cluster of LS genes in this same region with HLS changes occurring nearer to the centromere, are consistent with this view.

Additional clusters were also identified at other sites known to be particularly unstable and prone to rearrangement and duplication. For example, the 5q13 region (see inset to Figure 5, region I) is known to be involved in SMA, and deletions in the *BIRC1* gene, which we show is amplified uniquely in humans, are sometimes found in SMA patients. This region and another at 5p14.3–5p13.3 that also contains a cluster of LS genes are near the breakpoint sites of a pericentric inversion that occurred during hominoid speciation (Yunis and Prakash 1982). Another unstable region, the 2q14.1 region (see inset to Figure 5, region F), is known to be the site at which two ancestral ape chromosomes fused telomere-to-telomere to form human Chromosome 2 (IJdo et al. 1991; Fan et al. 2002). This region shows a complex pattern of LS genes, with aCGH gene signatures specific for at least four different hominoid lineage combinations represented within a genomic region of only 400 kb. Enrichment of LS genes was also found in regions associated with other genetic disorders, including Di George syndrome, Williams–Beuren syndrome, and Angelman and Prader–Willi syndromes. Taken together, these data support the view that regions of the genome that are particularly unstable are enriched for LS gene copy number changes and are often disease-associated hotspots of evolutionary change.

To assess the frequency and type of repeated sequences associated with the HLS gene and LS gene cluster datasets, the repeat content near these genes was determined. Of known repeat classes surveyed, only the Satellite class showed a major deviation from the overall genome frequency (Table 2). Satellite repeats associated with LS gene clusters and HLS genes were 10-fold and 4-fold enriched, respectively, over the genome average frequency. This may not be unexpected given the known pericentromeric and subtelomeric positional bias of Satellite sequences and their known involvement in interchromosomal duplication processes (Horvath et al. 2000). Relative frequencies of the subclasses of Satellite sequences associated with each cluster can be found in Table S3.

### Genes Showing HLS Variation in Copy Number

**Figure 7:**
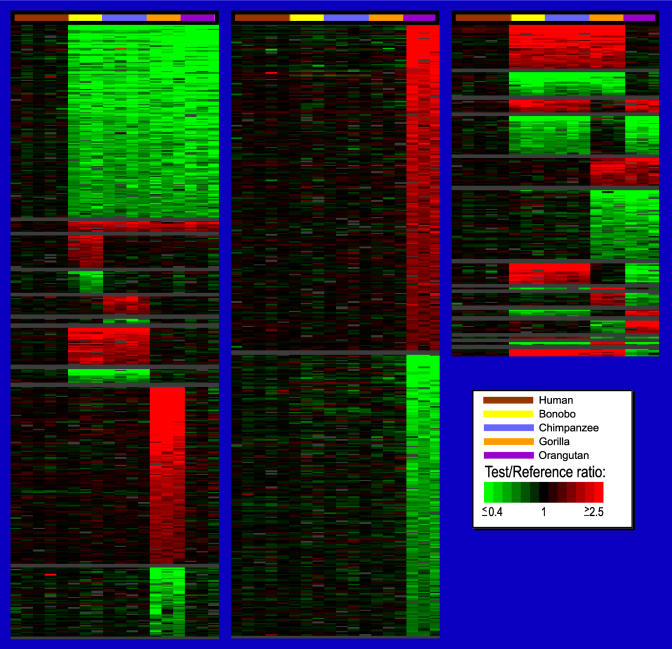
TreeView Images of LS Genes for Different Hominoid Lineages and Lineage Combinations Ranked as a Function of aCGH Ratio TreeView representation of cDNAs that exhibit great ape or human LS aCGH signatures are presented. Order of genes within each lineage is based on the average log_2_ fluorescence ratios (ordered highest to lowest) of the respective species. The dataset used for this figure was not collapsed by UniGene cluster to minimize the chance that significant LS cDNAs would be missed. Fluorescence ratios are depicted using a pseudocolor scale (indicated). The complete annotated LS dataset depicted here is available as Table S4 and can be viewed either as a TreeView image (see Protocol S1) or as a tab-delimited text file that can be opened in Microsoft Excel.

Of the 140 genes showing HLS variation in copy number, 134 represented human gene increases and six represented decreases (Figure 7; Table S4). While roughly half of these genes were represented as expressed sequence tags (ESTs) or uncharacterized genes with little or no information as to possible biological function, the remaining cDNAs corresponded to known genes. Among this latter category were a number with interesting predicted functional characteristics. For example, the gene encoding the neuronal apoptosis inhibitory protein (NAIP or *BIRC1*) maps to Chromosome 5q13 and was elevated specifically in the human lineage. NAIP has been implicated in delaying neuronal programmed cell death (Liston et al. 1996) and is known to have at least one duplicated copy in the genome that appears to be functional (Xu et al. 2002). If an increase in gene dosage results in an elevated functional effect, the possibility exists that such an LS increase in NAIP gene copy number may contribute to an increase in neuronal proliferation and/or brain size (either globally or regionally) in humans.

Several other genes implicated in neuronal function showed HLS changes in copy number: a neurotransmitter transporter for γ-aminobutyric acid (GABA) (*SLC6A13*), a leucine zipper-containing gene highly expressed in brain (*KIAAA0738*), α7 cholinergic receptor/*Fam7* fusion gene (*CHRFAM7A*), a p21-activated kinase (*PAK2*), a Rho GTPase-activating protein (*SRGAP2*), a Rho guanine nucleotide exchange factor (*ARHGEF5*) that is a member of the rhodopsin-like G protein-coupled receptor family, and Rho-dependent protein kinase (*ROCK1*). Inhibition of *ROCK1* has been shown to prevent long-term memory, and *ROCK1*, together with a RhoGEF and RhoGAP, have been recently implicated in a model of long-term memory based on fear conditioning (Lamprecht et al. 2002). Also, members of the ARHGEF, PAK, and RhoGAP gene families comprise a disproportionately high fraction of the genes known to produce syndromic or nonsyndromic forms of mental retardation (Ramakers 2000).

Another gene showing an HLS copy number increase, *USP10*, encodes a ubiquitin-specific protease, an enzymatic class implicated in learning and memory and in synaptic growth (DiAntonio et al. 2001). Overexpression of the *USP10* homologue in *Drosophila* leads to uncontrolled synaptic overgrowth and elaboration of the synaptic branching pattern (DiAntonio et al. 2001), raising the possibility that the human-specific copy number increase for *USP10* could be relevant to expanded synaptic growth in humans. Interestingly, the *USP10* gene at Chromosome 16q24 and an unknown gene (integrated molecular analysis of genomes and their expression [IMAGE] 854706) at Chromosome 19q13 that is significantly elevated in human relative to most hominoids map to the two chromosomal regions giving the highest LOD scores in a recent genome-wide scan related to specific language impairment (SLI Consortium 2002).

The aquaporin 7 gene (*AQP7*), which is thought to be involved in water transport across membranes, shows an HLS increase in copy number, while the genes immediately flanking it (*NFX1* and *AQP3*) do not show HLS aCGH signals. Similarly, Bailey et al. (2002a) predict that a 22 kb region containing the *AQP7* gene has been recently (less than 40 MYA) duplicated several times while flanking regions show no recent duplication. These data suggest that a series of HLS segmental duplications occurred that focused primarily on the *AQP7* gene, which spans 17 kb of the 22 kb duplication. This observation, together with the fact that several of the additional *AQP7* copies appear to be potentially functional (see below), raises the possibility that significant selection pressure may have been exerted on *AQP7*-like genes specifically in the human lineage.

### Genes Showing Copy Number Variation Specific to One or More Great Ape Lineages

In addition to identifying HLS gene changes, interhominoid cDNA aCGH allows genes to be identified that have changed during other branch points within the past 15 MY of hominoid evolutionary history. In the present study, 865 great ape LS genes were identified (Figure 7; Table S4), several of which are mentioned below.

Chimpanzees are known to be the original reservoir for HIV and show genetic resistance to progression to AIDS (Novembre et al. 1997; Gao et al. 1999), a process likely to be immunologically mediated. Among genes elevated in copy number in chimpanzees are several with possible relevance to immune function, including the *BMI1* gene (B-cell lymphoma Mo-MLV insertion region) and, in bonobos and chimps, the *FCER2* gene, encoding a lymphocyte IgE receptor, and the *IL1RL1* gene encoding an interleukin receptor 1-like protein. Also, it has been shown that chimpanzees can synthesize a form of sialic acid while humans cannot, owing to the loss of function in humans of a specific sialic acid hydroxylase (Muchmore et al. 1998). Interestingly, one of the genes elevated in chimpanzees and bonobos encodes a CMP-sialic acid transporter (SLC35A1).

As mentioned previously, of genes specifically amplified in the gorilla lineage, the *FLJ22004* gene showed the largest gorilla-specific aCGH signal increase. While the function of this gene is unknown, the encoded protein contains a *DUF6* domain, which is found in the *Erwinia* PecM protein involved in cellulase and pectinase regulation (Rieder et al. 1998). Interestingly, gorillas more than any other hominoid are folivorous. They eat leaves primarily, but also (like other hominoids) fruit, foods that contain energy-rich cellulose and pectin. This fact, together with the observation that *FLJ22004* is highly amplified only in the gorilla lineage, raises the possibility that amplification of this gene provides enhanced cellulase and pectinase capabilities, which in turn would facilitate utilization of the two key dietary staples of this species.

Another gene specifically increased in gorilla (average log_2_ ratio = 2.02) encodes the fibroblast growth factor receptor 3 (FGFR3), which when disrupted in humans causes achondroplasia, the most frequent form of short-limb dwarfism. The *SET8* gene is also significantly elevated in copy number only in gorilla (average log_2_ ratio = 2.65) and also related to development. The gene encodes a transcription factor and appears to be homologous (protein similarity of 43% over 110 amino acids) to the Drosophila trithorax gene, which functions in segmentation determination through interaction with *bithorax* and *antennapedia* complex genes, suggesting that it may serve a role in gorilla-specific development. There were a significant number of genes (28) showing increased copy numbers specifically in the African great apes (bonobo, chimpanzee, and gorilla). Among these were the *MSTP028* gene, encoding a voltage gated potassium channel; the *PLA2G4B* gene, encoding phospholipase A2β, which shows high brain and (in particular) cerebellar expression; and the *SPTBN5* gene, which encodes a nonerythroid spectrin. *SPTBN5* is immediately adjacent to *PLA2G4B* at Chromosome 15q15.1 in the genome and, like *PLA2G4B*, shows high cerebellar expression, raising the possibility that their function(s) in the African great apes may be linked. Finally, while the HLS and LS genes mentioned above have interesting biological implications related to human and great ape differences, each should be viewed as tentatively HLS or LS until the interhominoid copy number differences for these genes are confirmed by independent methods.

### Functional Classification of HLS and LS Genes

Classification of HLS and LS genes according to predicted molecular function was carried out by Gene Ontology (GO) analysis. For the great majority of functional categories, both HLS and LS gene groups gave GO distributions similar to that found with all known genes (UniGene collapsed set), with ligand binding, catalytic activity, signal transducer activity, and transporter activity being the four most highly represented functional categories (Figure S4; Table S5). This analysis should be tempered somewhat by the fact that almost half of all HLS and LS genes are unclassified or lack functional information and that some human genes are not present on the microarrays used (e.g., only 20–30 olfactory receptor-related cDNAs were on the microarrays while, in hominoids, this family is thought to be comprised of several hundred functional members [Gilad et al. 2003]).

It can be expected that copies arising from gene duplications will be a mix of functional genes and pseudogenes, the exact ratio of which will vary depending on the gene involved. Although definitive assessment of the functional status of the copies of HLS genes identified here requires additional study, a preliminary analysis of several HLS genes, including those mentioned above, found this general trend to be evident (Table S6). For example, analysis of BLAST-like alignment tool (BLAT) hits for the *AQP7* gene predicts that of seven closely related (greater than 90%) copies in the genome, at least four appear to be potentially functional. In contrast, the *FLJ13263* gene had four closely related sequences, and these all appear to be pseudogene-like. Finally, the fact that it has been shown that pseudogenes can play important functional roles (Hirotsune et al. 2003) implies that one cannot assume that even bonafide pseudogene copies will necessarily be functionally silent or unimportant to evolutionary differences between species.

### Human and Chimpanzee Genome Sequences

A human versus chimpanzee genome comparison is now publicly available, through the University of California, Santa Cruz (UCSC) database's best reciprocal alignment of the July 2003 human genome and the November 2003 Arachne 4X chimpanzee draft genome (http://genome.ucsc.edu/goldenPath/hg16/versusPt0/). Using this comparison, we have determined that genes that gave aCGH signatures indicative of copy number increase specifically in the human lineage, showed a 7-fold increase in the frequency of gaps and absent sequence homology in the chimpanzee draft compared to a randomly selected gene (EST) set (Table S7). Such a pronounced bias would be expected for genes with significant copy number increases in human relative to chimpanzee, independently supporting the accuracy of the HLS gene dataset we have defined.

However, a limitation of only comparing the human and chimpanzee genomes is that no out-group analysis is provided, preventing discrimination of ancestral and derived forms and limiting the ability to identify gene copy number changes unique to a specific hominoid lineage. In contrast, the interhominoid aCGH studies described here provide reliable genome-wide data for out-group analysis across five primate species, allowing easy identification of LS copy number differences.

In order to provide some perspective on the importance of out-group data when trying to identify LS gene changes, a comparison was carried out between two aCGH clone sets. One set contained 153 genes we identified by cDNA aCGH that were specifically increased in copy number in the human lineage when compared to each of the four great ape lineages (i.e., HLS). The other clone set, while derived from the same aCGH experiments using the same cutoff values, contained 353 genes that showed aCGH signals in which the human copy number was greater than the chimpanzee (i.e., “human > chimp”). Comparison of these two datasets allows one to determine how frequently a “human > chimp” gene is also HLS (i.e., human copy number is greater than *each* of the four great apes studied). Of the 353 genes that were “human > chimp,” 200 were not found in the HLS set, indicating that over half (57%) of the “human > chimp” genes were not HLS.

It has been pointed out that the human genome is a mosaic composed of some regions more closely related to chimpanzee and, less frequently, others more closely related to gorilla (Pääbo 2003). Data presented here contain a number of examples of genes showing such evolutionary histories, but also contains examples of other more complex phylogenetic patterns (Figure 7; see Table S4). For example, the significant number of genes showing copy number increases or decreases specifically in the African great apes, in which human and orangutan copy numbers were equivalent to one another, suggests that either more than one event occurred to produce this distribution or the genomic mosaicism found in the human genome extends back to include sequences present at the time the orangutan lineage split. Because of this unusual phylogenetic profile, we tested several such cDNAs by interhominoid real-time PCR (RT-PCR) and FISH as an independent verification of our aCGH results. In all cases, copy number estimates based on RT-PCR analysis showed high correlation (0.94–0.97) to estimates based on our aCGH data (Figure S5). Interestingly, FISH analysis using a BAC probe containing two genes (*PLA2G4B* and *SPTBN5*) specifically elevated in the African great apes, showed that, in chimpanzee, signals were widely distributed among many chromosomes, while in gorilla the signals were restricted to two sites, one single copy and the other multicopy (Figure S6). These results indicate that the increase in gene copy number in gorilla and chimp occurred independently of each other and therefore support the view that multiple separate events are likely responsible for the African great ape-specific aCGH signals we obtained.

In summary, the dataset presented here, containing over 714,000 aCGH datapoints, represents to our knowledge the first genome-wide survey of gene duplication and loss across five hominoid species. The changes identified likely represent most of the major LS gene-associated copy number changes that have occurred over the past 15 MY of human and great ape evolution. Further analyses of this dataset, of which only a fraction has been highlighted here, should provide additional insights into gene duplication and genome evolution, the relationship of genome instability, evolutionary adaptation, and disease, and the genes that underlie the phenotypic differences among human and great ape species.

## Materials and Methods

### Copy Number Variation, Sequence Divergence, and Repetitive Sequences

Though discussed above as copy number alterations, changes in cross-species cDNA aCGH signals could be due to changes in gene copy number between species, to pronounced exonic sequence divergence of the gene between species, or to a combination of both. To attempt to distinguish among these possibilities, we took advantage of the fact that, while cDNAs are randomly positioned on the microarrays, for analysis purposes they had previously been computationally grouped into two categories: cDNAs with single known genome locations (i.e., unique location) and cDNAs that mapped to multiple genomic locations (multiple locations). In this latter category, we also included a minority of cDNAs that had no assignable location in the genome assembly. We identified HLS cDNAs that showed stronger hybridization with human DNA (green signals in all great ape/human comparisons) and determined how many of these occurred in each of the two mapping categories. HLS signatures were found for 0.185% of unique location cDNAs (66/35,680) and 2.88% of multiple location cDNAs (116/4,031), a frequency difference of more than an order of magnitude (approximately 1:16).

Such a strong enrichment, in the multiple location category, of genes showing increased human aCGH signals specific to the human lineage would be expected if such genes were present as multiple closely related copies with distinct genome locations and, as a result, were placed in the multiple location group. No such gene distribution bias would be expected if the LS signatures were mainly due to sequence divergence.

Additionally, we estimated what fraction of LS cDNAs in each species were cDNAs with multiple human map positions. Values of 59%, 10%, 13%, 14%, 10%, and 20% were obtained for human, bonobo, chimp, bonobo/chimp total, gorilla, and orangutan, respectively, providing further support that the increased (i.e., green in all great ape:human comparisons) HLS aCGH signatures that were obtained are likely due to gene copy number increases specific to the human lineage.

We also carried out interhominoid FISH using a BAC probe (RP11-93K3) containing a gene (IMAGE 1882505) that gave a reduced signal specifically in the orangutan lineage, which is the lineage where sequence divergence might have its greatest artifactual contribution. Resulting FISH data (see Figure S1) showed 10–15 signals in human, bonobo, chimpanzee, and gorilla, while for orangutan only two signals were evident. Finally, further evidence of aCGH data reflecting copy number change comes from the three examples of literature-based validation of aCGH-predicted copy number changes (see Figure 4). In all three cases, the orangutan signals were reduced relative to the human signals, and each of these genes were shown in published reports to have fewer copies in orangutan relative to human.

Lastly, to address the possibility that such signals might be due to highly repetitive sequences associated with LS genes that were not effectively blocked during hybridization, we examined the cDNA sequences of five cDNAs that showed stronger hybridization with human DNA. In all cases no repeats were found that would account for the HLS aCGH data. In addition, hybridization using labeled *Cot-1* DNA (human *Cot-1* versus total human DNA) indicated that there was no correspondence between genes hybridizing more strongly to *Cot-1* and genes that are LS.

### DNAs

DNAs that were used for this study were derived from human (two females, two males), bonobo (three males), chimpanzee (one male, three females), gorilla (one male, two females), and orangutan (three females). Human and chimpanzee genomic DNA samples were isolated from blood cells using Super Quick-Gene kits from the Analytical Genetic Testing Center (Denver, Colorado, United States). One gorilla and two bonobo samples were isolated from cell lines using DNeasy Tissue kits from Qiagen (Valencia, California, United States). An orangutan sample and a gorilla sample were isolated from blood by other laboratories. Remaining DNAs (one bonobo, one gorilla, and two orangutan) were obtained from the Coriell Institute (Camden, New Jersey, United States) and originally derived from primary fibroblast cell lines.

### aCGH

DNA microarrays used in this study were fabricated by PCR-amplifying IMAGE clones (http://image.llnl.gov) and spotting them onto Corning GAPSII aminosilane slides using a custom-built robotic arrayer (http://cmgm.stanford.edu/pbrown/mguide/index.html). The labeling of genomic DNA and hybridization to cDNA microarrays were performed as previously described (Pollack et al. 1999). In brief, 4 μg of genomic DNA from test (hominoid DNA) and sex-matched reference (normal human DNA) were DpnII-digested (New England Biolabs, Beverly, Massachusetts, United States) and subsequently purified using Qiaquick PCR purification kit (Qiagen). Purified samples were random-primer labeled according to manufacturer's directions in a 50 μl reaction volume using BioPrime Labeling Kit (Invitrogen, Carlsbad, California, United States), with the exception of substituting the provided dNTP mix with dATP, dGTP, dTTP (120 μM), dCTP (60 μM), and Cy3-dCTP (reference) or Cy5-dCTP (test) at 60 μM. Labeled Cy3-dCTP and Cy5-dCTP products were copurified and concentrated using Microcon YM-30 filters (Millipore, Billerica, Massachusetts, United States) along with 50 μg of human *Cot-1* DNA (Invitrogen), 100 μg of yeast tRNA (Invitrogen), and 20 μg of poly(dA-dT) (Sigma, St. Louis, Missouri, United States) to block hybridization to nonspecific and repetitive elements in genomic DNA. We adjusted the final hybridization volume (40 μl) to contain 3.5× SSC and 0.3% SDS. Following sample denaturation (2 min at 100 °C) and a *Cot-1* preannealing step (20 min at 37 °C), we cohybridized test and reference samples to a cDNA microarray containing 39,711 nonredundant cDNA clones, representing 29,619 different human genes. Samples were hybridized at 65 °C for 16 h. Following hybridization, arrays were washed in 2× SSC, 0.03% SDS for 5 min at 65 °C, followed by successive washes in 1× and 0.2× SSC for 5 min each at room temperature.

### aCGH Data Analysis

Individual microarrays were imaged with a GenePix 4000B scanner (Axon Instruments, Union City, California, United States) and fluorescence intensities were extracted using GenePix Pro 3.0 software and uploaded into the Stanford Microarray Database (SMD) (http://genome-www5.stanford.edu) for analysis. For each experiment, fluorescence ratios were normalized by setting the average log_2_ fluorescence ratio for all array elements equal to 0. We included for analysis only those genes that were reliably measured (i.e., fluorescence intensity/background of greater than 1.4 in the reference channel) in greater than or equal to 50% of samples. Genes not meeting these criteria were viewed as absent. Map positions for cDNA clones on the array were assigned using the UCSC GoldenPath assembly (http://genome.ucsc.edu/), November 2002 freeze. Gene copy number ratios were visualized in log_2_ colorimetric scale with the genes ordered by chromosomal position using TreeView version 1.6 (http://rana.lbl.gov/EisenSoftware.htm). To provide the most accurate depiction of chromosomal gene distribution, cDNAs with multiple genome map positions (more than 1 Mb apart) were represented in TreeView at each assigned map location.

### Selection Criteria Applied to cDNA aCGH Data

#### **Genes showing copy number variation specific to a single hominoid lineage**

For selection of LS cDNAs, the values considered were the log_2_ of the aCGH fluorescence ratio of the test and reference genomic DNAs. Selection of LS cDNAs was based on the following criteria: First, for a given cDNA and a given species, no more than one value out of the species versus human comparisons for that species could be absent (see aCGH methods regarding absent signals). Second, for a gene copy number change to be considered unique to a particular species, at least half of the absolute values of comparisons within that species had to meet or exceed a threshold of 0.5 with all such values in the same direction, i.e., either all positive or all negative, and at least half of the absolute values of comparisons within each of the remaining species had to be below a threshold of 0.5. For example, for a gorilla LS gene, at least half of the gorilla comparisons had to meet or exceed the 0.5 threshold, while at least half of the comparisons within each of the remaining species had to be below the threshold. Third, in order to compensate for missing (i.e., “absent”) values for a given cDNA of all “present” values within each species, no more than one could fall below the threshold (0.5) for each species. Fourth, to ensure sufficiently high signal-to-noise in the identification of altered ratios, for a given cDNA and given great ape species, each absolute value of the average of the species versus human comparison for that species had to be at least 2.5-fold greater than the absolute value of each remaining species average, including human versus human comparisons. For HLS genes, the absolute value of each species average of the great ape versus human comparisons had to be at least 2.5-fold greater than the average of the absolute value of the human versus human comparisons.

#### **Genes showing copy number variation unique to more than one hominoid lineage**

For cDNAs in which the copy number was either increased or decreased in two or more hominoids relative to all the other hominoids, the same criteria were used as before, except the cDNA would have to meet or exceed the 0.5 threshold selection criteria for more than one species.

#### **Relationship of aCGH signal to gene copy number**

It is difficult to establish a precise relationship between gene copy number and interhominoid aCGH ratio because sequence divergence can influence hybridization signal strength and the sequences of additional gene copies are, in almost all cases, not known. However, prior studies by Pollack et al. (1999) showed that, using cell lines containing increasing numbers of X chromosomes, copy number, and aCGH signal exhibited a linear relationship over the copy number range tested, with an increase of a single gene copy corresponding to a ratio of 1.31 (log_2_ value = 0.39). In a similar manner, we took advantage of the fact the one of the human-to-human comparisons used in our experiments was between a male and female. In this context, X chromosome genes in the female should be present as two copies while in the male will exist as one copy. Calculation of the average aCGH ratios of 957 such genes in the male/female comparison yielded a log_2_ value of 0.21. The different values obtained in these two tests may reflect the fact that in the male/female comparison a Y chromosome was present, while this was not true in the other study, which used XO cell lines. The presence of sequences on the Y that are shared with the X could have produced a compression of aCGH fluorescence ratio values, accounting for the difference in X chromosome-related log_2_ ratios described above. Similar compression effects on X chromosome ratios have recently been reported (Snijders et al. 2001). While both the 0.39 and 0.21 values fall below the 0.5 threshold we employed for the selection of LS genes, 0.5 was used to insure that selection of false positives was minimized. In an interhominoid aCGH study, Locke et al. (2003) also determined a threshold of 0.5 to be most appropriate. Finally, the use of this relatively conservative threshold implies that the numbers presented here are likely to be underestimates of the actual number of genes that exhibit LS copy number differences between these hominoids.

### FISH Analysis

Using standard procedures, metaphase spreads and interphase nuclei were prepared from human lymphocytes (Homo sapiens [HSA]) and from great ape fibroblast cell lines, obtained from Coriell. The four great ape species studied were bonobo (Pan paniscus [PPA], Coriell #AG05253A), chimpanzee (Pan troglodytes [PTR], Coriell #AG06939A), lowland gorilla (Gorilla gorilla [GGO], Coriell #AG05251B), and Sumatran orangutan (Pongo pygmaeus [PPY], Coriell #AG12256).

One BAC clone (CTD-2288G6) containing all or portions of the coding regions for *OCLN*, *GTF2H2*, and *BIRC1* was selected as a probe for the region with increased copy number in human. A second BAC clone (RP11-1077O1) flanking the region amplified in human and containing portions of the *RAD17* gene was selected as a control probe. BAC clones were obtained from BACPAC Resources at the Children's Hospital Oakland Research Institute and from Research Genetics. Whole-cell PCR was done to verify that the *OCLN*, *GTF2H2*, and *BIRC1* genes were on BAC CTD-2288G5 and that the *RAD17* gene was on BAC RP11-1077O1. BAC DNAs were prepared using Large Construct Kits (Qiagen). BAC probes were directly labeled with Spectrum Green (Vysis, Downers Grove, Illinois, United States) and Spectrum Orange (Vysis) using the Vysis Nick Translation Kit and protocol.

FISH analyses with the BAC probes were performed using standard techniques. *Cot-1* DNA was used to block cross-hybridization of high-copy repeat sequences. In each experiment, dual-color hybridization was performed using a probe carrying genes with a predicted increase in copy number specifically in the human lineage (CTD-2288G6 or CTC-790E5) and a flanking probe (RP11-1077O1 or RP11-1113N2) containing a gene not predicted to show an HLS increase in copy number. For each species, two separate hybridizations were performed: one with the probe containing the genes showing increased human copy number labeled with Spectrum Green and the flanking probe with Spectrum Orange, and the other in which the dyes were reversed. For each probe combination for each species, a minimum of 200 interphase nuclei and ten metaphase spreads were examined. A whole chromosome painting probe for human Chromosome 5 (wcp5; Vysis) was used to confirm the gorilla Chromosome 19 to be syntenic with the human Chromosome 5 for the region of interest.

The hominoid cell lines used for FISH analysis were grown asynchronously in monolayer culture. Metaphase spreads and nuclei were obtained from a shake-off preparation and thus were somewhat selected for proliferative activity. Similarly, human lymphocyte cultures stimulated with the mitogen phytohemagglutinin contain cells in various stages of the cell cycle. In order to judge the replication state of the nuclei scored, dual-color FISH assays included probes both for DNA sequences that by aCGH showed copy number difference between test and reference DNA and for sequences on the same human chromosome that had the same (diploid) number of copies. Nuclei that showed diploid copy number of this control probe were assessed to be in G_0_. Nuclei that were in S/G_2_ demonstrated four copies of the control probe and the test probes were proportionately in multiple copies of the number established in the nonproliferating cells. Similar experimental conditions were used for the additional BAC FISH analyses described.

### Comparison of HLS Gene and WSSD Datasets

Sequences of IMAGE clones for each HLS gene were obtained using NCBI's Entrez (http://www.ncbi.nlm.nih.gov/Entrez) sequence retrieval tool and saved locally in FASTA format. Likewise, the random IMAGE clone sequences were obtained by first downloading GI numbers for all human IMAGE clones and then using a random number generator to pick approximately 200 random IMAGE clones from the list of GI numbers. These random IMAGE clone sequences were then downloaded from Entrez in a similar fashion. The April 2002 WSSD dataset was downloaded from the Segmental Duplication Database website (http://humanparalogy.gene.cwru.edu/SDD/). The two IMAGE clone sequence datasets were formatted and “BLASTed” against the WSSD sequences locally using NCBI's stand-alone BLAST executables for Windows. BLASTs were limited to an expect value of e^−20^ and then the best match was reported by a Perl (http://activestate.com/) script for each query. No restrictions on percent identity of the match or match length were imposed.

### HLS Gene Repeat Analysis

The HLS gene IMAGE clone sequences (see Table S4) were compared to the November 2002 build at UCSC using Dr. Jim Kent's BLAT program via the Human Genome Browser Gateway website (http://genome.ucsc.edu/cgi-bin/hgGateway). The BLAT hits were parsed such that only hits with a percent identity greater than or equal to 90% were reported. Furthermore, only hits with a match coverage (match length/query length) greater than or equal to 50% were reported.

Repeat annotation was downloaded from UCSC (http://genome.ucsc.edu/goldenPath/14nov2002/database/). Using the position data obtained from the BLAT alignments along with a 50 kb buffer on both sides of the alignments, the relative repeat content was determined for each HLS gene region using a Perl script. As a comparison, the relative repeat content was determined for the entire genome. Annotated gaps within the regions and the human genome were subtracted from the percent content calculation so that these content values were not skewed by gaps. Only long interspersed nuclear element (LINE), long terminal repeat (LTR), short interspersed nuclear element (SINE), Simple, and Satellite classes of repeats were included in the analysis.

### LS Gene Cluster Repeat Analysis

The 23 clusters of LS genes were compared to the human repeat database downloaded from UCSC (see HLS gene repeat analysis). Likewise, the Satellite repeat content for the LS genes within the 23 clusters was also determined in a similar fashion.

### GO Analysis of HLS and LS Genes

Primary GenBank accession numbers associated with both the HLS and LS gene lists were parsed into separate lists and stored as tab delimited text files. GenBank accession numbers were used as unique identifiers, and gene lists were annotated and functionally characterized using DAVID (Database for Annotation Visualization and Integrated Discovery) (http://apps1.niaid.nih.gov/david/upload.asp) (Dennis et al. 2003). Analyses were performed at level one for DAVID and at a threshold cutoff of 1, which provides high coverage but relatively low specificity and considers all classifications. Analysis was carried out on both lists, first using those genes with GenBank accession numbers, and then only those genes with known gene symbols. The analysis based on gene symbols recapitulates the analysis based on GenBank accessions, but contains correspondingly fewer classified genes.

In order to make meaningful comparisons between the LS genes, we identified and the entire genome, a nonredundant list of genome-wide UniGene numbers was adapted from EASE2.0 (Expression Analysis Systematic Explorer, http://apps1.niaid.nih.gov/david/) (Hosack et al. 2003), a program that facilitates the biological interpretation of gene lists. This tab-delimited text file, containing 33,655 unique UniGene numbers (updated 2 February 2004), was then uploaded to DAVID for GO analysis. The results for the molecular function analysis are graphically represented in Figure S4 and summarized in Table S5.

GenBank accession numbers were used for the HLS and LS analysis due to nearly half of the genes lacking UniGene numbers, thus making GenBank accession numbers more inclusive of the entire HLS and LS dataset analysis. Alternatively, UniGene numbers were used for the the genome-wide analysis because they provide a nonredundant dataset which is a much closer estimate to the number of genes (33,655) in the human genome versus the human RefSeq accession numbers. When subtracting all computer-based models from human RefSeq, only 20,850 RefSeq accession numbers were available for analysis.

### Human versus Chimpanzee Comparison

The HLS dataset is identical to that previously described. The random dataset chosen for this analysis was determined from UCSC's all_est annotation (http://genome.ucsc.edu/goldenPath/gbdDescriptions.html). From the all_est file, 200 random IMAGE clones were picked to ensure that at least one EST per IMAGE clone would map to the human genome. The EST sequences for both the HLS and random datasets were downloaded from GenBank and compared to the July 2003 human genome via a locally installed version of BLAT. BLAT output was parsed so that hits with a score greater than 200 and percent identity greater than 90% were examined for chimpanzee homology. The score and percent identity calculations mimic the calculations performed with the Web-based version of BLAT (http://genome.ucsc.edu/cgi-bin/hgBlat); the formula for these calculations was provided by Donna Karolchik.

The BLAT hits, as defined as one or more blocks of alignment within score and percent identity cutoffs, were compared to the chimpanzee versus human reciprocal best chain alignment annotation (http://genome.ucsc.edu/goldenPath/hg16/versusPt0/). For each BLAT hit, each block of alignment was compared to the chimpanzee versus human best chain annotation and was scored as follows: “chimp positive” indicates the block is entirely homologous to chimp; “chimp partial” indicates the block is partially homologous to chimp but there are gaps in the homology; “chimp gap” indicates the block is within a gap of the chimp homology; “chimp negative” indicates the block has no homology to chimpanzee. The summary numbers are based on all of the blocks of alignments and how they are scored in reference to chimpanzee homology.

The HLS dataset was compared to the “human > chimp” dataset by IMAGE clone identifiers. The “human > chimp” dataset is a redundant set that was not UniGene collapsed; thus, a redundant, non-UniGene collapsed HLS dataset was used for the comparison.

### RT-PCR Analysis

RT-PCR analysis of interhominoid DNA copy number variation was carried out using an ABI Prism 7700 sequence detector (Perkin Elmer Corporation/Applied Biosystems [PE/ABI], Torrance, California, United States) (Livak et al. 1995; Heid et al. 1996). Exon-specific primers and probe for *PLA2G4B*, *FLJ31659*, *BC040199*, and *CFTR* genes/cDNAs were designed with the assistance of the Prism 7700 sequence detection software (Primer Express, PE/ABI). The following primer/probe sequences were used: *PLA2G4B* F 5′-GCAGGTCTGGGTGAGGGTT-3′, *PLA2G4B* R 5′-GCTGCACCTGATCCCCACT-3′, and the probe 5′-VIC-CAGGAAGTTGCCACACAGGTGAGCA-TAMRA-3′; *FLJ31659* F 5′-GCTCAGACATCCAGGGACGA-3′, *FLJ31659* R 5′-CGCTTCTCCCAGGATTGGT-3′, and the probe 5′-VIC-CACATTCGTCCAACAGCGGTCGC-TAMRA-3′; *BC040199* F 5′-GAGGAAGGTTGGGTGTGGAG-3′, *BC040199* R 5′-ACTGGGTGTCCTGCTGGCT-3′, and the probe 5′-VIC-TTGCTTGCTGTGGCCCCAAGCT-TAMRA-3′; *CFTR* F 5′-CGCGATTTATCTAGGCATAGGC-3′, *CFTR* R 5′-TGTGATGAAGGCCAAAAATGG-3′, and the probe 5′-6FAM-TGCCTTCTCTTTATTGTGAGGACACTGCTCC-TAMRA-3′.

Amplification reactions were performed in MicroAmp optical tubes (PE/ABI) in a 50 μl mix containing 8% glycerol, 1× TaqMan buffer A (500 mM KCl, 100 mM Tris-HCl, 0.1 M EDTA, 600 nM passive reference dye ROX [pH 8.3 at room temperature]), 300 μM each of dATP, dGTP, and dCTP and 600 μM dUTP, 5.5 mM MgCl_2_, 900 nM forward primer, 300 nM reverse primer, 200 nM probe, 1.25 U AmpliTaq Gold DNA polymerase (PE/ABI), and the template genomic DNA. Thermal cycling conditions were as follows: activation of TaqGold at 95 °C for 10 min followed by 40 cycles of amplification at 95 °C for 15 s and 60 °C for 1 min.

After amplification, data analysis was carried out using a ratio of test gene to reference gene to obtain a normalized copy number estimate of the test gene (Bieche et al. 1998). The starting copy number in the template DNA was determined by the threshold cycle (C_t_), which represents the PCR cycle at which an increase in reporter fluorescence above a baseline signal can first be detected. The starting copy number of each test gene was quantified in the ape samples by determining the C_t_ of the test gene and using a standard curve for copy number. The standard curve for each gene was generated using the fluorescent data from five serial dilutions of human genomic DNA and calculating a single copy of each gene per haploid human genome, as annotated in the current genome build. Copy numbers of the test genes in ape samples were normalized to the copy number of the *CFTR* gene, which serves as a control representative of a single gene per haploid genome (Rochette et al. 2001). The ratio “N” of the test gene copy number to *CFTR* copy number in each sample normalized the results with respect to differing starting quantity and quality of the template DNA in each reaction (Bieche et al. 1998). Thus, “N” expresses the estimated copy number for each species using the derived standard curve and normalized to *CFTR*. RT-PCRs were carried out in triplicate for each gene in each species except human, in which five reactions were carried out for each gene to generate the standard curve.

## Supporting Information

Figure S1 BAC FISH Analysis of Gene Predicted to Be Reduced Only in Orangutan FISH images of BAC probe RP11-93K3 containing sequences from IMAGE cDNA clone 1882505.
(A) Normal human control, PHA-stimulated lymphocytes. Multiple (10–12) signals present: 9p12, 9q12, 4q arm, and two acrocentric p arm regions. The Chromosome 9 signals appear to flank the 9q heterochromatin and centromere regions, with the p arm signal a double signal.
(B) Bonobo fibroblast culture. Multiple (10–12) signals.
(C) Chimpanzee fibroblast culture. Multiple (10–12) signals.
(D) Gorilla fibroblast culture. Multiple (12–15) signals.
(E) Orangutan fibroblast culture. Two signals present on a pair of homologues (i.e., single copy in haploid genome). Also shown is a TreeView image (pseudocolor scale indicated) for IMAGE cDNA clone 1882505.
(3.29 MB EPS).

Figure S2 TreeView Image of cDNAs Selected Using Relaxed HLS Criteria Figure shows a TreeView image of blocks of HLS genes selected using increasingly relaxed selection criteria. The top-most group represents HLS genes selected using the standard 0.5 cutoff value described in Materials and Methods, while successive groups (separated by gray bars) represent additional cDNAs that were selected as the cutoff was progressively reduced to 0.45, 0.4, 0.35, and 0.3.
(1.9 MB EPS).

Figure S3 FISH Analysis with BAC Probe RP11-432G15 Containing the *FLJ22004* Gene (A) Normal human control, PHA-stimulated lymphocytes. Signal at Chromosome 2q13 and 22qtel.
(B) Bonobo fibroblast culture. Four signals.
(C) Chimpanzee fibroblast culture. Four signals.
(D) Gorilla fibroblast culture. More than 30 signals. Hybridization to most subtelomeric regions.
(E) Orangutan fibroblast culture. No apparent red signal. Probe BAC RP11-1007701 in green included as internal hybridization control. Also shown are aCGH TreeView images (pseudocolor scale indicated) for three *FLJ22004* cDNAs.
(6.94 MB EPS).

Figure S4 GO Categories Pie graphs showing the distribution of GO molecular function categories within HLS, LS, and whole genome gene lists. The top 22 categories are named in the legend in descending order of representation for all three groups. Categories were ranked by normalizing each category value for HLS and LS lists to the genome-wide list and then ranking the sum of these values for each category. Less well-represented categories were omitted from the graphs in order to enhance legibility, and zero values are not listed.
(1.1 MB EPS).

Figure S5 Interhominoid RT-PCR Analysis RT-PCR was used to provide an independent confirmation of interspecies cDNA aCGH data for three genes in which aCGH signals were different in the African great apes compared to human and orangutan. The chromosomal location, IMAGE clone ID, and GenBank accession numbers are provided for each cDNA. The species average log_2_ ratios for each cDNA clone and the copy number ratio of the test gene to the *CFTR* (control) gene, as determined by RT-PCR, are shown for the indicated species. Also shown are TreeView images of interhominoid aCGH results for the indicated cDNAs and a graphical depiction of the correlation between aCGH signal and copy number ratio to *CFTR* (RT-PCR).
(A) *PLA2G4B* cDNA clone located on human Chromosome 15 using the UCSC November 2002 human genome assembly. The correlation between RT-PCR and aCGH-based copy number estimates is 0.94.
(B) *FLJ31659* cDNA clone located on human Chromosome 4 using the UCSC November 2002 human genome assembly. As in (A), the correlation between RT-PCR and aCGH data is 0.97.
(C) *BC040199* transcript located on human Chromosome 7 using the UCSC November 2002 human genome assembly. As in (A), the correlation between RT-PCR and aCGH data is 0.97.
(1.29 MB EPS).

Figure S6 FISH Analysis with BAC Probe RP11–23P13 Containing the Human *PLA2G4B* and *SPTBN5* Genes (A) Normal human control, PHA-stimulated lymphocytes. Two signals localized to Chromosome 15q15.1.
(B) Chimpanzee fibroblast culture. Two signals on the chromosome syntenic to human Chromosome 15 (at arrows). Multiple additional signals in the subtelomeric regions.
(C) Gorilla fibroblast culture. Two signals on the chromosome syntenic to Chromosome 15 (at arrows). Two additional signals on a large metacentric chromosome, which in interphase appear as amplified signals.
(D) Orangutan fibroblast culture. Two signals on the chromosome syntenic to human Chromosome 15.
(4.39 MB EPS).

Protocol S1 How to View aCGH Data Using TreeView (2 KB TXT).

Table S1 Genome-Wide Interhominoid cDNA aCGH Gene Dataset Values are provided for genes (cDNAs) queried by interhominoid aCGH. For each IMAGE clone queried, log_2_ aCGH values are listed for the human versus human samples (*n* = 5), human versus bonobo samples (*n* = 3), human versus chimpanzee (*n* = 4), human versus gorilla (*n* = 3), and human versus orangutan (*n* = 3). This table is tab-delimited and can be opened in Microsoft Excel to view the raw numbers or can be browsed using TreeView (see Protocol S1). Column B provides information regarding IMAGE clone number, chromosome, and nucleotide position (UCSC November 2002 freeze), Unigene ID, EST accession numbers, and known gene information.
(12.84 MB TXT).

Table S2 Detailed Comparison of HLS Gene and WSSD Datasets For each IMAGE clone of the HLS genes, one or more EST sequences were used as a query for a BLAST search against the WSSD dataset. An expect value cutoff of e^–20^ was used and the best hit is reported in the table. Query refers to the HLS gene EST sequences; subject refers to the WSSD sequences. Score, expect value, and percent identity (ID) are reported for the best BLAST hit, while the start and stop positions and length for both query and subject are also reported.
(434 KB DOC).

Table S3 Satellite Repeat Subclass Analysis for LS Gene Clusters For each of the 23 LS gene clusters, Satellite repeat subclass analysis was performed. The table lists the cluster's cytogenetic region, the chromosome and start and stop positions, and the adjusted length after accounting for gaps in the genomic sequence. The percent content for 24 subclasses of Satellite repeats is listed for each of the 23 gene clusters. Summary information includes the average content of the 24 subclasses of Satellite repeats for all of the clusters as well as the average for the entire human genome. The difference and fold change are calculated based on comparing the cluster averages to the entire human genome averages.
(111 KB DOC).

Table S4 LS Gene Datasets Similar to Table S1, but only IMAGE clones with LS characteristics are listed, and each is ranked based on average fluorescence signal (highest to lowest) within each lineage.
(269 KB TXT).

Table S5 GO Analysis Comparing HLS and LS Genes to the Whole Genome (52 KB DOC).

Table S6 Functional Assessment of Copies of HLS Genes Presented are pertinent data from GO analysis with DAVID, including numbers of classified and unclassified genes in each gene list, as well as the data returned for each of the 22 most represented molecular function categories. Listed are GO identification numbers (GOIDs) and names for each of the top 22 categories, as well as raw values and relative percent values for HLS, LS, and genome classifications. Relative percent columns are taken as the ratio of the number of classifications in each category to the number of genes classified in the list. The average percent is also provided as the average of these relative percent values across the three groups. This is intended as a metric to help gauge deviations in group relative percent values from the combined average value.
(81 KB DOC).

Table S7 Comparison of Human HLS Genes to Chimpanzee Genomic Sequence The table has three sections: a summary showing the percentages of blocks in each respective chimpanzee homology scoring class; a table with the HLS versus chimpanzee data; and a table with the random versus chimpanzee data. The HLS versus chimpanzee and random versus chimpanzee tables have columns derived from both parsing the BLAT PSL data and from the chimpanzee homology comparison. The table lists the IMAGE clone and the EST accession number used as a query, the hit number, the score and percent identities, the start and stop positions in the query, the chromosome and chromosome start and stop positions, the number of blocks of alignment for the hit, the numbers of blocks that fall into each chimpanzee homology scoring class, and finally the respective chimpanzee scaffold(s) for each hit, if available.
(3.58 MB DOC).

### Accession Numbers

The GenBank accession number (http://www.ncbi.nlm.nih.gov/Genbank/) for *FGF7* is NM_002009 and for *morpheus* is AF132984.

## Abbreviations

aCGH: array-based comparative genomic hybridization
BAC: bacterial artificial chromosome
BLAST: basic local alignment search tool
BLAT: BLAST-like alignment tool
CGH: comparative genomic hybridization
DAVID: Database for Annotation Visualization and Integrated Discovery
EST: expressed sequence tag
FISH: fluorescence in situ hybridization
GABA: γ-aminobutyric acid
GGO: Gorilla gorilla (gorilla)
GO: Gene Ontology
HLS: refers to genes that show interhominoid aCGH signals indicative of a human lineage-specific variation in copy number
HSA: Homo sapiens (human)
IMAGE: integrated molecular analysis of genomes and their expression
LINE: long interspersed nuclear element
LTR: long terminal repeat
LOD score: log base 10 of the likelihood ratio under the hypotheses of linkage and nonlinkage
LS: refers to genes that show interhominoid aCGH signals indicative of a lineage-specific variation in copy number
MYA: million years ago
NAIP: neuronal apoptosis inhibitory protein
PECN: published estimate of gene copy number
PPA: Pan paniscus (bonobo)
PPY: Pongo pygmaeus (orangutan)
PTR: Pan troglodytes (chimpanzee)
RT-PCR: real-time PCR
SINE: short interspersed nuclear element
SMA: spinal muscular atrophy
SMD: Stanford Microarray Database
WSSD Database: Whole Genome Shotgun Segmental Duplication Database
