# Supplementary material for: Lineage-Specific Gene Duplication and Loss in Human and Great Ape Evolution
Source: PLoS Biol. 2004 Jul 13;2(7):e207. doi: 10.1371/journal.pbio.0020207 (PMC449870; doi:10.1371/journal.pbio.0020207)
Supplement: Table S2 — For each IMAGE clone of the HLS genes, one or more EST sequences were used as a query for a BLAST search against the WSSD dataset. An expect value cutoff of e–20 was used and the best hit is reported in the table. Query refers to the HLS gene EST sequences; subject refers to the WSSD sequences. Score, expect value, and percent identity (ID) are reported for the best BLAST hit, while the start and stop positions and length for both query and subject are also reported. (434 KB DOC). [file pbio.0020207.st002.doc]

| Table S1: Detailed comparison of HLS gene and WSSD datasets | | | | | | | | | | | | | |
| --- | --- | --- | --- | --- | --- | --- | --- | --- | --- | --- | --- | --- | --- |
| **NUM** | **CLONE** | **QUERY** | **QUERY LEN** | **SUBJECT** | **SUBJECT LENGTH** | **SCORE** | **E VALUE** | **ID** | **QUERY START** | **QUERY STOP** | **SUBJECT START** | **SUBJECT STOP** | **MATCH LENGTH** |
| 1 | IMAGE:1566212 | AI066560 | 489 | AL158816 | 14563 | 902 | 0 | 97 | 4 | 489 | 4443 | 4928 | 486 |
| 2 | IMAGE:1626299 | AI005134 | 432 | AC074386 | 67845 | 829 | 0 | 99 | 3 | 432 | 20702 | 21131 | 430 |
| 3 | IMAGE:50904 | H19234 | 442 |  |  |  |  |  |  |  |  |  |  |
|  |  | H19233 | 495 |  |  |  |  |  |  |  |  |  |  |
| 4 | IMAGE:843276 | AA486041 | 443 | AC023990 | 15152 | 327 | 2.00E-88 | 98 | 90 | 266 | 1203 | 1379 | 177 |
|  |  | AA488658 | 491 | AL355800 | 3875 | 351 | 1.00E-95 | 100 | 47 | 223 | 398 | 574 | 177 |
| 5 | IMAGE:1877990 | AI275888 | 402 | AC068255 | 20180 | 773 | 0 | 99 | 1 | 402 | 1260 | 1661 | 402 |
| 6 | IMAGE:279874 | BX093390 | 648 | AC036220 | 7857 | 1055 | 0 | 95 | 1 | 648 | 4887 | 5542 | 656 |
|  |  | N45002 | 427 | AC036220 | 7857 | 603 | e-171 | 97 | 1 | 341 | 5201 | 5542 | 342 |
|  |  | N40992 | 439 | AC036220 | 7791 | 392 | e-108 | 95 | 193 | 426 | 5381 | 5617 | 237 |
| 7 | IMAGE:1856246 | BX118334 | 469 | AC064811 | 10810 | 722 | 0 | 94 | 1 | 469 | 3880 | 4349 | 470 |
|  |  | AI240359 | 463 | AC064811 | 10810 | 827 | 0 | 97 | 2 | 461 | 4366 | 4832 | 467 |
| 8 | IMAGE:1699118 | AI792327 | 504 | AL354822 | 2278 | 944 | 0 | 99 | 14 | 504 | 987 | 1477 | 491 |
|  |  | AI733556 | 482 | AL354822 | 2278 | 946 | 0 | 99 | 2 | 482 | 1207 | 1687 | 481 |
|  |  | AI003508 | 490 | AL354822 | 2278 | 946 | 0 | 99 | 2 | 490 | 1200 | 1687 | 489 |
| 9 | IMAGE:365515 | BX094084 | 709 | AP001896 | 9933 | 438 | e-121 | 96 | 329 | 581 | 7371 | 7623 | 253 |
|  |  | AA009609 | 493 | AL356136 | 6603 | 513 | e-144 | 97 | 1 | 280 | 564 | 843 | 280 |
|  |  | AA009608 | 504 | AL163539 | 19678 | 163 | 7.00E-39 | 96 | 227 | 332 | 8742 | 8845 | 106 |
| 10 | IMAGE:796303 | AA461307 | 431 | AC103539 | 17830 | 813 | 0 | 98 | 1 | 431 | 491 | 921 | 431 |
|  |  | AA459724 | 365 | AC103539 | 17830 | 676 | 0 | 98 | 4 | 365 | 415 | 776 | 362 |
| 11 | IMAGE:814792 | AA465611 | 416 | AP000533 | 46503 | 654 | 0 | 96 | 18 | 414 | 42428 | 42824 | 397 |
|  |  | AA455233 | 444 | AP000534 | 38771 | 541 | e-153 | 95 | 1 | 336 | 665 | 1001 | 337 |
| 12 | IMAGE:1856923 | AI271431 | 412 | AL357493 | 81407 | 793 | 0 | 99 | 1 | 412 | 55181 | 55592 | 412 |
| 13 | IMAGE:1683035 | AI088089 | 190 | AL356136 | 6603 | 250 | 1.00E-65 | 97 | 53 | 190 | 3565 | 3702 | 138 |
| 14 | IMAGE:135010 | BX116199 | 537 | AC026106 | 1108 | 480 | e-134 | 98 | 104 | 357 | 211 | 464 | 254 |
|  |  | R32361 | 253 | AC026106 | 1108 | 230 | 2.00E-59 | 97 | 1 | 128 | 66 | 193 | 128 |
|  |  | R31645 | 336 | AC026106 | 1108 | 256 | 4.00E-67 | 94 | 104 | 316 | 261 | 464 | 213 |
| 15 | IMAGE:755093 | AI821682 | 715 | AC012369 | 14207 | 1215 | 0 | 96 | 9 | 715 | 1486 | 2191 | 707 |
|  |  | AI820932 | 420 | AC012369 | 14207 | 775 | 0 | 97 | 1 | 420 | 3215 | 3634 | 420 |
|  |  | AA482660 | 361 | AC012369 | 14207 | 636 | 0 | 97 | 15 | 360 | 3289 | 3634 | 346 |
|  |  | AA482508 | 512 | AC012369 | 14207 | 930 | 0 | 98 | 16 | 512 | 1491 | 1987 | 497 |
| 16 | IMAGE:298685 | BX105439 | 664 | AC008993 | 40936 | 1193 | 0 | 97 | 1 | 664 | 29238 | 29911 | 674 |
|  |  | W05240 | 326 | AC008993 | 40936 | 529 | e-149 | 98 | 1 | 311 | 29238 | 29543 | 311 |
|  |  | N74332 | 287 | AC008993 | 40936 | 454 | e-127 | 96 | 1 | 267 | 29769 | 30040 | 272 |
| 17 | IMAGE:136933 | R39745 | 418 |  |  |  |  |  |  |  |  |  |  |
|  |  | R36662 | 414 | AC008993 | 40936 | 511 | e-144 | 97 | 1 | 302 | 36784 | 37081 | 302 |
| 18 | IMAGE:1474402 | AI791559 | 429 | AC076959 | 33000 | 726 | 0 | 96 | 1 | 429 | 7887 | 8314 | 429 |
|  |  | AI733069 | 474 | AL158816 | 14563 | 454 | e-126 | 93 | 128 | 460 | 10492 | 10835 | 344 |
|  |  | AA922384 | 269 | AL158816 | 14563 | 454 | e-127 | 100 | 1 | 229 | 10720 | 10948 | 229 |
| 19 | IMAGE:1467026 | AA883127 | 452 | AC024002 | 9810 | 825 | 0 | 97 | 4 | 452 | 910 | 1358 | 449 |
| 20 | IMAGE:79581 | T62861 | 298 | AC068881 | 70320 | 551 | e-156 | 99 | 1 | 286 | 67499 | 67783 | 286 |
|  |  | T62711 | 301 | AC068881 | 70320 | 408 | e-113 | 95 | 5 | 296 | 67502 | 67783 | 292 |
| 21 | IMAGE:704320 | AA279467 | 406 | AC016767 | 37381 | 761 | 0 | 99 | 8 | 406 | 23039 | 23438 | 400 |
| 22 | IMAGE:238756 | H65193 | 358 | AL078463 | 144555 | 486 | e-136 | 96 | 1 | 305 | 136975 | 137276 | 305 |
|  |  | H64701 | 370 | AL078463 | 144555 | 632 | e-180 | 98 | 1 | 347 | 137073 | 137417 | 347 |
| 23 | IMAGE:384886 | AA708832 | 475 | AC068367 | 124028 | 926 | 0 | 99 | 1 | 475 | 38122 | 38596 | 475 |
| 24 | IMAGE:415089 | W94994 | 556 | AC009477 | 23726 | 373 | e-102 | 96 | 1 | 227 | 4273 | 4500 | 228 |
|  |  | W93379 | 398 | AC009477 | 23726 | 543 | e-153 | 92 | 11 | 398 | 3858 | 4250 | 393 |
| 25 | IMAGE:136324 | R34020 | 368 | AC068446 | 156020 | 355 | 6.00E-97 | 95 | 1 | 229 | 107538 | 107762 | 229 |
|  |  | R33482 | 405 | AC068446 | 156020 | 230 | 3.00E-59 | 92 | 1 | 168 | 107974 | 108146 | 173 |
| 26 | IMAGE:2029176 | AI793240 | 519 |  |  |  |  |  |  |  |  |  |  |
|  |  | AI793064 | 572 |  |  |  |  |  |  |  |  |  |  |
|  |  | AI253119 | 402 |  |  |  |  |  |  |  |  |  |  |
| 27 | IMAGE:1881469 | AI291184 | 468 | AC011244 | 6367 | 888 | 0 | 99 | 17 | 468 | 4053 | 4504 | 452 |
| 28 | IMAGE:298862 | BX114658 | 715 | AC002055 | 38065 | 1219 | 0 | 96 | 6 | 714 | 25183 | 25892 | 710 |
|  |  | W01652 | 375 | AC016629 | 14072 | 640 | 0 | 97 | 2 | 375 | 12228 | 12601 | 374 |
|  |  | N75356 | 241 | AF166490 | 8131 | 341 | 6.00E-93 | 94 | 30 | 241 | 5133 | 5345 | 213 |
| 29 | IMAGE:1706664 | BX098416 | 735 | AC098976 | 22614 | 1447 | 0 | 99 | 1 | 734 | 21639 | 22372 | 734 |
|  |  | AI148329 | 497 | AC116050 | 14079 | 969 | 0 | 99 | 1 | 497 | 8968 | 9464 | 497 |
| 30 | IMAGE:429093 | AA007587 | 394 | AL354697 | 8512 | 541 | e-153 | 97 | 3 | 306 | 1826 | 2128 | 304 |
|  |  | AA007586 | 413 | AL354822 | 14795 | 299 | 4.00E-80 | 96 | 103 | 277 | 10132 | 10306 | 175 |
| 31 | IMAGE:470261 | AA029012 | 230 | AL009030 | 8398 | 274 | 1.00E-72 | 97 | 81 | 230 | 2931 | 3080 | 150 |
|  |  | AA028921 | 169 | AC012369 | 60396 | 226 | 2.00E-58 | 93 | 1 | 147 | 14263 | 14408 | 147 |
| 32 | IMAGE:1874052 | AI339565 | 456 | AL158816 | 14563 | 866 | 0 | 100 | 20 | 456 | 3389 | 3825 | 437 |
| 33 | IMAGE:1468074 | AA889397 | 217 | AC012369 | 60396 | 406 | e-112 | 98 | 1 | 217 | 38790 | 39006 | 217 |
| 34 | IMAGE:109123 | BX098029 | 737 | AC017027 | 21839 | 1386 | 0 | 98 | 1 | 734 | 18534 | 19267 | 734 |
|  |  | T80979 | 387 | AC017027 | 21839 | 535 | e-151 | 97 | 3 | 327 | 18036 | 18355 | 325 |
|  |  | T80978 | 150 | AC017027 | 21839 | 281 | 3.00E-75 | 99 | 1 | 150 | 19072 | 19220 | 150 |
| 35 | IMAGE:1638749 | AI015610 | 527 | AC018865 | 18183 | 799 | 0 | 94 | 7 | 527 | 14750 | 15273 | 524 |
| 36 | IMAGE:281777 | BX108283 | 658 | AL357493 | 81407 | 716 | 0 | 99 | 289 | 653 | 61355 | 61719 | 365 |
|  |  | N53282 | 167 | AL357493 | 81407 | 283 | 8.00E-76 | 96 | 1 | 167 | 61119 | 61286 | 168 |
|  |  | N48085 | 462 | AL357493 | 81407 | 813 | 0 | 98 | 1 | 461 | 61488 | 61942 | 461 |
| 37 | IMAGE:824794 | AA489069 | 356 | AC099646 | 20691 | 575 | e-163 | 97 | 35 | 356 | 13970 | 14291 | 322 |
| 38 | IMAGE:1685642 | AI089407 | 613 |  |  |  |  |  |  |  |  |  |  |
| 39 | IMAGE:712622 | AA281797 | 318 | AC011244 | 17164 | 615 | e-175 | 99 | 1 | 318 | 5612 | 5928 | 318 |
| 40 | IMAGE:796775 | AA443157 | 527 | AC018593 | 8320 | 741 | 0 | 96 | 11 | 440 | 1930 | 2359 | 430 |
|  |  | AA443156 | 506 | AC017027 | 21839 | 1003 | 0 | 100 | 1 | 506 | 9347 | 9852 | 506 |
| 41 | IMAGE:809394 | AA456585 | 313 |  |  |  |  |  |  |  |  |  |  |
| 42 | IMAGE:1592675 | AI791777 | 191 | AC098976 | 22614 | 297 | 6.00E-80 | 96 | 18 | 191 | 15968 | 16141 | 174 |
|  |  | AI733462 | 173 | AC098976 | 22614 | 299 | 1.00E-80 | 97 | 3 | 173 | 15980 | 16150 | 171 |
|  |  | AA961653 | 141 | AC098976 | 22614 | 240 | 9.00E-63 | 96 | 1 | 141 | 15980 | 16120 | 141 |
| 43 | IMAGE:1930209 | AI312926 | 434 | AC011244 | 6367 | 846 | 0 | 99 | 1 | 434 | 2613 | 3047 | 435 |
| 44 | IMAGE:1634998 | BX090940 | 720 | AC067775 | 13497 | 1068 | 0 | 94 | 1 | 676 | 2183 | 2860 | 678 |
|  |  | AA994976 | 361 | AC067775 | 13497 | 438 | e-122 | 97 | 39 | 286 | 2612 | 2860 | 249 |
| 45 | IMAGE:345247 | W74030 | 405 | AC080000 | 19930 | 674 | 0 | 96 | 1 | 400 | 4820 | 5218 | 400 |
|  |  | W72369 | 600 | AC080000 | 19930 | 775 | 0 | 92 | 8 | 590 | 5663 | 6247 | 585 |
| 46 | IMAGE:191530 | H37860 | 502 | AC011244 | 17164 | 803 | 0 | 98 | 1 | 456 | 986 | 1435 | 456 |
|  |  | H37809 | 431 | AC011244 | 17164 | 728 | 0 | 97 | 10 | 431 | 815 | 1232 | 422 |
| 47 | IMAGE:823588 | AA497127 | 498 | AC079776 | 10731 | 569 | e-161 | 92 | 62 | 498 | 1177 | 1611 | 437 |
|  |  | AA497050 | 428 | AC079776 | 10731 | 511 | e-144 | 91 | 21 | 428 | 1148 | 1552 | 408 |
| 48 | IMAGE:768643 | AA430351 | 395 | AL359758 | 86058 | 767 | 0 | 99 | 1 | 395 | 45608 | 46002 | 395 |
|  |  | AA425630 | 422 | AL359758 | 86058 | 648 | 0 | 99 | 1 | 331 | 45211 | 45541 | 331 |
| 49 | IMAGE:1940641 | AI352281 | 329 | AP001896 | 29549 | 547 | e-155 | 96 | 2 | 329 | 7289 | 7613 | 328 |
| 50 | IMAGE:71432 | T47813 | 395 | AC091567 | 18297 | 365 | e-100 | 98 | 1 | 203 | 4596 | 4799 | 204 |
|  |  | T47812 | 432 |  |  |  |  |  |  |  |  |  |  |
| 51 | IMAGE:1641988 | BX117400 | 531 | AL445443 | 16093 | 476 | e-133 | 100 | 1 | 240 | 2550 | 2789 | 240 |
|  |  | AI018459 | 469 | AC060768 | 29310 | 819 | 0 | 97 | 2 | 469 | 5878 | 6346 | 469 |
| 52 | IMAGE:1557341 | AI792990 | 507 | AC008993 | 40936 | 422 | e-117 | 97 | 271 | 507 | 34126 | 34362 | 237 |
|  |  | AA935790 | 339 | AC008993 | 40936 | 551 | e-156 | 98 | 4 | 305 | 34126 | 34427 | 302 |
| 53 | IMAGE:823614 | AA496947 | 421 |  |  |  |  |  |  |  |  |  |  |
|  |  | AA490546 | 422 |  |  |  |  |  |  |  |  |  |  |
| 54 | IMAGE:1759573 | AI221541 | 298 | AC114498 | 17281 | 557 | e-158 | 98 | 4 | 298 | 7636 | 7931 | 296 |
| 55 | IMAGE:1534977 | AI822015 | 406 | AL356369 | 17476 | 620 | e-177 | 99 | 11 | 334 | 3531 | 3855 | 325 |
|  |  | AI792824 | 423 | AL356369 | 17476 | 599 | e-170 | 98 | 105 | 423 | 3537 | 3856 | 320 |
|  |  | AA918902 | 359 | AL356369 | 17476 | 668 | 0 | 99 | 5 | 359 | 3525 | 3881 | 357 |
| 56 | IMAGE:730649 | AA411761 | 359 | AL109948 | 15542 | 634 | 0 | 99 | 36 | 359 | 4692 | 5015 | 324 |
| 57 | IMAGE:900896 | BX101993 | 384 | AC027727 | 5928 | 716 | 0 | 99 | 1 | 369 | 4896 | 5264 | 369 |
|  |  | AA503815 | 364 | AC027727 | 5928 | 648 | 0 | 99 | 30 | 364 | 4930 | 5264 | 335 |
| 58 | IMAGE:297084 | W03793 | 482 | U74496 | 11124 | 839 | 0 | 97 | 1 | 482 | 5980 | 6457 | 482 |
|  |  | N73768 | 587 | U74496 | 11124 | 642 | 0 | 95 | 1 | 386 | 7081 | 7464 | 386 |
| 59 | IMAGE:969906 | AA663895 | 354 | AC027181 | 19371 | 579 | e-164 | 94 | 2 | 354 | 5991 | 6344 | 354 |
| 60 | IMAGE:743828 | BX098730 | 449 | AC023310 | 25455 | 852 | 0 | 99 | 1 | 434 | 12215 | 12648 | 434 |
|  |  | AA634379 | 156 | AC023310 | 25455 | 295 | 2.00E-79 | 99 | 4 | 156 | 12497 | 12649 | 153 |
| 61 | IMAGE:594758 | AA172236 | 393 | AC080000 | 23792 | 712 | 0 | 98 | 1 | 390 | 4734 | 5121 | 390 |
|  |  | AA172056 | 598 | AC080000 | 23792 | 811 | 0 | 95 | 2 | 535 | 4881 | 5425 | 545 |
| 62 | IMAGE:108471 | T80117 | 472 | AC074370 | 23583 | 523 | e-147 | 95 | 1 | 361 | 18714 | 19062 | 361 |
|  |  | T70234 | 304 | AL109948 | 17086 | 139 | 6.00E-32 | 94 | 133 | 225 | 596 | 687 | 93 |
| 63 | IMAGE:282884 | N45114 | 535 | AL360016 | 11748 | 480 | e-134 | 100 | 6 | 247 | 10509 | 10750 | 242 |
| 64 | IMAGE:811138 | AA486450 | 237 | AL135795 | 9648 | 333 | 1.00E-90 | 98 | 36 | 218 | 6122 | 6305 | 184 |
|  |  | AA485730 | 409 | AL135795 | 9648 | 708 | 0 | 96 | 1 | 409 | 3649 | 4062 | 414 |
| 65 | IMAGE:1461668 | BX109639 | 546 | AL445443 | 48102 | 640 | 0 | 100 | 209 | 531 | 18309 | 18631 | 323 |
|  |  | AA885313 | 473 | AL445443 | 48102 | 642 | 0 | 100 | 5 | 328 | 18309 | 18632 | 324 |
| 66 | IMAGE:795343 | AA453359 | 602 | AL109948 | 17932 | 539 | e-152 | 98 | 285 | 572 | 14126 | 14413 | 288 |
|  |  | AA453258 | 575 | AL109948 | 17932 | 813 | 0 | 98 | 3 | 436 | 15310 | 15743 | 434 |
| 67 | IMAGE:767345 | AA418633 | 596 | AC012369 | 60396 | 846 | 0 | 99 | 130 | 560 | 7365 | 7795 | 431 |
|  |  | AA418564 | 533 | AC012369 | 60396 | 995 | 0 | 98 | 8 | 533 | 7226 | 7751 | 526 |
| 68 | IMAGE:384872 | AA708826 | 373 |  |  |  |  |  |  |  |  |  |  |
| 69 | IMAGE:292567 | N91336 | 444 | AC069171 | 33474 | 551 | e-156 | 97 | 124 | 438 | 33164 | 33474 | 315 |
|  |  | N68492 | 462 | AC069171 | 33474 | 492 | e-138 | 97 | 1 | 299 | 33025 | 33317 | 299 |
| 70 | IMAGE:451095 | AA704519 | 372 | AC004534 | 33622 | 730 | 0 | 99 | 1 | 372 | 31779 | 32150 | 372 |
| 71 | IMAGE:1627621 | AI014703 | 395 | AL022240 | 24403 | 424 | e-118 | 98 | 166 | 395 | 4506 | 4735 | 230 |
| 72 | IMAGE:231802 | H92758 | 368 |  |  |  |  |  |  |  |  |  |  |
| 73 | IMAGE:1031047 | AA609881 | 393 | AC097374 | 22888 | 511 | e-144 | 98 | 14 | 283 | 9399 | 9668 | 270 |
| 74 | IMAGE:321470 | W44889 | 377 | AC079776 | 19169 | 369 | e-101 | 100 | 13 | 198 | 687 | 872 | 186 |
|  |  | W32303 | 417 | AC079776 | 10731 | 563 | e-159 | 97 | 16 | 326 | 1148 | 1458 | 311 |
| 75 | IMAGE:193990 | BX106160 | 761 | AC018348 | 33513 | 1114 | 0 | 95 | 1 | 683 | 24711 | 25394 | 684 |
|  |  | R83876 | 370 | AC018348 | 33513 | 200 | 2.00E-50 | 89 | 1 | 172 | 25413 | 25585 | 173 |
|  |  | R83875 | 455 | AC018348 | 33513 | 664 | 0 | 94 | 1 | 453 | 24710 | 25156 | 453 |
| 76 | IMAGE:745332 | BX115856 | 591 | AC027727 | 5928 | 692 | 0 | 99 | 216 | 576 | 3272 | 3632 | 361 |
|  |  | AA625642 | 379 | AC027727 | 5928 | 676 | 0 | 98 | 2 | 365 | 3272 | 3636 | 365 |
| 77 | IMAGE:1646649 | AI025974 | 377 | AL356136 | 28056 | 398 | e-110 | 99 | 14 | 218 | 16652 | 16856 | 205 |
| 78 | IMAGE:1030854 | AA621750 | 452 | AL136317 | 26125 | 785 | 0 | 99 | 5 | 408 | 16497 | 16900 | 404 |
| 79 | IMAGE:470930 | AA034103 | 216 |  |  |  |  |  |  |  |  |  |  |
|  |  | AA032090 | 312 | AC092821 | 50684 | 474 | e-133 | 95 | 1 | 298 | 1378 | 1677 | 300 |
| 80 | IMAGE:429109 | AA005047 | 323 |  |  |  |  |  |  |  |  |  |  |
|  |  | AA004801 | 521 |  |  |  |  |  |  |  |  |  |  |
| 81 | IMAGE:131316 | BX091384 | 720 | AL109948 | 13594 | 735 | 0 | 96 | 7 | 422 | 5367 | 5782 | 416 |
|  |  | R23055 | 344 | AL109948 | 13594 | 458 | e-128 | 95 | 2 | 285 | 5507 | 5787 | 284 |
|  |  | R22949 | 401 | AL109948 | 13594 | 482 | e-135 | 100 | 8 | 250 | 5085 | 5327 | 243 |
| 82 | IMAGE:252953 | H88599 | 400 |  |  |  |  |  |  |  |  |  |  |
|  |  | H88598 | 403 |  |  |  |  |  |  |  |  |  |  |
| 83 | IMAGE:119768 | T94500 | 296 | AC018593 | 79559 | 488 | e-137 | 98 | 1 | 265 | 60789 | 61053 | 265 |
|  |  | T94409 | 319 | AC018593 | 79559 | 620 | e-177 | 99 | 1 | 319 | 61019 | 61337 | 319 |
| 84 | IMAGE:162491 | H27752 | 417 | AL356218 | 10624 | 454 | e-126 | 94 | 18 | 339 | 3311 | 3625 | 322 |
| 85 | IMAGE:379670 | AA778039 | 435 |  |  |  |  |  |  |  |  |  |  |
| 86 | IMAGE:246820 | BX091415 | 737 | AC093526 | 8841 | 1306 | 0 | 96 | 3 | 737 | 7176 | 7910 | 735 |
|  |  | N59493 | 371 | AC093526 | 8841 | 472 | e-132 | 98 | 1 | 252 | 7604 | 7855 | 252 |
|  |  | N59089 | 377 | AC093526 | 8841 | 400 | e-110 | 99 | 1 | 210 | 6522 | 6730 | 210 |
| 87 | IMAGE:1641894 | BX090610 | 500 | AC016745 | 20154 | 115 | 1.00E-24 | 86 | 232 | 357 | 18055 | 18180 | 126 |
|  |  | AI018406 | 276 |  |  |  |  |  |  |  |  |  |  |
| 88 | IMAGE:730398 | BX103439 | 395 | AC096579 | 41305 | 513 | e-144 | 91 | 1 | 380 | 13226 | 13604 | 380 |
|  |  | AA470109 | 366 | AC096579 | 41305 | 498 | e-140 | 92 | 1 | 364 | 13226 | 13588 | 364 |
|  |  | AA469939 | 275 | AC073995 | 11967 | 367 | e-100 | 92 | 1 | 273 | 11631 | 11903 | 273 |
| 89 | IMAGE:307337 | W21055 | 462 | AP001896 | 29549 | 658 | 0 | 94 | 14 | 462 | 1409 | 1851 | 449 |
|  |  | N95226 | 610 | AL096772 | 18487 | 593 | e-168 | 92 | 56 | 492 | 6647 | 7082 | 437 |
| 90 | IMAGE:2461849 | AI935363 | 575 |  |  |  |  |  |  |  |  |  |  |
| 91 | IMAGE:251404 | H97969 | 422 |  |  |  |  |  |  |  |  |  |  |
| 92 | IMAGE:328821 | W45499 | 392 |  |  |  |  |  |  |  |  |  |  |
|  |  | W40422 | 364 |  |  |  |  |  |  |  |  |  |  |
| 93 | IMAGE:1557277 | AA935533 | 487 | AC013360 | 20096 | 942 | 0 | 99 | 1 | 487 | 10459 | 10945 | 487 |
| 94 | IMAGE:290337 | N92228 | 332 |  |  |  |  |  |  |  |  |  |  |
|  |  | N64494 | 439 |  |  |  |  |  |  |  |  |  |  |
| 95 | IMAGE:824758 | AA488998 | 429 |  |  |  |  |  |  |  |  |  |  |
|  |  | AA488782 | 460 |  |  |  |  |  |  |  |  |  |  |
| 96 | IMAGE:305677 | BX091389 | 206 | AC011244 | 17164 | 375 | e-103 | 98 | 11 | 206 | 15450 | 15645 | 196 |
|  |  | W19716 | 277 | AC011244 | 17164 | 541 | e-153 | 99 | 2 | 277 | 15381 | 15656 | 276 |
|  |  | N89973 | 411 | AC011244 | 17164 | 363 | 3.00E-99 | 95 | 151 | 411 | 14325 | 14577 | 261 |
| 97 | IMAGE:191877 | H40480 | 285 | AC011244 | 17164 | 391 | e-108 | 93 | 7 | 272 | 2897 | 3159 | 266 |
|  |  | H40479 | 432 | AC011244 | 17164 | 428 | e-119 | 99 | 179 | 410 | 3411 | 3640 | 232 |
| 98 | IMAGE:488945 | AA047078 | 375 | AC025933 | 5438 | 383 | e-105 | 99 | 1 | 201 | 412 | 611 | 201 |
|  |  | AA047077 | 286 | AC025933 | 10382 | 238 | 8.00E-62 | 100 | 105 | 224 | 2810 | 2929 | 120 |
| 99 | IMAGE:1554917 | AA954669 | 482 | AL356136 | 28056 | 892 | 0 | 98 | 1 | 482 | 15484 | 15964 | 482 |
| 100 | IMAGE:1606300 | AA991180 | 318 |  |  |  |  |  |  |  |  |  |  |
| 101 | IMAGE:741841 | AA402879 | 275 | AC092821 | 50684 | 504 | e-142 | 98 | 1 | 275 | 1351 | 1628 | 278 |
| 102 | IMAGE:594438 | AA164543 | 446 | AC026900 | 21471 | 831 | 0 | 98 | 1 | 446 | 13479 | 13925 | 447 |
|  |  | AA165085 | 372 | AC026900 | 21471 | 599 | e-170 | 96 | 6 | 372 | 13407 | 13772 | 367 |
| 103 | IMAGE:815163 | AA481222 | 351 |  |  |  |  |  |  |  |  |  |  |
|  |  | AA481144 | 546 |  |  |  |  |  |  |  |  |  |  |
| 104 | IMAGE:731119 | AA417272 | 480 | AC111200 | 12183 | 952 | 0 | 100 | 1 | 480 | 2343 | 2822 | 480 |
|  |  | AA417282 | 390 | AC111200 | 12183 | 327 | 2.00E-88 | 98 | 214 | 390 | 2766 | 2942 | 177 |
| 105 | IMAGE:234376 | N28268 | 436 | AC068735 | 8016 | 753 | 0 | 96 | 1 | 436 | 1737 | 2171 | 436 |
| 106 | IMAGE:415084 | W94963 | 504 | AC026570 | 7899 | 882 | 0 | 98 | 5 | 491 | 1292 | 1777 | 487 |
|  |  | W93369 | 405 | AC026570 | 7899 | 674 | 0 | 100 | 1 | 340 | 954 | 1293 | 340 |
| 107 | IMAGE:451080 | AA704503 | 386 | AC114498 | 17281 | 741 | 0 | 99 | 1 | 386 | 7094 | 7479 | 386 |
| 108 | IMAGE:344707 | W73304 | 441 | AC004166 | 59279 | 652 | 0 | 95 | 6 | 405 | 24424 | 24824 | 401 |
|  |  | W73039 | 450 | AC004166 | 59279 | 682 | 0 | 96 | 36 | 443 | 24417 | 24824 | 408 |
| 109 | IMAGE:323796 | BX097195 | 610 | AC016767 | 37381 | 1154 | 0 | 98 | 1 | 610 | 33427 | 34036 | 610 |
|  |  | AA284296 | 579 | AC016767 | 37381 | 1084 | 0 | 98 | 5 | 579 | 33425 | 33999 | 575 |
|  |  | W46155 | 378 | AC016767 | 37381 | 291 | 8.00E-78 | 97 | 2 | 162 | 33427 | 33587 | 161 |
|  |  | W46143 | 607 | AC016767 | 37381 | 1025 | 0 | 97 | 24 | 604 | 33470 | 34048 | 581 |
| 110 | IMAGE:1626304 | AI005125 | 441 |  |  |  |  |  |  |  |  |  |  |
| 111 | IMAGE:843429 | AA489520 | 460 |  |  |  |  |  |  |  |  |  |  |
|  |  | AA489519 | 173 |  |  |  |  |  |  |  |  |  |  |
| 112 | IMAGE:32257 | BX103613 | 543 |  |  |  |  |  |  |  |  |  |  |
|  |  | R43360 | 369 |  |  |  |  |  |  |  |  |  |  |
|  |  | R17642 | 175 |  |  |  |  |  |  |  |  |  |  |
| 113 | IMAGE:1031896 | AA609720 | 236 | AC013360 | 14913 | 460 | e-129 | 99 | 1 | 236 | 1879 | 2114 | 236 |
| 114 | IMAGE:626842 | AA191424 | 424 | AC080000 | 23792 | 767 | 0 | 97 | 1 | 424 | 5237 | 5661 | 425 |
| 115 | IMAGE:1855534 | AI306126 | 372 |  |  |  |  |  |  |  |  |  |  |
| 116 | IMAGE:1468630 | BX089889 | 763 | AC017002 | 16746 | 1475 | 0 | 99 | 1 | 748 | 12829 | 13576 | 748 |
|  |  | AA884636 | 437 | AC017002 | 16746 | 856 | 0 | 99 | 3 | 437 | 12827 | 13261 | 435 |
| 117 | IMAGE:753138 | AA400663 | 432 | AC024155 | 8835 | 835 | 0 | 99 | 1 | 432 | 1937 | 2369 | 433 |
|  |  | AA400715 | 402 | AC024155 | 8835 | 775 | 0 | 99 | 1 | 402 | 1937 | 2339 | 403 |
| 118 | IMAGE:126490 | BX115350 | 734 | AP002795 | 5880 | 1041 | 0 | 99 | 202 | 734 | 1692 | 2224 | 533 |
|  |  | R06675 | 357 | AP002795 | 5880 | 464 | e-130 | 96 | 1 | 305 | 1641 | 1936 | 305 |
|  |  | R06618 | 230 | AC019250 | 52520 | 186 | 2.00E-46 | 94 | 6 | 148 | 28852 | 28993 | 143 |
| 119 | IMAGE:731433 | AA470001 | 322 | AC069171 | 31514 | 638 | 0 | 100 | 1 | 322 | 27029 | 27350 | 322 |
|  |  | AA412217 | 306 | AC069171 | 31514 | 607 | e-173 | 100 | 1 | 306 | 27062 | 27367 | 306 |
| 120 | IMAGE:1687053 | AI094802 | 338 |  |  |  |  |  |  |  |  |  |  |
| 121 | IMAGE:1707475 | BX101365 | 477 | AC062027 | 21103 | 924 | 0 | 99 | 1 | 477 | 1812 | 2289 | 478 |
|  |  | AI096713 | 520 | AC062027 | 21103 | 961 | 0 | 99 | 21 | 520 | 1940 | 2438 | 500 |
| 122 | IMAGE:306513 | W31245 | 438 |  |  |  |  |  |  |  |  |  |  |
|  |  | N91811 | 351 |  |  |  |  |  |  |  |  |  |  |
| 123 | IMAGE:815167 | AA481223 | 486 |  |  |  |  |  |  |  |  |  |  |
|  |  | AA481146 | 577 |  |  |  |  |  |  |  |  |  |  |
| 124 | IMAGE:1572723 | AA969822 | 374 | AL354822 | 14795 | 513 | e-144 | 98 | 1 | 271 | 11946 | 12216 | 271 |
| 125 | IMAGE:840763 | AA486135 | 196 | AC004166 | 104755 | 389 | e-107 | 100 | 1 | 196 | 22304 | 22499 | 196 |
|  |  | AA486077 | 177 | AC004166 | 104755 | 351 | 5.00E-96 | 100 | 1 | 177 | 22322 | 22498 | 177 |
| 126 | IMAGE:897813 | AA598533 | 338 |  |  |  |  |  |  |  |  |  |  |
| 127 | IMAGE:2029211 | AI793246 | 357 |  |  |  |  |  |  |  |  |  |  |
|  |  | AI793071 | 376 |  |  |  |  |  |  |  |  |  |  |
|  |  | AI253140 | 359 |  |  |  |  |  |  |  |  |  |  |
| 128 | IMAGE:126229 | R06313 | 273 | AL590643 | 40411 | 424 | e-118 | 96 | 1 | 264 | 6964 | 7224 | 264 |
|  |  | R06258 | 374 | AL590643 | 40411 | 359 | 4.00E-98 | 98 | 93 | 300 | 8566 | 8770 | 208 |
| 129 | IMAGE:287618 | N79274 | 211 |  |  |  |  |  |  |  |  |  |  |
|  |  | N62144 | 458 |  |  |  |  |  |  |  |  |  |  |
| 130 | IMAGE:1212231 | AI821940 | 465 | AC011244 | 4554 | 866 | 0 | 98 | 9 | 465 | 3746 | 4202 | 457 |
|  |  | AI791206 | 533 | AC093243 | 3326 | 950 | 0 | 98 | 31 | 529 | 1393 | 1891 | 499 |
|  |  | AA643410 | 313 | AC011244 | 4554 | 551 | e-156 | 97 | 8 | 313 | 3897 | 4202 | 306 |
| 131 | IMAGE:506551 | BX095732 | 630 | AC064810 | 6882 | 1203 | 0 | 99 | 1 | 615 | 4594 | 5208 | 615 |
|  |  | AA709048 | 391 | AC064810 | 6882 | 731 | 0 | 98 | 1 | 391 | 4594 | 4984 | 391 |
| 132 | IMAGE:1534603 | AA923514 | 353 | AC069171 | 31514 | 670 | 0 | 99 | 1 | 353 | 28436 | 28789 | 354 |
| 133 | IMAGE:204740 | H57306 | 199 | AC011244 | 17164 | 365 | e-100 | 99 | 13 | 199 | 2905 | 3091 | 187 |
|  |  | H57305 | 487 | AC011244 | 17164 | 680 | 0 | 98 | 13 | 374 | 4424 | 4784 | 362 |
| 134 | IMAGE:240748 | H91337 | 281 | AC044842 | 15765 | 551 | e-156 | 99 | 1 | 281 | 13460 | 13740 | 281 |
|  |  | H91044 | 391 | AC044842 | 15765 | 640 | 0 | 98 | 1 | 357 | 13187 | 13539 | 357 |
