# Supplementary material for: Lineage-Specific Gene Duplication and Loss in Human and Great Ape Evolution
Source: PLoS Biol. 2004 Jul 13;2(7):e207. doi: 10.1371/journal.pbio.0020207 (PMC449870; doi:10.1371/journal.pbio.0020207)
Supplement: Table S3 — For each of the 23 LS gene clusters, Satellite repeat subclass analysis was performed. The table lists the cluster's cytogenetic region, the chromosome and start and stop positions, and the adjusted length after accounting for gaps in the genomic sequence. The percent content for 24 subclasses of Satellite repeats is listed for each of the 23 gene clusters. Summary information includes the average content of the 24 subclasses of Satellite repeats for all of the clusters as well as the average for the entire human genome. The difference and fold change are calculated based on comparing the cluster averages to the entire human genome averages. (111 KB DOC). [file pbio.0020207.st003.doc]

| Table S3. Satellite repeat sub-class analysis for LS gene clusters | | | | | | | | | | | | | | | | | | | | | | | | | | |
| --- | --- | --- | --- | --- | --- | --- | --- | --- | --- | --- | --- | --- | --- | --- | --- | --- | --- | --- | --- | --- | --- | --- | --- | --- | --- | --- |
|  | | | **Satellite Repeat Content of Cluster (%)** | | | | | | | | | | | | | | | | | | | | | | | |
| **Cluster** | **Cytogenetic Position** | **Nucleotide Position** | **ACRO1** | **ALR/Alpha** | **BSR/Beta** | **CATTCn** | **CER** | **D20S16** | **GAATGn** | **GSAT** | **GSATII** | **GSATX** | **HSAT4** | **HSAT5** | **HSAT6** | **HSATI** | **HSATII** | **LSAU** | **MSR1** | **REP522** | **SAR** | **SATR1** | **SATR2** | **SST1** | **SUBTEL_sa** | **TAR1** |
| A | 1p36.33 | 10205-370863 | 0.00 | 0.00 | 0.00 | 0.00 | 0.00 | 0.00 | 0.68 | 0.00 | 0.00 | 0.00 | 0.00 | 0.00 | 0.00 | 0.00 | 0.00 | 0.00 | 0.00 | 0.00 | 0.00 | 0.00 | 0.00 | 0.00 | 0.00 | 0.26 |
| B | 1p36.13 | 16040148-16248006 | 0.00 | 0.00 | 0.00 | 0.00 | 0.00 | 0.00 | 0.00 | 0.00 | 0.00 | 0.00 | 0.00 | 0.00 | 0.00 | 0.00 | 0.00 | 0.00 | 0.00 | 0.00 | 0.00 | 0.00 | 0.00 | 0.00 | 0.00 | 0.00 |
| C | 1p13.2-1q21.2 | 119385828-145366889 | 0.00 | 0.00 | 0.00 | 0.00 | 0.67 | 0.00 | 0.07 | 0.00 | 0.00 | 0.00 | 0.00 | 0.04 | 0.00 | 0.00 | 0.00 | 0.00 | 0.00 | 0.00 | 0.00 | 0.02 | 0.00 | 0.00 | 0.00 | 0.01 |
| D | 2p11.2 | 87371301-88563579 | 0.00 | 0.00 | 0.00 | 0.00 | 0.00 | 0.00 | 0.00 | 0.00 | 0.00 | 0.00 | 0.00 | 0.00 | 0.00 | 0.00 | 0.00 | 0.00 | 0.00 | 0.10 | 0.00 | 0.00 | 0.00 | 0.00 | 0.00 | 0.07 |
| E | 2p11.1-2q11.2 | 89358358-93970939 | 0.00 | 0.00 | 0.00 | 0.00 | 0.00 | 0.00 | 0.00 | 0.00 | 0.00 | 0.00 | 0.00 | 0.06 | 0.00 | 0.00 | 0.00 | 0.00 | 0.00 | 0.15 | 0.00 | 0.35 | 0.00 | 0.00 | 0.00 | 0.14 |
| F | 2q14.1 | 112101086-112411341 | 0.00 | 0.00 | 0.00 | 0.00 | 0.00 | 0.00 | 0.00 | 0.00 | 0.00 | 0.00 | 0.00 | 0.00 | 0.00 | 0.00 | 0.00 | 0.00 | 0.00 | 0.00 | 0.00 | 1.12 | 0.00 | 0.00 | 0.00 | 0.67 |
| G | 2q21.2-2q21.3 | 130634597-131402172 | 0.26 | 4.37 | 0.00 | 0.00 | 1.32 | 0.00 | 0.00 | 0.00 | 0.47 | 0.00 | 0.00 | 0.00 | 0.00 | 0.00 | 0.00 | 0.00 | 0.00 | 0.00 | 0.00 | 0.00 | 0.00 | 0.00 | 0.00 | 0.39 |
| H | 5p13.3-5p14.3 | 20943443-22425809 | 0.00 | 0.00 | 0.00 | 0.00 | 0.00 | 0.00 | 0.00 | 0.00 | 0.00 | 0.00 | 0.00 | 0.00 | 0.00 | 0.00 | 0.00 | 0.00 | 0.00 | 0.00 | 0.00 | 0.00 | 0.00 | 0.00 | 0.00 | 0.00 |
| I | 5q13.3 | 70353511-70903396 | 0.00 | 0.00 | 0.00 | 0.00 | 0.00 | 0.00 | 0.00 | 0.00 | 0.00 | 0.00 | 0.00 | 0.00 | 0.00 | 0.00 | 0.00 | 0.00 | 0.00 | 0.00 | 0.00 | 0.00 | 0.00 | 0.00 | 0.00 | 0.00 |
| J | 6p22.1 | 26692149-26992489 | 0.00 | 0.00 | 0.00 | 0.00 | 0.00 | 0.00 | 0.00 | 0.00 | 0.00 | 0.00 | 0.00 | 0.00 | 0.00 | 0.00 | 0.00 | 0.00 | 0.00 | 0.00 | 0.00 | 0.00 | 0.00 | 0.00 | 0.00 | 0.00 |
| K | 7q34 | 141632015-142216972 | 0.00 | 0.00 | 0.00 | 0.00 | 0.00 | 0.00 | 0.00 | 0.00 | 0.00 | 0.00 | 0.00 | 0.00 | 0.00 | 0.00 | 0.00 | 0.00 | 0.00 | 0.00 | 0.00 | 0.00 | 0.00 | 0.00 | 0.00 | 0.00 |
| L | 9p24.3 | 17070-17490 | 0.00 | 0.00 | 0.00 | 0.00 | 0.00 | 0.00 | 0.00 | 0.00 | 0.00 | 0.00 | 0.00 | 0.00 | 0.00 | 0.00 | 0.00 | 0.00 | 0.00 | 0.00 | 0.00 | 2.64 | 0.00 | 0.00 | 0.00 | 0.00 |
| M | 9p13.3-9q21.12 | 38562165-62840292 | 0.00 | 3.81 | 0.00 | 0.00 | 0.29 | 0.00 | 0.00 | 0.00 | 0.26 | 0.00 | 0.00 | 0.04 | 0.00 | 0.00 | 0.00 | 0.00 | 0.00 | 0.15 | 0.00 | 0.28 | 0.00 | 0.45 | 0.00 | 0.09 |
| N | 14p11.1 | 13063292-13805918 | 0.00 | 10.05 | 0.00 | 0.00 | 0.00 | 0.00 | 0.00 | 0.00 | 1.49 | 0.00 | 0.00 | 0.00 | 0.00 | 0.00 | 0.00 | 0.00 | 0.00 | 0.00 | 0.00 | 0.00 | 0.00 | 0.00 | 0.00 | 0.08 |
| O | 15p11.1-15p11.2 | 13039694-15384734 | 0.00 | 0.53 | 0.00 | 0.00 | 0.00 | 0.00 | 0.00 | 0.00 | 0.00 | 0.00 | 0.00 | 0.00 | 0.00 | 0.00 | 0.00 | 0.00 | 0.00 | 0.49 | 0.00 | 0.00 | 0.00 | 0.00 | 0.01 | 0.09 |
| P | 16p11.1-16p11.2 | 32314412-35474685 | 0.00 | 12.99 | 0.03 | 0.14 | 0.00 | 0.00 | 0.03 | 0.00 | 0.00 | 0.00 | 0.00 | 0.00 | 0.04 | 0.02 | 1.40 | 0.01 | 0.00 | 0.22 | 0.00 | 0.00 | 0.00 | 0.00 | 0.00 | 0.06 |
| Q | 18p11.1-18q11.21 | 14311227-18260062 | 0.00 | 1.26 | 0.00 | 0.00 | 0.00 | 0.00 | 0.00 | 0.00 | 0.00 | 0.00 | 0.00 | 0.09 | 0.00 | 0.00 | 0.00 | 0.00 | 0.00 | 0.79 | 0.00 | 0.00 | 0.00 | 0.00 | 0.00 | 0.12 |
| R | 19p13.3 | 16401-198604 | 0.00 | 0.00 | 0.00 | 0.00 | 0.00 | 0.00 | 0.00 | 0.00 | 0.00 | 0.00 | 0.00 | 0.00 | 0.00 | 0.00 | 0.00 | 0.00 | 0.00 | 0.00 | 0.00 | 0.00 | 0.00 | 0.00 | 0.00 | 1.01 |
| S | 20p11.1-20q11.21 | 25698233-29620848 | 0.00 | 9.54 | 0.00 | 0.00 | 0.00 | 0.00 | 3.14 | 0.00 | 0.00 | 0.00 | 0.00 | 0.00 | 0.00 | 0.00 | 0.01 | 0.00 | 0.00 | 0.32 | 0.00 | 0.00 | 0.00 | 4.40 | 0.00 | 0.00 |
| T | 21p11.2 | 7669179-11968553 | 0.00 | 2.51 | 0.00 | 0.00 | 0.77 | 0.00 | 0.00 | 0.00 | 0.00 | 0.00 | 0.00 | 0.08 | 0.00 | 0.06 | 0.00 | 0.00 | 0.00 | 0.63 | 0.00 | 0.00 | 0.00 | 0.00 | 0.00 | 0.04 |
| U | 22q11.1 | 13034022-14321656 | 0.00 | 0.09 | 0.00 | 0.00 | 10.53 | 0.00 | 0.00 | 0.00 | 0.24 | 0.00 | 0.00 | 0.00 | 0.00 | 0.00 | 1.26 | 0.00 | 0.00 | 0.31 | 0.00 | 0.00 | 0.00 | 0.06 | 0.00 | 0.09 |
| V | 22q13.33 | 47696896-47744592 | 0.00 | 0.00 | 0.00 | 0.00 | 0.00 | 0.00 | 0.00 | 0.00 | 0.00 | 0.00 | 0.00 | 0.00 | 0.00 | 0.00 | 0.00 | 0.00 | 0.00 | 0.00 | 0.00 | 0.00 | 0.00 | 0.00 | 0.00 | 0.00 |
| W | Yq11.223 | 20925957-27898184 | 0.00 | 0.00 | 6.38 | 0.00 | 0.00 | 0.00 | 0.00 | 0.00 | 0.00 | 0.00 | 0.00 | 0.00 | 0.00 | 0.00 | 0.00 | 0.00 | 0.00 | 0.02 | 0.00 | 0.00 | 0.00 | 0.41 | 0.00 | 0.01 |
|  |  |  |  |  |  |  |  |  |  |  |  |  |  |  |  |  |  |  |  |  |  |  |  |  |  |  |
| **Average Satellite Content for Clusters w/o Gaps** | | | 0.01 | 1.96 | 0.28 | 0.01 | 0.59 | 0.00 | 0.17 | 0.00 | 0.11 | 0.00 | 0.00 | 0.01 | 0.00 | 0.00 | 0.12 | 0.00 | 0.00 | 0.14 | 0.00 | 0.19 | 0.00 | 0.23 | 0.00 | 0.14 |
| **Average Satellite Content for Human Genome w/o Gaps** | | | 0.00 | 0.22 | 0.05 | 0.01 | 0.02 | 0.00 | 0.01 | 0.01 | 0.00 | 0.00 | 0.01 | 0.00 | 0.00 | 0.00 | 0.01 | 0.00 | 0.00 | 0.01 | 0.00 | 0.03 | 0.01 | 0.01 | 0.00 | 0.00 |
| **Difference** | | | 0.01 | 1.74 | 0.23 | 0.00 | 0.57 | 0.00 | 0.16 | -0.01 | 0.10 | 0.00 | -0.01 | 0.01 | 0.00 | 0.00 | 0.11 | 0.00 | 0.00 | 0.13 | 0.00 | 0.16 | -0.01 | 0.22 | 0.00 | 0.13 |
| **Fold Change** | | | 7.25 | 8.95 | 6.02 | 0.70 | 29.47 | 0.00 | 16.40 | 0.00 | 43.20 | 0.00 | 0.00 | 13.19 | 13.53 | 7.19 | 21.30 | 2.37 | 0.00 | 24.71 | 0.00 | 6.41 | 0.00 | 17.53 | 7.14 | 57.69 |
