# Supplementary material for: Lineage-Specific Gene Duplication and Loss in Human and Great Ape Evolution
Source: PLoS Biol. 2004 Jul 13;2(7):e207. doi: 10.1371/journal.pbio.0020207 (PMC449870; doi:10.1371/journal.pbio.0020207)
Supplement: Table S5 — (52 KB DOC). [file pbio.0020207.st005.doc]

|  |  | Category | HLS | Relative % | LS | Relative % | Genome | Relative % | Average % |
| --- | --- | --- | --- | --- | --- | --- | --- | --- | --- |
|  |  | Total Number of Unique Accessions | 134 | NA | 1,002 | NA | 33,655 | NA | NA |
|  |  | Total Number of Genes Examined | 52 | NA | 484 | NA | 33,655 | NA | NA |
|  |  | Total Number of Genes Classified | 27 | 51.90% | 264 | 54.50% | 6,458 | 19.20% | 41.87% |
|  |  | Total Number of Classifications Made | 49 | NA | 444 | NA | 10,274 | NA | 3589.00 |
| # | GOID | Total Number of Unclassified Genes | 25 | 48.10% | 220 | 45.50% | 27,197 | 80.80% | 58.13% |
| 1 | GO:0003674 | Ligand Binding | 20 | 38.50% | 165 | 34.10% | 3,689 | 10.96% | 27.85% |
| 2 | GO:0003824 | Catalytic Activity | 11 | 21.20% | 121 | 25.00% | 2,212 | 6.57% | 17.59% |
| 3 | GO:0004871 | Signal Transducer Activity | 5 | 9.62% | 29 | 5.99% | 1,287 | 3.82% | 6.48% |
| 4 | GO:0005215 | Transporter Activity | 3 | 5.77% | 40 | 8.26% | 956 | 2.84% | 5.62% |
| 5 | GO:0030528 | Transcription Regulator Activity | 1 | 1.92% | 22 | 4.55% | 655 | 1.95% | 2.80% |
| 6 | GO:0005198 | Structural Molecule Activity | 1 | 1.92% | 10 | 2.07% | 340 | 1.01% | 1.67% |
| 7 | GO:0005554 | Molecular Function Unknown | 4 | 7.69% | 20 | 4.13% | 318 | 0.94% | 4.26% |
| 8 | GO:0030234 | Enzyme Regulator Activity | 0 | 0.00% | 11 | 2.27% | 260 | 0.77% | 1.02% |
| 9 | GO:0005194 | Cell Adhesion Molecule Activity | 0 | 0.00% | 1 | 0.21% | 156 | 0.46% | 0.22% |
| 10 | GO:0003793 | Defense/Immunity Protein Activity | 1 | 1.92% | 4 | 0.83% | 104 | 0.31% | 1.02% |
| 11 | GO:0003754 | Chaperone Activity | 0 | 0.00% | 8 | 1.65% | 95 | 0.28% | 0.65% |
| 12 | GO:0003774 | Motor Activity | 1 | 1.92% | 1 | 0.21% | 73 | 0.22% | 0.78% |
| 13 | GO:0016329 | Apoptosis Regulator Activity | 1 | 1.92% | 3 | 0.62% | 59 | 0.18% | 0.91% |
| 14 | [GO:0045182](javascript:NewWindow('go.cgi?query=GO:0045182&view=details&search_constraint=terms&depth=0&session_id=941081373107', 'Details', '550', '650', 'custom', 'front');) | Translation Regulator Activity | 1 | 1.92% | 8 | 1.65% | 42 | 0.12% | 1.23% |
| 15 | GO:0016209 | Antioxidant Activity | 0 | 0.00% | 0 | 0.00% | 7 | 0.02% | 0.01% |
| 16 | GO:0015070 | Toxin Activity | 0 | 0.00% | 1 | 0.21% | 6 | 0.02% | 0.07% |
| 17 | GO:0008435 | Anticoagulant Activity | 0 | 0.00% | 0 | 0.00% | 3 | 0.01% | 0.00% |
| 18 | GO:0045735 | Nutrient Reservoir Activity | 0 | 0.00% | 0 | 0.00% | 3 | 0.01% | 0.00% |
| 19 | GO:0008638 | Protein Tagging Activity | 0 | 0.00% | 0 | 0.00% | 3 | 0.01% | 0.00% |
| 20 | GO:0019214 | Surfactant Activity | 0 | 0.00% | 0 | 0.00% | 3 | 0.01% | 0.00% |
| 21 | GO:0008580 | Cytoskeletal Regulator Activity | 0 | 0.00% | 0 | 0.00% | 2 | 0.01% | 0.00% |
| 22 | GO:0030188 | Chaperone Regulator Activity | 0 | 0.00% | 0 | 0.00% | 1 | 0.00% | 0.00% |

Table S5- GO Molecular Function Analysis Comparing HLS and LS Genes to the Whole Genome
