# Supplementary material for: Lineage-Specific Gene Duplication and Loss in Human and Great Ape Evolution
Source: PLoS Biol. 2004 Jul 13;2(7):e207. doi: 10.1371/journal.pbio.0020207 (PMC449870; doi:10.1371/journal.pbio.0020207)
Supplement: Table S6 — Presented are pertinent data from GO analysis with DAVID, including numbers of classified and unclassified genes in each gene list, as well as the data returned for each of the 22 most represented molecular function categories. Listed are GO identification numbers (GOIDs) and names for each of the top 22 categories, as well as raw values and relative percent values for HLS, LS, and genome classifications. Relative percent columns are taken as the ratio of the number of classifications in each category to the number of genes classified in the list. The average percent is also provided as the average of these relative percent values across the three groups. This is intended as a metric to help gauge deviations in group relative percent values from the combined average value. (81 KB DOC). [file pbio.0020207.st006.doc]

| Table S6: Pseudogene Analysis and Functional Assessment of Several HLS Gene Copies | | | | | | | | | | | |
| --- | --- | --- | --- | --- | --- | --- | --- | --- | --- | --- | --- |
| **GENE DATA** | | | | | **HUMAN GENOME DATA** | | | | **BLAT DATA** | |  |
| **Name** | **Acc #** | **Len** | **Start** | **Stop** | **Chr** | **Start** | **Stop** | **Strand** | **Score** | **Percent ID** | **Classification** |
| BIRC1 | NM_004536 | 6133 | 1 | 6133 | 5 | 70464552 | 70520765 | + | 6115 | 100.00% | BIRC1 |
|  |  | 6133 | 1 | 6133 | 5 | 34863901 | 35155265 | + | 5438 | 100.00% | GENE-LIKE |
|  |  | 6133 | 3741 | 6133 | 5 | 34796969 | 34805549 | + | 2390 | 100.00% | AMBIGUOUS |
| SLC6A13 | NM_016615 | 2215 | 1 | 2185 | 12 | 184052 | 226263 | - | 2167 | 100.00% | SLC6A13 |
|  |  | 2215 | 156 | 1114 | 12 | 160803 | 173299 | - | 461 | 90.20% | SLC6A12 |
| CHRFAM7A | NM_139320 | 2858 | 1 | 2858 | 15 | 23461833 | 23494254 | - | 2849 | 100.00% | CHRFAM7A |
|  |  | 2858 | 1 | 2858 | 15 | 25165337 | 25262470 | + | 2834 | 99.90% | AMBIGUOUS |
|  |  | 2858 | 1 | 646 | 15 | 23259348 | 23272699 | + | 639 | 99.70% | PSEUDOGENE-LIKE |
|  |  | 2858 | 1 | 646 | 15 | 23233522 | 23246861 | - | 637 | 99.60% | PSEUDOGENE-LIKE |
| SRGAP2 | AB007925 | 6305 | 494 | 6305 | 1 | 202267585 | 202347999 | + | 5779 | 100.00% | SRGAP2 |
|  |  | 6305 | 138 | 1428 | 1 | 202022665 | 202125629 | - | 1275 | 99.70% | AMBIGUOUS |
|  |  | 6305 | 773 | 1428 | 1 | 206570162 | 206590693 | + | 647 | 99.60% | AMBIGUOUS |
| OR2A4 | NM_030908 | 933 | 1 | 933 | 6 | 131868727 | 131869659 | - | 933 | 100.00% | OR2A4 |
|  |  | 933 | 1 | 933 | 7 | 142202238 | 142203170 | - | 925 | 99.60% | OR2A7 |
|  |  | 933 | 27 | 446 | 7 | 142261693 | 142262112 | + | 338 | 90.30% | AMBIGUOUS |
|  |  | 933 | 27 | 446 | 7 | 142175940 | 142176359 | - | 336 | 90.00% | AMBIGUOUS |
|  |  | 933 | 27 | 446 | 7 | 142242941 | 142243360 | + | 335 | 90.20% | AMBIGUOUS |
| ARHGEF5 | NM_005435 | 2227 | 12 | 2227 | 7 | 142309895 | 142324173 | + | 2199 | 100.00% | ARHGEF5 |
|  |  | 2227 | 12 | 1390 | 7 | 142215987 | 142226825 | - | 1369 | 100.00% | GENE-LIKE |
| ROCK1 | NM_005406 | 4065 | 1 | 4065 | 18 | 18263710 | 18421067 | - | 4032 | 100.00% | ROCK1 |
|  |  | 4065 | 3513 | 4065 | 18 | 104444 | 106110 | + | 541 | 99.10% | GENE-LIKE |
| USP10 | NM_005153 | 3009 | 1 | 3009 | 16 | 85700278 | 85779930 | + | 2983 | 99.90% | USP10 |
|  |  | 3009 | 27 | 3009 | 14 | 13420693 | 13423555 | + | 2655 | 96.00% | AMBIGUOUS |
|  |  | 3009 | 27 | 3009 | 14 | 13302649 | 13305512 | + | 2651 | 95.80% | AMBIGUOUS |
|  |  | 3009 | 27 | 3009 | 22 | 13358152 | 13361005 | + | 2647 | 95.90% | AMBIGUOUS |
|  |  | 3009 | 27 | 3009 | 14 | 13933375 | 13936232 | + | 2645 | 95.80% | AMBIGUOUS |
| AQP7 | NM_001170 | 1258 | 1 | 1256 | 9 | 33554391 | 33571960 | - | 1249 | 100.00% | AQP7 |
|  |  | 1258 | 1 | 1252 | 9 | 57903139 | 57920500 | - | 1149 | 96.40% | GENE-LIKE |
|  |  | 1258 | 1 | 1252 | 9 | 52975170 | 52992514 | + | 1149 | 96.40% | MIP1 |
|  |  | 1258 | 1 | 1252 | 9 | 48522868 | 48540230 | + | 1149 | 96.40% | GENE-LIKE |
|  |  | 1258 | 1 | 1252 | 9 | 59109435 | 59126781 | - | 1142 | 96.10% | AMBIGUOUS |
|  |  | 1258 | 1 | 1252 | 9 | 53192834 | 53210197 | + | 1138 | 96.00% | AMBIGUOUS |
|  |  | 1258 | 1 | 1252 | 9 | 62429367 | 62446723 | + | 1131 | 95.90% | MIP5 |
| FLJ13263 | NM_025125 | 2100 | 1 | 2036 | 10 | 81060215 | 81074096 | + | 2029 | 100.00% | FLJ13263 |
|  |  | 2100 | 282 | 2036 | 2 | 128648086 | 128649872 | - | 1482 | 92.60% | PSEUDOGENE-LIKE |
|  |  | 2100 | 282 | 2036 | 14 | 13907568 | 13909368 | - | 1453 | 92.40% | PSEUDOGENE-LIKE |
|  |  | 2100 | 282 | 2036 | 14 | 13276802 | 13278572 | - | 1448 | 92.10% | PSEUDOGENE-LIKE |
|  |  | 2100 | 282 | 2036 | 22 | 13332354 | 13334154 | - | 1434 | 92.40% | PSEUDOGENE-LIKE |
| PLEKHA3 | NM_019091 | 2350 | 2 | 2313 | 2 | 178016498 | 178040927 | + | 2282 | 99.70% | PLEKHA3 |
|  |  | 2350 | 2 | 1941 | 19 | 42418320 | 42420312 | - | 1823 | 97.60% | GENE-LIKE |
