# Supplementary material for: Lineage-Specific Gene Duplication and Loss in Human and Great Ape Evolution
Source: PLoS Biol. 2004 Jul 13;2(7):e207. doi: 10.1371/journal.pbio.0020207 (PMC449870; doi:10.1371/journal.pbio.0020207)
Supplement: Table S7 — The table has three sections: a summary showing the percentages of blocks in each respective chimpanzee homology scoring class; a table with the HLS versus chimpanzee data; and a table with the random versus chimpanzee data. The HLS versus chimpanzee and random versus chimpanzee tables have columns derived from both parsing the BLAT PSL data and from the chimpanzee homology comparison. The table lists the IMAGE clone and the EST accession number used as a query, the hit number, the score and percent identities, the start and stop positions in the query, the chromosome and chromosome start and stop positions, the number of blocks of alignment for the hit, the numbers of blocks that fall into each chimpanzee homology scoring class, and finally the respective chimpanzee scaffold(s) for each hit, if available. (3.58 MB DOC). [file pbio.0020207.st007.doc]

| **Summary** | | | | | |
| --- | --- | --- | --- | --- | --- |
| **Query** | **Chimp Pos** | **Chimp Partial** | **Chimp Gap** | **Chimp Negative** | **Chimp Gap + Chimp Neg** |
| **HLS** | 26.03% | 8.27% | 21.08% | 44.62% | 65.70% |
| **Random** | 81.51% | 8.72% | 6.18% | 3.59% | 9.77% |
| **Fold Difference** | | | | | 6.73 |

| **HLS** | | | | | | | | | | | | | | | |
| --- | --- | --- | --- | --- | --- | --- | --- | --- | --- | --- | --- | --- | --- | --- | --- |
| **clone** | **query** | **num** | **score** | **id** | **q_start** | **q_stop** | **chr** | **chr_start** | **chr_stop** | **block_count** | **num_chimp_pos** | **num_chimp_partial** | **num_chimp_gap** | **num_chimp_neg** | **chimp_scaffold(s)** |
| IMAGE:1566212 | AI066560 | 1 | 479 | 99.4 | 4 | 489 | chr6 | 132003641 | 132004126 | 1 | 0 | 0 | 1 | 0 |  |
|  |  | 2 | 485 | 100 | 4 | 489 | chr7 | 143348996 | 143349481 | 1 | 0 | 0 | 1 | 0 |  |
| IMAGE:1626299 | AI005134 | 1 | 430 | 100 | 3 | 432 | chr7 | 143386953 | 143387382 | 1 | 0 | 0 | 1 | 0 |  |
|  |  | 2 | 430 | 100 | 3 | 432 | chr7 | 143341262 | 143341691 | 1 | 0 | 0 | 1 | 0 |  |
| IMAGE:50904 | H19233 | 1 | 428 | 93.3 | 1 | 490 | chr21 | 46569417 | 46569891 | 12 | 0 | 0 | 12 | 0 |  |
|  |  | 2 | 429 | 95.3 | 1 | 490 | chr21 | 46568973 | 46569595 | 12 | 0 | 0 | 12 | 0 |  |
|  |  | 3 | 424 | 92.9 | 1 | 490 | chr21 | 46569713 | 46570187 | 12 | 0 | 0 | 12 | 0 |  |
|  |  | 4 | 426 | 95.1 | 1 | 490 | chr21 | 46568751 | 46569743 | 12 | 1 | 1 | 10 | 0 | scaffold_33342 |
|  |  | 5 | 408 | 93.9 | 1 | 456 | chr21 | 46570009 | 46570454 | 9 | 0 | 0 | 9 | 0 |  |
|  |  | 6 | 415 | 94.4 | 1 | 490 | chr21 | 46568677 | 46569225 | 12 | 1 | 1 | 10 | 0 | scaffold_33342 |
|  |  | 7 | 394 | 93.7 | 1 | 438 | chr21 | 46569343 | 46569772 | 8 | 0 | 0 | 8 | 0 |  |
|  |  | 8 | 394 | 93.1 | 1 | 449 | chr21 | 46570379 | 46570817 | 9 | 7 | 1 | 1 | 0 | scaffold_33342 |
|  |  | 9 | 411 | 94.7 | 1 | 490 | chr21 | 46568603 | 46569447 | 12 | 2 | 1 | 9 | 0 | scaffold_33342 |
|  |  | 10 | 325 | 96.8 | 1 | 349 | chr21 | 46569121 | 46569468 | 2 | 0 | 0 | 2 | 0 |  |
|  |  | 11 | 280 | 97.3 | 1 | 292 | chr21 | 46570527 | 46570817 | 2 | 0 | 1 | 1 | 0 | scaffold_33342 |
| IMAGE:50904 | H19234 | 1 | 342 | 92.5 | 23 | 442 | chr21 | 46570500 | 46570914 | 10 | 2 | 1 | 7 | 0 | scaffold_33342 |
|  |  | 2 | 261 | 93.9 | 117 | 442 | chr21 | 46569242 | 46570225 | 9 | 0 | 0 | 9 | 0 |  |
|  |  | 3 | 258 | 93.2 | 117 | 442 | chr21 | 46569094 | 46570151 | 9 | 0 | 0 | 9 | 0 |  |
|  |  | 4 | 255 | 93.5 | 117 | 442 | chr21 | 46568724 | 46569559 | 9 | 5 | 0 | 4 | 0 | scaffold_33342 |
| IMAGE:843276 | AA486041 | 1 | 418 | 97.7 | 1 | 443 | chr1 | 143069214 | 143071582 | 5 | 0 | 0 | 0 | 5 |  |
|  |  | 2 | 418 | 97.7 | 1 | 443 | chr1 | 143083387 | 143085739 | 5 | 0 | 0 | 0 | 5 |  |
|  |  | 3 | 418 | 97.7 | 1 | 443 | chr1 | 143102275 | 143104647 | 5 | 0 | 0 | 0 | 5 |  |
|  |  | 4 | 418 | 97.7 | 1 | 443 | chr1 | 143088107 | 143090459 | 5 | 0 | 0 | 0 | 5 |  |
|  |  | 5 | 418 | 97.7 | 1 | 443 | chr1 | 143078669 | 143081033 | 5 | 0 | 0 | 0 | 5 |  |
|  |  | 6 | 418 | 97.7 | 1 | 443 | chr1 | 143064494 | 143066864 | 5 | 3 | 1 | 0 | 1 | scaffold_1219 |
|  |  | 7 | 418 | 97.7 | 1 | 443 | chr1 | 143092831 | 143095183 | 5 | 0 | 0 | 0 | 5 |  |
|  |  | 8 | 418 | 97.7 | 1 | 443 | chr1 | 143097555 | 143099913 | 5 | 0 | 0 | 0 | 5 |  |
|  |  | 9 | 418 | 97.7 | 1 | 443 | chr1 | 143107007 | 143115637 | 5 | 0 | 0 | 0 | 5 |  |
|  |  | 10 | 414 | 97.3 | 1 | 443 | chr1 | 143073938 | 143076303 | 5 | 0 | 0 | 0 | 5 |  |
|  |  | 11 | 404 | 95.9 | 1 | 443 | chr1 | 143962717 | 143965097 | 5 | 1 | 0 | 0 | 4 | scaffold_5788 |
|  |  | 12 | 402 | 95.7 | 1 | 443 | chr1 | 143957957 | 143960337 | 5 | 0 | 0 | 0 | 5 |  |
|  |  | 13 | 402 | 95.7 | 1 | 443 | chr1 | 143967477 | 143969857 | 5 | 0 | 0 | 0 | 5 |  |
|  |  | 14 | 405 | 97 | 1 | 443 | chr1 | 145956221 | 146115899 | 6 | 2 | 0 | 4 | 0 | scaffold_965 |
|  |  | 15 | 418 | 97.7 | 1 | 443 | chr1 | 142580658 | 143062144 | 5 | 2 | 1 | 2 | 0 | scaffold_23067 scaffold_1219 |
|  |  | 16 | 330 | 97.2 | 90 | 443 | chr1 | 142576631 | 142578298 | 4 | 0 | 0 | 0 | 4 |  |
|  |  | 17 | 422 | 98.2 | 1 | 443 | chr1 | 145705696 | 145709619 | 5 | 0 | 0 | 5 | 0 |  |
|  |  | 18 | 330 | 97.2 | 90 | 443 | chr1 | 145711977 | 145713648 | 4 | 0 | 0 | 4 | 0 |  |
|  |  | 19 | 326 | 96.3 | 90 | 443 | chr1 | 143766691 | 143768358 | 4 | 0 | 0 | 0 | 4 |  |
|  |  | 20 | 296 | 94.3 | 90 | 433 | chr1 | 145704132 | 145707367 | 4 | 0 | 0 | 4 | 0 |  |
|  |  | 21 | 280 | 97 | 90 | 397 | chr1 | 16273304 | 16274931 | 4 | 2 | 0 | 0 | 2 | scaffold_14405 |
|  |  | 22 | 221 | 91.7 | 90 | 395 | chr1 | 146452570 | 146457238 | 5 | 5 | 0 | 0 | 0 | scaffold_36950 |
|  |  | 23 | 422 | 98.2 | 1 | 443 | chr1_random | 5468979 | 5471381 | 5 | 0 | 0 | 0 | 5 |  |
|  |  | 24 | 422 | 98.2 | 1 | 443 | chr1_random | 5464200 | 5466606 | 5 | 0 | 0 | 0 | 5 |  |
|  |  | 25 | 420 | 98 | 1 | 443 | chr1_random | 5453333 | 5455726 | 5 | 0 | 0 | 0 | 5 |  |
|  |  | 26 | 420 | 98 | 1 | 443 | chr1_random | 5459444 | 5461840 | 5 | 0 | 0 | 0 | 5 |  |
|  |  | 27 | 370 | 97.2 | 1 | 443 | chr1_random | 5458092 | 5474568 | 4 | 0 | 0 | 0 | 4 |  |
|  |  | 28 | 334 | 97.7 | 90 | 443 | chr1_random | 5449289 | 5450960 | 4 | 0 | 0 | 0 | 4 |  |
|  |  | 29 | 310 | 95.9 | 1 | 397 | chr1_random | 5473749 | 5479340 | 4 | 0 | 0 | 0 | 4 |  |
|  |  | 30 | 251 | 97.4 | 1 | 266 | chr1_random | 1020576 | 1141774 | 2 | 0 | 0 | 0 | 2 |  |
|  |  | 31 | 224 | 94.1 | 90 | 443 | chr1_random | 1018099 | 1021394 | 3 | 0 | 0 | 0 | 3 |  |
|  |  | 32 | 422 | 98.2 | 1 | 443 | chr1_random | 5423209 | 5425599 | 5 | 0 | 0 | 0 | 5 |  |
|  |  | 33 | 421 | 98.2 | 1 | 443 | chr1_random | 5430428 | 5432829 | 5 | 0 | 0 | 0 | 5 |  |
|  |  | 34 | 408 | 96.6 | 1 | 443 | chr1_random | 5415246 | 5420841 | 5 | 0 | 0 | 0 | 5 |  |
|  |  | 35 | 307 | 98.4 | 1 | 319 | chr1_random | 5405996 | 5407670 | 3 | 0 | 0 | 0 | 3 |  |
|  |  | 36 | 215 | 97.4 | 1 | 443 | chr1_random | 5410041 | 5410860 | 4 | 0 | 0 | 0 | 4 |  |
|  |  | 37 | 209 | 93 | 90 | 443 | chr1_random | 5418443 | 5421748 | 3 | 0 | 0 | 0 | 3 |  |
| IMAGE:843276 | AA488658 | 1 | 479 | 99.2 | 1 | 491 | chr1 | 145703313 | 145706465 | 6 | 0 | 0 | 6 | 0 |  |
|  |  | 2 | 433 | 99.1 | 1 | 491 | chr1 | 145709590 | 145712746 | 4 | 0 | 0 | 4 | 0 |  |
|  |  | 3 | 427 | 98.2 | 1 | 449 | chr1 | 143764294 | 143766818 | 6 | 0 | 1 | 0 | 5 | scaffold_34605 |
|  |  | 4 | 368 | 96.5 | 1 | 449 | chr1 | 16272449 | 16274931 | 5 | 1 | 1 | 0 | 3 | scaffold_14405 |
|  |  | 5 | 327 | 91.2 | 50 | 449 | chr1 | 146454000 | 146457238 | 4 | 4 | 0 | 0 | 0 | scaffold_36950 |
|  |  | 6 | 322 | 90.5 | 50 | 449 | chr1 | 21222205 | 21223928 | 4 | 1 | 1 | 0 | 2 | scaffold_37569 |
|  |  | 7 | 481 | 99.4 | 1 | 491 | chr1 | 143099148 | 143102304 | 6 | 0 | 0 | 0 | 6 |  |
|  |  | 8 | 481 | 99.4 | 1 | 491 | chr1 | 143094418 | 143107036 | 6 | 0 | 0 | 0 | 6 |  |
|  |  | 9 | 477 | 99 | 1 | 491 | chr1 | 146115134 | 146118294 | 6 | 1 | 0 | 5 | 0 | scaffold_965 |
|  |  | 10 | 475 | 98.8 | 1 | 491 | chr1 | 142577533 | 142580687 | 6 | 1 | 0 | 0 | 5 | scaffold_23067 |
|  |  | 11 | 473 | 98.6 | 1 | 491 | chr1 | 143954808 | 143957986 | 6 | 0 | 0 | 0 | 6 |  |
|  |  | 12 | 473 | 98.6 | 1 | 491 | chr1 | 143964333 | 143967506 | 6 | 0 | 0 | 0 | 6 |  |
|  |  | 13 | 472 | 98.6 | 1 | 491 | chr1 | 143969093 | 143972310 | 7 | 0 | 0 | 0 | 7 |  |
|  |  | 14 | 471 | 99 | 6 | 491 | chr1 | 145953086 | 145956289 | 7 | 0 | 0 | 7 | 0 |  |
|  |  | 15 | 437 | 99.5 | 1 | 491 | chr1 | 143061371 | 143064523 | 4 | 1 | 2 | 1 | 0 | scaffold_1219 |
|  |  | 16 | 437 | 99.5 | 1 | 491 | chr1 | 143066099 | 143073967 | 4 | 1 | 1 | 0 | 2 | scaffold_1219 |
|  |  | 17 | 435 | 99.3 | 1 | 491 | chr1 | 143084974 | 143088136 | 4 | 0 | 0 | 0 | 4 |  |
|  |  | 18 | 435 | 99.3 | 1 | 491 | chr1 | 143080268 | 143083416 | 4 | 0 | 0 | 0 | 4 |  |
|  |  | 19 | 435 | 99.3 | 1 | 491 | chr1 | 143089694 | 143097584 | 4 | 0 | 0 | 0 | 4 |  |
|  |  | 20 | 435 | 99.3 | 1 | 491 | chr1 | 143070817 | 143078698 | 4 | 0 | 0 | 0 | 4 |  |
|  |  | 21 | 436 | 99.5 | 1 | 491 | chr1 | 143103880 | 143113342 | 5 | 0 | 0 | 0 | 5 |  |
|  |  | 22 | 427 | 98.4 | 1 | 491 | chr1 | 143959573 | 143962746 | 4 | 0 | 2 | 0 | 2 | scaffold_5788 |
|  |  | 23 | 478 | 99.2 | 1 | 491 | chr1_random | 5420812 | 5423980 | 7 | 0 | 0 | 0 | 7 |  |
|  |  | 24 | 477 | 99.2 | 1 | 491 | chr1_random | 5410831 | 5419220 | 6 | 0 | 0 | 0 | 6 |  |
|  |  | 25 | 475 | 99.2 | 1 | 491 | chr1_random | 5425570 | 5431208 | 6 | 0 | 0 | 0 | 6 |  |
|  |  | 26 | 475 | 99 | 1 | 491 | chr1_random | 5407641 | 5428731 | 6 | 0 | 0 | 0 | 6 |  |
|  |  | 27 | 450 | 98.9 | 1 | 491 | chr1_random | 5432800 | 5435965 | 6 | 0 | 0 | 0 | 6 |  |
|  |  | 28 | 392 | 99 | 1 | 449 | chr1_random | 5416040 | 5418570 | 3 | 0 | 0 | 0 | 3 |  |
|  |  | 29 | 325 | 96.3 | 47 | 449 | chr1_random | 5411591 | 5432110 | 4 | 0 | 0 | 0 | 4 |  |
|  |  | 30 | 209 | 96.9 | 106 | 449 | chr1_random | 5406827 | 5410168 | 2 | 0 | 0 | 0 | 2 |  |
|  |  | 31 | 480 | 99.4 | 1 | 491 | chr1_random | 1017461 | 1023837 | 7 | 1 | 1 | 0 | 5 | scaffold_36312 |
|  |  | 32 | 480 | 99.4 | 1 | 491 | chr1_random | 5461063 | 5464229 | 7 | 0 | 0 | 0 | 7 |  |
|  |  | 33 | 477 | 99.2 | 1 | 491 | chr1_random | 5450191 | 5453362 | 6 | 0 | 0 | 0 | 6 |  |
|  |  | 34 | 477 | 99.2 | 1 | 491 | chr1_random | 5465827 | 5469008 | 6 | 0 | 0 | 0 | 6 |  |
|  |  | 35 | 477 | 99.2 | 1 | 491 | chr1_random | 5454952 | 5459473 | 6 | 0 | 0 | 0 | 6 |  |
|  |  | 36 | 477 | 99.2 | 1 | 491 | chr1_random | 5470600 | 5476979 | 6 | 0 | 0 | 0 | 6 |  |
|  |  | 37 | 434 | 98.7 | 1 | 449 | chr1_random | 1018099 | 1020605 | 5 | 0 | 0 | 0 | 5 |  |
|  |  | 38 | 434 | 98.9 | 1 | 449 | chr1_random | 5471254 | 5473778 | 5 | 0 | 0 | 0 | 5 |  |
|  |  | 39 | 326 | 96.8 | 47 | 449 | chr1_random | 5477667 | 5479435 | 4 | 0 | 0 | 0 | 4 |  |
|  |  | 40 | 229 | 92.3 | 1 | 449 | chr1_random | 5449289 | 5459517 | 3 | 0 | 0 | 0 | 3 |  |
|  |  | 41 | 213 | 92.1 | 106 | 449 | chr1_random | 5458811 | 5477784 | 3 | 0 | 0 | 0 | 3 |  |
| IMAGE:1877990 | AI275888 | 1 | 362 | 95 | 1 | 402 | chr11 | 134345724 | 134346125 | 1 | 0 | 0 | 1 | 0 |  |
|  |  | 2 | 398 | 99.5 | 1 | 402 | chr18 | 14967431 | 14967832 | 1 | 0 | 0 | 1 | 0 |  |
| IMAGE:279874 | BX093390 | 1 | 579 | 95.4 | 1 | 648 | chr1 | 146370821 | 146371475 | 8 | 0 | 0 | 8 | 0 |  |
|  |  | 2 | 589 | 95.8 | 1 | 648 | chr1 | 146039447 | 146040101 | 8 | 6 | 1 | 1 | 0 | scaffold_11571 |
|  |  | 3 | 538 | 95.3 | 45 | 648 | chr1 | 146201130 | 146201738 | 7 | 0 | 0 | 7 | 0 |  |
|  |  | 4 | 587 | 95.6 | 1 | 648 | chr15 | 18374142 | 18374797 | 8 | 0 | 0 | 0 | 8 |  |
|  |  | 5 | 593 | 96.1 | 1 | 648 | chr16 | 34717212 | 34717867 | 7 | 0 | 0 | 0 | 7 |  |
|  |  | 6 | 584 | 95.5 | 1 | 648 | chr16 | 32178115 | 32178766 | 9 | 0 | 0 | 0 | 9 |  |
|  |  | 7 | 581 | 96.2 | 1 | 648 | chr16 | 34204049 | 34204700 | 9 | 0 | 0 | 0 | 9 |  |
|  |  | 8 | 577 | 95.1 | 1 | 648 | chr16 | 33121201 | 33121853 | 9 | 0 | 0 | 0 | 9 |  |
|  |  | 9 | 595 | 96.3 | 1 | 648 | chr16 | 34143739 | 34144394 | 7 | 0 | 0 | 0 | 7 |  |
|  |  | 10 | 583 | 96 | 1 | 648 | chr16 | 34655060 | 34655711 | 8 | 5 | 1 | 2 | 0 | scaffold_36587 |
|  |  | 11 | 574 | 95 | 1 | 648 | chr16 | 33870322 | 33870974 | 9 | 0 | 0 | 0 | 9 |  |
|  |  | 12 | 567 | 95.1 | 1 | 648 | chr16 | 32843956 | 32844608 | 9 | 0 | 0 | 0 | 9 |  |
|  |  | 13 | 571 | 95.1 | 1 | 648 | chr17 | 25448295 | 25448948 | 8 | 6 | 2 | 0 | 0 | scaffold_18715 |
|  |  | 14 | 576 | 95.6 | 1 | 636 | chr18 | 15167129 | 15167758 | 7 | 6 | 1 | 0 | 0 | scaffold_16266 |
|  |  | 15 | 561 | 94.6 | 1 | 636 | chr2 | 91104846 | 91105487 | 7 | 6 | 1 | 0 | 0 | scaffold_21851 |
|  |  | 16 | 642 | 99.2 | 1 | 648 | chr2 | 132992441 | 132993086 | 3 | 0 | 0 | 0 | 3 |  |
|  |  | 17 | 566 | 94.5 | 1 | 648 | chr7 | 61218045 | 61218694 | 10 | 0 | 0 | 0 | 10 |  |
|  |  | 18 | 570 | 94.7 | 1 | 648 | chr7 | 61146486 | 61147135 | 10 | 0 | 0 | 0 | 10 |  |
|  |  | 19 | 560 | 94.4 | 1 | 636 | chr7 | 52971024 | 52971665 | 7 | 4 | 2 | 0 | 1 | scaffold_33738 |
|  |  | 20 | 554 | 93.7 | 1 | 636 | chr7 | 64370955 | 64371587 | 10 | 0 | 0 | 0 | 10 |  |
|  |  | 21 | 507 | 93.6 | 1 | 576 | chr7 | 57698983 | 57699552 | 7 | 0 | 0 | 7 | 0 |  |
|  |  | 22 | 577 | 95 | 1 | 648 | chr9_random | 460730 | 461382 | 8 | 3 | 2 | 0 | 3 | scaffold_36387 scaffold_10967 |
| IMAGE:279874 | N40992 | 1 | 388 | 95.4 | 1 | 439 | chr1 | 146039051 | 146039506 | 8 | 8 | 0 | 0 | 0 | scaffold_11571 |
|  |  | 2 | 384 | 94.9 | 1 | 439 | chr1 | 146200734 | 146201189 | 8 | 0 | 0 | 8 | 0 |  |
|  |  | 3 | 384 | 94.9 | 1 | 439 | chr1 | 146371416 | 146371871 | 8 | 0 | 0 | 8 | 0 |  |
|  |  | 4 | 380 | 96 | 1 | 439 | chr15 | 18374737 | 18375189 | 9 | 5 | 2 | 0 | 2 | scaffold_26102 scaffold_2829 |
|  |  | 5 | 389 | 95.4 | 1 | 439 | chr16 | 32843563 | 32844015 | 7 | 0 | 0 | 0 | 7 |  |
|  |  | 6 | 385 | 94.9 | 1 | 439 | chr16 | 33869929 | 33870381 | 7 | 0 | 0 | 0 | 7 |  |
|  |  | 7 | 382 | 95.1 | 1 | 439 | chr16 | 34143352 | 34143798 | 8 | 0 | 0 | 0 | 8 |  |
|  |  | 8 | 381 | 95.5 | 1 | 439 | chr16 | 34654672 | 34655119 | 11 | 10 | 1 | 0 | 0 | scaffold_36587 |
|  |  | 9 | 389 | 95.4 | 1 | 439 | chr16 | 33121794 | 33122246 | 7 | 0 | 0 | 0 | 7 |  |
|  |  | 10 | 386 | 95.2 | 1 | 439 | chr16 | 32178707 | 32179162 | 8 | 0 | 0 | 0 | 8 |  |
|  |  | 11 | 382 | 95.1 | 1 | 439 | chr16 | 34717808 | 34718254 | 8 | 0 | 0 | 0 | 8 |  |
|  |  | 12 | 382 | 95.5 | 1 | 439 | chr16 | 34204641 | 34205089 | 11 | 0 | 0 | 0 | 11 |  |
|  |  | 13 | 400 | 97 | 1 | 439 | chr17 | 25448889 | 25449345 | 10 | 9 | 1 | 0 | 0 | scaffold_18715 |
|  |  | 14 | 379 | 94.7 | 2 | 439 | chr18 | 15166724 | 15167177 | 10 | 7 | 3 | 0 | 0 | scaffold_16266 |
|  |  | 15 | 432 | 99.8 | 1 | 439 | chr2 | 132992058 | 132992496 | 1 | 0 | 0 | 0 | 1 |  |
|  |  | 16 | 371 | 95 | 1 | 426 | chr2 | 91105456 | 91105893 | 9 | 7 | 2 | 0 | 0 | scaffold_21851 |
|  |  | 17 | 386 | 94.9 | 1 | 439 | chr7 | 57698521 | 57698968 | 6 | 0 | 0 | 6 | 0 |  |
|  |  | 18 | 385 | 96 | 10 | 439 | chr7 | 64370563 | 64371003 | 12 | 0 | 0 | 0 | 12 |  |
|  |  | 19 | 372 | 94 | 1 | 439 | chr7 | 61146089 | 61146546 | 10 | 0 | 0 | 0 | 10 |  |
|  |  | 20 | 373 | 95 | 1 | 439 | chr7 | 52970621 | 52971072 | 7 | 4 | 3 | 0 | 0 | scaffold_17821 scaffold_33738 |
|  |  | 21 | 371 | 93.7 | 1 | 439 | chr7 | 61218634 | 61219091 | 10 | 0 | 0 | 0 | 10 |  |
|  |  | 22 | 383 | 94.9 | 1 | 439 | chr9_random | 461323 | 461779 | 10 | 0 | 0 | 0 | 10 |  |
| IMAGE:279874 | N45002 | 1 | 325 | 94.4 | 1 | 366 | chr1 | 146370821 | 146371184 | 6 | 0 | 0 | 6 | 0 |  |
|  |  | 2 | 327 | 94.7 | 1 | 366 | chr1 | 146039738 | 146040101 | 6 | 4 | 1 | 1 | 0 | scaffold_11571 |
|  |  | 3 | 323 | 94.1 | 1 | 366 | chr1 | 146201421 | 146201784 | 6 | 0 | 0 | 6 | 0 |  |
|  |  | 4 | 327 | 94.7 | 1 | 366 | chr15 | 18374142 | 18374505 | 6 | 0 | 0 | 0 | 6 |  |
|  |  | 5 | 329 | 94.7 | 1 | 366 | chr16 | 32178115 | 32178477 | 6 | 0 | 0 | 0 | 6 |  |
|  |  | 6 | 329 | 95 | 1 | 366 | chr16 | 33121201 | 33121564 | 6 | 0 | 0 | 0 | 6 |  |
|  |  | 7 | 327 | 94.7 | 1 | 366 | chr16 | 34717212 | 34717575 | 6 | 0 | 0 | 0 | 6 |  |
|  |  | 8 | 315 | 93.8 | 1 | 366 | chr16 | 34204049 | 34204408 | 8 | 0 | 0 | 0 | 8 |  |
|  |  | 9 | 329 | 95 | 1 | 366 | chr16 | 34144031 | 34144394 | 6 | 0 | 0 | 0 | 6 |  |
|  |  | 10 | 329 | 95 | 1 | 366 | chr16 | 32844245 | 32844608 | 6 | 0 | 0 | 0 | 6 |  |
|  |  | 11 | 327 | 94.7 | 1 | 366 | chr16 | 33870611 | 33870974 | 6 | 0 | 0 | 0 | 6 |  |
|  |  | 12 | 321 | 94.2 | 1 | 366 | chr16 | 34655352 | 34655711 | 7 | 4 | 1 | 2 | 0 | scaffold_36587 |
|  |  | 13 | 317 | 95.6 | 1 | 347 | chr17 | 25448295 | 25448639 | 5 | 4 | 1 | 0 | 0 | scaffold_18715 |
|  |  | 14 | 321 | 93.9 | 1 | 366 | chr18 | 15167410 | 15167758 | 7 | 6 | 1 | 0 | 0 | scaffold_16266 |
|  |  | 15 | 315 | 92.2 | 1 | 366 | chr2 | 91104846 | 91105206 | 7 | 6 | 1 | 0 | 0 | scaffold_21851 |
|  |  | 16 | 361 | 97.3 | 1 | 371 | chr2 | 132992720 | 132993086 | 5 | 0 | 0 | 0 | 5 |  |
|  |  | 17 | 328 | 93.5 | 1 | 366 | chr7 | 61218045 | 61218401 | 8 | 0 | 0 | 0 | 8 |  |
|  |  | 18 | 330 | 93.8 | 1 | 366 | chr7 | 61146779 | 61147135 | 8 | 0 | 0 | 0 | 8 |  |
|  |  | 19 | 324 | 93.7 | 1 | 366 | chr7 | 57699198 | 57699552 | 7 | 0 | 0 | 7 | 0 |  |
|  |  | 20 | 321 | 93.8 | 1 | 366 | chr7 | 52971305 | 52971665 | 7 | 5 | 1 | 0 | 1 | scaffold_33738 |
|  |  | 21 | 298 | 91.6 | 1 | 348 | chr7 | 64371252 | 64371587 | 7 | 0 | 0 | 0 | 7 |  |
|  |  | 22 | 327 | 94.4 | 1 | 366 | chr9_random | 460730 | 461090 | 7 | 6 | 1 | 0 | 0 | scaffold_36387 scaffold_10967 |
| IMAGE:1856246 | AI240359 | 1 | 445 | 98.7 | 6 | 463 | chr1 | 141746355 | 141746819 | 2 | 0 | 1 | 0 | 1 | scaffold_26820 |
|  |  | 2 | 442 | 98.5 | 6 | 463 | chr1 | 146219134 | 146219599 | 3 | 0 | 0 | 3 | 0 |  |
|  |  | 3 | 433 | 97 | 2 | 463 | chr10 | 41955296 | 41955764 | 2 | 0 | 0 | 0 | 2 |  |
|  |  | 4 | 425 | 96.3 | 2 | 463 | chr10 | 38896172 | 38896639 | 4 | 0 | 0 | 0 | 4 |  |
|  |  | 5 | 437 | 97.4 | 2 | 463 | chr15 | 18403755 | 18404222 | 3 | 0 | 1 | 2 | 0 | scaffold_37081 |
|  |  | 6 | 419 | 95.4 | 2 | 463 | chr15 | 18359195 | 18359662 | 3 | 1 | 2 | 0 | 0 | scaffold_2962 |
|  |  | 7 | 445 | 98.3 | 2 | 463 | chr16 | 33887077 | 33887545 | 2 | 0 | 1 | 0 | 1 | scaffold_24448 |
|  |  | 8 | 441 | 97.8 | 2 | 463 | chr16 | 32527128 | 32527596 | 2 | 0 | 2 | 0 | 0 | scaffold_29068 |
|  |  | 9 | 439 | 97.6 | 2 | 463 | chr16 | 33523168 | 33523636 | 2 | 0 | 0 | 0 | 2 |  |
|  |  | 10 | 439 | 97.6 | 2 | 463 | chr16 | 32860626 | 32861094 | 2 | 1 | 1 | 0 | 0 | scaffold_6431 |
|  |  | 11 | 433 | 97.2 | 2 | 463 | chr16 | 33105288 | 33105753 | 3 | 0 | 0 | 0 | 3 |  |
|  |  | 12 | 431 | 96.9 | 2 | 463 | chr16 | 32161992 | 32162456 | 4 | 0 | 0 | 0 | 4 |  |
|  |  | 13 | 419 | 95.5 | 2 | 463 | chr16 | 34982449 | 34982917 | 2 | 0 | 0 | 0 | 2 |  |
|  |  | 14 | 419 | 95.5 | 2 | 463 | chr17 | 25433832 | 25434300 | 2 | 0 | 0 | 0 | 2 |  |
|  |  | 15 | 421 | 95.9 | 2 | 463 | chr18 | 15187047 | 15187511 | 4 | 2 | 2 | 0 | 0 | scaffold_27760 |
|  |  | 16 | 447 | 98.5 | 2 | 463 | chr2 | 91717293 | 91717761 | 2 | 0 | 0 | 0 | 2 |  |
|  |  | 17 | 396 | 93 | 2 | 463 | chr2 | 133007909 | 133008357 | 3 | 0 | 0 | 0 | 3 |  |
|  |  | 18 | 446 | 98.3 | 2 | 463 | chr2 | 91152979 | 91153440 | 1 | 0 | 0 | 0 | 1 |  |
|  |  | 19 | 447 | 98.5 | 2 | 463 | chr2 | 91480575 | 91481043 | 2 | 1 | 1 | 0 | 0 | scaffold_6964 |
|  |  | 20 | 427 | 96.3 | 2 | 463 | chr22 | 15240707 | 15241175 | 2 | 0 | 0 | 0 | 2 |  |
|  |  | 21 | 417 | 95.2 | 2 | 463 | chr7 | 64450307 | 64450775 | 2 | 0 | 0 | 0 | 2 |  |
|  |  | 22 | 413 | 94.8 | 2 | 463 | chr7 | 64387423 | 64387891 | 2 | 0 | 0 | 0 | 2 |  |
|  |  | 23 | 399 | 94.1 | 2 | 463 | chr7 | 57708092 | 57708556 | 3 | 0 | 0 | 0 | 3 |  |
|  |  | 24 | 391 | 93.4 | 2 | 459 | chr7 | 52931805 | 52932273 | 3 | 1 | 2 | 0 | 0 | scaffold_37409 |
|  |  | 25 | 417 | 95.2 | 2 | 463 | chr7 | 63993900 | 63994368 | 2 | 0 | 0 | 0 | 2 |  |
|  |  | 26 | 437 | 97.4 | 2 | 463 | chr9 | 65918915 | 65919383 | 2 | 0 | 0 | 0 | 2 |  |
|  |  | 27 | 428 | 96.7 | 2 | 463 | chr9 | 66140475 | 66140939 | 3 | 0 | 0 | 3 | 0 |  |
| IMAGE:1856246 | BX118334 | 1 | 465 | 99.6 | 1 | 469 | chr1 | 146219614 | 146220082 | 1 | 0 | 0 | 1 | 0 |  |
|  |  | 2 | 457 | 98.7 | 1 | 469 | chr1 | 141746834 | 141747302 | 1 | 0 | 1 | 0 | 0 | scaffold_26820 |
|  |  | 3 | 430 | 95.9 | 1 | 469 | chr10 | 41954812 | 41955281 | 3 | 0 | 0 | 0 | 3 |  |
|  |  | 4 | 404 | 92.7 | 1 | 469 | chr10 | 38895705 | 38896157 | 2 | 0 | 0 | 0 | 2 |  |
|  |  | 5 | 417 | 94.5 | 1 | 469 | chr15 | 18403272 | 18403740 | 1 | 0 | 1 | 0 | 0 | scaffold_37081 |
|  |  | 6 | 392 | 93 | 1 | 461 | chr15 | 18358712 | 18359172 | 4 | 2 | 2 | 0 | 0 | scaffold_2962 |
|  |  | 7 | 453 | 98.3 | 1 | 469 | chr16 | 32161509 | 32161977 | 1 | 0 | 0 | 0 | 1 |  |
|  |  | 8 | 451 | 98.1 | 1 | 469 | chr16 | 33104805 | 33105273 | 1 | 0 | 1 | 0 | 0 | scaffold_1512 |
|  |  | 9 | 401 | 93.7 | 1 | 461 | chr16 | 34981963 | 34982426 | 3 | 0 | 0 | 0 | 3 |  |
|  |  | 10 | 449 | 97.9 | 1 | 469 | chr16 | 33887560 | 33888028 | 1 | 0 | 1 | 0 | 0 | scaffold_24448 |
|  |  | 11 | 449 | 97.9 | 1 | 469 | chr16 | 32861109 | 32861577 | 1 | 0 | 0 | 0 | 1 |  |
|  |  | 12 | 419 | 94.2 | 1 | 469 | chr16 | 33523651 | 33524118 | 2 | 0 | 0 | 0 | 2 |  |
|  |  | 13 | 417 | 94 | 1 | 469 | chr16 | 32527611 | 32528078 | 2 | 0 | 0 | 0 | 2 |  |
|  |  | 14 | 400 | 92.7 | 1 | 469 | chr17 | 25433348 | 25433817 | 3 | 0 | 0 | 0 | 3 |  |
|  |  | 15 | 401 | 94.3 | 1 | 469 | chr18 | 15187526 | 15187998 | 3 | 0 | 0 | 3 | 0 |  |
|  |  | 16 | 429 | 95.9 | 1 | 469 | chr2 | 91152494 | 91153093 | 3 | 0 | 0 | 0 | 3 |  |
|  |  | 17 | 420 | 94.9 | 1 | 469 | chr2 | 91480090 | 91480560 | 2 | 0 | 0 | 0 | 2 |  |
|  |  | 18 | 420 | 94.9 | 1 | 469 | chr2 | 91717776 | 91718246 | 2 | 0 | 0 | 0 | 2 |  |
|  |  | 19 | 403 | 93.9 | 1 | 460 | chr2 | 133008381 | 133008841 | 3 | 0 | 0 | 0 | 3 |  |
|  |  | 20 | 424 | 94.6 | 1 | 469 | chr22 | 15240227 | 15240693 | 2 | 1 | 1 | 0 | 0 | scaffold_8122 |
|  |  | 21 | 409 | 94.2 | 2 | 465 | chr7 | 63993417 | 63993881 | 3 | 0 | 0 | 0 | 3 |  |
|  |  | 22 | 409 | 94.2 | 2 | 465 | chr7 | 64450794 | 64451258 | 3 | 0 | 0 | 0 | 3 |  |
|  |  | 23 | 406 | 94 | 1 | 469 | chr7 | 52932294 | 52932762 | 3 | 3 | 0 | 0 | 0 | scaffold_37409 |
|  |  | 24 | 400 | 93.1 | 1 | 465 | chr7 | 64387910 | 64388376 | 2 | 0 | 0 | 0 | 2 |  |
|  |  | 25 | 391 | 92.8 | 1 | 458 | chr7 | 57708575 | 57709033 | 3 | 0 | 0 | 0 | 3 |  |
|  |  | 26 | 420 | 94.9 | 1 | 469 | chr9 | 66140954 | 66141424 | 2 | 0 | 0 | 2 | 0 |  |
|  |  | 27 | 416 | 94.4 | 1 | 469 | chr9 | 65919398 | 65919867 | 3 | 0 | 0 | 0 | 3 |  |
| IMAGE:1699118 | AI003508 | 1 | 473 | 98.2 | 2 | 490 | chr1 | 140860187 | 140860674 | 2 | 1 | 1 | 0 | 0 | scaffold_29734 |
|  |  | 2 | 438 | 95.2 | 2 | 490 | chr2 | 95069855 | 95070381 | 4 | 4 | 0 | 0 | 0 | scaffold_12453 |
|  |  | 3 | 475 | 98.4 | 2 | 490 | chr4 | 49455108 | 49455595 | 2 | 0 | 0 | 0 | 2 |  |
|  |  | 4 | 477 | 98.2 | 2 | 490 | chr4 | 49174862 | 49175348 | 3 | 0 | 0 | 0 | 3 |  |
|  |  | 5 | 445 | 94.6 | 2 | 490 | chrUn_random | 928497 | 928980 | 4 | 4 | 0 | 0 | 0 | scaffold_4115 |
| IMAGE:1699118 | AI733556 | 1 | 467 | 98.5 | 2 | 482 | chr1 | 140860187 | 140860667 | 1 | 0 | 1 | 0 | 0 | scaffold_29734 |
|  |  | 2 | 367 | 90.9 | 2 | 476 | chr18 | 14662965 | 14663737 | 7 | 0 | 0 | 7 | 0 |  |
|  |  | 3 | 434 | 95.4 | 2 | 482 | chr2 | 95069862 | 95070381 | 4 | 4 | 0 | 0 | 0 | scaffold_12453 |
|  |  | 4 | 469 | 98.8 | 2 | 482 | chr4 | 49455108 | 49455588 | 1 | 0 | 0 | 0 | 1 |  |
|  |  | 5 | 471 | 98.5 | 2 | 482 | chr4 | 49174869 | 49175348 | 2 | 0 | 0 | 0 | 2 |  |
|  |  | 6 | 439 | 94.8 | 2 | 482 | chrUn_random | 928504 | 928980 | 3 | 3 | 0 | 0 | 0 | scaffold_4115 |
| IMAGE:1699118 | AI792327 | 1 | 474 | 98.4 | 14 | 504 | chr1 | 140860397 | 140860887 | 1 | 0 | 1 | 0 | 0 | scaffold_29734 |
|  |  | 2 | 413 | 94.8 | 16 | 499 | chr2 | 95062737 | 95070169 | 7 | 7 | 0 | 0 | 0 | scaffold_12453 |
|  |  | 3 | 476 | 98.2 | 14 | 504 | chr4 | 49174649 | 49175138 | 2 | 0 | 0 | 0 | 2 |  |
|  |  | 4 | 470 | 98 | 14 | 504 | chr4 | 49455318 | 49455808 | 1 | 0 | 0 | 0 | 1 |  |
|  |  | 5 | 449 | 95.5 | 15 | 504 | chrUn_random | 928285 | 928773 | 2 | 1 | 1 | 0 | 0 | scaffold_24397 scaffold_4115 |
| IMAGE:365515 | AA009608 | 1 | 444 | 97 | 1 | 491 | chr15 | 47432611 | 47492714 | 10 | 10 | 0 | 0 | 0 | scaffold_37412 scaffold_33062 |
| IMAGE:365515 | AA009609 | 1 | 333 | 95.7 | 1 | 375 | chr15 | 47493325 | 47493689 | 7 | 7 | 0 | 0 | 0 | scaffold_33062 |
|  |  | 2 | 329 | 95.1 | 1 | 375 | chr18 | 14875768 | 14876132 | 7 | 4 | 2 | 1 | 0 | scaffold_3521 |
|  |  | 3 | 329 | 95.1 | 1 | 375 | chr21 | 13645410 | 13645774 | 7 | 4 | 2 | 0 | 1 | scaffold_26020 scaffold_10238 |
|  |  | 4 | 329 | 95.1 | 1 | 375 | chr9 | 41447585 | 41447949 | 7 | 6 | 1 | 0 | 0 | scaffold_26934 |
|  |  | 5 | 329 | 95.1 | 1 | 375 | chr9 | 63719009 | 63719373 | 7 | 0 | 0 | 0 | 7 |  |
|  |  | 6 | 327 | 94.8 | 1 | 375 | chr9 | 62635031 | 62635395 | 7 | 0 | 0 | 0 | 7 |  |
| IMAGE:365515 | BX094084 | 1 | 705 | 99.9 | 1 | 709 | chr15 | 47432611 | 47492941 | 3 | 3 | 0 | 0 | 0 | scaffold_37412 scaffold_33062 |
|  |  | 2 | 451 | 97.1 | 227 | 709 | chr18 | 14876518 | 14878085 | 4 | 0 | 0 | 4 | 0 |  |
|  |  | 3 | 459 | 97.7 | 227 | 709 | chr21 | 13643457 | 13645024 | 4 | 0 | 0 | 0 | 4 |  |
|  |  | 4 | 466 | 98.3 | 227 | 709 | chr9 | 63717051 | 63718620 | 2 | 0 | 0 | 0 | 2 |  |
|  |  | 5 | 457 | 97.7 | 227 | 707 | chr9 | 62633064 | 62634640 | 3 | 0 | 0 | 0 | 3 |  |
|  |  | 6 | 466 | 98.3 | 227 | 709 | chr9 | 41448338 | 41449907 | 2 | 0 | 1 | 1 | 0 | scaffold_26934 |
| IMAGE:796303 | AA459724 | 1 | 363 | 99.7 | 1 | 365 | chr1 | 141752578 | 141752942 | 1 | 0 | 0 | 0 | 1 |  |
|  |  | 2 | 362 | 100 | 4 | 365 | chr1 | 203664384 | 203664745 | 1 | 0 | 1 | 0 | 0 | scaffold_37640 |
| IMAGE:796303 | AA461307 | 1 | 431 | 100 | 1 | 431 | chr1 | 203664239 | 203664669 | 1 | 1 | 0 | 0 | 0 | scaffold_37640 |
|  |  | 2 | 431 | 100 | 1 | 431 | chr1 | 141752657 | 141753087 | 1 | 0 | 0 | 0 | 1 |  |
| IMAGE:814792 | AA455233 | 1 | 407 | 95.9 | 1 | 444 | chr14 | 18138785 | 18139229 | 2 | 0 | 2 | 0 | 0 | scaffold_37076 |
|  |  | 2 | 442 | 99.8 | 1 | 444 | chr16 | 84592776 | 84593219 | 1 | 1 | 0 | 0 | 0 | scaffold_37614 |
|  |  | 3 | 411 | 96.4 | 1 | 444 | chr22 | 14790898 | 14791342 | 2 | 0 | 0 | 0 | 2 |  |
| IMAGE:814792 | AA465611 | 1 | 381 | 95.3 | 1 | 416 | chr14 | 18137673 | 18138084 | 4 | 1 | 3 | 0 | 0 | scaffold_37076 |
|  |  | 2 | 407 | 99.8 | 1 | 415 | chr16 | 84577401 | 84585927 | 4 | 4 | 0 | 0 | 0 | scaffold_37614 |
|  |  | 3 | 377 | 95.1 | 1 | 416 | chr22 | 14789796 | 14790207 | 4 | 0 | 0 | 0 | 4 |  |
| IMAGE:1856923 | AI271431 | 1 | 402 | 98.8 | 1 | 412 | chrUn_random | 2161960 | 2162371 | 1 | 0 | 1 | 0 | 0 | scaffold_37640 |
| IMAGE:135010 | BX116199 | 1 | 523 | 99.2 | 2 | 533 | chr1 | 141749862 | 141750393 | 1 | 0 | 0 | 0 | 1 |  |
|  |  | 2 | 385 | 94.7 | 104 | 537 | chr10 | 38893066 | 38893619 | 3 | 0 | 0 | 0 | 3 |  |
|  |  | 3 | 381 | 95.3 | 104 | 537 | chr10 | 41951850 | 41952403 | 4 | 0 | 0 | 0 | 4 |  |
|  |  | 4 | 398 | 96.1 | 104 | 535 | chr15 | 18400281 | 18400712 | 1 | 0 | 1 | 0 | 0 | scaffold_10712 |
|  |  | 5 | 207 | 93.3 | 292 | 531 | chr15 | 18355919 | 18356160 | 2 | 2 | 0 | 0 | 0 | scaffold_2962 |
|  |  | 6 | 396 | 96 | 104 | 533 | chr16 | 33101809 | 33102238 | 1 | 0 | 0 | 0 | 1 |  |
|  |  | 7 | 394 | 95.8 | 104 | 533 | chr16 | 32158518 | 32158947 | 1 | 0 | 0 | 0 | 1 |  |
|  |  | 8 | 383 | 94.7 | 104 | 533 | chr16 | 34978949 | 34979406 | 2 | 1 | 1 | 0 | 0 | scaffold_36816 |
|  |  | 9 | 418 | 98.4 | 104 | 535 | chr16 | 33890591 | 33891022 | 1 | 0 | 1 | 0 | 0 | scaffold_27082 |
|  |  | 10 | 415 | 98.1 | 104 | 534 | chr16 | 32864141 | 32864571 | 1 | 0 | 1 | 0 | 0 | scaffold_17987 |
|  |  | 11 | 371 | 92.9 | 104 | 527 | chr17 | 25430394 | 25430813 | 2 | 0 | 1 | 0 | 1 | scaffold_30554 |
|  |  | 12 | 371 | 93.3 | 104 | 533 | chr18 | 15190541 | 15190968 | 4 | 4 | 0 | 0 | 0 | scaffold_6986 |
|  |  | 13 | 414 | 97.9 | 104 | 536 | chr2 | 91149503 | 91149935 | 3 | 0 | 0 | 0 | 3 |  |
|  |  | 14 | 402 | 96.7 | 104 | 533 | chr2 | 91477106 | 91477535 | 1 | 0 | 0 | 0 | 1 |  |
|  |  | 15 | 403 | 96.8 | 104 | 534 | chr2 | 91720801 | 91721231 | 1 | 0 | 0 | 0 | 1 |  |
|  |  | 16 | 388 | 94.9 | 104 | 537 | chr2 | 133011077 | 133011825 | 3 | 0 | 3 | 0 | 0 | scaffold_10178 |
|  |  | 17 | 390 | 94.8 | 104 | 531 | chr22 | 15237261 | 15237685 | 2 | 0 | 0 | 0 | 2 |  |
|  |  | 18 | 388 | 95.8 | 104 | 527 | chr7 | 63990441 | 63990864 | 1 | 0 | 0 | 0 | 1 |  |
|  |  | 19 | 396 | 95.4 | 104 | 535 | chr7 | 57711568 | 57711998 | 2 | 0 | 0 | 0 | 2 |  |
|  |  | 20 | 389 | 95.3 | 105 | 533 | chr7 | 64390913 | 64391341 | 1 | 0 | 0 | 0 | 1 |  |
|  |  | 21 | 388 | 95.8 | 104 | 527 | chr7 | 64453806 | 64454229 | 1 | 0 | 0 | 0 | 1 |  |
|  |  | 22 | 369 | 93.4 | 104 | 529 | chr7 | 52935337 | 52935763 | 2 | 2 | 0 | 0 | 0 | scaffold_37409 |
|  |  | 23 | 401 | 96.5 | 106 | 537 | chr9 | 66143462 | 66144378 | 2 | 0 | 0 | 2 | 0 |  |
|  |  | 24 | 399 | 96.3 | 106 | 537 | chr9 | 65921909 | 65922825 | 2 | 0 | 0 | 0 | 2 |  |
| IMAGE:135010 | R31645 | 1 | 288 | 94.1 | 2 | 316 | chr1 | 141750088 | 141750393 | 9 | 0 | 0 | 0 | 9 |  |
| IMAGE:135010 | R32361 | 1 | 231 | 93.5 | 1 | 253 | chr1 | 141749893 | 141750139 | 7 | 0 | 0 | 0 | 7 |  |
|  |  | 2 | 210 | 90.1 | 1 | 253 | chr10 | 38893218 | 38893463 | 7 | 0 | 0 | 0 | 7 |  |
|  |  | 3 | 214 | 92.3 | 1 | 253 | chr15 | 18400433 | 18400679 | 7 | 6 | 1 | 0 | 0 | scaffold_10712 |
|  |  | 4 | 228 | 93.1 | 1 | 253 | chr16 | 32864173 | 32864419 | 7 | 7 | 0 | 0 | 0 | scaffold_17987 |
|  |  | 5 | 226 | 92.7 | 1 | 253 | chr16 | 33890624 | 33890870 | 7 | 6 | 1 | 0 | 0 | scaffold_27082 |
|  |  | 6 | 214 | 91.3 | 1 | 253 | chr16 | 32158670 | 32158916 | 7 | 0 | 0 | 0 | 7 |  |
|  |  | 7 | 213 | 90.8 | 1 | 253 | chr16 | 33101961 | 33102207 | 7 | 0 | 0 | 0 | 7 |  |
|  |  | 8 | 214 | 90.2 | 1 | 253 | chr2 | 91720833 | 91721079 | 7 | 0 | 0 | 0 | 7 |  |
|  |  | 9 | 221 | 92.5 | 1 | 253 | chr2 | 91149655 | 91149901 | 8 | 0 | 0 | 0 | 8 |  |
|  |  | 10 | 214 | 90.2 | 1 | 253 | chr2 | 91477258 | 91477504 | 7 | 0 | 0 | 0 | 7 |  |
|  |  | 11 | 211 | 90.1 | 1 | 253 | chr7 | 63990593 | 63990839 | 7 | 0 | 0 | 0 | 7 |  |
|  |  | 12 | 209 | 90.4 | 1 | 253 | chr9 | 66143982 | 66144228 | 7 | 0 | 0 | 7 | 0 |  |
| IMAGE:755093 | AA482508 | 1 | 507 | 99.8 | 4 | 512 | chr5 | 70481299 | 70481807 | 1 | 0 | 0 | 1 | 0 |  |
|  |  | 2 | 507 | 99.8 | 4 | 512 | chr5 | 69658102 | 69658610 | 1 | 0 | 0 | 0 | 1 |  |
|  |  | 3 | 505 | 99.6 | 4 | 512 | chr5 | 69938438 | 69938946 | 1 | 0 | 0 | 0 | 1 |  |
|  |  | 4 | 507 | 99.8 | 4 | 512 | chr5 | 69046786 | 69047294 | 1 | 1 | 0 | 0 | 0 | scaffold_37073 |
| IMAGE:755093 | AA482660 | 1 | 344 | 99.7 | 15 | 360 | chr5 | 69045139 | 69045484 | 1 | 1 | 0 | 0 | 0 | scaffold_37073 |
|  |  | 2 | 344 | 99.7 | 15 | 360 | chr5 | 70483109 | 70483454 | 1 | 0 | 0 | 1 | 0 |  |
|  |  | 3 | 344 | 99.7 | 15 | 360 | chr5 | 69940248 | 69940593 | 1 | 0 | 0 | 0 | 1 |  |
|  |  | 4 | 344 | 99.7 | 15 | 360 | chr5 | 69659912 | 69660257 | 1 | 0 | 0 | 0 | 1 |  |
| IMAGE:755093 | AI820932 | 1 | 416 | 99.5 | 1 | 420 | chr5 | 69045139 | 69045558 | 1 | 1 | 0 | 0 | 0 | scaffold_37073 |
|  |  | 2 | 416 | 99.5 | 1 | 420 | chr5 | 70483035 | 70483454 | 1 | 0 | 0 | 1 | 0 |  |
|  |  | 3 | 416 | 99.5 | 1 | 420 | chr5 | 69940174 | 69940593 | 1 | 0 | 0 | 0 | 1 |  |
|  |  | 4 | 416 | 99.5 | 1 | 420 | chr5 | 69659838 | 69660257 | 1 | 0 | 0 | 0 | 1 |  |
| IMAGE:755093 | AI821682 | 1 | 677 | 98 | 9 | 715 | chr5 | 70481306 | 70482011 | 2 | 0 | 0 | 2 | 0 |  |
|  |  | 2 | 677 | 98 | 9 | 715 | chr5 | 69658109 | 69658814 | 2 | 0 | 0 | 0 | 2 |  |
|  |  | 3 | 675 | 97.9 | 9 | 715 | chr5 | 69938445 | 69939150 | 2 | 0 | 0 | 0 | 2 |  |
|  |  | 4 | 677 | 98 | 9 | 715 | chr5 | 69046582 | 69047287 | 2 | 2 | 0 | 0 | 0 | scaffold_37073 |
| IMAGE:298685 | BX105439 | 1 | 646 | 99.1 | 1 | 664 | chr1 | 17554 | 18227 | 5 | 2 | 3 | 0 | 0 | scaffold_17856 |
|  |  | 2 | 646 | 99.1 | 1 | 664 | chr15 | 100238063 | 100238736 | 5 | 4 | 1 | 0 | 0 | scaffold_13714 |
|  |  | 3 | 646 | 99.1 | 1 | 664 | chr19 | 20299 | 20972 | 5 | 0 | 0 | 0 | 5 |  |
|  |  | 4 | 658 | 99.2 | 1 | 664 | chr2 | 114437812 | 114438472 | 2 | 0 | 0 | 0 | 2 |  |
|  |  | 5 | 644 | 98.9 | 1 | 664 | chr9 | 17804 | 18477 | 5 | 0 | 0 | 0 | 5 |  |
| IMAGE:298685 | N74332 | 1 | 250 | 99.2 | 1 | 256 | chr1 | 17425 | 17685 | 3 | 2 | 1 | 0 | 0 | scaffold_17856 |
|  |  | 2 | 250 | 99.2 | 1 | 256 | chr15 | 100238605 | 100238865 | 3 | 2 | 1 | 0 | 0 | scaffold_13714 |
|  |  | 3 | 250 | 99.2 | 1 | 256 | chr19 | 20170 | 20430 | 3 | 0 | 0 | 0 | 3 |  |
|  |  | 4 | 276 | 97.9 | 1 | 287 | chr2 | 114438318 | 114438601 | 2 | 0 | 0 | 0 | 2 |  |
|  |  | 5 | 250 | 99.2 | 1 | 256 | chr9 | 17675 | 17935 | 3 | 0 | 0 | 0 | 3 |  |
| IMAGE:298685 | W05240 | 1 | 305 | 95.9 | 1 | 326 | chr1 | 17909 | 18227 | 6 | 5 | 1 | 0 | 0 | scaffold_17856 |
|  |  | 2 | 301 | 95.3 | 1 | 326 | chr15 | 100238063 | 100238381 | 6 | 6 | 0 | 0 | 0 | scaffold_13714 |
|  |  | 3 | 305 | 95.9 | 1 | 326 | chr19 | 20654 | 20972 | 6 | 0 | 0 | 0 | 6 |  |
|  |  | 4 | 307 | 96.2 | 1 | 326 | chr2 | 114437812 | 114438130 | 6 | 0 | 0 | 0 | 6 |  |
|  |  | 5 | 305 | 95.9 | 1 | 326 | chr9 | 18159 | 18477 | 6 | 0 | 0 | 0 | 6 |  |
| IMAGE:136933 | R36662 | 1 | 289 | 98.3 | 1 | 304 | chr1 | 10384 | 10710 | 4 | 3 | 0 | 1 | 0 | scaffold_17856 |
|  |  | 2 | 285 | 97.7 | 1 | 304 | chr15 | 100245581 | 100245907 | 4 | 0 | 0 | 0 | 4 |  |
|  |  | 3 | 287 | 98.3 | 1 | 302 | chr19 | 13129 | 13471 | 4 | 0 | 0 | 0 | 4 |  |
|  |  | 4 | 279 | 97.3 | 1 | 302 | chr2 | 114445302 | 114445641 | 5 | 0 | 0 | 0 | 5 |  |
|  |  | 5 | 285 | 97.7 | 1 | 304 | chr9 | 10634 | 10960 | 4 | 0 | 0 | 0 | 4 |  |
| IMAGE:1474402 | AA922384 | 1 | 267 | 98.9 | 1 | 269 | chr6 | 132009879 | 132010146 | 2 | 0 | 0 | 2 | 0 |  |
|  |  | 2 | 269 | 100 | 1 | 269 | chr7 | 143274919 | 143275187 | 1 | 0 | 0 | 1 | 0 |  |
|  |  | 3 | 219 | 94.9 | 1 | 252 | chr7 | 142742415 | 142742665 | 6 | 6 | 0 | 0 | 0 | scaffold_37657 |
|  |  | 4 | 267 | 98.9 | 1 | 269 | chr7 | 143355236 | 143355503 | 2 | 0 | 0 | 2 | 0 |  |
|  |  | 5 | 224 | 94 | 1 | 252 | chr7 | 142843668 | 142843918 | 4 | 0 | 0 | 4 | 0 |  |
| IMAGE:1474402 | AI733069 | 1 | 376 | 98.5 | 68 | 474 | chr6 | 132009438 | 132010101 | 19 | 0 | 0 | 19 | 0 |  |
|  |  | 2 | 378 | 98.8 | 68 | 474 | chr7 | 143274964 | 143275603 | 19 | 0 | 0 | 19 | 0 |  |
|  |  | 3 | 264 | 93.9 | 94 | 432 | chr7 | 142742488 | 142742850 | 14 | 13 | 1 | 0 | 0 | scaffold_37657 |
|  |  | 4 | 376 | 98.5 | 68 | 474 | chr7 | 143354795 | 143355458 | 19 | 0 | 0 | 19 | 0 |  |
|  |  | 5 | 261 | 94.4 | 94 | 432 | chr7 | 142843483 | 142843845 | 15 | 0 | 0 | 15 | 0 |  |
| IMAGE:1474402 | AI791559 | 1 | 399 | 98.5 | 1 | 429 | chr7 | 143275564 | 143275991 | 4 | 0 | 0 | 4 | 0 |  |
|  |  | 2 | 349 | 96.1 | 1 | 429 | chr7 | 142743067 | 143374574 | 5 | 2 | 2 | 1 | 0 | scaffold_37657 |
| IMAGE:1467026 | AA883127 | 1 | 426 | 97.1 | 1 | 452 | chr1 | 146375153 | 146375604 | 1 | 0 | 0 | 1 | 0 |  |
|  |  | 2 | 428 | 96.9 | 1 | 452 | chr1 | 146035091 | 146035541 | 2 | 0 | 0 | 2 | 0 |  |
|  |  | 3 | 426 | 97.1 | 1 | 452 | chr1 | 146197001 | 146197452 | 1 | 0 | 0 | 1 | 0 |  |
|  |  | 4 | 390 | 93.1 | 1 | 452 | chr10 | 41939026 | 41939477 | 1 | 0 | 0 | 0 | 1 |  |
|  |  | 5 | 406 | 94.9 | 1 | 452 | chr15 | 18378446 | 18378897 | 1 | 0 | 1 | 0 | 0 | scaffold_2829 |
|  |  | 6 | 452 | 100 | 1 | 452 | chr16 | 32182644 | 32183095 | 1 | 0 | 0 | 0 | 1 |  |
|  |  | 7 | 446 | 99.3 | 1 | 452 | chr16 | 33125731 | 33126182 | 1 | 0 | 1 | 0 | 0 | scaffold_17126 |
|  |  | 8 | 398 | 94 | 1 | 452 | chr16 | 34721483 | 34721934 | 1 | 0 | 1 | 0 | 0 | scaffold_31090 |
|  |  | 9 | 394 | 93.5 | 7 | 452 | chr16 | 34208368 | 34208810 | 2 | 0 | 0 | 0 | 2 |  |
|  |  | 10 | 448 | 99.6 | 1 | 452 | chr16 | 33866217 | 33866668 | 1 | 0 | 0 | 0 | 1 |  |
|  |  | 11 | 446 | 99.3 | 1 | 452 | chr16 | 32839627 | 32840078 | 1 | 0 | 0 | 0 | 1 |  |
|  |  | 12 | 414 | 95.8 | 1 | 452 | chr16 | 46274818 | 46275269 | 1 | 0 | 0 | 0 | 1 |  |
|  |  | 13 | 406 | 94.9 | 1 | 452 | chr16 | 33086546 | 33086997 | 1 | 0 | 0 | 1 | 0 |  |
|  |  | 14 | 406 | 94.9 | 1 | 452 | chr16 | 32143257 | 32143708 | 1 | 0 | 0 | 0 | 1 |  |
|  |  | 15 | 398 | 94 | 1 | 452 | chr16 | 34139672 | 34140123 | 1 | 0 | 0 | 0 | 1 |  |
|  |  | 16 | 390 | 93.5 | 7 | 452 | chr16 | 34650949 | 34651393 | 2 | 1 | 1 | 0 | 0 | scaffold_36587 |
|  |  | 17 | 398 | 94 | 1 | 452 | chr17 | 25452624 | 25453075 | 1 | 1 | 0 | 0 | 0 | scaffold_19909 |
|  |  | 18 | 406 | 94.5 | 1 | 452 | chr18 | 15162992 | 15163442 | 2 | 2 | 0 | 0 | 0 | scaffold_16266 |
|  |  | 19 | 396 | 93.3 | 1 | 452 | chr2 | 91109166 | 91109616 | 2 | 0 | 0 | 2 | 0 |  |
|  |  | 20 | 379 | 92.5 | 12 | 452 | chr2 | 132988343 | 132988782 | 2 | 0 | 0 | 0 | 2 |  |
|  |  | 21 | 241 | 96.5 | 194 | 452 | chr6 | 83269745 | 83270003 | 1 | 1 | 0 | 0 | 0 | scaffold_37647 |
|  |  | 22 | 403 | 94.7 | 1 | 452 | chr7 | 61222349 | 61222802 | 2 | 0 | 0 | 0 | 2 |  |
|  |  | 23 | 409 | 95.3 | 1 | 451 | chr7 | 60854222 | 60854672 | 1 | 0 | 1 | 0 | 0 | scaffold_15414 |
|  |  | 24 | 403 | 94.7 | 1 | 452 | chr7 | 61142376 | 61142829 | 2 | 0 | 0 | 0 | 2 |  |
|  |  | 25 | 400 | 93.5 | 1 | 452 | chr7 | 57694807 | 57695253 | 2 | 0 | 0 | 2 | 0 |  |
|  |  | 26 | 393 | 93.6 | 1 | 452 | chr7 | 52968886 | 52969338 | 3 | 2 | 1 | 0 | 0 | scaffold_17821 |
|  |  | 27 | 402 | 94.5 | 1 | 452 | chr9_random | 465044 | 465495 | 1 | 0 | 0 | 0 | 1 |  |
| IMAGE:704320 | AA279467 | 1 | 371 | 95.8 | 1 | 406 | chr1 | 224908 | 225314 | 2 | 0 | 0 | 0 | 2 |  |
|  |  | 2 | 365 | 95.1 | 1 | 406 | chr1 | 504118 | 504524 | 2 | 0 | 0 | 0 | 2 |  |
|  |  | 3 | 395 | 98.8 | 1 | 406 | chr10 | 135019609 | 135020015 | 2 | 0 | 2 | 0 | 0 | scaffold_28680 |
|  |  | 4 | 365 | 95.1 | 1 | 406 | chr16 | 90032284 | 90032690 | 2 | 1 | 1 | 0 | 0 | scaffold_21962 |
|  |  | 5 | 361 | 94.6 | 1 | 406 | chr17_random | 587871 | 588277 | 2 | 0 | 0 | 0 | 2 |  |
|  |  | 6 | 357 | 94.3 | 2 | 405 | chr18 | 90640 | 91044 | 2 | 0 | 0 | 0 | 2 |  |
|  |  | 7 | 395 | 98.8 | 1 | 406 | chr19 | 209605 | 210011 | 2 | 0 | 0 | 0 | 2 |  |
|  |  | 8 | 392 | 98 | 1 | 406 | chr19 | 63793704 | 63794108 | 3 | 2 | 1 | 0 | 0 | scaffold_12768 |
|  |  | 9 | 402 | 99.3 | 1 | 406 | chr2 | 114473021 | 114473425 | 3 | 1 | 2 | 0 | 0 | scaffold_16940 scaffold_10473 |
|  |  | 10 | 396 | 98.5 | 1 | 406 | chr21 | 46958421 | 46958825 | 3 | 0 | 0 | 0 | 3 |  |
|  |  | 11 | 395 | 98.3 | 1 | 406 | chr22 | 49361043 | 49361446 | 3 | 0 | 0 | 0 | 3 |  |
|  |  | 12 | 353 | 93.8 | 2 | 405 | chr4 | 13698 | 14102 | 2 | 1 | 1 | 0 | 0 | scaffold_32227 |
|  |  | 13 | 353 | 93.8 | 2 | 405 | chr4 | 191716215 | 191716619 | 2 | 0 | 0 | 0 | 2 |  |
|  |  | 14 | 365 | 95.1 | 1 | 406 | chr5 | 181001974 | 181002380 | 2 | 0 | 0 | 0 | 2 |  |
|  |  | 15 | 371 | 95.8 | 1 | 406 | chr6 | 170904454 | 170904860 | 2 | 0 | 0 | 0 | 2 |  |
|  |  | 16 | 363 | 94.8 | 1 | 406 | chr8 | 2458 | 2864 | 2 | 0 | 1 | 0 | 1 | scaffold_14386 |
| IMAGE:238756 | H64701 | 1 | 341 | 98.3 | 1 | 347 | chr1 | 141931654 | 141931998 | 3 | 2 | 1 | 0 | 0 | scaffold_37640 |
| IMAGE:238756 | H65193 | 1 | 302 | 96.1 | 1 | 329 | chr1 | 141931556 | 141931878 | 8 | 7 | 1 | 0 | 0 | scaffold_37640 |
| IMAGE:384886 | AA708832 | 1 | 467 | 99.2 | 1 | 475 | chr9 | 63658323 | 63658797 | 1 | 0 | 0 | 0 | 1 |  |
|  |  | 2 | 455 | 97.9 | 1 | 475 | chr9 | 40844036 | 40844510 | 1 | 0 | 0 | 0 | 1 |  |
|  |  | 3 | 471 | 99.6 | 1 | 475 | chr9 | 62644139 | 62644613 | 1 | 0 | 0 | 0 | 1 |  |
|  |  | 4 | 453 | 97.7 | 1 | 475 | chr9 | 41508184 | 41508658 | 1 | 1 | 0 | 0 | 0 | scaffold_18313 |
| IMAGE:415089 | W93379 | 1 | 395 | 99.7 | 1 | 398 | chr1 | 208915841 | 208916239 | 3 | 3 | 0 | 0 | 0 | scaffold_37640 |
|  |  | 2 | 342 | 94.6 | 11 | 398 | chr14 | 18088551 | 18088943 | 4 | 3 | 1 | 0 | 0 | scaffold_37076 |
|  |  | 3 | 347 | 94.6 | 6 | 398 | chr2 | 132145640 | 132146036 | 5 | 4 | 1 | 0 | 0 | scaffold_25436 |
|  |  | 4 | 340 | 94.3 | 11 | 398 | chr22 | 14740680 | 14741072 | 4 | 0 | 0 | 0 | 4 |  |
| IMAGE:415089 | W94994 | 1 | 505 | 96.4 | 1 | 556 | chr1 | 208915938 | 208919934 | 15 | 15 | 0 | 0 | 0 | scaffold_37640 |
|  |  | 2 | 423 | 92.4 | 1 | 540 | chr14 | 18088301 | 18088837 | 11 | 10 | 1 | 0 | 0 | scaffold_37076 |
|  |  | 3 | 424 | 92.3 | 1 | 540 | chr2 | 132145750 | 132146280 | 12 | 12 | 0 | 0 | 0 | scaffold_25436 |
|  |  | 4 | 424 | 92.6 | 1 | 540 | chr22 | 14740430 | 14740966 | 11 | 0 | 0 | 0 | 11 |  |
| IMAGE:136324 | R33482 | 1 | 235 | 95.9 | 1 | 249 | chr3 | 197885611 | 197885856 | 6 | 6 | 0 | 0 | 0 | scaffold_26859 |
| IMAGE:136324 | R34020 | 1 | 206 | 94.1 | 1 | 229 | chr15 | 19182572 | 19182796 | 3 | 0 | 0 | 0 | 3 |  |
|  |  | 2 | 265 | 92 | 1 | 321 | chr3 | 197885218 | 197885519 | 12 | 12 | 0 | 0 | 0 | scaffold_26859 |
| IMAGE:2029176 | AI253119 | 1 | 394 | 100 | 9 | 402 | chr12 | 238431 | 238824 | 1 | 0 | 1 | 0 | 0 | scaffold_37354 |
|  |  | 2 | 360 | 98.4 | 31 | 402 | chr12 | 238393 | 238764 | 1 | 0 | 1 | 0 | 0 | scaffold_37354 |
|  |  | 3 | 355 | 98.4 | 24 | 390 | chr12 | 238506 | 238872 | 1 | 0 | 1 | 0 | 0 | scaffold_37354 |
|  |  | 4 | 300 | 98.1 | 91 | 402 | chr12 | 238393 | 238704 | 1 | 0 | 1 | 0 | 0 | scaffold_37354 |
| IMAGE:2029176 | AI793064 | 1 | 570 | 99.8 | 1 | 572 | chr12 | 238534 | 239105 | 1 | 0 | 1 | 0 | 0 | scaffold_37354 |
|  |  | 2 | 327 | 98.2 | 234 | 572 | chr12 | 238474 | 238812 | 1 | 0 | 0 | 1 | 0 |  |
|  |  | 3 | 323 | 97.6 | 234 | 572 | chr12 | 238414 | 238752 | 1 | 0 | 0 | 1 | 0 |  |
|  |  | 4 | 288 | 98 | 234 | 533 | chr12 | 238393 | 238692 | 1 | 0 | 1 | 0 | 0 | scaffold_37354 |
| IMAGE:2029176 | AI793240 | 1 | 513 | 100 | 7 | 519 | chr12 | 238438 | 238950 | 1 | 0 | 1 | 0 | 0 | scaffold_37354 |
|  |  | 2 | 408 | 98.6 | 22 | 441 | chr12 | 238393 | 238812 | 1 | 0 | 1 | 0 | 0 | scaffold_37354 |
|  |  | 3 | 355 | 98.4 | 15 | 381 | chr12 | 238506 | 238872 | 1 | 0 | 1 | 0 | 0 | scaffold_37354 |
|  |  | 4 | 344 | 97.8 | 82 | 441 | chr12 | 238393 | 238752 | 1 | 0 | 1 | 0 | 0 | scaffold_37354 |
|  |  | 5 | 288 | 98 | 142 | 441 | chr12 | 238393 | 238692 | 1 | 0 | 1 | 0 | 0 | scaffold_37354 |
| IMAGE:1881469 | AI291184 | 1 | 452 | 99.8 | 15 | 468 | chr5 | 70569295 | 70569748 | 1 | 0 | 0 | 1 | 0 |  |
|  |  | 2 | 450 | 99.6 | 15 | 468 | chr5 | 69621193 | 69621646 | 1 | 0 | 0 | 0 | 1 |  |
|  |  | 3 | 452 | 99.8 | 15 | 468 | chr5 | 68958861 | 68959314 | 1 | 1 | 0 | 0 | 0 | scaffold_37073 |
| IMAGE:298862 | BX114658 | 1 | 668 | 96.7 | 6 | 715 | chr1 | 506008 | 506715 | 2 | 0 | 0 | 0 | 2 |  |
|  |  | 2 | 639 | 94.9 | 1 | 714 | chr1 | 246039871 | 246040585 | 4 | 0 | 0 | 0 | 4 |  |
|  |  | 3 | 693 | 98.6 | 6 | 715 | chr1 | 222703 | 223411 | 2 | 1 | 1 | 0 | 0 | scaffold_92 |
|  |  | 4 | 646 | 95.4 | 1 | 714 | chr10 | 135021508 | 135022221 | 3 | 2 | 1 | 0 | 0 | scaffold_28680 |
|  |  | 5 | 709 | 100 | 6 | 715 | chr16 | 90034189 | 90034898 | 1 | 0 | 1 | 0 | 0 | scaffold_30141 |
|  |  | 6 | 672 | 97.2 | 1 | 715 | chr17_random | 585680 | 586533 | 3 | 0 | 0 | 0 | 3 |  |
|  |  | 7 | 254 | 91.2 | 1 | 315 | chr18 | 88797 | 89120 | 3 | 0 | 0 | 0 | 3 |  |
|  |  | 8 | 645 | 95.4 | 1 | 714 | chr19 | 63795598 | 63796311 | 5 | 0 | 0 | 0 | 5 |  |
|  |  | 9 | 638 | 94.5 | 1 | 714 | chr19 | 207347 | 208059 | 4 | 0 | 2 | 0 | 2 | scaffold_12895 |
|  |  | 10 | 642 | 95.5 | 6 | 714 | chr2 | 114470806 | 114471516 | 3 | 0 | 1 | 0 | 2 | scaffold_16940 |
|  |  | 11 | 647 | 95.5 | 1 | 714 | chr21 | 46960335 | 46961048 | 5 | 0 | 0 | 0 | 5 |  |
|  |  | 12 | 646 | 95.8 | 6 | 714 | chr22 | 49362932 | 49363642 | 3 | 0 | 0 | 0 | 3 |  |
|  |  | 13 | 615 | 93.6 | 1 | 714 | chr4 | 191718132 | 191718854 | 5 | 0 | 0 | 0 | 5 |  |
|  |  | 14 | 615 | 93.6 | 1 | 714 | chr4 | 11468 | 12190 | 5 | 0 | 0 | 0 | 5 |  |
|  |  | 15 | 668 | 96.7 | 6 | 715 | chr5 | 181003864 | 181004571 | 2 | 1 | 1 | 0 | 0 | scaffold_13297 |
|  |  | 16 | 634 | 95.3 | 6 | 714 | chr5 | 70949 | 71658 | 2 | 0 | 0 | 0 | 2 |  |
|  |  | 17 | 683 | 98.2 | 6 | 715 | chr6 | 170906341 | 170907050 | 1 | 0 | 0 | 0 | 1 |  |
|  |  | 18 | 514 | 90.8 | 1 | 658 | chr6 | 170763878 | 170764529 | 5 | 0 | 0 | 0 | 5 |  |
|  |  | 19 | 679 | 97.9 | 6 | 715 | chr8 | 251 | 960 | 1 | 0 | 0 | 0 | 1 |  |
|  |  | 20 | 636 | 94.8 | 1 | 714 | chr8 | 157777 | 158617 | 4 | 0 | 0 | 0 | 4 |  |
| IMAGE:298862 | N75356 | 1 | 239 | 99.6 | 1 | 241 | chr1 | 222309 | 222549 | 1 | 0 | 1 | 0 | 0 | scaffold_954 |
|  |  | 2 | 223 | 94.9 | 1 | 241 | chr1 | 506869 | 507103 | 4 | 0 | 0 | 0 | 4 |  |
|  |  | 3 | 212 | 95.2 | 1 | 241 | chr1 | 246040738 | 246040977 | 5 | 0 | 0 | 0 | 5 |  |
|  |  | 4 | 212 | 95.2 | 1 | 241 | chr10 | 135022374 | 135022613 | 5 | 3 | 2 | 0 | 0 | scaffold_28680 |
|  |  | 5 | 241 | 100 | 1 | 241 | chr16 | 90035052 | 90035292 | 1 | 0 | 1 | 0 | 0 | scaffold_30141 |
|  |  | 6 | 223 | 94.9 | 1 | 241 | chr17_random | 585292 | 585526 | 4 | 0 | 0 | 0 | 4 |  |
|  |  | 7 | 214 | 94.9 | 1 | 241 | chr19 | 206953 | 207192 | 5 | 3 | 2 | 0 | 0 | scaffold_18595 |
|  |  | 8 | 212 | 95.2 | 1 | 241 | chr19 | 63796464 | 63796703 | 5 | 0 | 0 | 0 | 5 |  |
|  |  | 9 | 212 | 95.2 | 1 | 241 | chr2 | 114470414 | 114470653 | 5 | 0 | 0 | 0 | 5 |  |
|  |  | 10 | 210 | 94.8 | 1 | 241 | chr21 | 46961201 | 46961440 | 5 | 0 | 0 | 0 | 5 |  |
|  |  | 11 | 208 | 94.4 | 1 | 241 | chr22 | 49363795 | 49364034 | 5 | 0 | 0 | 0 | 5 |  |
|  |  | 12 | 206 | 95.6 | 5 | 241 | chr4 | 11073 | 11316 | 5 | 3 | 1 | 0 | 1 | scaffold_21641 |
|  |  | 13 | 204 | 94.4 | 5 | 241 | chr4 | 191719006 | 191719249 | 4 | 0 | 0 | 0 | 4 |  |
|  |  | 14 | 220 | 94.5 | 1 | 241 | chr5 | 70558 | 70794 | 3 | 0 | 0 | 0 | 3 |  |
|  |  | 15 | 223 | 94.9 | 1 | 241 | chr5 | 181004725 | 181004959 | 4 | 0 | 0 | 4 | 0 |  |
|  |  | 16 | 237 | 97.9 | 1 | 241 | chr6 | 170907204 | 170907441 | 2 | 0 | 0 | 0 | 2 |  |
|  |  | 17 | 216 | 94.1 | 1 | 241 | chr8 | 157384 | 157622 | 3 | 0 | 0 | 0 | 3 |  |
| IMAGE:298862 | W01652 | 1 | 362 | 97.9 | 2 | 375 | chr1 | 506004 | 506376 | 2 | 0 | 0 | 0 | 2 |  |
|  |  | 2 | 329 | 94.1 | 2 | 375 | chr1 | 246039871 | 246040244 | 3 | 0 | 0 | 0 | 3 |  |
|  |  | 3 | 370 | 98.7 | 2 | 375 | chr1 | 223044 | 223415 | 3 | 1 | 2 | 0 | 0 | scaffold_92 |
|  |  | 4 | 339 | 95.4 | 2 | 375 | chr10 | 135021508 | 135021881 | 3 | 2 | 1 | 0 | 0 | scaffold_28680 |
|  |  | 5 | 372 | 99.2 | 2 | 375 | chr16 | 90034185 | 90034557 | 2 | 0 | 0 | 0 | 2 |  |
|  |  | 6 | 362 | 97.9 | 2 | 375 | chr17_random | 586019 | 586391 | 2 | 0 | 0 | 0 | 2 |  |
|  |  | 7 | 251 | 91.7 | 2 | 316 | chr18 | 88797 | 89120 | 3 | 0 | 0 | 0 | 3 |  |
|  |  | 8 | 335 | 94.4 | 2 | 375 | chr19 | 63795598 | 63795970 | 4 | 0 | 0 | 0 | 4 |  |
|  |  | 9 | 325 | 93 | 2 | 375 | chr19 | 207687 | 208059 | 4 | 1 | 2 | 0 | 1 | scaffold_12895 |
|  |  | 10 | 337 | 95.2 | 2 | 375 | chr2 | 114471147 | 114471520 | 3 | 0 | 1 | 0 | 2 | scaffold_16940 |
|  |  | 11 | 337 | 94.6 | 2 | 375 | chr21 | 46960335 | 46960707 | 4 | 0 | 0 | 0 | 4 |  |
|  |  | 12 | 339 | 95.4 | 2 | 375 | chr22 | 49362928 | 49363301 | 3 | 0 | 0 | 0 | 3 |  |
|  |  | 13 | 319 | 93.4 | 2 | 375 | chr4 | 191718132 | 191718514 | 3 | 0 | 0 | 0 | 3 |  |
|  |  | 14 | 319 | 93.4 | 2 | 375 | chr4 | 11808 | 12190 | 3 | 0 | 0 | 0 | 3 |  |
|  |  | 15 | 362 | 97.9 | 2 | 375 | chr5 | 181003860 | 181004232 | 2 | 1 | 1 | 0 | 0 | scaffold_13297 |
|  |  | 16 | 331 | 95.6 | 5 | 375 | chr5 | 71289 | 71659 | 3 | 0 | 0 | 0 | 3 |  |
|  |  | 17 | 364 | 98.1 | 2 | 375 | chr6 | 170906337 | 170906709 | 2 | 0 | 0 | 0 | 2 |  |
|  |  | 18 | 282 | 90.9 | 2 | 363 | chr6 | 170763878 | 170764242 | 4 | 0 | 0 | 0 | 4 |  |
|  |  | 19 | 366 | 98.4 | 2 | 375 | chr8 | 592 | 964 | 2 | 0 | 0 | 0 | 2 |  |
|  |  | 20 | 325 | 94.8 | 5 | 375 | chr8 | 158118 | 158488 | 3 | 0 | 0 | 0 | 3 |  |
| IMAGE:1706664 | AI148329 | 1 | 495 | 99.8 | 1 | 497 | chr1 | 146266087 | 146266583 | 1 | 0 | 0 | 1 | 0 |  |
|  |  | 2 | 489 | 99.2 | 1 | 497 | chr2 | 91356951 | 91357447 | 1 | 0 | 1 | 0 | 0 | scaffold_30330 |
|  |  | 3 | 449 | 94.5 | 1 | 497 | chr4 | 9535748 | 9536241 | 2 | 1 | 1 | 0 | 0 | scaffold_37361 |
| IMAGE:1706664 | BX098416 | 1 | 730 | 99.7 | 1 | 734 | chr1 | 146265604 | 146266337 | 1 | 0 | 0 | 1 | 0 |  |
|  |  | 2 | 724 | 99.3 | 1 | 734 | chr2 | 91357197 | 91357930 | 1 | 1 | 0 | 0 | 0 | scaffold_30330 |
|  |  | 3 | 673 | 95.6 | 1 | 734 | chr4 | 9535264 | 9535995 | 3 | 1 | 2 | 0 | 0 | scaffold_37361 |
| IMAGE:429093 | AA007586 | 1 | 252 | 97 | 44 | 339 | chrUn_random | 836761 | 891810 | 4 | 0 | 0 | 1 | 3 |  |
|  |  | 2 | 287 | 96.5 | 1 | 339 | chrUn_random | 1954647 | 2014230 | 5 | 3 | 1 | 0 | 1 | scaffold_19166 scaffold_14124 |
| IMAGE:429093 | AA007587 | 1 | 258 | 93.9 | 3 | 305 | chr2 | 95027022 | 95027324 | 3 | 2 | 1 | 0 | 0 | scaffold_12453 |
|  |  | 2 | 359 | 96.1 | 3 | 394 | chrUn_random | 1952794 | 1954026 | 5 | 0 | 0 | 0 | 5 |  |
|  |  | 3 | 345 | 94.6 | 3 | 394 | chrUn_random | 892431 | 893662 | 6 | 0 | 0 | 0 | 6 |  |
| IMAGE:470261 | AA029012 | 1 | 225 | 100 | 1 | 230 | chr5 | 70287268 | 70289336 | 2 | 1 | 0 | 0 | 1 | scaffold_22334 |
|  |  | 2 | 225 | 100 | 1 | 230 | chr5 | 69535061 | 69537128 | 2 | 0 | 0 | 0 | 2 |  |
|  |  | 3 | 221 | 99.1 | 1 | 230 | chr5 | 21540712 | 21542780 | 2 | 0 | 0 | 0 | 2 |  |
|  |  | 4 | 225 | 100 | 1 | 230 | chr5 | 69997447 | 69999514 | 2 | 0 | 0 | 0 | 2 |  |
|  |  | 5 | 225 | 100 | 1 | 230 | chr5 | 69259703 | 69261770 | 2 | 0 | 0 | 0 | 2 |  |
|  |  | 6 | 225 | 100 | 1 | 230 | chr5 | 69239246 | 69241313 | 2 | 0 | 0 | 0 | 2 |  |
|  |  | 7 | 223 | 99.6 | 1 | 230 | chr6 | 26959127 | 26961197 | 2 | 0 | 1 | 0 | 1 | scaffold_18301 |
|  |  | 8 | 217 | 98.2 | 1 | 230 | chr6 | 58297557 | 58299620 | 2 | 0 | 0 | 2 | 0 |  |
|  |  | 9 | 225 | 100 | 1 | 230 | chr8_random | 1363861 | 1365929 | 2 | 0 | 0 | 0 | 2 |  |
| IMAGE:1874052 | AI339565 | 1 | 454 | 100 | 3 | 456 | chr6 | 132002570 | 132003023 | 1 | 0 | 0 | 1 | 0 |  |
|  |  | 2 | 454 | 100 | 3 | 456 | chr7 | 143347921 | 143348374 | 1 | 0 | 0 | 1 | 0 |  |
| IMAGE:1468074 | AA889397 | 1 | 211 | 98.6 | 1 | 217 | chr5 | 69959635 | 69959851 | 1 | 0 | 0 | 1 | 0 |  |
|  |  | 2 | 211 | 98.6 | 1 | 217 | chr5 | 69714384 | 69714600 | 1 | 0 | 0 | 0 | 1 |  |
|  |  | 3 | 211 | 98.6 | 1 | 217 | chr5 | 69201371 | 69201587 | 1 | 0 | 1 | 0 | 0 | scaffold_36757 |
|  |  | 4 | 217 | 100 | 1 | 217 | chr5 | 70326999 | 70327215 | 1 | 0 | 1 | 0 | 0 | scaffold_37690 |
|  |  | 5 | 211 | 98.6 | 1 | 217 | chr5 | 21989341 | 21989557 | 1 | 0 | 0 | 0 | 1 |  |
|  |  | 6 | 217 | 100 | 1 | 217 | chr8_random | 1403592 | 1403808 | 1 | 0 | 0 | 0 | 1 |  |
| IMAGE:109123 | BX098029 | 1 | 720 | 99.2 | 1 | 734 | chr1 | 146796404 | 146797138 | 2 | 0 | 2 | 0 | 0 | scaffold_37612 |
|  |  | 2 | 727 | 99.6 | 1 | 734 | chr1 | 145625717 | 145626450 | 1 | 0 | 0 | 1 | 0 |  |
|  |  | 3 | 727 | 99.6 | 1 | 734 | chr1 | 142084960 | 142085693 | 1 | 0 | 0 | 0 | 1 |  |
|  |  | 4 | 726 | 99.6 | 1 | 734 | chr1 | 142408052 | 142408786 | 2 | 0 | 0 | 0 | 2 |  |
|  |  | 5 | 724 | 99.5 | 1 | 734 | chr1 | 142227803 | 142228537 | 2 | 0 | 0 | 0 | 2 |  |
|  |  | 6 | 682 | 97 | 1 | 734 | chr5 | 49981986 | 49982723 | 4 | 2 | 2 | 0 | 0 | scaffold_37612 |
| IMAGE:109123 | T80979 | 1 | 312 | 95.4 | 1 | 332 | chr1 | 142227303 | 142227628 | 7 | 0 | 0 | 0 | 7 |  |
|  |  | 2 | 312 | 95.4 | 1 | 332 | chr1 | 145625217 | 145625542 | 7 | 0 | 0 | 7 | 0 |  |
|  |  | 3 | 312 | 95.4 | 1 | 332 | chr1 | 142084460 | 142084785 | 7 | 0 | 0 | 0 | 7 |  |
|  |  | 4 | 312 | 95.4 | 1 | 332 | chr1 | 142407552 | 142407877 | 7 | 0 | 0 | 0 | 7 |  |
|  |  | 5 | 312 | 95.4 | 1 | 332 | chr1 | 146797313 | 146797638 | 7 | 7 | 0 | 0 | 0 | scaffold_37612 |
|  |  | 6 | 290 | 92.3 | 1 | 332 | chr5 | 49982898 | 49983223 | 7 | 7 | 0 | 0 | 0 | scaffold_37612 |
| IMAGE:1638749 | AI015610 | 1 | 479 | 96.5 | 7 | 527 | chr14 | 18153949 | 18154469 | 6 | 1 | 5 | 0 | 0 | scaffold_37076 |
|  |  | 2 | 515 | 99.4 | 4 | 524 | chr2 | 130874368 | 130874888 | 1 | 0 | 0 | 0 | 1 |  |
|  |  | 3 | 418 | 93.3 | 4 | 482 | chr2 | 131265116 | 131265582 | 8 | 0 | 0 | 0 | 8 |  |
|  |  | 4 | 477 | 96 | 4 | 527 | chr2 | 132281668 | 132282193 | 5 | 2 | 3 | 0 | 0 | scaffold_33236 |
|  |  | 5 | 462 | 94.1 | 4 | 527 | chr2 | 131675061 | 131675573 | 9 | 0 | 0 | 9 | 0 |  |
|  |  | 6 | 479 | 96.5 | 7 | 527 | chr22 | 14806096 | 14806616 | 6 | 0 | 0 | 0 | 6 |  |
| IMAGE:281777 | BX108283 | 1 | 651 | 99.5 | 1 | 658 | chrUn_random | 2155830 | 2156489 | 3 | 3 | 0 | 0 | 0 | scaffold_37640 |
| IMAGE:281777 | N48085 | 1 | 448 | 97.4 | 1 | 461 | chrUn_random | 2155611 | 2156065 | 7 | 7 | 0 | 0 | 0 | scaffold_37640 |
| IMAGE:824794 | AA489069 | 1 | 326 | 100 | 31 | 356 | chr2 | 96087706 | 96088031 | 1 | 0 | 1 | 0 | 0 | scaffold_32842 |
|  |  | 2 | 316 | 98.5 | 31 | 356 | chr2 | 97316209 | 97316534 | 1 | 0 | 0 | 1 | 0 |  |
|  |  | 3 | 298 | 96.9 | 31 | 349 | chr2 | 88976016 | 88976335 | 2 | 1 | 1 | 0 | 0 | scaffold_36716 |
| IMAGE:1685642 | AI089407 | 1 | 612 | 100 | 1 | 613 | chr8 | 82404003 | 82404615 | 1 | 0 | 1 | 0 | 0 | scaffold_37639 |
| IMAGE:712622 | AA281797 | 1 | 316 | 99.1 | 1 | 318 | chr5 | 70533436 | 70533752 | 2 | 0 | 0 | 2 | 0 |  |
|  |  | 2 | 316 | 99.1 | 1 | 318 | chr5 | 69585383 | 69585699 | 2 | 0 | 0 | 0 | 2 |  |
|  |  | 3 | 316 | 99.1 | 1 | 318 | chr5 | 68994850 | 68995166 | 2 | 2 | 0 | 0 | 0 | scaffold_37073 |
| IMAGE:796775 | AA443156 | 1 | 504 | 99.8 | 1 | 506 | chr1 | 145616547 | 145617052 | 1 | 0 | 0 | 1 | 0 |  |
|  |  | 2 | 504 | 99.8 | 1 | 506 | chr1 | 142398908 | 142399413 | 1 | 0 | 0 | 0 | 1 |  |
|  |  | 3 | 504 | 99.8 | 1 | 506 | chr1 | 142218657 | 142219162 | 1 | 0 | 0 | 0 | 1 |  |
|  |  | 4 | 504 | 99.8 | 1 | 506 | chr1 | 142075808 | 142076313 | 1 | 0 | 0 | 0 | 1 |  |
|  |  | 5 | 502 | 99.6 | 1 | 506 | chr1 | 146805820 | 146806325 | 1 | 0 | 1 | 0 | 0 | scaffold_37612 |
|  |  | 6 | 468 | 96.2 | 1 | 506 | chr5 | 49991420 | 49991925 | 1 | 1 | 0 | 0 | 0 | scaffold_37612 |
| IMAGE:796775 | AA443157 | 1 | 514 | 98.9 | 1 | 527 | chr1 | 146803722 | 146804729 | 2 | 1 | 1 | 0 | 0 | scaffold_37612 |
|  |  | 2 | 516 | 99.1 | 1 | 527 | chr1 | 145618145 | 145619150 | 2 | 0 | 0 | 2 | 0 |  |
|  |  | 3 | 512 | 98.7 | 1 | 527 | chr1 | 142400504 | 142401510 | 2 | 0 | 0 | 0 | 2 |  |
|  |  | 4 | 512 | 98.7 | 1 | 527 | chr1 | 142220253 | 142221258 | 2 | 0 | 0 | 0 | 2 |  |
|  |  | 5 | 513 | 98.9 | 1 | 527 | chr1 | 142077404 | 142078412 | 3 | 0 | 0 | 0 | 3 |  |
|  |  | 6 | 464 | 95 | 13 | 527 | chr5 | 49989819 | 49990320 | 5 | 5 | 0 | 0 | 0 | scaffold_37612 |
| IMAGE:809394 | AA456585 | 1 | 296 | 96.1 | 1 | 313 | chr11 | 106232352 | 106232660 | 2 | 0 | 0 | 2 | 0 |  |
|  |  | 2 | 313 | 100 | 1 | 313 | chr12 | 21515136 | 21515448 | 1 | 0 | 1 | 0 | 0 | scaffold_37674 |
| IMAGE:1930209 | AI312926 | 1 | 431 | 99.8 | 1 | 434 | chr5 | 70567857 | 70568291 | 2 | 0 | 0 | 2 | 0 |  |
|  |  | 2 | 431 | 99.8 | 1 | 434 | chr5 | 69619755 | 69620189 | 2 | 0 | 0 | 0 | 2 |  |
|  |  | 3 | 433 | 100 | 1 | 434 | chr5 | 68960318 | 68960752 | 2 | 2 | 0 | 0 | 0 | scaffold_37073 |
| IMAGE:1634998 | AA994976 | 1 | 330 | 97.7 | 9 | 361 | chr1 | 146280851 | 146281207 | 3 | 0 | 0 | 3 | 0 |  |
|  |  | 2 | 334 | 98.3 | 9 | 361 | chr2 | 91342356 | 91342712 | 3 | 3 | 0 | 0 | 0 | scaffold_30330 |
|  |  | 3 | 351 | 99.7 | 9 | 361 | chr4 | 9550287 | 9550639 | 1 | 1 | 0 | 0 | 0 | scaffold_37361 |
| IMAGE:1634998 | BX090940 | 1 | 653 | 96.9 | 1 | 706 | chr1 | 146280851 | 146281561 | 4 | 0 | 0 | 4 | 0 |  |
|  |  | 2 | 655 | 97 | 1 | 706 | chr2 | 91342002 | 91342712 | 4 | 3 | 1 | 0 | 0 | scaffold_30330 |
|  |  | 3 | 701 | 99.7 | 1 | 706 | chr4 | 9550287 | 9550992 | 1 | 0 | 1 | 0 | 0 | scaffold_37361 |
| IMAGE:345247 | W72369 | 1 | 453 | 91.5 | 1 | 581 | chr13 | 33423180 | 33423758 | 9 | 2 | 4 | 3 | 0 | scaffold_34718 |
|  |  | 2 | 560 | 97.7 | 1 | 581 | chr2 | 132697196 | 132697773 | 4 | 4 | 0 | 0 | 0 | scaffold_37204 |
|  |  | 3 | 506 | 94.9 | 5 | 581 | chr22 | 14497564 | 14498142 | 6 | 5 | 1 | 0 | 0 | scaffold_10652 |
| IMAGE:345247 | W74030 | 1 | 347 | 93.2 | 1 | 397 | chr13 | 33422325 | 33422720 | 2 | 2 | 0 | 0 | 0 | scaffold_34718 |
|  |  | 2 | 400 | 99.5 | 1 | 405 | chr2 | 132696345 | 132697237 | 3 | 2 | 1 | 0 | 0 | scaffold_37204 |
|  |  | 3 | 372 | 96.2 | 1 | 400 | chr22 | 14498596 | 14498994 | 2 | 1 | 1 | 0 | 0 | scaffold_10652 |
| IMAGE:191530 | H37809 | 1 | 408 | 97.1 | 1 | 431 | chr5 | 69580574 | 69581000 | 5 | 0 | 0 | 0 | 5 |  |
|  |  | 2 | 408 | 97.1 | 1 | 431 | chr5 | 70528636 | 70529062 | 5 | 0 | 0 | 5 | 0 |  |
|  |  | 3 | 408 | 97.1 | 1 | 431 | chr5 | 68999547 | 68999973 | 5 | 4 | 1 | 0 | 0 | scaffold_37073 |
| IMAGE:191530 | H37860 | 1 | 446 | 96.7 | 1 | 469 | chr5 | 68999344 | 68999803 | 7 | 6 | 1 | 0 | 0 | scaffold_37073 |
|  |  | 2 | 446 | 96.7 | 1 | 469 | chr5 | 69580744 | 69581203 | 7 | 0 | 0 | 0 | 7 |  |
|  |  | 3 | 446 | 96.7 | 1 | 469 | chr5 | 70528806 | 70529265 | 7 | 0 | 0 | 7 | 0 |  |
| IMAGE:823588 | AA497050 | 1 | 405 | 99 | 20 | 428 | chr10 | 81516477 | 81516884 | 2 | 2 | 0 | 0 | 0 | scaffold_37464 |
|  |  | 2 | 325 | 91 | 20 | 428 | chr14 | 18110230 | 18110635 | 6 | 0 | 0 | 6 | 0 |  |
|  |  | 3 | 335 | 90.1 | 20 | 428 | chr2 | 130817432 | 130817837 | 5 | 0 | 0 | 5 | 0 |  |
|  |  | 4 | 325 | 91 | 20 | 428 | chr22 | 14762356 | 14762761 | 6 | 0 | 0 | 0 | 6 |  |
| IMAGE:823588 | AA497127 | 1 | 496 | 99.8 | 1 | 498 | chr10 | 81516357 | 81516854 | 1 | 1 | 0 | 0 | 0 | scaffold_37464 |
|  |  | 2 | 410 | 91.5 | 2 | 498 | chr14 | 18110260 | 18110808 | 5 | 0 | 0 | 5 | 0 |  |
|  |  | 3 | 413 | 91.7 | 1 | 498 | chr2 | 130817462 | 130817984 | 5 | 0 | 0 | 5 | 0 |  |
|  |  | 4 | 408 | 91.3 | 2 | 498 | chr22 | 14762386 | 14762934 | 5 | 0 | 0 | 0 | 5 |  |
| IMAGE:768643 | AA425630 | 1 | 421 | 100 | 1 | 422 | chr1 | 142884815 | 142885239 | 2 | 0 | 0 | 0 | 2 |  |
|  |  | 2 | 422 | 100 | 1 | 422 | chr12 | 92298094 | 92298515 | 1 | 0 | 1 | 0 | 0 | scaffold_37435 |
| IMAGE:768643 | AA430351 | 1 | 391 | 99.5 | 1 | 395 | chr1 | 142885215 | 142885609 | 1 | 0 | 0 | 0 | 1 |  |
|  |  | 2 | 391 | 99.5 | 1 | 395 | chr12 | 92297724 | 92298118 | 1 | 1 | 0 | 0 | 0 | scaffold_37435 |
| IMAGE:1940641 | AI352281 | 1 | 276 | 90.9 | 2 | 329 | chr6 | 46867171 | 46867492 | 4 | 2 | 2 | 0 | 0 | scaffold_37490 |
|  |  | 2 | 304 | 95.1 | 2 | 329 | chr9 | 63362170 | 63362494 | 4 | 4 | 0 | 0 | 0 | scaffold_21369 |
|  |  | 3 | 302 | 94.8 | 2 | 329 | chr9 | 43806039 | 43806363 | 4 | 0 | 0 | 0 | 4 |  |
| IMAGE:71432 | T47812 | 1 | 375 | 93.5 | 1 | 432 | chr16 | 15091326 | 15091753 | 6 | 5 | 1 | 0 | 0 | scaffold_6525 |
| IMAGE:71432 | T47813 | 1 | 303 | 95.2 | 1 | 347 | chr1 | 16464768 | 16465235 | 8 | 0 | 0 | 0 | 8 |  |
|  |  | 2 | 307 | 95.8 | 1 | 347 | chr1 | 16357384 | 16357851 | 8 | 0 | 0 | 8 | 0 |  |
|  |  | 3 | 323 | 98.2 | 1 | 347 | chr3 | 49680433 | 49680900 | 8 | 8 | 0 | 0 | 0 | scaffold_37645 |
| IMAGE:1641988 | AI018459 | 1 | 434 | 96.8 | 2 | 469 | chr14 | 18255262 | 18255730 | 2 | 0 | 0 | 2 | 0 |  |
|  |  | 2 | 462 | 99.8 | 2 | 469 | chr15 | 19880737 | 19881205 | 2 | 0 | 0 | 0 | 2 |  |
| IMAGE:1641988 | BX117400 | 1 | 488 | 96 | 1 | 531 | chr14 | 18254664 | 18255471 | 3 | 0 | 0 | 3 | 0 |  |
|  |  | 2 | 427 | 90.4 | 1 | 531 | chr14 | 18285768 | 18286577 | 4 | 4 | 0 | 0 | 0 | scaffold_37076 |
|  |  | 3 | 528 | 99.8 | 1 | 531 | chr15 | 19880137 | 19880946 | 2 | 0 | 0 | 0 | 2 |  |
|  |  | 4 | 364 | 90.6 | 31 | 480 | chr15 | 19911156 | 19911884 | 4 | 0 | 0 | 0 | 4 |  |
| IMAGE:1557341 | AA935790 | 1 | 316 | 96.8 | 1 | 339 | chr1 | 13035 | 14638 | 2 | 1 | 1 | 0 | 0 | scaffold_17856 |
|  |  | 2 | 324 | 97.9 | 1 | 339 | chr15 | 100241653 | 100243256 | 2 | 1 | 0 | 0 | 1 | scaffold_32713 |
|  |  | 3 | 316 | 96.8 | 1 | 339 | chr19 | 15780 | 17383 | 2 | 0 | 0 | 0 | 2 |  |
|  |  | 4 | 320 | 97.3 | 1 | 339 | chr2 | 114441388 | 114442991 | 2 | 1 | 0 | 0 | 1 | scaffold_24638 |
|  |  | 5 | 314 | 96.5 | 1 | 339 | chr9 | 13285 | 14888 | 2 | 0 | 0 | 0 | 2 |  |
| IMAGE:1557341 | AI792990 | 1 | 459 | 96.9 | 17 | 507 | chr1 | 13103 | 17507 | 3 | 3 | 0 | 0 | 0 | scaffold_17856 |
|  |  | 2 | 465 | 97.6 | 17 | 507 | chr15 | 100238783 | 100243188 | 3 | 2 | 0 | 0 | 1 | scaffold_29621 scaffold_32713 |
|  |  | 3 | 459 | 96.9 | 17 | 507 | chr19 | 15848 | 20252 | 3 | 0 | 0 | 0 | 3 |  |
|  |  | 4 | 459 | 96.9 | 17 | 507 | chr2 | 114438519 | 114442923 | 3 | 1 | 0 | 0 | 2 | scaffold_24638 |
|  |  | 5 | 457 | 96.7 | 17 | 507 | chr9 | 13353 | 17757 | 3 | 0 | 0 | 0 | 3 |  |
| IMAGE:823614 | AA490546 | 1 | 414 | 99.1 | 1 | 422 | chr11 | 56629048 | 56629469 | 1 | 0 | 0 | 1 | 0 |  |
|  |  | 2 | 422 | 100 | 1 | 422 | chr12 | 102884548 | 102884969 | 1 | 1 | 0 | 0 | 0 | scaffold_37435 |
|  |  | 3 | 408 | 98.3 | 1 | 422 | chr12 | 25849943 | 25850364 | 1 | 1 | 0 | 0 | 0 | scaffold_37674 |
| IMAGE:823614 | AA496947 | 1 | 355 | 98.6 | 57 | 421 | chr11 | 56628956 | 56629320 | 1 | 0 | 0 | 1 | 0 |  |
|  |  | 2 | 421 | 100 | 1 | 421 | chr12 | 102884697 | 102885117 | 1 | 1 | 0 | 0 | 0 | scaffold_37435 |
|  |  | 3 | 357 | 98.4 | 53 | 421 | chr12 | 25850092 | 25850460 | 1 | 0 | 1 | 0 | 0 | scaffold_37674 |
| IMAGE:1759573 | AI221541 | 1 | 289 | 99.3 | 4 | 298 | chr1 | 493690 | 493985 | 2 | 0 | 0 | 0 | 2 |  |
|  |  | 2 | 287 | 99 | 4 | 298 | chr1 | 569522 | 569817 | 2 | 0 | 0 | 0 | 2 |  |
|  |  | 3 | 290 | 99.7 | 4 | 298 | chr17_random | 598411 | 598706 | 2 | 2 | 0 | 0 | 0 | scaffold_28636 |
|  |  | 4 | 289 | 99.3 | 4 | 298 | chr5 | 180991547 | 180991842 | 2 | 0 | 0 | 0 | 2 |  |
|  |  | 5 | 287 | 99 | 4 | 298 | chr6 | 170893710 | 170894005 | 2 | 0 | 0 | 0 | 2 |  |
|  |  | 6 | 287 | 99 | 4 | 298 | chr8 | 18949 | 19244 | 2 | 0 | 0 | 0 | 2 |  |
| IMAGE:1534977 | AA918902 | 1 | 349 | 99.4 | 5 | 359 | chr1 | 16472881 | 16473237 | 3 | 0 | 0 | 0 | 3 |  |
|  |  | 2 | 341 | 98.3 | 5 | 359 | chr1 | 232036901 | 232037257 | 3 | 3 | 0 | 0 | 0 | scaffold_36933 |
| IMAGE:1534977 | AI792824 | 1 | 406 | 98.3 | 1 | 423 | chr1 | 232036822 | 232037245 | 2 | 2 | 0 | 0 | 0 | scaffold_36933 |
|  |  | 2 | 414 | 99.3 | 1 | 423 | chr1 | 16472893 | 16473316 | 2 | 0 | 0 | 0 | 2 |  |
| IMAGE:1534977 | AI822015 | 1 | 389 | 99.2 | 11 | 406 | chr1 | 16472887 | 16473283 | 2 | 0 | 0 | 0 | 2 |  |
|  |  | 2 | 381 | 98.2 | 11 | 406 | chr1 | 232036855 | 232037251 | 2 | 2 | 0 | 0 | 0 | scaffold_36933 |
| IMAGE:730649 | AA411761 | 1 | 352 | 99.4 | 4 | 359 | chr1 | 146936620 | 146936975 | 1 | 1 | 0 | 0 | 0 | scaffold_36950 |
| IMAGE:900896 | AA503815 | 1 | 334 | 99.7 | 29 | 364 | chr1 | 142412818 | 142413153 | 1 | 0 | 0 | 0 | 1 |  |
|  |  | 2 | 332 | 99.4 | 29 | 364 | chr1 | 145630508 | 145630843 | 1 | 0 | 0 | 1 | 0 |  |
|  |  | 3 | 332 | 99.4 | 29 | 364 | chr1 | 142232604 | 142232939 | 1 | 0 | 0 | 0 | 1 |  |
|  |  | 4 | 330 | 99.1 | 29 | 364 | chr1 | 142089744 | 142090079 | 1 | 0 | 0 | 0 | 1 |  |
|  |  | 5 | 328 | 98.8 | 29 | 364 | chr1 | 146792014 | 146792349 | 1 | 1 | 0 | 0 | 0 | scaffold_37612 |
|  |  | 6 | 299 | 93.3 | 29 | 364 | chr5 | 49977656 | 49977984 | 3 | 2 | 1 | 0 | 0 | scaffold_37612 |
| IMAGE:900896 | BX101993 | 1 | 360 | 98.6 | 1 | 370 | chr1 | 146791980 | 146792349 | 1 | 1 | 0 | 0 | 0 | scaffold_37612 |
|  |  | 2 | 368 | 99.7 | 1 | 370 | chr1 | 142412818 | 142413187 | 1 | 0 | 0 | 0 | 1 |  |
|  |  | 3 | 366 | 99.5 | 1 | 370 | chr1 | 145630508 | 145630877 | 1 | 0 | 0 | 1 | 0 |  |
|  |  | 4 | 366 | 99.5 | 1 | 370 | chr1 | 142232604 | 142232973 | 1 | 0 | 0 | 0 | 1 |  |
|  |  | 5 | 364 | 99.2 | 1 | 370 | chr1 | 142089744 | 142090113 | 1 | 0 | 0 | 0 | 1 |  |
|  |  | 6 | 333 | 93.9 | 1 | 370 | chr5 | 49977622 | 49977984 | 3 | 2 | 1 | 0 | 0 | scaffold_37612 |
| IMAGE:297084 | N73768 | 1 | 338 | 95.9 | 2 | 383 | chr1 | 222373 | 222758 | 5 | 5 | 0 | 0 | 0 | scaffold_954 |
|  |  | 2 | 371 | 98.7 | 1 | 386 | chr1 | 246040528 | 246040911 | 2 | 0 | 0 | 0 | 2 |  |
|  |  | 3 | 344 | 96.5 | 2 | 383 | chr1 | 506660 | 507041 | 3 | 0 | 0 | 0 | 3 |  |
|  |  | 4 | 367 | 98.1 | 1 | 386 | chr10 | 135022164 | 135022547 | 2 | 0 | 2 | 0 | 0 | scaffold_28680 |
|  |  | 5 | 333 | 94.9 | 2 | 386 | chr16 | 90034840 | 90035228 | 5 | 5 | 0 | 0 | 0 | scaffold_30141 |
|  |  | 6 | 344 | 96.5 | 2 | 383 | chr17_random | 585354 | 585735 | 3 | 0 | 0 | 0 | 3 |  |
|  |  | 7 | 385 | 95.4 | 1 | 556 | chr19 | 207019 | 207566 | 5 | 4 | 1 | 0 | 0 | scaffold_18595 scaffold_12895 |
|  |  | 8 | 369 | 98.4 | 1 | 386 | chr19 | 63796254 | 63796637 | 2 | 0 | 0 | 0 | 2 |  |
|  |  | 9 | 398 | 96.8 | 1 | 556 | chr2 | 114470480 | 114471025 | 4 | 0 | 0 | 0 | 4 |  |
|  |  | 10 | 368 | 97.9 | 1 | 386 | chr21 | 46960991 | 46961374 | 2 | 0 | 0 | 0 | 2 |  |
|  |  | 11 | 365 | 97.9 | 1 | 386 | chr22 | 49363585 | 49363968 | 2 | 0 | 0 | 0 | 2 |  |
|  |  | 12 | 313 | 94.1 | 6 | 383 | chr4 | 11143 | 11522 | 7 | 0 | 1 | 0 | 6 | scaffold_21641 |
|  |  | 13 | 310 | 94 | 6 | 383 | chr4 | 191718800 | 191719179 | 7 | 0 | 0 | 0 | 7 |  |
|  |  | 14 | 370 | 94.5 | 1 | 556 | chr5 | 70617 | 71168 | 7 | 0 | 0 | 0 | 7 |  |
|  |  | 15 | 344 | 96.5 | 2 | 383 | chr5 | 181004516 | 181004897 | 3 | 0 | 1 | 2 | 0 | scaffold_13297 |
|  |  | 16 | 358 | 95.2 | 2 | 556 | chr6 | 170764366 | 170907380 | 7 | 0 | 0 | 0 | 7 |  |
|  |  | 17 | 301 | 91.6 | 1 | 380 | chr6 | 170764534 | 170764907 | 8 | 0 | 0 | 0 | 8 |  |
|  |  | 18 | 340 | 96.2 | 1 | 386 | chr8 | 157444 | 157834 | 4 | 0 | 0 | 0 | 4 |  |
|  |  | 19 | 258 | 95.4 | 86 | 383 | chr8 | 5 | 306 | 5 | 0 | 0 | 0 | 5 |  |
| IMAGE:297084 | W03793 | 1 | 427 | 93.5 | 1 | 482 | chr1 | 246039424 | 246039901 | 5 | 0 | 0 | 0 | 5 |  |
|  |  | 2 | 427 | 93.7 | 1 | 482 | chr1 | 505557 | 506034 | 5 | 0 | 0 | 0 | 5 |  |
|  |  | 3 | 429 | 93.7 | 1 | 482 | chr1 | 223385 | 223862 | 5 | 4 | 1 | 0 | 0 | scaffold_92 |
|  |  | 4 | 457 | 96.4 | 1 | 482 | chr10 | 135021061 | 135021538 | 5 | 5 | 0 | 0 | 0 | scaffold_28680 |
|  |  | 5 | 429 | 93.7 | 1 | 482 | chr16 | 90033738 | 90034215 | 5 | 0 | 0 | 0 | 5 |  |
|  |  | 6 | 427 | 93.7 | 1 | 482 | chr17_random | 586361 | 586838 | 5 | 0 | 0 | 0 | 5 |  |
|  |  | 7 | 395 | 91.5 | 1 | 482 | chr18 | 89090 | 89572 | 7 | 0 | 0 | 0 | 7 |  |
|  |  | 8 | 461 | 96.9 | 1 | 482 | chr19 | 63795151 | 63795628 | 5 | 0 | 0 | 0 | 5 |  |
|  |  | 9 | 423 | 95.1 | 1 | 482 | chr19 | 208029 | 208538 | 8 | 0 | 0 | 0 | 8 |  |
|  |  | 10 | 467 | 97.5 | 1 | 482 | chr2 | 114471490 | 114471967 | 5 | 4 | 1 | 0 | 0 | scaffold_16940 |
|  |  | 11 | 449 | 95.6 | 1 | 482 | chr21 | 46959888 | 46960365 | 5 | 0 | 0 | 0 | 5 |  |
|  |  | 12 | 454 | 96.2 | 1 | 482 | chr22 | 49362485 | 49362958 | 6 | 0 | 0 | 0 | 6 |  |
|  |  | 13 | 397 | 91.3 | 1 | 482 | chr4 | 191717689 | 191718162 | 9 | 0 | 0 | 0 | 9 |  |
|  |  | 14 | 399 | 91.3 | 1 | 482 | chr4 | 12160 | 12633 | 9 | 4 | 2 | 0 | 3 | scaffold_32227 |
|  |  | 15 | 427 | 93.7 | 1 | 482 | chr5 | 181003413 | 181003890 | 5 | 4 | 1 | 0 | 0 | scaffold_13297 |
|  |  | 16 | 369 | 90.7 | 17 | 482 | chr5 | 1653696 | 1654146 | 9 | 9 | 0 | 0 | 0 | scaffold_34375 |
|  |  | 17 | 426 | 93.4 | 1 | 482 | chr5 | 71632 | 72107 | 7 | 0 | 0 | 0 | 7 |  |
|  |  | 18 | 415 | 92.4 | 1 | 482 | chr6 | 170905893 | 170906367 | 6 | 0 | 0 | 0 | 6 |  |
|  |  | 19 | 421 | 92.9 | 1 | 482 | chr8 | 934 | 1411 | 5 | 0 | 0 | 0 | 5 |  |
|  |  | 20 | 417 | 93.3 | 1 | 482 | chr8 | 158461 | 158935 | 6 | 0 | 0 | 0 | 6 |  |
| IMAGE:969906 | AA663895 | 1 | 347 | 99.2 | 2 | 354 | chr1 | 142102918 | 142103270 | 1 | 0 | 0 | 0 | 1 |  |
|  |  | 2 | 344 | 98.9 | 2 | 354 | chr1 | 145643651 | 145644004 | 2 | 0 | 0 | 2 | 0 |  |
|  |  | 3 | 342 | 98.6 | 2 | 354 | chr1 | 142245753 | 142246106 | 2 | 0 | 0 | 0 | 2 |  |
|  |  | 4 | 346 | 99.2 | 2 | 354 | chr1 | 146177671 | 146178024 | 2 | 0 | 0 | 2 | 0 |  |
|  |  | 5 | 345 | 98.9 | 2 | 354 | chr1 | 146778860 | 146779212 | 1 | 0 | 0 | 1 | 0 |  |
|  |  | 6 | 340 | 98.3 | 2 | 354 | chr1 | 146015677 | 146016029 | 3 | 0 | 3 | 0 | 0 | scaffold_28218 |
|  |  | 7 | 262 | 94.3 | 2 | 304 | chr5 | 49954033 | 49954335 | 5 | 2 | 3 | 0 | 0 | scaffold_37612 |
| IMAGE:743828 | BX098730 | 1 | 426 | 99.1 | 1 | 435 | chr15 | 19107504 | 19107940 | 2 | 0 | 0 | 0 | 2 |  |
| IMAGE:594758 | AA172056 | 1 | 493 | 96.4 | 2 | 573 | chr2 | 132665909 | 132666494 | 13 | 12 | 1 | 0 | 0 | scaffold_37204 |
|  |  | 2 | 510 | 98.7 | 2 | 535 | chr22 | 14559856 | 14560399 | 10 | 8 | 2 | 0 | 0 | scaffold_10652 |
| IMAGE:594758 | AA172236 | 1 | 358 | 95.5 | 1 | 390 | chr2 | 132665805 | 132666191 | 4 | 4 | 0 | 0 | 0 | scaffold_37204 |
|  |  | 2 | 377 | 97.9 | 1 | 390 | chr22 | 14560159 | 14560546 | 3 | 1 | 2 | 0 | 0 | scaffold_10652 |
| IMAGE:108471 | T70234 | 1 | 271 | 97.1 | 1 | 283 | chr12 | 107481950 | 107482230 | 3 | 2 | 1 | 0 | 0 | scaffold_37435 |
| IMAGE:108471 | T80117 | 1 | 358 | 93.7 | 1 | 405 | chr1 | 148836652 | 148837038 | 14 | 0 | 0 | 14 | 0 |  |
|  |  | 2 | 314 | 94 | 1 | 349 | chr10 | 74776462 | 74776799 | 9 | 0 | 0 | 0 | 9 |  |
|  |  | 3 | 360 | 93.9 | 1 | 405 | chr13 | 39329254 | 39329640 | 14 | 0 | 0 | 14 | 0 |  |
|  |  | 4 | 358 | 93.7 | 1 | 405 | chr19 | 47870846 | 47871232 | 14 | 0 | 0 | 14 | 0 |  |
|  |  | 5 | 358 | 93.7 | 1 | 405 | chr2 | 85691282 | 85691668 | 14 | 14 | 0 | 0 | 0 | scaffold_36190 |
|  |  | 6 | 296 | 94.8 | 1 | 323 | chr20 | 47181071 | 47181383 | 8 | 0 | 1 | 7 | 0 | scaffold_26016 |
|  |  | 7 | 351 | 93.3 | 1 | 405 | chr3 | 191513466 | 191513848 | 15 | 0 | 0 | 15 | 0 |  |
|  |  | 8 | 358 | 93.7 | 1 | 405 | chr4 | 3570000 | 3570386 | 14 | 0 | 0 | 14 | 0 |  |
|  |  | 9 | 355 | 93.7 | 1 | 405 | chr5 | 149076650 | 149077038 | 15 | 0 | 0 | 15 | 0 |  |
|  |  | 10 | 360 | 93.9 | 1 | 405 | chr8 | 81320918 | 81321304 | 14 | 0 | 0 | 14 | 0 |  |
| IMAGE:282884 | N45114 | 1 | 510 | 98.8 | 1 | 527 | chr6 | 26742590 | 26744978 | 8 | 6 | 1 | 1 | 0 | scaffold_37460 |
|  |  | 2 | 505 | 98.8 | 6 | 527 | chr9 | 95337471 | 95339849 | 8 | 0 | 0 | 8 | 0 |  |
| IMAGE:811138 | AA485730 | 1 | 393 | 98.8 | 1 | 409 | chr1 | 4269 | 4677 | 3 | 2 | 1 | 0 | 0 | scaffold_17856 |
|  |  | 2 | 396 | 98.5 | 1 | 409 | chr15 | 100251615 | 100252028 | 2 | 0 | 2 | 0 | 0 | scaffold_24036 |
|  |  | 3 | 392 | 98.3 | 1 | 409 | chr16 | 4087 | 4496 | 4 | 0 | 0 | 0 | 4 |  |
|  |  | 4 | 396 | 98.5 | 1 | 409 | chr2 | 114451344 | 114451757 | 2 | 0 | 1 | 0 | 1 | scaffold_17856 |
|  |  | 5 | 384 | 97 | 1 | 409 | chr9 | 4519 | 4925 | 4 | 0 | 1 | 0 | 3 | scaffold_17856 |
|  |  | 6 | 393 | 98 | 1 | 409 | chrX | 153686752 | 153687160 | 1 | 0 | 0 | 0 | 1 |  |
|  |  | 7 | 393 | 98 | 1 | 409 | chrY | 50280916 | 50281324 | 1 | 0 | 0 | 0 | 1 |  |
| IMAGE:811138 | AA486450 | 1 | 230 | 99.2 | 1 | 237 | chr1 | 6610 | 7134 | 4 | 3 | 1 | 0 | 0 | scaffold_17856 |
|  |  | 2 | 226 | 98.3 | 1 | 237 | chr15 | 100249156 | 100249680 | 4 | 0 | 0 | 0 | 4 |  |
|  |  | 3 | 230 | 99.2 | 1 | 237 | chr16 | 6430 | 6954 | 4 | 0 | 0 | 0 | 4 |  |
|  |  | 4 | 228 | 98.7 | 1 | 237 | chr2 | 114448887 | 114449411 | 4 | 2 | 1 | 0 | 1 | scaffold_17856 |
|  |  | 5 | 224 | 97.9 | 1 | 237 | chr9 | 6858 | 7382 | 4 | 0 | 0 | 0 | 4 |  |
|  |  | 6 | 228 | 98.7 | 1 | 237 | chrX | 153684292 | 153684816 | 4 | 0 | 0 | 0 | 4 |  |
|  |  | 7 | 228 | 98.7 | 1 | 237 | chrY | 50278456 | 50278980 | 4 | 0 | 0 | 0 | 4 |  |
| IMAGE:1461668 | AA885313 | 1 | 466 | 99.8 | 5 | 473 | chr15 | 19778450 | 19780037 | 2 | 0 | 0 | 2 | 0 |  |
|  |  | 2 | 466 | 99.8 | 5 | 473 | chr15 | 19448091 | 19449678 | 2 | 1 | 1 | 0 | 0 | scaffold_21397 |
| IMAGE:1461668 | BX109639 | 1 | 528 | 99.8 | 1 | 532 | chr15 | 19775684 | 19780037 | 3 | 0 | 0 | 3 | 0 |  |
|  |  | 2 | 528 | 99.8 | 1 | 532 | chr15 | 19445324 | 19449678 | 3 | 2 | 1 | 0 | 0 | scaffold_21397 |
| IMAGE:795343 | AA453258 | 1 | 558 | 99.1 | 3 | 571 | chr1 | 146978341 | 146979807 | 2 | 1 | 1 | 0 | 0 | scaffold_36950 |
| IMAGE:795343 | AA453359 | 1 | 589 | 99.2 | 1 | 602 | chr1 | 146930109 | 146979405 | 4 | 4 | 0 | 0 | 0 | scaffold_36950 |
| IMAGE:767345 | AA418564 | 1 | 522 | 99.1 | 1 | 533 | chr5 | 69169553 | 69170330 | 2 | 0 | 0 | 0 | 2 |  |
|  |  | 2 | 520 | 98.9 | 1 | 533 | chr5 | 69682507 | 69683331 | 2 | 1 | 1 | 0 | 0 | scaffold_7629 scaffold_29297 |
|  |  | 3 | 520 | 98.9 | 1 | 533 | chr5 | 70358257 | 70359029 | 2 | 0 | 0 | 2 | 0 |  |
|  |  | 4 | 508 | 97.7 | 1 | 533 | chr5 | 22020617 | 22021390 | 3 | 1 | 2 | 0 | 0 | scaffold_2979 |
|  |  | 5 | 520 | 98.9 | 1 | 533 | chr8_random | 1248046 | 1248818 | 2 | 0 | 0 | 0 | 2 |  |
| IMAGE:767345 | AA418633 | 1 | 594 | 99.8 | 1 | 596 | chr5 | 70358084 | 70358679 | 1 | 0 | 1 | 0 | 0 | scaffold_37690 |
|  |  | 2 | 578 | 98.2 | 1 | 596 | chr5 | 22020445 | 22021039 | 2 | 0 | 2 | 0 | 0 | scaffold_2979 |
|  |  | 3 | 596 | 100 | 1 | 596 | chr5 | 69169908 | 69170503 | 1 | 0 | 0 | 0 | 1 |  |
|  |  | 4 | 594 | 99.8 | 1 | 596 | chr5 | 69682909 | 69683504 | 1 | 0 | 1 | 0 | 0 | scaffold_29297 |
|  |  | 5 | 594 | 99.8 | 1 | 596 | chr8_random | 1247873 | 1248468 | 1 | 0 | 0 | 0 | 1 |  |
| IMAGE:384872 | AA708826 | 1 | 371 | 100 | 2 | 373 | chr17 | 73987327 | 73988167 | 2 | 1 | 1 | 0 | 0 | scaffold_37475 |
| IMAGE:292567 | N68492 | 1 | 323 | 92.7 | 1 | 450 | chr2 | 87940355 | 87940769 | 13 | 13 | 0 | 0 | 0 | scaffold_18785 |
|  |  | 2 | 231 | 97.5 | 1 | 243 | chr2 | 87364259 | 87364500 | 2 | 2 | 0 | 0 | 0 | scaffold_18785 |
|  |  | 3 | 324 | 92.4 | 1 | 450 | chr2 | 87232481 | 87232895 | 14 | 0 | 0 | 0 | 14 |  |
|  |  | 4 | 274 | 93.5 | 1 | 304 | chr2 | 112655287 | 112655583 | 8 | 0 | 0 | 8 | 0 |  |
| IMAGE:292567 | N91336 | 1 | 408 | 97.8 | 17 | 444 | chr2 | 87232338 | 87232761 | 5 | 0 | 0 | 0 | 5 |  |
|  |  | 2 | 408 | 97.8 | 17 | 444 | chr2 | 87940489 | 87940912 | 5 | 4 | 1 | 0 | 0 | scaffold_18785 |
|  |  | 3 | 204 | 94.5 | 17 | 248 | chr6 | 161023502 | 161023728 | 4 | 4 | 0 | 0 | 0 | scaffold_31971 |
| IMAGE:451095 | AA704519 | 1 | 372 | 100 | 1 | 372 | chr7 | 143362265 | 143362636 | 1 | 0 | 0 | 1 | 0 |  |
|  |  | 2 | 370 | 99.7 | 1 | 372 | chr7 | 143464667 | 143465038 | 1 | 1 | 0 | 0 | 0 | scaffold_37657 |
| IMAGE:1627621 | AI014703 | 1 | 371 | 98.2 | 10 | 395 | chr1 | 146792338 | 146793003 | 2 | 2 | 0 | 0 | 0 | scaffold_37612 |
|  |  | 2 | 224 | 98.7 | 166 | 395 | chr1 | 142089089 | 142089318 | 1 | 0 | 0 | 0 | 1 |  |
|  |  | 3 | 220 | 97.8 | 166 | 395 | chr1 | 142231950 | 142232179 | 1 | 0 | 0 | 0 | 1 |  |
|  |  | 4 | 218 | 97.4 | 166 | 395 | chr1 | 145629853 | 145630082 | 1 | 0 | 0 | 1 | 0 |  |
|  |  | 5 | 212 | 95.2 | 166 | 395 | chr1 | 142412165 | 142412393 | 2 | 0 | 0 | 0 | 2 |  |
|  |  | 6 | 334 | 93.4 | 10 | 395 | chr5 | 49977973 | 49978625 | 7 | 0 | 1 | 6 | 0 | scaffold_37612 |
| IMAGE:231802 | H92758 | 1 | 351 | 97.5 | 1 | 368 | chr17 | 18756166 | 18756530 | 4 | 0 | 0 | 0 | 4 |  |
|  |  | 2 | 353 | 97.8 | 1 | 368 | chr5 | 43571919 | 43572283 | 4 | 4 | 0 | 0 | 0 | scaffold_37592 |
| IMAGE:1031047 | AA609881 | 1 | 318 | 92.3 | 14 | 393 | chr1 | 140849890 | 140852086 | 4 | 2 | 2 | 0 | 0 | scaffold_29734 |
|  |  | 2 | 252 | 96.3 | 12 | 283 | chr2 | 95078423 | 95078694 | 1 | 1 | 0 | 0 | 0 | scaffold_12453 |
|  |  | 3 | 295 | 93 | 14 | 383 | chr4 | 49183210 | 49185417 | 4 | 0 | 0 | 0 | 4 |  |
|  |  | 4 | 238 | 92.9 | 14 | 283 | chr4 | 49446727 | 49446993 | 2 | 0 | 0 | 0 | 2 |  |
|  |  | 5 | 352 | 98.6 | 12 | 383 | chr9 | 63318551 | 63320731 | 3 | 0 | 0 | 0 | 3 |  |
|  |  | 6 | 379 | 99.7 | 12 | 393 | chr9 | 43847697 | 43849899 | 2 | 1 | 1 | 0 | 0 | scaffold_23351 scaffold_31582 |
|  |  | 7 | 224 | 91.2 | 12 | 283 | chr9 | 92591367 | 92591638 | 1 | 0 | 0 | 0 | 1 |  |
| IMAGE:321470 | W32303 | 1 | 255 | 92.4 | 15 | 316 | chr10 | 81516583 | 81516884 | 3 | 3 | 0 | 0 | 0 | scaffold_37464 |
|  |  | 2 | 323 | 94 | 15 | 417 | chr14 | 18110230 | 18110623 | 6 | 0 | 0 | 6 | 0 |  |
|  |  | 3 | 356 | 96.2 | 15 | 417 | chr2 | 130817432 | 130817825 | 7 | 0 | 0 | 7 | 0 |  |
|  |  | 4 | 321 | 93.7 | 15 | 417 | chr22 | 14762356 | 14762749 | 6 | 0 | 0 | 0 | 6 |  |
| IMAGE:321470 | W44889 | 1 | 272 | 95 | 13 | 334 | chr11 | 73153652 | 73198068 | 6 | 3 | 1 | 2 | 0 | scaffold_37360 |
|  |  | 2 | 311 | 98.4 | 13 | 334 | chr2 | 132331710 | 132332142 | 5 | 5 | 0 | 0 | 0 | scaffold_34606 |
|  |  | 3 | 355 | 98.9 | 13 | 377 | chr2 | 130829889 | 130831308 | 7 | 7 | 0 | 0 | 0 | scaffold_30883 |
| IMAGE:193990 | BX106160 | 1 | 755 | 100 | 1 | 761 | chr1 | 226615013 | 226627221 | 6 | 5 | 1 | 0 | 0 | scaffold_36933 |
|  |  | 2 | 680 | 95.8 | 6 | 758 | chr15 | 26251909 | 26252667 | 5 | 0 | 0 | 5 | 0 |  |
|  |  | 3 | 681 | 96 | 6 | 758 | chr15 | 20238520 | 20239279 | 6 | 2 | 4 | 0 | 0 | scaffold_32334 |
|  |  | 4 | 678 | 95.7 | 6 | 758 | chr15 | 26479447 | 26480205 | 5 | 0 | 0 | 0 | 5 |  |
|  |  | 5 | 686 | 95.9 | 6 | 758 | chr15_random | 283915 | 284674 | 6 | 0 | 0 | 0 | 6 |  |
| IMAGE:193990 | R83875 | 1 | 436 | 98.9 | 1 | 453 | chr1 | 226615327 | 226627222 | 10 | 10 | 0 | 0 | 0 | scaffold_36933 |
|  |  | 2 | 406 | 95.3 | 1 | 453 | chr15 | 26251734 | 26252349 | 8 | 0 | 0 | 8 | 0 |  |
|  |  | 3 | 407 | 95.5 | 1 | 453 | chr15 | 20238838 | 20239454 | 9 | 1 | 2 | 6 | 0 | scaffold_32334 |
|  |  | 4 | 404 | 95 | 1 | 453 | chr15 | 26479765 | 26480380 | 8 | 0 | 0 | 0 | 8 |  |
|  |  | 5 | 405 | 95.3 | 1 | 453 | chr15_random | 283740 | 284354 | 8 | 0 | 0 | 0 | 8 |  |
| IMAGE:193990 | R83876 | 1 | 352 | 96.4 | 1 | 370 | chr1 | 226614904 | 226615266 | 7 | 6 | 1 | 0 | 0 | scaffold_36933 |
|  |  | 2 | 311 | 92.9 | 1 | 370 | chr15 | 26479336 | 26479704 | 11 | 0 | 0 | 0 | 11 |  |
|  |  | 3 | 309 | 92.6 | 1 | 370 | chr15 | 20238409 | 20238777 | 11 | 4 | 3 | 4 | 0 | scaffold_32334 scaffold_35196 |
|  |  | 4 | 311 | 93 | 1 | 370 | chr15 | 26252410 | 26252778 | 11 | 0 | 0 | 11 | 0 |  |
|  |  | 5 | 315 | 93.3 | 1 | 370 | chr15_random | 284415 | 284785 | 11 | 0 | 0 | 0 | 11 |  |
| IMAGE:745332 | AA625642 | 1 | 359 | 99.5 | 2 | 365 | chr1 | 142091370 | 142091734 | 2 | 0 | 0 | 0 | 2 |  |
|  |  | 2 | 353 | 98.6 | 2 | 365 | chr1 | 142414449 | 142414813 | 2 | 0 | 0 | 0 | 2 |  |
|  |  | 3 | 351 | 98.4 | 2 | 365 | chr1 | 145632135 | 145632499 | 2 | 0 | 0 | 2 | 0 |  |
|  |  | 4 | 351 | 98.4 | 2 | 365 | chr1 | 142234231 | 142234595 | 2 | 0 | 0 | 0 | 2 |  |
|  |  | 5 | 351 | 98.4 | 2 | 365 | chr1 | 146790368 | 146790732 | 2 | 0 | 0 | 2 | 0 |  |
|  |  | 6 | 341 | 97 | 2 | 365 | chr5 | 49964818 | 49965182 | 2 | 2 | 0 | 0 | 0 | scaffold_37612 |
| IMAGE:745332 | BX115856 | 1 | 564 | 98.8 | 1 | 580 | chr1 | 146753744 | 146790732 | 4 | 0 | 0 | 4 | 0 |  |
|  |  | 2 | 574 | 99.7 | 1 | 580 | chr1 | 142091370 | 142271189 | 4 | 0 | 0 | 0 | 4 |  |
|  |  | 3 | 564 | 98.8 | 1 | 580 | chr1 | 145632135 | 145669062 | 4 | 0 | 0 | 4 | 0 |  |
|  |  | 4 | 505 | 98.8 | 63 | 580 | chr1 | 142234231 | 142235618 | 2 | 0 | 0 | 0 | 2 |  |
|  |  | 5 | 505 | 98.8 | 63 | 580 | chr1 | 142414449 | 142415834 | 2 | 0 | 0 | 0 | 2 |  |
|  |  | 6 | 535 | 97.2 | 1 | 580 | chr5 | 49929013 | 49965182 | 4 | 3 | 1 | 0 | 0 | scaffold_37612 |
| IMAGE:1646649 | AI025974 | 1 | 351 | 97.8 | 10 | 377 | chr9 | 40813143 | 40813511 | 3 | 0 | 0 | 0 | 3 |  |
|  |  | 2 | 366 | 99.2 | 10 | 377 | chr9 | 62675149 | 62675515 | 2 | 0 | 0 | 0 | 2 |  |
|  |  | 3 | 351 | 97.8 | 10 | 377 | chr9 | 41539159 | 41539527 | 3 | 2 | 1 | 0 | 0 | scaffold_27765 |
| IMAGE:1030854 | AA621750 | 1 | 364 | 95.5 | 7 | 408 | chr1 | 90214689 | 90215104 | 3 | 3 | 0 | 0 | 0 | scaffold_37509 |
|  |  | 2 | 325 | 90.9 | 5 | 408 | chr13 | 17090907 | 17091292 | 5 | 0 | 0 | 0 | 5 |  |
|  |  | 3 | 429 | 98.2 | 5 | 451 | chr2 | 94881277 | 94882258 | 4 | 2 | 1 | 1 | 0 | scaffold_17128 scaffold_13436 |
|  |  | 4 | 358 | 93 | 5 | 452 | chr21 | 14112844 | 14113806 | 6 | 6 | 0 | 0 | 0 | scaffold_12964 |
|  |  | 5 | 443 | 99.6 | 5 | 452 | chr9 | 44311401 | 44312381 | 2 | 0 | 0 | 0 | 2 |  |
|  |  | 6 | 443 | 99.6 | 5 | 452 | chr9 | 44055404 | 44056384 | 2 | 0 | 0 | 0 | 2 |  |
|  |  | 7 | 441 | 99.3 | 5 | 452 | chr9 | 65635525 | 65636505 | 2 | 0 | 0 | 0 | 2 |  |
| IMAGE:470930 | AA032090 | 1 | 287 | 97.4 | 1 | 312 | chr12 | 9461596 | 9461907 | 5 | 0 | 0 | 5 | 0 |  |
|  |  | 2 | 293 | 97.7 | 1 | 312 | chr12 | 31148662 | 31148972 | 3 | 3 | 0 | 0 | 0 | scaffold_37674 |
|  |  | 3 | 284 | 97 | 1 | 312 | chr12 | 9357620 | 9357931 | 4 | 3 | 1 | 0 | 0 | scaffold_36989 |
| IMAGE:470930 | AA034103 | 1 | 212 | 99.5 | 1 | 216 | chr2 | 65471508 | 65471723 | 1 | 0 | 1 | 0 | 0 | scaffold_26889 |
| IMAGE:429109 | AA004801 | 1 | 485 | 98 | 2 | 521 | chr2 | 27885378 | 27892367 | 8 | 8 | 0 | 0 | 0 | scaffold_37688 |
|  |  | 2 | 471 | 96.1 | 12 | 521 | chr9 | 30548606 | 30549109 | 6 | 0 | 0 | 6 | 0 |  |
| IMAGE:429109 | AA005047 | 1 | 291 | 98.3 | 1 | 313 | chr2 | 27882652 | 27892088 | 9 | 9 | 0 | 0 | 0 | scaffold_37688 |
|  |  | 2 | 291 | 95.3 | 1 | 313 | chr9 | 30548875 | 30549179 | 6 | 0 | 0 | 6 | 0 |  |
| IMAGE:131316 | BX091384 | 1 | 689 | 99.4 | 7 | 706 | chrUn_random | 2176963 | 2177662 | 3 | 1 | 2 | 0 | 0 | scaffold_37640 |
| IMAGE:131316 | R22949 | 1 | 319 | 95.8 | 7 | 351 | chrUn_random | 2177324 | 2177662 | 5 | 3 | 2 | 0 | 0 | scaffold_37640 |
| IMAGE:131316 | R23055 | 1 | 290 | 96 | 2 | 312 | chrUn_random | 2176958 | 2177263 | 6 | 6 | 0 | 0 | 0 | scaffold_37640 |
| IMAGE:252953 | H88598 | 1 | 349 | 96.4 | 1 | 374 | chr7 | 120544596 | 120544964 | 6 | 6 | 0 | 0 | 0 | scaffold_37671 |
|  |  | 2 | 338 | 96 | 1 | 374 | chrX | 22456367 | 22456735 | 5 | 0 | 0 | 5 | 0 |  |
| IMAGE:252953 | H88599 | 1 | 376 | 97.6 | 1 | 387 | chr7 | 120544584 | 120544966 | 5 | 5 | 0 | 0 | 0 | scaffold_37671 |
|  |  | 2 | 368 | 96.6 | 1 | 387 | chrX | 22456365 | 22456747 | 5 | 0 | 0 | 5 | 0 |  |
| IMAGE:119768 | T94409 | 1 | 317 | 100 | 1 | 319 | chr1 | 146660653 | 146660971 | 1 | 1 | 0 | 0 | 0 | scaffold_36950 |
|  |  | 2 | 317 | 100 | 1 | 319 | chr1 | 146900257 | 146900575 | 1 | 0 | 0 | 1 | 0 |  |
| IMAGE:119768 | T94500 | 1 | 281 | 97.9 | 1 | 296 | chr1 | 146900027 | 146900320 | 4 | 0 | 0 | 4 | 0 |  |
|  |  | 2 | 279 | 97.6 | 1 | 296 | chr1 | 146660423 | 146660716 | 4 | 3 | 1 | 0 | 0 | scaffold_36950 |
| IMAGE:162491 | H27752 | 1 | 305 | 95.6 | 17 | 349 | chr9 | 33374963 | 33375287 | 7 | 0 | 0 | 7 | 0 |  |
|  |  | 2 | 293 | 93.9 | 18 | 348 | chr9 | 44184216 | 44184538 | 7 | 7 | 0 | 0 | 0 | scaffold_31231 |
|  |  | 3 | 293 | 93.9 | 18 | 348 | chr9 | 64390804 | 64391126 | 7 | 6 | 1 | 0 | 0 | scaffold_31421 |
|  |  | 4 | 289 | 93.3 | 18 | 348 | chr9 | 65764379 | 65764701 | 7 | 0 | 0 | 0 | 7 |  |
|  |  | 5 | 291 | 93.6 | 18 | 348 | chr9 | 64216895 | 64217217 | 7 | 0 | 0 | 0 | 7 |  |
| IMAGE:379670 | AA778039 | 1 | 430 | 99.8 | 1 | 435 | chr17 | 42587175 | 42587612 | 4 | 2 | 2 | 0 | 0 | scaffold_37116 |
|  |  | 2 | 430 | 99.8 | 1 | 435 | chr8 | 35438791 | 35439228 | 4 | 0 | 0 | 4 | 0 |  |
| IMAGE:246820 | BX091415 | 1 | 736 | 100 | 1 | 737 | chr16 | 18574768 | 18575504 | 1 | 0 | 0 | 0 | 1 |  |
|  |  | 2 | 724 | 98.9 | 1 | 737 | chr16 | 16821815 | 16822550 | 2 | 0 | 2 | 0 | 0 | scaffold_36951 |
| IMAGE:246820 | N59089 | 1 | 367 | 97.9 | 1 | 377 | chr16 | 16821161 | 16821535 | 3 | 3 | 0 | 0 | 0 | scaffold_36951 |
|  |  | 2 | 373 | 98.7 | 1 | 377 | chr16 | 18575784 | 18576158 | 3 | 0 | 0 | 0 | 3 |  |
| IMAGE:246820 | N59493 | 1 | 278 | 97.9 | 1 | 291 | chr16 | 18574825 | 18575114 | 2 | 0 | 0 | 0 | 2 |  |
|  |  | 2 | 278 | 97.9 | 1 | 291 | chr16 | 16822204 | 16822493 | 2 | 2 | 0 | 0 | 0 | scaffold_36951 |
| IMAGE:1641894 | AI018406 | 1 | 274 | 99.6 | 1 | 276 | chr10 | 127180147 | 127180422 | 1 | 1 | 0 | 0 | 0 | scaffold_34590 |
|  |  | 2 | 259 | 97.1 | 1 | 276 | chrY | 49937616 | 49937957 | 3 | 0 | 0 | 0 | 3 |  |
| IMAGE:1641894 | BX090610 | 1 | 479 | 100 | 22 | 500 | chr10 | 127179838 | 127180316 | 1 | 0 | 1 | 0 | 0 | scaffold_34590 |
|  |  | 2 | 422 | 93.2 | 22 | 500 | chrY | 49937722 | 49938195 | 3 | 0 | 0 | 0 | 3 |  |
| IMAGE:730398 | AA469939 | 1 | 245 | 94.9 | 1 | 275 | chr2 | 96082926 | 96083206 | 3 | 1 | 2 | 0 | 0 | scaffold_33337 |
|  |  | 2 | 275 | 100 | 1 | 275 | chr2 | 88980986 | 88981260 | 1 | 0 | 1 | 0 | 0 | scaffold_36716 |
|  |  | 3 | 236 | 94 | 1 | 275 | chr2 | 97322497 | 97322771 | 3 | 0 | 0 | 3 | 0 |  |
| IMAGE:730398 | AA470109 | 1 | 264 | 91.3 | 1 | 355 | chr16 | 34178765 | 34179132 | 5 | 0 | 0 | 0 | 5 |  |
|  |  | 2 | 266 | 91.6 | 1 | 355 | chr16 | 34682518 | 34682885 | 5 | 0 | 0 | 5 | 0 |  |
|  |  | 3 | 366 | 100 | 1 | 366 | chr2 | 88980878 | 88981243 | 1 | 0 | 1 | 0 | 0 | scaffold_36716 |
|  |  | 4 | 327 | 95.8 | 1 | 364 | chr2 | 97322389 | 97322752 | 3 | 0 | 0 | 3 | 0 |  |
|  |  | 5 | 269 | 92.2 | 1 | 355 | chr2 | 97289551 | 97289924 | 6 | 0 | 0 | 6 | 0 |  |
|  |  | 6 | 332 | 95.9 | 1 | 364 | chr2 | 96082945 | 96083314 | 3 | 1 | 2 | 0 | 0 | scaffold_33337 |
|  |  | 7 | 287 | 92.6 | 1 | 351 | chr2 | 96116338 | 96116705 | 6 | 5 | 1 | 0 | 0 | scaffold_36568 |
|  |  | 8 | 270 | 92.5 | 1 | 355 | chr2 | 97801901 | 97802274 | 6 | 6 | 0 | 0 | 0 | scaffold_2082 |
|  |  | 9 | 293 | 92.6 | 1 | 355 | chrY | 27502439 | 27502806 | 6 | 0 | 0 | 0 | 6 |  |
|  |  | 10 | 283 | 91.1 | 1 | 355 | chrY | 49893050 | 49893420 | 5 | 0 | 0 | 0 | 5 |  |
| IMAGE:730398 | BX103439 | 1 | 283 | 90.6 | 1 | 384 | chr16 | 34178765 | 34179161 | 5 | 0 | 0 | 0 | 5 |  |
|  |  | 2 | 285 | 90.9 | 1 | 384 | chr16 | 34682489 | 34682885 | 5 | 0 | 0 | 5 | 0 |  |
|  |  | 3 | 384 | 100 | 1 | 384 | chr2 | 88980878 | 88981261 | 1 | 0 | 1 | 0 | 0 | scaffold_36716 |
|  |  | 4 | 343 | 95.5 | 1 | 384 | chr2 | 97322389 | 97322772 | 3 | 0 | 0 | 3 | 0 |  |
|  |  | 5 | 288 | 91.4 | 1 | 384 | chr2 | 97289551 | 97289953 | 6 | 0 | 0 | 6 | 0 |  |
|  |  | 6 | 348 | 95.6 | 1 | 384 | chr2 | 96082925 | 96083314 | 3 | 1 | 2 | 0 | 0 | scaffold_33337 |
|  |  | 7 | 308 | 91.7 | 1 | 384 | chr2 | 96116305 | 96116705 | 6 | 5 | 1 | 0 | 0 | scaffold_36568 |
|  |  | 8 | 289 | 91.7 | 1 | 384 | chr2 | 97801872 | 97802274 | 6 | 6 | 0 | 0 | 0 | scaffold_2082 |
|  |  | 9 | 303 | 91.4 | 1 | 377 | chrY | 27502439 | 27502828 | 6 | 0 | 0 | 0 | 6 |  |
| IMAGE:307337 | N95226 | 1 | 489 | 98.8 | 1 | 511 | chr6 | 46868115 | 46868631 | 6 | 5 | 1 | 0 | 0 | scaffold_37490 |
|  |  | 2 | 441 | 94.4 | 1 | 511 | chr9 | 63363121 | 63363637 | 9 | 7 | 2 | 0 | 0 | scaffold_21369 |
|  |  | 3 | 441 | 94.4 | 1 | 511 | chr9 | 43804896 | 43805412 | 9 | 0 | 0 | 0 | 9 |  |
| IMAGE:307337 | W21055 | 1 | 440 | 98 | 1 | 462 | chr6 | 46873170 | 46873627 | 6 | 6 | 0 | 0 | 0 | scaffold_37490 |
|  |  | 2 | 405 | 94.4 | 1 | 462 | chr9 | 62605797 | 62606253 | 7 | 0 | 0 | 0 | 7 |  |
| IMAGE:2461849 | AI935363 | 1 | 558 | 98.9 | 1 | 575 | chr10 | 120459218 | 120459791 | 2 | 1 | 1 | 0 | 0 | scaffold_37641 |
|  |  | 2 | 554 | 99.1 | 1 | 575 | chr2 | 70472992 | 70473570 | 4 | 0 | 0 | 4 | 0 |  |
|  |  | 3 | 434 | 91.7 | 48 | 575 | chr4 | 158883556 | 158884082 | 4 | 3 | 1 | 0 | 0 | scaffold_37184 |
| IMAGE:251404 | H97969 | 1 | 419 | 99.3 | 1 | 422 | chr7 | 17141100 | 17141520 | 2 | 2 | 0 | 0 | 0 | scaffold_34502 |
| IMAGE:328821 | W40422 | 1 | 349 | 98.3 | 1 | 364 | chr2 | 131378915 | 131379279 | 6 | 6 | 0 | 0 | 0 | scaffold_36780 |
|  |  | 2 | 349 | 98.3 | 1 | 364 | chr2 | 131443139 | 131443503 | 6 | 0 | 0 | 6 | 0 |  |
| IMAGE:328821 | W45499 | 1 | 388 | 99.5 | 1 | 392 | chr2 | 131442976 | 131443367 | 1 | 0 | 0 | 1 | 0 |  |
|  |  | 2 | 388 | 99.5 | 1 | 392 | chr2 | 131379051 | 131379442 | 1 | 1 | 0 | 0 | 0 | scaffold_36780 |
| IMAGE:1557277 | AA935533 | 1 | 487 | 100 | 1 | 487 | chr2 | 11607207 | 11607693 | 1 | 1 | 0 | 0 | 0 | scaffold_37585 |
|  |  | 2 | 479 | 99.2 | 1 | 487 | chr22 | 19819332 | 19819818 | 1 | 0 | 0 | 0 | 1 |  |
|  |  | 3 | 481 | 99.4 | 1 | 487 | chr22 | 17197159 | 17197645 | 1 | 0 | 0 | 0 | 1 |  |
|  |  | 4 | 477 | 99 | 1 | 487 | chr22 | 19948400 | 19948886 | 1 | 0 | 1 | 0 | 0 | scaffold_36542 |
| IMAGE:290337 | N64494 | 1 | 422 | 97.7 | 1 | 438 | chr3 | 95546150 | 95546585 | 3 | 0 | 0 | 3 | 0 |  |
|  |  | 2 | 424 | 98.6 | 1 | 438 | chr7 | 102299457 | 102299892 | 2 | 2 | 0 | 0 | 0 | scaffold_32051 |
| IMAGE:290337 | N92228 | 1 | 326 | 99.1 | 1 | 332 | chr3 | 95546797 | 95547128 | 1 | 0 | 0 | 1 | 0 |  |
|  |  | 2 | 330 | 100 | 1 | 332 | chr7 | 102284758 | 102293600 | 3 | 3 | 0 | 0 | 0 | scaffold_32051 |
| IMAGE:824758 | AA488782 | 1 | 448 | 98.7 | 1 | 460 | chr18 | 111606 | 112065 | 1 | 0 | 0 | 0 | 1 |  |
|  |  | 2 | 458 | 99.8 | 1 | 460 | chr18 | 16781971 | 16782430 | 1 | 1 | 0 | 0 | 0 | scaffold_37707 |
| IMAGE:824758 | AA488998 | 1 | 395 | 99.5 | 31 | 429 | chr18 | 16781875 | 16782273 | 1 | 1 | 0 | 0 | 0 | scaffold_37707 |
|  |  | 2 | 385 | 98.2 | 31 | 429 | chr18 | 111763 | 112161 | 1 | 0 | 0 | 0 | 1 |  |
| IMAGE:305677 | BX091389 | 1 | 200 | 99 | 1 | 206 | chr5 | 68985124 | 68985330 | 2 | 2 | 0 | 0 | 0 | scaffold_37073 |
|  |  | 2 | 200 | 99 | 1 | 206 | chr5 | 70543279 | 70543485 | 2 | 0 | 0 | 2 | 0 |  |
|  |  | 3 | 200 | 99 | 1 | 206 | chr5 | 69595189 | 69595395 | 2 | 0 | 0 | 0 | 2 |  |
| IMAGE:305677 | N89973 | 1 | 383 | 96 | 1 | 411 | chr5 | 69593914 | 69594316 | 7 | 0 | 0 | 0 | 7 |  |
|  |  | 2 | 379 | 95.7 | 1 | 411 | chr5 | 70542009 | 70542406 | 8 | 0 | 0 | 8 | 0 |  |
|  |  | 3 | 377 | 95.4 | 1 | 411 | chr5 | 68986203 | 68986600 | 8 | 7 | 1 | 0 | 0 | scaffold_37073 |
| IMAGE:305677 | W19716 | 1 | 275 | 100 | 2 | 277 | chr5 | 68985124 | 68985399 | 1 | 1 | 0 | 0 | 0 | scaffold_37073 |
|  |  | 2 | 275 | 100 | 2 | 277 | chr5 | 70543210 | 70543485 | 1 | 0 | 0 | 1 | 0 |  |
|  |  | 3 | 275 | 100 | 2 | 277 | chr5 | 69595120 | 69595395 | 1 | 0 | 0 | 0 | 1 |  |
| IMAGE:191877 | H40479 | 1 | 417 | 98.8 | 1 | 432 | chr5 | 68996949 | 68997387 | 7 | 6 | 1 | 0 | 0 | scaffold_37073 |
|  |  | 2 | 417 | 98.8 | 1 | 432 | chr5 | 69583160 | 69583600 | 7 | 0 | 0 | 0 | 7 |  |
|  |  | 3 | 409 | 97.9 | 1 | 432 | chr5 | 70531220 | 70531654 | 7 | 0 | 0 | 7 | 0 |  |
| IMAGE:191877 | H40480 | 1 | 261 | 95.9 | 5 | 285 | chr5 | 69582663 | 69582939 | 5 | 0 | 0 | 0 | 5 |  |
|  |  | 2 | 259 | 95.5 | 5 | 285 | chr5 | 70530723 | 70530999 | 5 | 0 | 0 | 5 | 0 |  |
|  |  | 3 | 261 | 95.9 | 5 | 285 | chr5 | 68997608 | 68997884 | 5 | 3 | 2 | 0 | 0 | scaffold_37073 |
| IMAGE:488945 | AA047077 | 1 | 257 | 98.5 | 3 | 277 | chr1 | 103472714 | 103475042 | 7 | 7 | 0 | 0 | 0 | scaffold_32862 |
|  |  | 2 | 250 | 97 | 1 | 277 | chr1 | 103519065 | 103521418 | 7 | 7 | 0 | 0 | 0 | scaffold_32862 |
|  |  | 3 | 248 | 96.6 | 1 | 277 | chr1 | 103613190 | 103615543 | 7 | 0 | 0 | 7 | 0 |  |
|  |  | 4 | 241 | 95.5 | 3 | 277 | chr1 | 103557850 | 103560208 | 7 | 0 | 0 | 7 | 0 |  |
|  |  | 5 | 241 | 95.5 | 3 | 277 | chr1 | 103651988 | 103654346 | 7 | 7 | 0 | 0 | 0 | scaffold_32862 |
|  |  | 6 | 240 | 95.4 | 3 | 277 | chr1 | 103586311 | 103588670 | 7 | 0 | 0 | 7 | 0 |  |
| IMAGE:488945 | AA047078 | 1 | 343 | 97.2 | 1 | 371 | chr1 | 103566285 | 103578672 | 11 | 0 | 0 | 11 | 0 |  |
|  |  | 2 | 343 | 97.2 | 1 | 371 | chr1 | 103495367 | 103507748 | 11 | 11 | 0 | 0 | 0 | scaffold_32862 |
| IMAGE:1554917 | AA954669 | 1 | 482 | 100 | 1 | 482 | chr9 | 40814042 | 40814523 | 1 | 0 | 0 | 0 | 1 |  |
|  |  | 2 | 482 | 100 | 1 | 482 | chr9 | 41538138 | 41538619 | 1 | 0 | 1 | 0 | 0 | scaffold_27765 |
|  |  | 3 | 465 | 98.3 | 1 | 482 | chr9 | 62674140 | 62674623 | 3 | 0 | 0 | 0 | 3 |  |
| IMAGE:1606300 | AA991180 | 1 | 316 | 99.7 | 1 | 318 | chr10 | 88349203 | 88349520 | 1 | 1 | 0 | 0 | 0 | scaffold_36993 |
|  |  | 2 | 310 | 98.7 | 1 | 318 | chr11 | 120768743 | 120769060 | 1 | 0 | 0 | 1 | 0 |  |
| IMAGE:741841 | AA402879 | 1 | 272 | 100 | 1 | 275 | chr12 | 9461569 | 9461846 | 4 | 0 | 0 | 4 | 0 |  |
|  |  | 2 | 270 | 99.6 | 1 | 275 | chr12 | 9357681 | 9357958 | 4 | 3 | 1 | 0 | 0 | scaffold_36989 |
|  |  | 3 | 256 | 97.1 | 1 | 275 | chr12 | 31148723 | 31148999 | 5 | 5 | 0 | 0 | 0 | scaffold_37674 |
| IMAGE:594438 | AA164543 | 1 | 412 | 97.3 | 1 | 446 | chr1 | 143795910 | 143796634 | 4 | 3 | 1 | 0 | 0 | scaffold_20657 |
|  |  | 2 | 393 | 94.2 | 1 | 446 | chr1 | 146487004 | 146487450 | 2 | 0 | 1 | 1 | 0 | scaffold_36950 |
|  |  | 3 | 366 | 91.8 | 1 | 446 | chr1 | 16312994 | 16313441 | 3 | 1 | 2 | 0 | 0 | scaffold_19041 |
|  |  | 4 | 435 | 98.9 | 1 | 446 | chr1 | 145930339 | 145930785 | 2 | 0 | 0 | 2 | 0 |  |
|  |  | 5 | 431 | 98.4 | 1 | 446 | chr1 | 142496578 | 142497024 | 2 | 0 | 0 | 0 | 2 |  |
|  |  | 6 | 387 | 93.5 | 1 | 446 | chr1 | 21246805 | 21247251 | 2 | 2 | 0 | 0 | 0 | scaffold_37408 |
|  |  | 7 | 437 | 99.1 | 1 | 446 | chr1_random | 996196 | 996642 | 2 | 0 | 0 | 0 | 2 |  |
| IMAGE:594438 | AA165085 | 1 | 313 | 97.2 | 6 | 333 | chr1 | 145930267 | 145930593 | 4 | 0 | 0 | 4 | 0 |  |
|  |  | 2 | 311 | 96.9 | 6 | 333 | chr1 | 142496506 | 142496832 | 4 | 0 | 1 | 0 | 3 | scaffold_28824 |
|  |  | 3 | 250 | 92.7 | 27 | 315 | chr1 | 21246754 | 21247041 | 4 | 3 | 1 | 0 | 0 | scaffold_37408 |
|  |  | 4 | 332 | 95 | 6 | 372 | chr1 | 143796112 | 143796477 | 4 | 4 | 0 | 0 | 0 | scaffold_20657 |
|  |  | 5 | 292 | 91.1 | 20 | 372 | chr1 | 146487157 | 146487508 | 4 | 0 | 1 | 3 | 0 | scaffold_36950 |
|  |  | 6 | 202 | 90.2 | 69 | 315 | chr1 | 16313205 | 16313450 | 4 | 3 | 1 | 0 | 0 | scaffold_19041 |
|  |  | 7 | 344 | 96.7 | 6 | 372 | chr1_random | 996349 | 996714 | 4 | 0 | 0 | 0 | 4 |  |
| IMAGE:815163 | AA481144 | 1 | 535 | 99.4 | 10 | 546 | chr14 | 33997601 | 33998136 | 2 | 2 | 0 | 0 | 0 | scaffold_37441 |
|  |  | 2 | 521 | 97 | 1 | 546 | chr9 | 103669300 | 103669835 | 4 | 0 | 0 | 4 | 0 |  |
| IMAGE:815163 | AA481222 | 1 | 320 | 95.7 | 1 | 351 | chr9 | 129647948 | 129648299 | 2 | 1 | 1 | 0 | 0 | scaffold_37486 |
| IMAGE:731119 | AA417272 | 1 | 480 | 100 | 1 | 480 | chr2 | 87134165 | 87134644 | 1 | 0 | 0 | 0 | 1 |  |
|  |  | 2 | 474 | 99.4 | 1 | 480 | chr2 | 113136829 | 113137308 | 1 | 0 | 1 | 0 | 0 | scaffold_25486 |
|  |  | 3 | 478 | 99.8 | 1 | 480 | chr2 | 88172113 | 88172592 | 1 | 1 | 0 | 0 | 0 | scaffold_36716 |
| IMAGE:731119 | AA417282 | 1 | 380 | 98.7 | 1 | 390 | chr2 | 88171780 | 88172169 | 1 | 0 | 1 | 0 | 0 | scaffold_36716 |
|  |  | 2 | 384 | 99.2 | 1 | 390 | chr2 | 87134588 | 87134977 | 1 | 0 | 0 | 0 | 1 |  |
|  |  | 3 | 358 | 95.3 | 1 | 390 | chr2 | 113137252 | 113137638 | 3 | 3 | 0 | 0 | 0 | scaffold_25486 |
| IMAGE:234376 | N28268 | 1 | 431 | 99.1 | 1 | 436 | chr5 | 34239230 | 34239664 | 2 | 0 | 0 | 0 | 2 |  |
|  |  | 2 | 407 | 96.3 | 1 | 436 | chr5 | 69255851 | 69256285 | 2 | 0 | 0 | 0 | 2 |  |
|  |  | 3 | 407 | 96.3 | 1 | 436 | chr5 | 69276304 | 69276738 | 2 | 0 | 0 | 0 | 2 |  |
|  |  | 4 | 407 | 96.3 | 1 | 436 | chr5 | 70014063 | 70014497 | 2 | 0 | 0 | 0 | 2 |  |
|  |  | 5 | 390 | 95.1 | 1 | 435 | chr5 | 99804740 | 99805172 | 3 | 1 | 2 | 0 | 0 | scaffold_37626 |
|  |  | 6 | 419 | 97.7 | 1 | 436 | chr5 | 21526529 | 21526963 | 2 | 0 | 0 | 0 | 2 |  |
|  |  | 7 | 407 | 96.3 | 1 | 436 | chr5 | 69522512 | 69522946 | 2 | 0 | 0 | 0 | 2 |  |
|  |  | 8 | 407 | 96.3 | 1 | 436 | chr5 | 69540544 | 69540978 | 2 | 0 | 0 | 0 | 2 |  |
|  |  | 9 | 407 | 96.3 | 1 | 436 | chr5 | 70272302 | 70272736 | 2 | 1 | 1 | 0 | 0 | scaffold_34340 |
|  |  | 10 | 394 | 95.6 | 1 | 429 | chr5 | 37515289 | 37515716 | 2 | 0 | 1 | 1 | 0 | scaffold_37592 |
|  |  | 11 | 396 | 94.6 | 1 | 435 | chr5 | 98933830 | 98934259 | 4 | 0 | 0 | 4 | 0 |  |
|  |  | 12 | 413 | 97 | 1 | 436 | chr6 | 26976035 | 26976469 | 2 | 0 | 0 | 0 | 2 |  |
|  |  | 13 | 409 | 96.5 | 1 | 436 | chr6 | 58312317 | 58312751 | 2 | 0 | 0 | 2 | 0 |  |
|  |  | 14 | 407 | 96.3 | 1 | 436 | chr8_random | 1348894 | 1349328 | 2 | 0 | 0 | 0 | 2 |  |
| IMAGE:415084 | W93369 | 1 | 338 | 99.7 | 1 | 340 | chr15 | 30165544 | 30165883 | 1 | 1 | 0 | 0 | 0 | scaffold_37002 |
|  |  | 2 | 380 | 97.2 | 1 | 405 | chr15 | 28381328 | 28381725 | 5 | 0 | 0 | 0 | 5 |  |
| IMAGE:415084 | W94963 | 1 | 488 | 98.4 | 1 | 504 | chr15 | 28380898 | 28381399 | 6 | 0 | 0 | 0 | 6 |  |
|  |  | 2 | 486 | 98.2 | 1 | 504 | chr15 | 30165870 | 30166371 | 6 | 6 | 0 | 0 | 0 | scaffold_37002 |
| IMAGE:451080 | AA704503 | 1 | 380 | 99.2 | 1 | 386 | chr1 | 493148 | 493533 | 1 | 0 | 0 | 0 | 1 |  |
|  |  | 2 | 370 | 97.9 | 1 | 386 | chr1 | 569974 | 570359 | 1 | 0 | 0 | 0 | 1 |  |
|  |  | 3 | 380 | 99.2 | 1 | 386 | chr17_random | 598863 | 599248 | 1 | 0 | 1 | 0 | 0 | scaffold_28636 |
|  |  | 4 | 380 | 99.2 | 1 | 386 | chr5 | 180991005 | 180991390 | 1 | 0 | 0 | 0 | 1 |  |
|  |  | 5 | 380 | 99.2 | 1 | 386 | chr6 | 170893168 | 170893553 | 1 | 0 | 0 | 0 | 1 |  |
|  |  | 6 | 368 | 97.7 | 1 | 386 | chr8 | 19401 | 19786 | 1 | 0 | 0 | 0 | 1 |  |
| IMAGE:344707 | W73039 | 1 | 414 | 99.8 | 28 | 443 | chr7 | 99488800 | 99489215 | 1 | 0 | 1 | 0 | 0 | scaffold_37686 |
|  |  | 2 | 382 | 95.9 | 28 | 443 | chr7 | 74494016 | 74494431 | 1 | 1 | 0 | 0 | 0 | scaffold_6353 |
|  |  | 3 | 382 | 95.9 | 28 | 443 | chr7 | 74079857 | 74080272 | 1 | 0 | 0 | 1 | 0 |  |
|  |  | 4 | 382 | 95.9 | 28 | 443 | chr7 | 73776954 | 73777369 | 1 | 0 | 0 | 1 | 0 |  |
|  |  | 5 | 382 | 95.9 | 28 | 443 | chr7 | 71966405 | 71966820 | 1 | 0 | 0 | 1 | 0 |  |
| IMAGE:344707 | W73304 | 1 | 377 | 95.9 | 1 | 413 | chr7 | 73776955 | 73777369 | 3 | 0 | 0 | 3 | 0 |  |
|  |  | 2 | 377 | 95.9 | 1 | 413 | chr7 | 71966406 | 71966820 | 3 | 0 | 0 | 3 | 0 |  |
|  |  | 3 | 409 | 99.8 | 1 | 413 | chr7 | 99488800 | 99489214 | 3 | 2 | 1 | 0 | 0 | scaffold_37686 |
|  |  | 4 | 377 | 95.9 | 1 | 413 | chr7 | 74494016 | 74494430 | 3 | 3 | 0 | 0 | 0 | scaffold_6353 |
|  |  | 5 | 377 | 95.9 | 1 | 413 | chr7 | 74079857 | 74080271 | 3 | 0 | 0 | 3 | 0 |  |
| IMAGE:323796 | AA284296 | 1 | 452 | 97.9 | 5 | 476 | chr1 | 246047470 | 246047941 | 1 | 0 | 0 | 0 | 1 |  |
|  |  | 2 | 218 | 93.6 | 5 | 254 | chr1 | 246047098 | 246047347 | 1 | 0 | 0 | 0 | 1 |  |
|  |  | 3 | 216 | 93.2 | 5 | 254 | chr1 | 246046633 | 246046882 | 1 | 0 | 0 | 0 | 1 |  |
|  |  | 4 | 219 | 94 | 5 | 254 | chr1 | 246046726 | 246047068 | 2 | 0 | 0 | 0 | 2 |  |
|  |  | 5 | 214 | 92.8 | 5 | 254 | chr1 | 217292 | 217541 | 1 | 0 | 0 | 0 | 1 |  |
|  |  | 6 | 214 | 92.8 | 5 | 254 | chr1 | 217385 | 217634 | 1 | 0 | 0 | 0 | 1 |  |
|  |  | 7 | 542 | 97.2 | 5 | 579 | chr10 | 135029692 | 135030636 | 2 | 0 | 1 | 1 | 0 | scaffold_28680 |
|  |  | 8 | 227 | 95.6 | 5 | 254 | chr10 | 135029228 | 135029569 | 3 | 0 | 0 | 3 | 0 |  |
|  |  | 9 | 222 | 94.4 | 5 | 254 | chr10 | 135029135 | 135029384 | 1 | 0 | 1 | 0 | 0 | scaffold_28680 |
|  |  | 10 | 559 | 98.6 | 5 | 579 | chr17_random | 576702 | 577276 | 1 | 0 | 0 | 0 | 1 |  |
|  |  | 11 | 228 | 95.6 | 5 | 254 | chr17_random | 577766 | 578015 | 1 | 0 | 0 | 0 | 1 |  |
|  |  | 12 | 228 | 95.6 | 5 | 254 | chr17_random | 577859 | 578108 | 1 | 0 | 0 | 0 | 1 |  |
|  |  | 13 | 216 | 93.2 | 5 | 254 | chr17_random | 578504 | 578753 | 1 | 0 | 0 | 0 | 1 |  |
|  |  | 14 | 219 | 94 | 5 | 254 | chr17_random | 578138 | 578660 | 2 | 0 | 0 | 0 | 2 |  |
|  |  | 15 | 214 | 92.8 | 5 | 254 | chr17_random | 578597 | 578846 | 1 | 0 | 0 | 0 | 1 |  |
|  |  | 16 | 220 | 94.4 | 5 | 254 | chr17_random | 577399 | 578384 | 3 | 0 | 0 | 0 | 3 |  |
|  |  | 17 | 218 | 94 | 5 | 254 | chr17_random | 577306 | 577922 | 3 | 0 | 0 | 0 | 3 |  |
|  |  | 18 | 540 | 97 | 5 | 579 | chr19 | 63803525 | 63804469 | 2 | 0 | 0 | 0 | 2 |  |
|  |  | 19 | 218 | 93.6 | 5 | 254 | chr19 | 63803432 | 63803681 | 1 | 0 | 0 | 0 | 1 |  |
|  |  | 20 | 218 | 93.6 | 5 | 254 | chr19 | 63803618 | 63803867 | 1 | 0 | 0 | 0 | 1 |  |
|  |  | 21 | 214 | 92.8 | 5 | 254 | chr19 | 63803711 | 63803960 | 1 | 0 | 0 | 0 | 1 |  |
|  |  | 22 | 212 | 92.4 | 5 | 254 | chr19 | 63803153 | 63803402 | 1 | 0 | 0 | 0 | 1 |  |
|  |  | 23 | 511 | 94.6 | 5 | 579 | chr19 | 203431 | 204193 | 3 | 0 | 0 | 0 | 3 |  |
|  |  | 24 | 551 | 97.9 | 5 | 579 | chr2 | 114462459 | 114463033 | 1 | 0 | 0 | 0 | 1 |  |
|  |  | 25 | 236 | 97.2 | 5 | 254 | chr2 | 114462877 | 114463126 | 1 | 0 | 0 | 0 | 1 |  |
|  |  | 26 | 222 | 94.4 | 5 | 254 | chr2 | 114463249 | 114463498 | 1 | 0 | 0 | 0 | 1 |  |
|  |  | 27 | 222 | 94.4 | 5 | 254 | chr2 | 114463435 | 114463684 | 1 | 0 | 0 | 0 | 1 |  |
|  |  | 28 | 218 | 93.6 | 5 | 254 | chr2 | 114463342 | 114463591 | 1 | 0 | 0 | 0 | 1 |  |
|  |  | 29 | 212 | 91.6 | 5 | 254 | chr2 | 114463714 | 114463962 | 2 | 0 | 0 | 0 | 2 |  |
|  |  | 30 | 555 | 98.3 | 5 | 579 | chr21 | 46968436 | 46969010 | 1 | 0 | 1 | 0 | 0 | scaffold_29006 |
|  |  | 31 | 224 | 95.2 | 5 | 254 | chr21 | 46967973 | 46968499 | 3 | 3 | 0 | 0 | 0 | scaffold_29006 |
|  |  | 32 | 214 | 92.8 | 5 | 254 | chr21 | 46967880 | 46968129 | 1 | 1 | 0 | 0 | 0 | scaffold_29006 |
|  |  | 33 | 573 | 99.8 | 5 | 579 | chr22 | 49371517 | 49372091 | 1 | 0 | 0 | 0 | 1 |  |
|  |  | 34 | 244 | 98.8 | 5 | 254 | chr22 | 49371424 | 49371673 | 1 | 0 | 0 | 0 | 1 |  |
|  |  | 35 | 242 | 98.4 | 5 | 254 | chr22 | 49371331 | 49371580 | 1 | 0 | 0 | 0 | 1 |  |
|  |  | 36 | 240 | 98 | 5 | 254 | chr22 | 49371238 | 49371487 | 1 | 0 | 0 | 0 | 1 |  |
|  |  | 37 | 228 | 95.6 | 5 | 254 | chr22 | 49371145 | 49371394 | 1 | 0 | 0 | 0 | 1 |  |
|  |  | 38 | 219 | 92.7 | 5 | 254 | chr22 | 49370868 | 49371115 | 2 | 0 | 0 | 0 | 2 |  |
|  |  | 39 | 216 | 93.2 | 5 | 254 | chr22 | 49370682 | 49370931 | 1 | 0 | 0 | 0 | 1 |  |
|  |  | 40 | 210 | 92 | 5 | 254 | chr22 | 49370589 | 49370838 | 1 | 0 | 0 | 0 | 1 |  |
|  |  | 41 | 210 | 92 | 5 | 254 | chr22 | 49370496 | 49370745 | 1 | 0 | 0 | 0 | 1 |  |
|  |  | 42 | 533 | 96.3 | 5 | 579 | chr5 | 181010562 | 181011136 | 1 | 0 | 1 | 0 | 0 | scaffold_13297 |
|  |  | 43 | 228 | 95.6 | 5 | 254 | chr5 | 181009818 | 181010067 | 1 | 0 | 0 | 1 | 0 |  |
|  |  | 44 | 226 | 95.2 | 5 | 254 | chr5 | 181009911 | 181010160 | 1 | 0 | 0 | 1 | 0 |  |
|  |  | 45 | 224 | 94.8 | 5 | 254 | chr5 | 181009725 | 181009974 | 1 | 0 | 0 | 1 | 0 |  |
|  |  | 46 | 217 | 93.6 | 5 | 253 | chr5 | 181010469 | 181010717 | 1 | 0 | 1 | 0 | 0 | scaffold_13297 |
|  |  | 47 | 434 | 92.2 | 64 | 579 | chr8 | 152229 | 152745 | 3 | 0 | 0 | 0 | 3 |  |
| IMAGE:323796 | BX097195 | 1 | 447 | 98.3 | 8 | 470 | chr1 | 246047479 | 246047941 | 1 | 0 | 0 | 0 | 1 |  |
|  |  | 2 | 215 | 94.6 | 8 | 248 | chr1 | 246047014 | 246047254 | 1 | 0 | 0 | 0 | 1 |  |
|  |  | 3 | 213 | 94.2 | 8 | 248 | chr1 | 246046828 | 246047068 | 1 | 0 | 0 | 0 | 1 |  |
|  |  | 4 | 212 | 94.2 | 9 | 248 | chr1 | 246046643 | 246046882 | 1 | 0 | 0 | 0 | 1 |  |
|  |  | 5 | 210 | 93.8 | 9 | 248 | chr1 | 217385 | 217624 | 1 | 0 | 0 | 0 | 1 |  |
|  |  | 6 | 209 | 93.4 | 8 | 248 | chr1 | 217292 | 217532 | 1 | 0 | 0 | 0 | 1 |  |
|  |  | 7 | 577 | 97.4 | 1 | 610 | chr10 | 135029787 | 135030673 | 2 | 0 | 1 | 1 | 0 | scaffold_28680 |
|  |  | 8 | 220 | 94.4 | 1 | 248 | chr10 | 135029137 | 135029384 | 1 | 0 | 1 | 0 | 0 | scaffold_28680 |
|  |  | 9 | 216 | 95 | 9 | 248 | chr10 | 135029052 | 135029291 | 1 | 0 | 1 | 0 | 0 | scaffold_28680 |
|  |  | 10 | 215 | 94.5 | 12 | 248 | chr10 | 135029334 | 135029569 | 2 | 0 | 0 | 2 | 0 |  |
|  |  | 11 | 594 | 98.7 | 1 | 610 | chr17_random | 576665 | 577274 | 1 | 0 | 0 | 0 | 1 |  |
|  |  | 12 | 226 | 95.6 | 1 | 248 | chr17_random | 577766 | 578013 | 1 | 0 | 0 | 0 | 1 |  |
|  |  | 13 | 223 | 96.3 | 8 | 248 | chr17_random | 577859 | 578099 | 1 | 0 | 0 | 0 | 1 |  |
|  |  | 14 | 211 | 93.8 | 8 | 248 | chr17_random | 578504 | 578744 | 1 | 0 | 0 | 0 | 1 |  |
|  |  | 15 | 214 | 94.6 | 8 | 248 | chr17_random | 578138 | 578651 | 2 | 0 | 0 | 0 | 2 |  |
|  |  | 16 | 210 | 93.8 | 9 | 248 | chr17_random | 578597 | 578836 | 1 | 0 | 0 | 0 | 1 |  |
|  |  | 17 | 219 | 94.7 | 1 | 248 | chr17_random | 577399 | 577920 | 4 | 0 | 0 | 0 | 4 |  |
|  |  | 18 | 570 | 97.3 | 8 | 610 | chr19 | 63803627 | 63804506 | 2 | 0 | 0 | 0 | 2 |  |
|  |  | 19 | 213 | 94.2 | 8 | 248 | chr19 | 63803441 | 63803681 | 1 | 0 | 0 | 0 | 1 |  |
|  |  | 20 | 208 | 93.3 | 9 | 248 | chr19 | 63803163 | 63803402 | 1 | 0 | 0 | 0 | 1 |  |
|  |  | 21 | 207 | 92.9 | 8 | 248 | chr19 | 63803255 | 63803495 | 1 | 0 | 0 | 0 | 1 |  |
|  |  | 22 | 539 | 94.9 | 8 | 610 | chr19 | 203394 | 204184 | 3 | 0 | 0 | 0 | 3 |  |
|  |  | 23 | 586 | 98 | 1 | 610 | chr2 | 114462422 | 114463031 | 1 | 0 | 0 | 0 | 1 |  |
|  |  | 24 | 234 | 97.2 | 1 | 248 | chr2 | 114462877 | 114463124 | 1 | 0 | 0 | 0 | 1 |  |
|  |  | 25 | 217 | 95 | 8 | 248 | chr2 | 114463249 | 114463489 | 1 | 0 | 0 | 0 | 1 |  |
|  |  | 26 | 217 | 95 | 8 | 248 | chr2 | 114463435 | 114463675 | 1 | 0 | 0 | 0 | 1 |  |
|  |  | 27 | 213 | 94.2 | 8 | 248 | chr2 | 114463342 | 114463582 | 1 | 0 | 0 | 0 | 1 |  |
|  |  | 28 | 208 | 92.5 | 9 | 248 | chr2 | 114463714 | 114463952 | 2 | 0 | 0 | 0 | 2 |  |
|  |  | 29 | 587 | 98.7 | 8 | 610 | chr21 | 46968445 | 46969047 | 1 | 0 | 1 | 0 | 0 | scaffold_29006 |
|  |  | 30 | 216 | 93.7 | 8 | 248 | chr21 | 46968354 | 46968592 | 2 | 1 | 1 | 0 | 0 | scaffold_29006 |
|  |  | 31 | 210 | 93.8 | 9 | 248 | chr21 | 46967890 | 46968129 | 1 | 1 | 0 | 0 | 0 | scaffold_29006 |
|  |  | 32 | 608 | 99.8 | 1 | 610 | chr22 | 49371519 | 49372128 | 1 | 0 | 0 | 0 | 1 |  |
|  |  | 33 | 242 | 98.8 | 1 | 248 | chr22 | 49371426 | 49371673 | 1 | 0 | 0 | 0 | 1 |  |
|  |  | 34 | 240 | 98.4 | 1 | 248 | chr22 | 49371333 | 49371580 | 1 | 0 | 0 | 0 | 1 |  |
|  |  | 35 | 238 | 98 | 1 | 248 | chr22 | 49371240 | 49371487 | 1 | 0 | 0 | 0 | 1 |  |
|  |  | 36 | 221 | 95.9 | 8 | 248 | chr22 | 49371061 | 49371301 | 1 | 0 | 0 | 0 | 1 |  |
|  |  | 37 | 214 | 93.3 | 8 | 248 | chr22 | 49370784 | 49371022 | 2 | 0 | 0 | 0 | 2 |  |
|  |  | 38 | 205 | 92.5 | 8 | 248 | chr22 | 49370598 | 49370838 | 1 | 0 | 0 | 0 | 1 |  |
|  |  | 39 | 568 | 96.6 | 1 | 610 | chr5 | 181010564 | 181011173 | 1 | 0 | 1 | 0 | 0 | scaffold_13297 |
|  |  | 40 | 224 | 95.2 | 1 | 248 | chr5 | 181010378 | 181010625 | 1 | 0 | 1 | 0 | 0 | scaffold_13297 |
|  |  | 41 | 223 | 96.3 | 8 | 248 | chr5 | 181009920 | 181010160 | 1 | 0 | 0 | 1 | 0 |  |
|  |  | 42 | 222 | 94.8 | 1 | 248 | chr5 | 181009727 | 181009974 | 1 | 0 | 0 | 1 | 0 |  |
|  |  | 43 | 463 | 92 | 58 | 610 | chr8 | 152192 | 152745 | 3 | 0 | 0 | 0 | 3 |  |
| IMAGE:323796 | W46143 | 1 | 402 | 97.4 | 176 | 607 | chr1 | 246047512 | 246047941 | 5 | 0 | 0 | 0 | 5 |  |
|  |  | 2 | 545 | 97.4 | 21 | 607 | chr10 | 135029084 | 135030688 | 6 | 1 | 3 | 2 | 0 | scaffold_28680 |
|  |  | 3 | 555 | 97.2 | 21 | 607 | chr17_random | 576650 | 577234 | 3 | 0 | 0 | 0 | 3 |  |
|  |  | 4 | 510 | 94.6 | 21 | 604 | chr19 | 203379 | 203963 | 6 | 0 | 0 | 0 | 6 |  |
|  |  | 5 | 541 | 96.7 | 21 | 607 | chr19 | 63803474 | 63804521 | 5 | 0 | 0 | 0 | 5 |  |
|  |  | 6 | 550 | 96.9 | 21 | 607 | chr2 | 114462407 | 114462991 | 5 | 0 | 0 | 0 | 5 |  |
|  |  | 7 | 560 | 97.8 | 21 | 607 | chr21 | 46968478 | 46969062 | 5 | 4 | 1 | 0 | 0 | scaffold_29006 |
|  |  | 8 | 568 | 99 | 21 | 607 | chr22 | 49371373 | 49372143 | 5 | 0 | 0 | 0 | 5 |  |
|  |  | 9 | 539 | 96.5 | 21 | 607 | chr5 | 181009674 | 181011188 | 6 | 1 | 2 | 3 | 0 | scaffold_13297 |
|  |  | 10 | 326 | 91.4 | 21 | 488 | chr6 | 170768613 | 170769422 | 8 | 0 | 0 | 0 | 8 |  |
|  |  | 11 | 463 | 92 | 21 | 590 | chr8 | 152177 | 152745 | 4 | 0 | 0 | 0 | 4 |  |
| IMAGE:323796 | W46155 | 1 | 285 | 95.5 | 2 | 378 | chr1 | 246047379 | 246047840 | 7 | 0 | 0 | 0 | 7 |  |
|  |  | 2 | 285 | 96.1 | 2 | 378 | chr10 | 135029787 | 135030432 | 7 | 5 | 1 | 1 | 0 | scaffold_28680 |
|  |  | 3 | 297 | 97.4 | 2 | 378 | chr17_random | 576906 | 578013 | 7 | 0 | 0 | 0 | 7 |  |
|  |  | 4 | 279 | 96.3 | 9 | 378 | chr19 | 63803627 | 63804265 | 7 | 0 | 0 | 0 | 7 |  |
|  |  | 5 | 265 | 94.3 | 9 | 372 | chr19 | 203643 | 204184 | 7 | 0 | 0 | 0 | 7 |  |
|  |  | 6 | 291 | 94.2 | 2 | 378 | chr2 | 114462663 | 114463031 | 7 | 0 | 0 | 0 | 7 |  |
|  |  | 7 | 286 | 94.4 | 9 | 378 | chr21 | 46968445 | 46968806 | 7 | 5 | 2 | 0 | 0 | scaffold_29006 |
|  |  | 8 | 301 | 95.8 | 2 | 378 | chr22 | 49371519 | 49371887 | 7 | 0 | 0 | 0 | 7 |  |
|  |  | 9 | 291 | 97.7 | 9 | 378 | chr5 | 181009920 | 181010932 | 7 | 3 | 1 | 3 | 0 | scaffold_13297 |
| IMAGE:1626304 | AI005125 | 1 | 441 | 100 | 1 | 441 | chr11 | 103315635 | 103316075 | 1 | 1 | 0 | 0 | 0 | scaffold_37627 |
| IMAGE:843429 | AA489520 | 1 | 302 | 93.9 | 1 | 406 | chr2 | 172774442 | 172785338 | 6 | 6 | 0 | 0 | 0 | scaffold_37634 |
| IMAGE:32257 | BX103613 | 1 | 524 | 99.2 | 14 | 543 | chr1 | 222952271 | 222952799 | 2 | 1 | 1 | 0 | 0 | scaffold_37457 |
|  |  | 2 | 530 | 98.9 | 1 | 543 | chr10 | 93230383 | 93230931 | 3 | 0 | 0 | 3 | 0 |  |
| IMAGE:32257 | R43360 | 1 | 337 | 95.7 | 10 | 369 | chr1 | 222952447 | 222952799 | 8 | 7 | 1 | 0 | 0 | scaffold_37457 |
|  |  | 2 | 342 | 96.9 | 1 | 369 | chr10 | 93230387 | 93230755 | 9 | 0 | 0 | 9 | 0 |  |
| IMAGE:1031896 | AA609720 | 1 | 232 | 99.2 | 1 | 236 | chr22 | 19791634 | 19791869 | 1 | 0 | 1 | 0 | 0 | scaffold_37083 |
|  |  | 2 | 234 | 99.6 | 1 | 236 | chr22 | 19976335 | 19976570 | 1 | 0 | 0 | 0 | 1 |  |
|  |  | 3 | 234 | 99.6 | 1 | 236 | chr22 | 17223614 | 17223849 | 1 | 0 | 0 | 0 | 1 |  |
| IMAGE:626842 | AA191424 | 1 | 394 | 95.7 | 1 | 424 | chr2 | 132666307 | 132666726 | 2 | 1 | 1 | 0 | 0 | scaffold_37204 |
|  |  | 2 | 423 | 100 | 1 | 424 | chr22 | 14559620 | 14560043 | 1 | 0 | 1 | 0 | 0 | scaffold_10652 |
| IMAGE:1855534 | AI306126 | 1 | 364 | 98.9 | 1 | 372 | chr10 | 46510701 | 46511072 | 1 | 1 | 0 | 0 | 0 | scaffold_33151 |
|  |  | 2 | 366 | 99.2 | 1 | 372 | chr10_random | 473013 | 473384 | 1 | 0 | 0 | 0 | 1 |  |
| IMAGE:1468630 | AA884636 | 1 | 434 | 100 | 3 | 437 | chr2 | 112436328 | 112436762 | 1 | 1 | 0 | 0 | 0 | scaffold_33337 |
|  |  | 2 | 434 | 100 | 3 | 437 | chr2 | 87479236 | 87479670 | 1 | 0 | 0 | 0 | 1 |  |
| IMAGE:1468630 | BX089889 | 1 | 748 | 99.9 | 1 | 750 | chr2 | 87478921 | 87479670 | 1 | 0 | 0 | 0 | 1 |  |
|  |  | 2 | 748 | 99.9 | 1 | 750 | chr2 | 112436328 | 112437077 | 1 | 1 | 0 | 0 | 0 | scaffold_33337 |
| IMAGE:753138 | AA400663 | 1 | 426 | 99.5 | 1 | 432 | chr7 | 142777304 | 142777740 | 3 | 0 | 0 | 3 | 0 |  |
|  |  | 2 | 429 | 99.8 | 1 | 432 | chr7 | 142941056 | 142941488 | 2 | 1 | 1 | 0 | 0 | scaffold_37657 |
| IMAGE:753138 | AA400715 | 1 | 399 | 99.8 | 1 | 402 | chr7 | 142941056 | 142941458 | 2 | 1 | 1 | 0 | 0 | scaffold_37657 |
|  |  | 2 | 396 | 99.5 | 1 | 402 | chr7 | 142777334 | 142777740 | 3 | 0 | 0 | 3 | 0 |  |
| IMAGE:126490 | BX115350 | 1 | 730 | 99.9 | 3 | 734 | chr1 | 142950293 | 142951024 | 1 | 0 | 0 | 0 | 1 |  |
|  |  | 2 | 528 | 99.3 | 199 | 734 | chr11 | 93366175 | 93366710 | 1 | 1 | 0 | 0 | 0 | scaffold_37405 |
| IMAGE:126490 | R06618 | 1 | 205 | 93.1 | 1 | 230 | chr1 | 142950769 | 142950992 | 11 | 0 | 0 | 0 | 11 |  |
| IMAGE:126490 | R06675 | 1 | 288 | 94.9 | 1 | 305 | chr1 | 142950242 | 142950537 | 9 | 0 | 0 | 0 | 9 |  |
|  |  | 2 | 282 | 94.2 | 1 | 305 | chr11 | 93366124 | 93366419 | 9 | 9 | 0 | 0 | 0 | scaffold_37405 |
| IMAGE:731433 | AA412217 | 1 | 306 | 100 | 1 | 306 | chr2 | 112621785 | 112622090 | 1 | 1 | 0 | 0 | 0 | scaffold_20264 |
|  |  | 2 | 304 | 99.7 | 1 | 306 | chr2 | 87398216 | 87398521 | 1 | 0 | 0 | 0 | 1 |  |
| IMAGE:731433 | AA470001 | 1 | 320 | 99.7 | 1 | 322 | chr2 | 87398183 | 87398504 | 1 | 0 | 0 | 0 | 1 |  |
|  |  | 2 | 322 | 100 | 1 | 322 | chr2 | 112621802 | 112622123 | 1 | 1 | 0 | 0 | 0 | scaffold_20264 |
| IMAGE:1687053 | AI094802 | 1 | 336 | 99.7 | 1 | 338 | chr1 | 12248451 | 12248788 | 1 | 1 | 0 | 0 | 0 | scaffold_34468 |
|  |  | 2 | 278 | 99.6 | 59 | 338 | chr17 | 19622963 | 19623242 | 1 | 0 | 0 | 1 | 0 |  |
| IMAGE:1707475 | AI096713 | 1 | 516 | 99.4 | 2 | 520 | chr2 | 112463134 | 112463651 | 2 | 1 | 1 | 0 | 0 | scaffold_33337 |
|  |  | 2 | 506 | 98.5 | 2 | 520 | chr2 | 87452326 | 87452843 | 2 | 0 | 0 | 0 | 2 |  |
| IMAGE:1707475 | BX101365 | 1 | 470 | 99.4 | 1 | 477 | chr2 | 87452198 | 87452675 | 2 | 0 | 0 | 0 | 2 |  |
|  |  | 2 | 476 | 100 | 1 | 477 | chr2 | 112463302 | 112463779 | 2 | 1 | 1 | 0 | 0 | scaffold_33337 |
| IMAGE:306513 | N91811 | 1 | 316 | 98.4 | 9 | 338 | chr12 | 64146627 | 64146954 | 3 | 3 | 0 | 0 | 0 | scaffold_27980 |
| IMAGE:306513 | W31245 | 1 | 416 | 96 | 2 | 438 | chr12 | 64145284 | 64145712 | 9 | 8 | 1 | 0 | 0 | scaffold_27980 |
| IMAGE:815167 | AA481146 | 1 | 564 | 100 | 11 | 574 | chr2 | 179571798 | 179572361 | 1 | 0 | 1 | 0 | 0 | scaffold_37634 |
|  |  | 2 | 311 | 90.3 | 50 | 442 | chr8 | 2888598 | 2888989 | 6 | 5 | 1 | 0 | 0 | scaffold_34487 |
| IMAGE:815167 | AA481223 | 1 | 480 | 99.4 | 1 | 486 | chr13 | 18394031 | 18394516 | 1 | 1 | 0 | 0 | 0 | scaffold_21055 |
| IMAGE:1572723 | AA969822 | 1 | 331 | 94.4 | 1 | 374 | chr2 | 95026950 | 95027324 | 2 | 2 | 0 | 0 | 0 | scaffold_12453 |
|  |  | 2 | 370 | 99.5 | 1 | 374 | chrUn_random | 1952794 | 1953167 | 1 | 0 | 0 | 0 | 1 |  |
|  |  | 3 | 336 | 94.6 | 1 | 374 | chrUn_random | 893290 | 893662 | 2 | 0 | 0 | 0 | 2 |  |
| IMAGE:897813 | AA598533 | 1 | 329 | 98.8 | 1 | 338 | chr17 | 18755371 | 18755754 | 4 | 0 | 0 | 0 | 4 |  |
|  |  | 2 | 329 | 97.3 | 1 | 338 | chr5 | 43572696 | 43573029 | 4 | 4 | 0 | 0 | 0 | scaffold_37592 |
|  |  | 3 | 203 | 90.3 | 98 | 338 | chr6 | 30260571 | 30260806 | 4 | 4 | 0 | 0 | 0 | scaffold_37619 |
| IMAGE:2029211 | AI253140 | 1 | 336 | 100 | 22 | 359 | chr18 | 11887185 | 11898259 | 3 | 3 | 0 | 0 | 0 | scaffold_37707 |
|  |  | 2 | 334 | 99.4 | 22 | 359 | chr22 | 20564488 | 20564825 | 1 | 0 | 0 | 1 | 0 |  |
| IMAGE:2029211 | AI793071 | 1 | 368 | 99.2 | 1 | 376 | chr18 | 11887189 | 11898301 | 3 | 3 | 0 | 0 | 0 | scaffold_37707 |
|  |  | 2 | 362 | 98.1 | 1 | 376 | chr22 | 20564492 | 20564867 | 1 | 0 | 0 | 1 | 0 |  |
| IMAGE:2029211 | AI793246 | 1 | 343 | 99.7 | 11 | 357 | chr18 | 11887185 | 11898268 | 3 | 3 | 0 | 0 | 0 | scaffold_37707 |
|  |  | 2 | 341 | 99.1 | 11 | 357 | chr22 | 20564488 | 20564834 | 1 | 0 | 0 | 1 | 0 |  |
| IMAGE:126229 | R06258 | 1 | 325 | 97.3 | 3 | 369 | chr1 | 16325832 | 16326198 | 10 | 9 | 1 | 0 | 0 | scaffold_20514 |
| IMAGE:126229 | R06313 | 1 | 229 | 94.8 | 1 | 264 | chr1 | 16660736 | 16660997 | 5 | 4 | 1 | 0 | 0 | scaffold_27762 |
|  |  | 2 | 244 | 97.2 | 1 | 264 | chr1 | 16327481 | 16327741 | 4 | 3 | 1 | 0 | 0 | scaffold_20514 |
| IMAGE:287618 | N62144 | 1 | 453 | 99.6 | 1 | 458 | chr2 | 179572178 | 179572635 | 1 | 0 | 1 | 0 | 0 | scaffold_37634 |
| IMAGE:1212231 | AA643410 | 1 | 300 | 99 | 8 | 313 | chr5 | 69245568 | 69245873 | 1 | 0 | 0 | 0 | 1 |  |
|  |  | 2 | 300 | 99 | 8 | 313 | chr5 | 70003781 | 70004086 | 1 | 0 | 0 | 0 | 1 |  |
|  |  | 3 | 296 | 98.4 | 8 | 313 | chr5 | 69266024 | 69266329 | 1 | 0 | 0 | 0 | 1 |  |
|  |  | 4 | 287 | 97.1 | 8 | 313 | chr5 | 99480341 | 99480648 | 2 | 0 | 0 | 2 | 0 |  |
|  |  | 5 | 283 | 96.4 | 8 | 313 | chr5 | 34228957 | 34229264 | 2 | 0 | 0 | 0 | 2 |  |
|  |  | 6 | 284 | 96.7 | 8 | 313 | chr5 | 99793787 | 99794106 | 3 | 3 | 0 | 0 | 0 | scaffold_9312 |
|  |  | 7 | 300 | 99 | 8 | 313 | chr5 | 69530490 | 69530795 | 1 | 0 | 0 | 0 | 1 |  |
|  |  | 8 | 298 | 98.7 | 8 | 313 | chr5 | 70282712 | 70283017 | 1 | 0 | 0 | 0 | 1 |  |
|  |  | 9 | 287 | 97.1 | 8 | 313 | chr5 | 98943770 | 98944077 | 2 | 0 | 0 | 2 | 0 |  |
|  |  | 10 | 283 | 96.4 | 8 | 313 | chr5 | 21536146 | 21536453 | 2 | 0 | 0 | 0 | 2 |  |
|  |  | 11 | 285 | 96.7 | 8 | 313 | chr6 | 26965426 | 26965733 | 2 | 1 | 1 | 0 | 0 | scaffold_10781 |
|  |  | 12 | 285 | 96.7 | 8 | 313 | chr6 | 58303889 | 58304196 | 2 | 0 | 0 | 2 | 0 |  |
|  |  | 13 | 298 | 98.7 | 8 | 313 | chr8_random | 1359304 | 1359609 | 1 | 0 | 0 | 0 | 1 |  |
| IMAGE:1212231 | AI791206 | 1 | 501 | 99.8 | 31 | 533 | chr5 | 69530225 | 69530727 | 1 | 0 | 0 | 0 | 1 |  |
|  |  | 2 | 499 | 99.6 | 31 | 533 | chr5 | 70282447 | 70282949 | 1 | 0 | 0 | 0 | 1 |  |
|  |  | 3 | 485 | 98.2 | 31 | 533 | chr5 | 98943505 | 98944007 | 1 | 0 | 0 | 1 | 0 |  |
|  |  | 4 | 479 | 97.6 | 31 | 533 | chr5 | 21535881 | 21536383 | 1 | 0 | 0 | 0 | 1 |  |
|  |  | 5 | 318 | 97.9 | 31 | 362 | chr5 | 37532751 | 37533082 | 1 | 1 | 0 | 0 | 0 | scaffold_37592 |
|  |  | 6 | 501 | 99.8 | 31 | 533 | chr5 | 70003849 | 70004351 | 1 | 0 | 0 | 0 | 1 |  |
|  |  | 7 | 499 | 99.6 | 31 | 533 | chr5 | 69266092 | 69266594 | 1 | 0 | 0 | 0 | 1 |  |
|  |  | 8 | 497 | 99.4 | 31 | 533 | chr5 | 69245636 | 69246138 | 1 | 0 | 0 | 0 | 1 |  |
|  |  | 9 | 479 | 97.6 | 31 | 533 | chr5 | 34229027 | 34229529 | 1 | 0 | 0 | 0 | 1 |  |
|  |  | 10 | 476 | 96.8 | 31 | 533 | chr5 | 99480411 | 99480909 | 2 | 0 | 0 | 2 | 0 |  |
|  |  | 11 | 266 | 97.5 | 253 | 533 | chr5 | 99793857 | 99794149 | 2 | 1 | 1 | 0 | 0 | scaffold_9312 |
|  |  | 12 | 214 | 98.2 | 31 | 252 | chr5 | 99794440 | 99794661 | 1 | 1 | 0 | 0 | 0 | scaffold_9312 |
|  |  | 13 | 487 | 98.4 | 31 | 533 | chr6 | 58303959 | 58304461 | 1 | 0 | 0 | 1 | 0 |  |
|  |  | 14 | 476 | 96.6 | 31 | 533 | chr6 | 26965496 | 26965994 | 2 | 0 | 2 | 0 | 0 | scaffold_10781 scaffold_8444 |
|  |  | 15 | 499 | 99.6 | 31 | 533 | chr8_random | 1359039 | 1359541 | 1 | 0 | 0 | 0 | 1 |  |
| IMAGE:1212231 | AI821940 | 1 | 457 | 100 | 9 | 465 | chr5 | 70003781 | 70004237 | 1 | 0 | 0 | 0 | 1 |  |
|  |  | 2 | 453 | 99.6 | 9 | 465 | chr5 | 69245568 | 69246024 | 1 | 0 | 0 | 0 | 1 |  |
|  |  | 3 | 453 | 99.6 | 9 | 465 | chr5 | 69266024 | 69266480 | 1 | 0 | 0 | 0 | 1 |  |
|  |  | 4 | 440 | 98.2 | 9 | 465 | chr5 | 99480341 | 99480799 | 2 | 0 | 0 | 2 | 0 |  |
|  |  | 5 | 434 | 97.6 | 9 | 465 | chr5 | 34228957 | 34229415 | 2 | 0 | 0 | 0 | 2 |  |
|  |  | 6 | 438 | 98.2 | 9 | 465 | chr5 | 99793787 | 99794547 | 4 | 3 | 1 | 0 | 0 | scaffold_9312 |
|  |  | 7 | 457 | 100 | 9 | 465 | chr5 | 69530339 | 69530795 | 1 | 0 | 0 | 0 | 1 |  |
|  |  | 8 | 455 | 99.8 | 9 | 465 | chr5 | 70282561 | 70283017 | 1 | 0 | 0 | 0 | 1 |  |
|  |  | 9 | 438 | 98 | 9 | 465 | chr5 | 98943619 | 98944077 | 2 | 0 | 0 | 2 | 0 |  |
|  |  | 10 | 434 | 97.6 | 9 | 465 | chr5 | 21535995 | 21536453 | 2 | 0 | 0 | 0 | 2 |  |
|  |  | 11 | 208 | 97.7 | 248 | 465 | chr5 | 37532865 | 37533082 | 1 | 1 | 0 | 0 | 0 | scaffold_37592 |
|  |  | 12 | 438 | 98 | 9 | 465 | chr6 | 58303889 | 58304347 | 2 | 0 | 0 | 2 | 0 |  |
|  |  | 13 | 433 | 97.1 | 9 | 465 | chr6 | 26965426 | 26965880 | 3 | 1 | 2 | 0 | 0 | scaffold_10781 scaffold_8444 |
|  |  | 14 | 455 | 99.8 | 9 | 465 | chr8_random | 1359153 | 1359609 | 1 | 0 | 0 | 0 | 1 |  |
| IMAGE:506551 | AA709048 | 1 | 383 | 99.2 | 1 | 391 | chr2 | 112119582 | 112119972 | 1 | 1 | 0 | 0 | 0 | scaffold_33337 |
|  |  | 2 | 377 | 98.5 | 1 | 391 | chr2 | 87885302 | 87885692 | 1 | 0 | 0 | 0 | 1 |  |
| IMAGE:506551 | BX095732 | 1 | 616 | 98.9 | 1 | 630 | chr2 | 87885078 | 87885707 | 1 | 0 | 0 | 0 | 1 |  |
|  |  | 2 | 626 | 99.7 | 1 | 630 | chr2 | 112119567 | 112120196 | 1 | 1 | 0 | 0 | 0 | scaffold_33337 |
| IMAGE:1534603 | AA923514 | 1 | 351 | 99.7 | 1 | 353 | chr2 | 112620364 | 112620716 | 1 | 0 | 1 | 0 | 0 | scaffold_20264 |
|  |  | 2 | 346 | 99.2 | 1 | 353 | chr2 | 87399590 | 87399943 | 2 | 0 | 0 | 0 | 2 |  |
| IMAGE:204740 | H57305 | 1 | 383 | 96.4 | 13 | 439 | chr5 | 68995994 | 68996409 | 6 | 5 | 1 | 0 | 0 | scaffold_37073 |
|  |  | 2 | 374 | 97.1 | 13 | 404 | chr5 | 69584169 | 69584555 | 5 | 0 | 0 | 0 | 5 |  |
|  |  | 3 | 370 | 96.6 | 13 | 404 | chr5 | 70532222 | 70532608 | 5 | 0 | 0 | 5 | 0 |  |
| IMAGE:240748 | H91044 | 1 | 360 | 97 | 1 | 388 | chr6 | 295295 | 295676 | 5 | 5 | 0 | 0 | 0 | scaffold_37414 |
| IMAGE:240748 | H91337 | 1 | 280 | 100 | 1 | 281 | chr6 | 295123 | 295403 | 1 | 1 | 0 | 0 | 0 | scaffold_37414 |
| **Sum** | | | | | | | | | | 4388 | 1142 | 363 | 925 | 1958 |  |
| **Average** | | | | | | | | | |  | 26.03% | 8.27% | 21.08% | 44.62% |  |

| **RANDOM** | | | | | | | | | | | | | | | |
| --- | --- | --- | --- | --- | --- | --- | --- | --- | --- | --- | --- | --- | --- | --- | --- |
| **clone** | **query** | **num** | **score** | **id** | **q_start** | **q_stop** | **chr** | **chr_start** | **chr_stop** | **block_count** | **num_chimp_pos** | **num_chimp_partial** | **num_chimp_gap** | **num_chimp_neg** | **chimp_scaffold(s)** |
| IMAGE:1047897 | AA773931 | 1 | 379 | 99.5 | 1 | 385 | chr7 | 29346162 | 29348354 | 3 | 3 | 0 | 0 | 0 | scaffold_34502 |
| IMAGE:1071184 | AA599423 | 1 | 370 | 100 | 1 | 370 | chr6 | 135224140 | 135224509 | 1 | 1 | 0 | 0 | 0 | scaffold_34646 |
| IMAGE:1089259 | AA587265 | 1 | 550 | 99.8 | 1 | 553 | chr12 | 108497237 | 108497790 | 2 | 1 | 1 | 0 | 0 | scaffold_32336 |
| IMAGE:1112263 | AA605134 | 1 | 337 | 99.1 | 1 | 345 | chr6 | 30136020 | 30138904 | 3 | 3 | 0 | 0 | 0 | scaffold_37619 |
| IMAGE:1158383 | AA631097 | 1 | 602 | 91 | 1 | 759 | chr1 | 119015497 | 119016215 | 11 | 5 | 1 | 5 | 0 | scaffold_37569 |
|  |  | 2 | 595 | 91 | 1 | 753 | chr1 | 223600980 | 223601728 | 11 | 10 | 1 | 0 | 0 | scaffold_37457 |
|  |  | 3 | 723 | 96.4 | 4 | 814 | chr10 | 85984795 | 85985639 | 15 | 15 | 0 | 0 | 0 | scaffold_34649 |
|  |  | 4 | 632 | 93.1 | 3 | 753 | chr10 | 97019023 | 97019767 | 9 | 8 | 1 | 0 | 0 | scaffold_37705 |
|  |  | 5 | 582 | 90.6 | 1 | 751 | chr12 | 8402871 | 8403608 | 16 | 13 | 3 | 0 | 0 | scaffold_20622 |
|  |  | 6 | 621 | 93.7 | 36 | 810 | chr13 | 24949047 | 24949826 | 13 | 12 | 1 | 0 | 0 | scaffold_25860 |
|  |  | 7 | 522 | 91.4 | 4 | 796 | chr14 | 33927556 | 33928645 | 16 | 16 | 0 | 0 | 0 | scaffold_37441 |
|  |  | 8 | 683 | 96.3 | 1 | 753 | chr15 | 41123907 | 41124660 | 8 | 6 | 2 | 0 | 0 | scaffold_37412 |
|  |  | 9 | 711 | 97.5 | 1 | 753 | chr15 | 57776694 | 57777447 | 8 | 6 | 2 | 0 | 0 | scaffold_37184 |
|  |  | 10 | 694 | 95.9 | 1 | 810 | chr18 | 55965801 | 55966612 | 13 | 13 | 0 | 0 | 0 | scaffold_37621 |
|  |  | 11 | 545 | 92.5 | 1 | 648 | chr2 | 160748224 | 160749182 | 5 | 5 | 0 | 0 | 0 | scaffold_37694 |
|  |  | 12 | 580 | 92.4 | 1 | 698 | chr22 | 25142541 | 25143569 | 8 | 8 | 0 | 0 | 0 | scaffold_37586 |
|  |  | 13 | 593 | 90.1 | 1 | 751 | chr3 | 75512155 | 75512892 | 14 | 12 | 1 | 0 | 1 | scaffold_20493 |
|  |  | 14 | 527 | 90.8 | 1 | 648 | chr4 | 4142934 | 4143567 | 8 | 0 | 0 | 0 | 8 |  |
|  |  | 15 | 715 | 98.6 | 3 | 753 | chr4 | 152599289 | 152603416 | 14 | 13 | 1 | 0 | 0 | scaffold_37633 |
|  |  | 16 | 728 | 96.7 | 6 | 810 | chr5 | 110604099 | 110604932 | 17 | 17 | 0 | 0 | 0 | scaffold_37656 |
|  |  | 17 | 681 | 95.1 | 1 | 753 | chr5 | 78663982 | 78664727 | 10 | 10 | 0 | 0 | 0 | scaffold_31458 |
|  |  | 18 | 586 | 91.2 | 1 | 749 | chr5 | 78088577 | 78089625 | 16 | 13 | 3 | 0 | 0 | scaffold_37690 |
|  |  | 19 | 748 | 97.2 | 1 | 814 | chr7 | 97626457 | 97627274 | 16 | 16 | 0 | 0 | 0 | scaffold_37686 |
|  |  | 20 | 674 | 95.4 | 1 | 753 | chr7 | 93936320 | 93937071 | 9 | 6 | 3 | 0 | 0 | scaffold_37515 |
|  |  | 21 | 559 | 91 | 1 | 694 | chr7 | 97139003 | 97139682 | 12 | 5 | 1 | 0 | 6 | scaffold_13730 |
| IMAGE:1203065 | AA654376 | 1 | 334 | 99.1 | 9 | 363 | chr12 | 14818870 | 14819226 | 5 | 5 | 0 | 0 | 0 | scaffold_37674 |
| IMAGE:1203365 | AA657424 | 1 | 317 | 98.8 | 1 | 331 | chr19 | 59396874 | 59402076 | 6 | 6 | 0 | 0 | 0 | scaffold_37207 |
|  |  | 2 | 299 | 95.7 | 1 | 331 | chr19 | 54914810 | 54915141 | 4 | 4 | 0 | 0 | 0 | scaffold_36551 |
|  |  | 3 | 257 | 90.9 | 6 | 331 | chr21 | 36425158 | 36425482 | 5 | 5 | 0 | 0 | 0 | scaffold_37138 |
|  |  | 4 | 297 | 95.4 | 1 | 331 | chr22 | 39703104 | 39703435 | 4 | 4 | 0 | 0 | 0 | scaffold_37547 |
| IMAGE:1269681 | AA747563 | 1 | 323 | 99.7 | 1 | 325 | chr1 | 99999469 | 99999793 | 1 | 0 | 1 | 0 | 0 | scaffold_37180 |
| IMAGE:1284299 | AA748861 | 1 | 240 | 99.2 | 8 | 251 | chr11 | 45918420 | 45918663 | 1 | 1 | 0 | 0 | 0 | scaffold_37695 |
| IMAGE:130476 | R21832 | 1 | 322 | 96.4 | 1 | 342 | chr17 | 41846415 | 41846753 | 4 | 2 | 1 | 1 | 0 | scaffold_37479 |
|  |  | 2 | 322 | 96.4 | 1 | 342 | chr17_random | 27730 | 28068 | 4 | 0 | 0 | 0 | 4 |  |
| IMAGE:130476 | R22363 | 1 | 359 | 98.9 | 1 | 369 | chr7 | 93128077 | 93130409 | 6 | 6 | 0 | 0 | 0 | scaffold_37515 |
| IMAGE:1324483 | AA768761 | 1 | 292 | 97.4 | 2 | 316 | chr12 | 47602013 | 47603903 | 4 | 4 | 0 | 0 | 0 | scaffold_37077 |
| IMAGE:1333714 | AA811805 | 1 | 423 | 99.5 | 1 | 435 | chr6 | 31854648 | 31856532 | 7 | 7 | 0 | 0 | 0 | scaffold_37619 |
| IMAGE:1466547 | AA885139 | 1 | 320 | 100 | 3 | 323 | chr19 | 1386362 | 1386683 | 2 | 0 | 2 | 0 | 0 | scaffold_34636 |
| IMAGE:1560202 | AA948127 | 1 | 459 | 96 | 3 | 524 | chr20 | 43030029 | 43030551 | 4 | 4 | 0 | 0 | 0 | scaffold_37443 |
| IMAGE:1563185 | AA917806 | 1 | 217 | 99.5 | 1 | 220 | chr4 | 75490394 | 75490614 | 2 | 0 | 1 | 1 | 0 | scaffold_37596 |
| IMAGE:1598330 | AA961704 | 1 | 449 | 100 | 1 | 454 | chr8 | 61479749 | 61554490 | 6 | 6 | 0 | 0 | 0 | scaffold_37639 |
| IMAGE:1616289 | AA985155 | 1 | 215 | 100 | 1 | 215 | chr2 | 199209397 | 199209611 | 1 | 0 | 1 | 0 | 0 | scaffold_37634 |
| IMAGE:1618733 | AI015309 | 1 | 416 | 99 | 3 | 422 | chr14 | 101799131 | 101799549 | 2 | 1 | 1 | 0 | 0 | scaffold_37603 |
|  |  | 2 | 379 | 94.5 | 1 | 422 | chr20 | 33505144 | 33505564 | 4 | 3 | 1 | 0 | 0 | scaffold_37132 |
| IMAGE:1629239 | AI003372 | 1 | 223 | 99.1 | 27 | 255 | chr10 | 51558138 | 51558413 | 3 | 1 | 2 | 0 | 0 | scaffold_37597 |
| IMAGE:1644905 | AI027738 | 1 | 392 | 97.1 | 4 | 423 | chr5 | 150436640 | 150437059 | 1 | 1 | 0 | 0 | 0 | scaffold_37144 |
| IMAGE:1688909 | AI140444 | 1 | 356 | 100 | 18 | 373 | chr4 | 1684293 | 1684648 | 1 | 0 | 0 | 1 | 0 |  |
| IMAGE:1698549 | AI142501 | 1 | 444 | 99.3 | 1 | 450 | chr20 | 37442293 | 37442742 | 1 | 1 | 0 | 0 | 0 | scaffold_37418 |
| IMAGE:1714689 | AI128422 | 1 | 411 | 99.8 | 1 | 413 | chr3 | 30024526 | 30024938 | 1 | 0 | 1 | 0 | 0 | scaffold_37683 |
| IMAGE:1741125 | AI186876 | 1 | 412 | 90.6 | 12 | 510 | chr1 | 9346194 | 9346685 | 4 | 4 | 0 | 0 | 0 | scaffold_37442 |
|  |  | 2 | 502 | 93.9 | 4 | 602 | chr10 | 44193862 | 44194528 | 7 | 6 | 1 | 0 | 0 | scaffold_37643 |
|  |  | 3 | 507 | 92.6 | 1 | 600 | chr14 | 33721788 | 33722387 | 5 | 5 | 0 | 0 | 0 | scaffold_37441 |
|  |  | 4 | 600 | 99.8 | 1 | 607 | chr15 | 80766765 | 80767376 | 6 | 4 | 2 | 0 | 0 | scaffold_37430 |
|  |  | 5 | 600 | 99.8 | 1 | 607 | chr15 | 80380224 | 80380835 | 6 | 0 | 1 | 0 | 5 | scaffold_36547 |
|  |  | 6 | 552 | 96.2 | 1 | 607 | chr15 | 64387215 | 64387995 | 9 | 7 | 1 | 1 | 0 | scaffold_37693 |
|  |  | 7 | 513 | 92.7 | 1 | 607 | chr15 | 38488894 | 38489494 | 9 | 8 | 1 | 0 | 0 | scaffold_37412 |
|  |  | 8 | 493 | 92 | 9 | 602 | chr17 | 47243015 | 47243612 | 5 | 5 | 0 | 0 | 0 | scaffold_37549 |
|  |  | 9 | 471 | 91.1 | 4 | 607 | chr17 | 15547595 | 15548177 | 8 | 8 | 0 | 0 | 0 | scaffold_37659 |
|  |  | 10 | 544 | 96.1 | 1 | 602 | chr19 | 35138729 | 35139653 | 8 | 7 | 1 | 0 | 0 | scaffold_32026 |
|  |  | 11 | 479 | 90.8 | 6 | 607 | chr2 | 209098548 | 209099144 | 8 | 8 | 0 | 0 | 0 | scaffold_36996 |
|  |  | 12 | 523 | 99.8 | 11 | 548 | chr4 | 39352937 | 39356515 | 6 | 6 | 0 | 0 | 0 | scaffold_37596 |
|  |  | 13 | 506 | 92.8 | 1 | 607 | chr5 | 31867880 | 31868858 | 9 | 8 | 1 | 0 | 0 | scaffold_37078 |
|  |  | 14 | 536 | 94.6 | 2 | 602 | chr6 | 64322467 | 64323062 | 6 | 5 | 1 | 0 | 0 | scaffold_37200 |
|  |  | 15 | 597 | 99.7 | 1 | 607 | chrX | 23216174 | 23216787 | 7 | 6 | 1 | 0 | 0 | scaffold_1814 |
| IMAGE:1850933 | AI241449 | 1 | 436 | 100 | 2 | 438 | chr16 | 23551827 | 23552263 | 1 | 0 | 1 | 0 | 0 | scaffold_37487 |
| IMAGE:1854154 | AI243766 | 1 | 337 | 99.4 | 11 | 351 | chr17 | 37043244 | 37043584 | 1 | 1 | 0 | 0 | 0 | scaffold_37479 |
| IMAGE:1866585 | AI271952 | 1 | 416 | 100 | 1 | 416 | chr20 | 33157432 | 33157847 | 1 | 1 | 0 | 0 | 0 | scaffold_37132 |
| IMAGE:193756 | H47790 | 1 | 346 | 99.1 | 1 | 359 | chr11 | 5239831 | 5240308 | 5 | 5 | 0 | 0 | 0 | scaffold_37629 |
|  |  | 2 | 344 | 98.9 | 1 | 359 | chr11 | 5234907 | 5235384 | 5 | 5 | 0 | 0 | 0 | scaffold_37629 |
| IMAGE:193756 | H48129 | 1 | 211 | 99.1 | 1 | 223 | chr11 | 5238764 | 5239872 | 2 | 2 | 0 | 0 | 0 | scaffold_37629 |
|  |  | 2 | 207 | 98.1 | 1 | 223 | chr11 | 5233846 | 5234948 | 2 | 2 | 0 | 0 | 0 | scaffold_37629 |
| IMAGE:194284 | H50639 | 1 | 389 | 96.3 | 1 | 412 | chr2 | 96401410 | 96401814 | 8 | 7 | 1 | 0 | 0 | scaffold_36851 |
| IMAGE:194284 | H50725 | 1 | 464 | 97.5 | 1 | 482 | chr2 | 96401447 | 96401924 | 9 | 8 | 1 | 0 | 0 | scaffold_36851 |
| IMAGE:1947548 | AI342040 | 1 | 369 | 93.1 | 1 | 426 | chr1 | 86932548 | 86932956 | 5 | 4 | 1 | 0 | 0 | scaffold_37483 |
|  |  | 2 | 424 | 99.8 | 1 | 426 | chr19 | 39652211 | 39652636 | 1 | 1 | 0 | 0 | 0 | scaffold_37497 |
|  |  | 3 | 405 | 97.6 | 2 | 426 | chr6 | 142365005 | 142365429 | 1 | 1 | 0 | 0 | 0 | scaffold_37467 |
| IMAGE:198873 | H82878 | 1 | 330 | 93.9 | 6 | 417 | chr1 | 77661259 | 77661959 | 11 | 10 | 1 | 0 | 0 | scaffold_37644 |
|  |  | 2 | 308 | 91.1 | 6 | 394 | chr1 | 221025483 | 221025865 | 10 | 10 | 0 | 0 | 0 | scaffold_37457 |
|  |  | 3 | 285 | 93.2 | 12 | 367 | chr15 | 41996739 | 41997092 | 10 | 9 | 1 | 0 | 0 | scaffold_27084 |
|  |  | 4 | 337 | 91 | 6 | 426 | chr22 | 39712621 | 39713030 | 11 | 10 | 1 | 0 | 0 | scaffold_37547 |
|  |  | 5 | 320 | 90.6 | 6 | 425 | chr5 | 131070354 | 131070763 | 12 | 0 | 0 | 12 | 0 |  |
|  |  | 6 | 365 | 91.4 | 9 | 455 | chr5 | 77166059 | 77166494 | 12 | 10 | 2 | 0 | 0 | scaffold_37690 |
|  |  | 7 | 319 | 91.8 | 8 | 417 | chr5 | 97751932 | 97752331 | 9 | 8 | 1 | 0 | 0 | scaffold_37626 |
|  |  | 8 | 341 | 93.1 | 11 | 453 | chr6 | 146905746 | 146906262 | 11 | 8 | 1 | 2 | 0 | scaffold_37467 |
|  |  | 9 | 405 | 94.4 | 6 | 456 | chr7 | 5311125 | 5311563 | 11 | 11 | 0 | 0 | 0 | scaffold_37557 |
|  |  | 10 | 241 | 93.1 | 9 | 325 | chr9 | 105576784 | 105577405 | 8 | 6 | 2 | 0 | 0 | scaffold_37692 |
|  |  | 11 | 308 | 93.3 | 6 | 414 | chr9 | 98874663 | 98875404 | 12 | 12 | 0 | 0 | 0 | scaffold_37692 |
|  |  | 12 | 294 | 90.5 | 6 | 391 | chrX | 45192827 | 45193206 | 11 | 11 | 0 | 0 | 0 | scaffold_37620 |
|  |  | 13 | 311 | 93.1 | 6 | 391 | chrX | 43699426 | 43700135 | 8 | 7 | 1 | 0 | 0 | scaffold_15062 |
| IMAGE:198873 | H83101 | 1 | 273 | 93 | 13 | 339 | chr2 | 132232537 | 132595441 | 5 | 0 | 0 | 3 | 2 |  |
|  |  | 2 | 254 | 90.9 | 13 | 328 | chr2 | 131627286 | 131627597 | 3 | 0 | 0 | 3 | 0 |  |
|  |  | 3 | 235 | 90.5 | 13 | 297 | chr2 | 132595120 | 132595403 | 2 | 0 | 0 | 2 | 0 |  |
|  |  | 4 | 271 | 90.3 | 5 | 327 | chr5 | 77165410 | 77165728 | 5 | 4 | 1 | 0 | 0 | scaffold_37690 |
|  |  | 5 | 344 | 98 | 4 | 368 | chr7 | 5311971 | 5312421 | 9 | 9 | 0 | 0 | 0 | scaffold_37557 |
| IMAGE:2010571 | AI360780 | 1 | 228 | 99.6 | 1 | 230 | chr1 | 222952562 | 222952791 | 1 | 0 | 1 | 0 | 0 | scaffold_37457 |
|  |  | 2 | 225 | 99.1 | 1 | 230 | chr10 | 93230404 | 93230640 | 2 | 0 | 0 | 2 | 0 |  |
| IMAGE:2012139 | AI356623 | 1 | 537 | 99.8 | 1 | 540 | chr12 | 130901434 | 130901973 | 1 | 1 | 0 | 0 | 0 | scaffold_32145 |
| IMAGE:201475 | R99896 | 1 | 388 | 98.7 | 1 | 426 | chrX | 151528040 | 151531492 | 10 | 10 | 0 | 0 | 0 | scaffold_37341 |
| IMAGE:201475 | R99897 | 1 | 279 | 99.6 | 1 | 290 | chrX | 151531110 | 151531847 | 3 | 3 | 0 | 0 | 0 | scaffold_37341 |
| IMAGE:2018364 | AI362830 | 1 | 451 | 99.8 | 1 | 453 | chr11 | 60989102 | 60989554 | 1 | 1 | 0 | 0 | 0 | scaffold_37461 |
| IMAGE:2019594 | AI391677 | 1 | 358 | 99.7 | 1 | 376 | chr5 | 3653874 | 3654252 | 3 | 3 | 0 | 0 | 0 | scaffold_34673 |
| IMAGE:2052208 | AI349033 | 1 | 218 | 99.5 | 13 | 232 | chr3 | 50273389 | 50273608 | 1 | 1 | 0 | 0 | 0 | scaffold_37645 |
| IMAGE:2096034 | AI418679 | 1 | 342 | 99.7 | 1 | 345 | chr22 | 31580678 | 31581022 | 1 | 1 | 0 | 0 | 0 | scaffold_37586 |
| IMAGE:2116229 | AI401305 | 1 | 541 | 99.3 | 3 | 560 | chr16 | 89371409 | 89373254 | 10 | 10 | 0 | 0 | 0 | scaffold_37630 |
|  |  | 2 | 525 | 97.8 | 16 | 578 | chr17 | 17487235 | 17487803 | 8 | 7 | 1 | 0 | 0 | scaffold_34420 |
|  |  | 3 | 428 | 91.1 | 3 | 560 | chr19 | 61420314 | 61420852 | 13 | 12 | 1 | 0 | 0 | scaffold_37560 |
|  |  | 4 | 436 | 93.1 | 20 | 554 | chr20 | 45413695 | 45414255 | 7 | 7 | 0 | 0 | 0 | scaffold_37443 |
| IMAGE:2164262 | AI570601 | 1 | 293 | 97.4 | 1 | 310 | chr1 | 96385659 | 96385969 | 2 | 1 | 1 | 0 | 0 | scaffold_37180 |
|  |  | 2 | 240 | 91.4 | 20 | 310 | chr1 | 191447715 | 191448407 | 2 | 0 | 2 | 0 | 0 | scaffold_37227 |
|  |  | 3 | 235 | 91.8 | 1 | 289 | chr1 | 194355043 | 194355341 | 4 | 4 | 0 | 0 | 0 | scaffold_37677 |
|  |  | 4 | 286 | 96.7 | 1 | 310 | chr10 | 113972660 | 113972988 | 4 | 4 | 0 | 0 | 0 | scaffold_32851 |
|  |  | 5 | 282 | 95.5 | 1 | 310 | chr10 | 128797220 | 128797529 | 1 | 1 | 0 | 0 | 0 | scaffold_27098 |
|  |  | 6 | 285 | 97 | 1 | 310 | chr12 | 8124895 | 8125241 | 3 | 0 | 3 | 0 | 0 | scaffold_37591 |
|  |  | 7 | 276 | 93.8 | 2 | 310 | chr12 | 19500158 | 19500462 | 2 | 2 | 0 | 0 | 0 | scaffold_37674 |
|  |  | 8 | 250 | 91.7 | 2 | 310 | chr12 | 95796629 | 95796959 | 6 | 4 | 2 | 0 | 0 | scaffold_37435 |
|  |  | 9 | 271 | 95.4 | 9 | 310 | chr12 | 42341155 | 42341494 | 4 | 4 | 0 | 0 | 0 | scaffold_37551 |
|  |  | 10 | 234 | 94.1 | 9 | 296 | chr12 | 17036247 | 17036858 | 5 | 5 | 0 | 0 | 0 | scaffold_37674 |
|  |  | 11 | 290 | 96.8 | 1 | 310 | chr13 | 93969634 | 93969943 | 1 | 0 | 1 | 0 | 0 | scaffold_37396 |
|  |  | 12 | 240 | 92.9 | 9 | 310 | chr13 | 26588018 | 26588329 | 8 | 8 | 0 | 0 | 0 | scaffold_37433 |
|  |  | 13 | 210 | 93 | 14 | 297 | chr14 | 80119260 | 80119671 | 4 | 3 | 1 | 0 | 0 | scaffold_37670 |
|  |  | 14 | 276 | 95.7 | 1 | 310 | chr15 | 50945098 | 50945408 | 2 | 1 | 1 | 0 | 0 | scaffold_37399 |
|  |  | 15 | 260 | 94.2 | 14 | 310 | chr16 | 27111066 | 27111370 | 3 | 2 | 1 | 0 | 0 | scaffold_37487 |
|  |  | 16 | 281 | 96.1 | 1 | 310 | chr17 | 17689409 | 17690021 | 5 | 5 | 0 | 0 | 0 | scaffold_34420 |
|  |  | 17 | 274 | 94.2 | 1 | 310 | chr19 | 40566781 | 40567090 | 1 | 0 | 1 | 0 | 0 | scaffold_37497 |
|  |  | 18 | 286 | 95.8 | 2 | 310 | chr2 | 106934897 | 106935203 | 2 | 1 | 1 | 0 | 0 | scaffold_31970 |
|  |  | 19 | 274 | 94.2 | 1 | 310 | chr21 | 23685172 | 23685481 | 1 | 1 | 0 | 0 | 0 | scaffold_37624 |
|  |  | 20 | 256 | 93.6 | 1 | 310 | chr3 | 185064772 | 185065086 | 6 | 6 | 0 | 0 | 0 | scaffold_37140 |
|  |  | 21 | 286 | 96.1 | 1 | 309 | chr3 | 39361990 | 39362297 | 2 | 1 | 1 | 0 | 0 | scaffold_37683 |
|  |  | 22 | 212 | 90.8 | 1 | 310 | chr3 | 139866064 | 139866387 | 4 | 3 | 1 | 0 | 0 | scaffold_36172 |
|  |  | 23 | 245 | 91.8 | 2 | 310 | chr4 | 96639670 | 96639981 | 4 | 4 | 0 | 0 | 0 | scaffold_37147 |
|  |  | 24 | 289 | 96.8 | 1 | 310 | chr4 | 106866041 | 106866351 | 2 | 2 | 0 | 0 | 0 | scaffold_36289 |
|  |  | 25 | 266 | 94.1 | 1 | 310 | chr4 | 136426810 | 136427119 | 3 | 3 | 0 | 0 | 0 | scaffold_37448 |
|  |  | 26 | 304 | 99 | 1 | 310 | chr5 | 14704492 | 14704801 | 1 | 1 | 0 | 0 | 0 | scaffold_37681 |
|  |  | 27 | 294 | 97.4 | 1 | 310 | chr5 | 43540383 | 43540692 | 1 | 0 | 1 | 0 | 0 | scaffold_37592 |
|  |  | 28 | 232 | 92.3 | 14 | 299 | chr5 | 126591375 | 126591664 | 2 | 2 | 0 | 0 | 0 | scaffold_37656 |
|  |  | 29 | 310 | 100 | 1 | 310 | chr6 | 74222838 | 74223147 | 1 | 1 | 0 | 0 | 0 | scaffold_37647 |
|  |  | 30 | 306 | 99.4 | 1 | 310 | chr7 | 22292210 | 22292519 | 1 | 1 | 0 | 0 | 0 | scaffold_37662 |
|  |  | 31 | 285 | 96.1 | 2 | 310 | chr7 | 143735728 | 143736036 | 1 | 1 | 0 | 0 | 0 | scaffold_37657 |
|  |  | 32 | 240 | 93.5 | 9 | 309 | chr7 | 87881750 | 87882579 | 7 | 3 | 2 | 2 | 0 | scaffold_37515 |
|  |  | 33 | 308 | 99.7 | 1 | 310 | chr9 | 131172290 | 131172599 | 1 | 1 | 0 | 0 | 0 | scaffold_37662 |
|  |  | 34 | 250 | 92.5 | 14 | 310 | chrX | 84186804 | 84187108 | 6 | 6 | 0 | 0 | 0 | scaffold_37388 |
|  |  | 35 | 226 | 93.8 | 18 | 310 | chrX | 118097815 | 118098422 | 5 | 5 | 0 | 0 | 0 | scaffold_7558 |
| IMAGE:2179619 | AI571881 | 1 | 543 | 97.4 | 1 | 583 | chr1 | 96385385 | 96385969 | 3 | 1 | 2 | 0 | 0 | scaffold_37180 |
|  |  | 2 | 449 | 92 | 20 | 579 | chr1 | 191447451 | 191448407 | 4 | 2 | 2 | 0 | 0 | scaffold_37227 |
|  |  | 3 | 236 | 92.2 | 1 | 289 | chr1 | 194355043 | 194355341 | 4 | 4 | 0 | 0 | 0 | scaffold_37677 |
|  |  | 4 | 480 | 96.2 | 1 | 549 | chr10 | 113972660 | 113973220 | 7 | 7 | 0 | 0 | 0 | scaffold_32851 |
|  |  | 5 | 299 | 95.4 | 1 | 330 | chr10 | 128797200 | 128797529 | 1 | 1 | 0 | 0 | 0 | scaffold_27098 |
|  |  | 6 | 534 | 93.3 | 2 | 633 | chr12 | 19500158 | 19500787 | 6 | 6 | 0 | 0 | 0 | scaffold_37674 |
|  |  | 7 | 454 | 90.8 | 2 | 575 | chr12 | 95796629 | 95797567 | 7 | 4 | 3 | 0 | 0 | scaffold_37435 |
|  |  | 8 | 324 | 97.4 | 1 | 350 | chr12 | 8124895 | 8125281 | 3 | 0 | 3 | 0 | 0 | scaffold_37591 |
|  |  | 9 | 472 | 95.4 | 9 | 537 | chr12 | 42340928 | 42341494 | 4 | 4 | 0 | 0 | 0 | scaffold_37551 |
|  |  | 10 | 467 | 93.4 | 9 | 589 | chr12 | 17035955 | 17036858 | 8 | 8 | 0 | 0 | 0 | scaffold_37674 |
|  |  | 11 | 446 | 91.9 | 9 | 570 | chr13 | 26587758 | 26588329 | 8 | 7 | 1 | 0 | 0 | scaffold_37433 |
|  |  | 12 | 346 | 97.3 | 1 | 367 | chr13 | 93969577 | 93969943 | 1 | 0 | 1 | 0 | 0 | scaffold_37396 |
|  |  | 13 | 381 | 90.6 | 14 | 526 | chr14 | 80119031 | 80119671 | 4 | 3 | 1 | 0 | 0 | scaffold_37670 |
|  |  | 14 | 504 | 94.7 | 1 | 579 | chr15 | 50945098 | 50945676 | 3 | 2 | 1 | 0 | 0 | scaffold_37399 |
|  |  | 15 | 483 | 93.8 | 14 | 575 | chr16 | 27111066 | 27111635 | 3 | 2 | 1 | 0 | 0 | scaffold_37487 |
|  |  | 16 | 341 | 96.7 | 1 | 371 | chr17 | 17689348 | 17690021 | 5 | 4 | 1 | 0 | 0 | scaffold_34420 |
|  |  | 17 | 521 | 93.5 | 1 | 615 | chr19 | 40566474 | 40567090 | 5 | 4 | 1 | 0 | 0 | scaffold_37497 |
|  |  | 18 | 569 | 95.4 | 2 | 650 | chr2 | 106934897 | 106935548 | 6 | 5 | 1 | 0 | 0 | scaffold_31970 |
|  |  | 19 | 506 | 94.1 | 1 | 583 | chr21 | 23684898 | 23685481 | 2 | 2 | 0 | 0 | 0 | scaffold_37624 |
|  |  | 20 | 514 | 92.8 | 1 | 632 | chr3 | 185064772 | 185065410 | 10 | 10 | 0 | 0 | 0 | scaffold_37140 |
|  |  | 21 | 518 | 94.9 | 1 | 585 | chr3 | 39361714 | 39362297 | 6 | 4 | 2 | 0 | 0 | scaffold_37683 |
|  |  | 22 | 429 | 91.7 | 1 | 575 | chr3 | 139865800 | 139866387 | 6 | 5 | 1 | 0 | 0 | scaffold_36172 |
|  |  | 23 | 297 | 92.6 | 2 | 376 | chr4 | 96639670 | 96640046 | 5 | 5 | 0 | 0 | 0 | scaffold_37147 |
|  |  | 24 | 599 | 96 | 1 | 679 | chr4 | 106865665 | 106866351 | 10 | 10 | 0 | 0 | 0 | scaffold_36289 |
|  |  | 25 | 484 | 93.5 | 1 | 571 | chr4 | 136426549 | 136427119 | 3 | 3 | 0 | 0 | 0 | scaffold_37448 |
|  |  | 26 | 619 | 97 | 1 | 686 | chr5 | 14704492 | 14705185 | 8 | 8 | 0 | 0 | 0 | scaffold_37681 |
|  |  | 27 | 576 | 96.9 | 1 | 637 | chr5 | 43540383 | 43541019 | 7 | 6 | 1 | 0 | 0 | scaffold_37592 |
|  |  | 28 | 345 | 91.8 | 14 | 435 | chr5 | 126591375 | 126591792 | 3 | 3 | 0 | 0 | 0 | scaffold_37656 |
|  |  | 29 | 612 | 98.4 | 1 | 652 | chr6 | 74222838 | 74223589 | 6 | 6 | 0 | 0 | 0 | scaffold_37647 |
|  |  | 30 | 605 | 97.9 | 1 | 650 | chr7 | 22292210 | 22292864 | 5 | 0 | 1 | 4 | 0 | scaffold_37334 |
|  |  | 31 | 516 | 94.2 | 2 | 595 | chr7 | 143735728 | 143736322 | 4 | 3 | 1 | 0 | 0 | scaffold_37657 |
|  |  | 32 | 454 | 92.1 | 9 | 583 | chr7 | 87881454 | 87882579 | 9 | 5 | 2 | 2 | 0 | scaffold_37515 |
|  |  | 33 | 629 | 97.7 | 1 | 686 | chr9 | 131171906 | 131172599 | 8 | 8 | 0 | 0 | 0 | scaffold_37662 |
|  |  | 34 | 479 | 92.1 | 14 | 595 | chrX | 84186804 | 84187394 | 9 | 9 | 0 | 0 | 0 | scaffold_37388 |
|  |  | 35 | 373 | 92.3 | 18 | 615 | chrX | 118097815 | 118098829 | 9 | 9 | 0 | 0 | 0 | scaffold_7558 |
| IMAGE:2284991 | AI628415 | 1 | 449 | 99.8 | 14 | 466 | chr3 | 20170401 | 20170853 | 1 | 1 | 0 | 0 | 0 | scaffold_37683 |
| IMAGE:2288232 | AI699707 | 1 | 285 | 97.9 | 17 | 313 | chr5 | 86756156 | 86756451 | 4 | 4 | 0 | 0 | 0 | scaffold_37690 |
| IMAGE:2288939 | AI862957 | 1 | 274 | 91.5 | 1 | 368 | chr4 | 162187958 | 162188619 | 7 | 7 | 0 | 0 | 0 | scaffold_37184 |
|  |  | 2 | 362 | 99.7 | 3 | 368 | chr6 | 13741752 | 13742119 | 3 | 2 | 1 | 0 | 0 | scaffold_37460 |
| IMAGE:2288939 | BX106043 | 1 | 332 | 92.3 | 9 | 440 | chr4 | 162187895 | 162188621 | 6 | 6 | 0 | 0 | 0 | scaffold_37184 |
|  |  | 2 | 427 | 99.8 | 13 | 441 | chr6 | 13741691 | 13742119 | 1 | 0 | 1 | 0 | 0 | scaffold_37460 |
| IMAGE:2300271 | AI631894 | 1 | 430 | 100 | 1 | 430 | chr20 | 62985980 | 62986409 | 1 | 0 | 1 | 0 | 0 | scaffold_37704 |
| IMAGE:2314101 | AI674580 | 1 | 443 | 99.3 | 1 | 450 | chr21 | 41756876 | 41758048 | 2 | 2 | 0 | 0 | 0 | scaffold_31899 |
| IMAGE:2343116 | AI701488 | 1 | 486 | 99.8 | 1 | 488 | chrX | 44011721 | 44012208 | 1 | 1 | 0 | 0 | 0 | scaffold_3562 |
| IMAGE:2343116 | BX108336 | 1 | 401 | 99.8 | 1 | 404 | chrX | 44011496 | 44011899 | 1 | 1 | 0 | 0 | 0 | scaffold_3562 |
| IMAGE:2346427 | AI669042 | 1 | 344 | 91.6 | 1 | 422 | chr1 | 43982010 | 43982457 | 8 | 7 | 1 | 0 | 0 | scaffold_37599 |
|  |  | 2 | 386 | 96 | 1 | 422 | chr1 | 154261116 | 154261544 | 4 | 3 | 1 | 0 | 0 | scaffold_36950 |
|  |  | 3 | 296 | 90.8 | 17 | 420 | chr10 | 8559425 | 8559821 | 5 | 5 | 0 | 0 | 0 | scaffold_37507 |
|  |  | 4 | 260 | 92.8 | 20 | 329 | chr10 | 68767718 | 68768031 | 5 | 5 | 0 | 0 | 0 | scaffold_37597 |
|  |  | 5 | 343 | 90.9 | 1 | 422 | chr11 | 4708018 | 4708445 | 5 | 3 | 2 | 0 | 0 | scaffold_37629 |
|  |  | 6 | 343 | 92 | 12 | 421 | chr11 | 119010982 | 119011389 | 6 | 5 | 1 | 0 | 0 | scaffold_37627 |
|  |  | 7 | 408 | 99.8 | 13 | 422 | chr12 | 51577242 | 51577651 | 1 | 1 | 0 | 0 | 0 | scaffold_37077 |
|  |  | 8 | 323 | 90.5 | 8 | 422 | chr14 | 37187554 | 37188011 | 4 | 4 | 0 | 0 | 0 | scaffold_32092 |
|  |  | 9 | 334 | 90.5 | 6 | 422 | chr15 | 40808979 | 40809389 | 7 | 6 | 1 | 0 | 0 | scaffold_19996 |
|  |  | 10 | 330 | 90.9 | 21 | 422 | chr15 | 68868149 | 68868539 | 7 | 5 | 2 | 0 | 0 | scaffold_37693 |
|  |  | 11 | 336 | 91.2 | 1 | 414 | chr2 | 203907340 | 203907761 | 4 | 2 | 2 | 0 | 0 | scaffold_37505 |
|  |  | 12 | 329 | 90.8 | 12 | 422 | chr2 | 184138066 | 184138483 | 6 | 5 | 1 | 0 | 0 | scaffold_37634 |
|  |  | 13 | 311 | 92.2 | 1 | 422 | chr2 | 220020409 | 220020839 | 10 | 9 | 1 | 0 | 0 | scaffold_37336 |
|  |  | 14 | 340 | 92.3 | 1 | 422 | chr2 | 178265653 | 178266357 | 5 | 4 | 1 | 0 | 0 | scaffold_37634 |
|  |  | 15 | 362 | 95.1 | 1 | 422 | chr3 | 139140975 | 139141404 | 5 | 4 | 1 | 0 | 0 | scaffold_36172 |
|  |  | 16 | 287 | 90.2 | 13 | 380 | chr4 | 104108094 | 104108448 | 5 | 0 | 1 | 4 | 0 | scaffold_20635 |
|  |  | 17 | 302 | 91.1 | 1 | 389 | chr4 | 33299350 | 33299744 | 8 | 6 | 2 | 0 | 0 | scaffold_37506 |
|  |  | 18 | 359 | 93.4 | 1 | 421 | chr5 | 122812570 | 122812973 | 6 | 5 | 1 | 0 | 0 | scaffold_37656 |
|  |  | 19 | 329 | 90.9 | 16 | 422 | chr5 | 60055029 | 60055445 | 5 | 5 | 0 | 0 | 0 | scaffold_37612 |
|  |  | 20 | 329 | 91.6 | 1 | 422 | chr5 | 146114007 | 146114446 | 8 | 7 | 1 | 0 | 0 | scaffold_37144 |
|  |  | 21 | 284 | 91.6 | 5 | 354 | chr6 | 134598444 | 134598808 | 6 | 5 | 1 | 0 | 0 | scaffold_34646 |
|  |  | 22 | 336 | 92.6 | 3 | 416 | chr7 | 38436830 | 38437252 | 5 | 3 | 2 | 0 | 0 | scaffold_33340 |
|  |  | 23 | 393 | 96.9 | 1 | 422 | chr8 | 62542019 | 62542441 | 3 | 2 | 1 | 0 | 0 | scaffold_27056 |
|  |  | 24 | 338 | 91.4 | 6 | 422 | chr9 | 97448559 | 97448980 | 8 | 7 | 1 | 0 | 0 | scaffold_37692 |
|  |  | 25 | 340 | 92.5 | 1 | 422 | chrX | 150266937 | 150267359 | 4 | 0 | 4 | 0 | 0 | scaffold_37326 |
|  |  | 26 | 313 | 91.1 | 7 | 422 | chrX | 56979186 | 56979777 | 6 | 6 | 0 | 0 | 0 | scaffold_37519 |
| IMAGE:2356687 | AI806191 | 1 | 444 | 97.2 | 1 | 479 | chr7 | 23313165 | 23313642 | 2 | 2 | 0 | 0 | 0 | scaffold_34502 |
| IMAGE:2401541 | AI767981 | 1 | 367 | 100 | 4 | 370 | chr1 | 109434492 | 109434858 | 1 | 1 | 0 | 0 | 0 | scaffold_36156 |
| IMAGE:2403105 | AI796421 | 1 | 487 | 100 | 1 | 491 | chr12 | 100012936 | 100024186 | 4 | 4 | 0 | 0 | 0 | scaffold_37435 |
| IMAGE:2406177 | AI831494 | 1 | 439 | 97.6 | 1 | 465 | chr19 | 16297144 | 16297606 | 2 | 2 | 0 | 0 | 0 | scaffold_37632 |
| IMAGE:2426006 | AI866096 | 1 | 343 | 100 | 1 | 343 | chr16 | 58214996 | 58215338 | 1 | 0 | 1 | 0 | 0 | scaffold_37666 |
| IMAGE:243456 | BX101179 | 1 | 745 | 99.7 | 1 | 751 | chr8 | 67402919 | 67403669 | 1 | 1 | 0 | 0 | 0 | scaffold_37639 |
| IMAGE:243456 | N39016 | 1 | 323 | 97.9 | 1 | 332 | chr8 | 67403380 | 67403709 | 3 | 3 | 0 | 0 | 0 | scaffold_37639 |
| IMAGE:243456 | N48211 | 1 | 484 | 97.6 | 1 | 502 | chr8 | 67402920 | 67403416 | 6 | 6 | 0 | 0 | 0 | scaffold_37639 |
| IMAGE:2447867 | AI933639 | 1 | 355 | 99.7 | 1 | 358 | chr3 | 113636545 | 113636902 | 3 | 3 | 0 | 0 | 0 | scaffold_37456 |
| IMAGE:2455273 | AI922800 | 1 | 486 | 99.6 | 1 | 496 | chr14 | 104145133 | 104145853 | 5 | 4 | 1 | 0 | 0 | scaffold_33291 |
|  |  | 2 | 422 | 97.5 | 1 | 444 | chr14 | 104024902 | 104025345 | 1 | 0 | 0 | 0 | 1 |  |
| IMAGE:252472 | H87601 | 1 | 361 | 99.2 | 14 | 383 | chr4 | 185318748 | 185325488 | 5 | 5 | 0 | 0 | 0 | scaffold_32874 |
| IMAGE:252472 | H87602 | 1 | 377 | 96.4 | 1 | 395 | chr4 | 185330224 | 185330612 | 7 | 7 | 0 | 0 | 0 | scaffold_32874 |
| IMAGE:2558598 | AW050718 | 1 | 367 | 99.5 | 1 | 371 | chr2 | 119331310 | 119331680 | 1 | 1 | 0 | 0 | 0 | scaffold_37685 |
| IMAGE:2581298 | AW087544 | 1 | 438 | 99.3 | 15 | 454 | chr12 | 123162065 | 123162503 | 2 | 2 | 0 | 0 | 0 | scaffold_37680 |
| IMAGE:2618534 | AW149331 | 1 | 295 | 99 | 14 | 314 | chr7 | 143469166 | 143469466 | 1 | 1 | 0 | 0 | 0 | scaffold_37657 |
| IMAGE:2675248 | AW189306 | 1 | 208 | 92.2 | 3 | 276 | chr3 | 152616600 | 152616875 | 4 | 3 | 1 | 0 | 0 | scaffold_37635 |
|  |  | 2 | 372 | 92 | 1 | 449 | chr4 | 54869703 | 54870149 | 6 | 6 | 0 | 0 | 0 | scaffold_37623 |
|  |  | 3 | 300 | 92.9 | 1 | 356 | chr5 | 52089839 | 52090194 | 5 | 5 | 0 | 0 | 0 | scaffold_37612 |
|  |  | 4 | 448 | 100 | 1 | 449 | chrX | 101702290 | 101702739 | 2 | 1 | 0 | 1 | 0 | scaffold_37494 |
| IMAGE:2689920 | AW237597 | 1 | 315 | 99.4 | 1 | 322 | chr10 | 128520732 | 128544860 | 4 | 4 | 0 | 0 | 0 | scaffold_27098 |
| IMAGE:2698222 | AW170435 | 1 | 270 | 99.3 | 5 | 278 | chr8 | 51196979 | 51197252 | 1 | 1 | 0 | 0 | 0 | scaffold_32035 |
| IMAGE:2698561 | AW170091 | 1 | 477 | 99.6 | 1 | 481 | chr15 | 82618298 | 82618778 | 1 | 0 | 0 | 0 | 1 |  |
|  |  | 2 | 475 | 99.4 | 1 | 481 | chr15 | 80908389 | 80908869 | 1 | 1 | 0 | 0 | 0 | scaffold_18904 |
|  |  | 3 | 475 | 99.4 | 1 | 481 | chr15 | 80513811 | 80514291 | 1 | 1 | 0 | 0 | 0 | scaffold_7487 |
|  |  | 4 | 471 | 99 | 1 | 481 | chr15 | 83507404 | 83507884 | 1 | 1 | 0 | 0 | 0 | scaffold_34393 |
|  |  | 5 | 479 | 99.8 | 1 | 481 | chr15 | 80749492 | 80749972 | 1 | 0 | 0 | 0 | 1 |  |
|  |  | 6 | 475 | 99.4 | 1 | 481 | chr15 | 80363005 | 80363485 | 1 | 0 | 0 | 0 | 1 |  |
|  |  | 7 | 473 | 99.2 | 1 | 481 | chr15 | 80670492 | 80670972 | 1 | 0 | 0 | 0 | 1 |  |
|  |  | 8 | 477 | 99.6 | 1 | 481 | chr15_random | 685742 | 686222 | 1 | 0 | 0 | 0 | 1 |  |
|  |  | 9 | 473 | 99.2 | 1 | 481 | chr15_random | 435715 | 436195 | 1 | 0 | 0 | 0 | 1 |  |
|  |  | 10 | 479 | 99.8 | 1 | 481 | chr15_random | 489630 | 490110 | 1 | 0 | 0 | 0 | 1 |  |
|  |  | 11 | 473 | 99.2 | 1 | 481 | chr15_random | 427643 | 428123 | 1 | 0 | 0 | 0 | 1 |  |
| IMAGE:2707174 | AW513634 | 1 | 473 | 99.6 | 7 | 483 | chr13 | 51423717 | 51424193 | 1 | 1 | 0 | 0 | 0 | scaffold_37562 |
| IMAGE:2711441 | AW016037 | 1 | 441 | 99.6 | 16 | 463 | chr1 | 230285471 | 230315880 | 5 | 4 | 1 | 0 | 0 | scaffold_36933 |
| IMAGE:273148 | N36781 | 1 | 605 | 98.2 | 1 | 643 | chr18 | 20284837 | 20285479 | 7 | 7 | 0 | 0 | 0 | scaffold_37707 |
| IMAGE:273148 | N45942 | 1 | 321 | 100 | 3 | 324 | chr18 | 20284702 | 20285023 | 1 | 0 | 1 | 0 | 0 | scaffold_37707 |
| IMAGE:2754020 | AW265287 | 1 | 400 | 100 | 1 | 401 | chr12 | 13260583 | 13261003 | 2 | 1 | 1 | 0 | 0 | scaffold_37674 |
| IMAGE:2760019 | AW276438 | 1 | 393 | 98.8 | 19 | 421 | chr3 | 50094522 | 50094924 | 1 | 1 | 0 | 0 | 0 | scaffold_1893 |
| IMAGE:2783514 | AW162899 | 1 | 465 | 100 | 1 | 469 | chr4 | 5927295 | 5954691 | 3 | 2 | 0 | 1 | 0 | scaffold_32170 |
| IMAGE:2817753 | AW470907 | 1 | 325 | 99.4 | 135 | 467 | chr2 | 39453511 | 39462330 | 5 | 5 | 0 | 0 | 0 | scaffold_37688 |
| IMAGE:2822399 | AW246933 | 1 | 398 | 99.8 | 10 | 421 | chr20 | 2439508 | 2446436 | 5 | 5 | 0 | 0 | 0 | scaffold_36962 |
| IMAGE:2822399 | AW250796 | 1 | 559 | 100 | 10 | 571 | chr20 | 2437291 | 2438875 | 3 | 3 | 0 | 0 | 0 | scaffold_36962 |
| IMAGE:2956355 | AW612098 | 1 | 322 | 100 | 1 | 322 | chr22 | 29052554 | 29052875 | 1 | 1 | 0 | 0 | 0 | scaffold_37586 |
| IMAGE:2960310 | BE299665 | 1 | 532 | 99.6 | 33 | 571 | chrX | 46091444 | 46092154 | 5 | 5 | 0 | 0 | 0 | scaffold_36155 |
| IMAGE:2960310 | BE300307 | 1 | 561 | 99.6 | 44 | 613 | chrX | 46091448 | 46092192 | 7 | 7 | 0 | 0 | 0 | scaffold_36155 |
| IMAGE:299751 | W05803 | 1 | 554 | 98.4 | 1 | 587 | chr8 | 22412371 | 22420553 | 11 | 10 | 1 | 0 | 0 | scaffold_37701 |
| IMAGE:3032949 | AW772103 | 1 | 491 | 100 | 1 | 491 | chr17 | 57663639 | 57664129 | 1 | 0 | 1 | 0 | 0 | scaffold_37659 |
|  |  | 2 | 463 | 96.4 | 1 | 500 | chr9 | 120900478 | 120900979 | 2 | 0 | 0 | 2 | 0 |  |
| IMAGE:30332828 | CB993859 | 1 | 585 | 90.5 | 69 | 794 | chr1 | 151603741 | 151604455 | 11 | 11 | 0 | 0 | 0 | scaffold_36950 |
|  |  | 2 | 712 | 99 | 28 | 760 | chr19 | 43557268 | 43564707 | 11 | 9 | 0 | 2 | 0 | scaffold_37497 |
| IMAGE:30341153 | CB989555 | 1 | 523 | 99.4 | 28 | 562 | chr11 | 65202957 | 65205775 | 7 | 6 | 1 | 0 | 0 | scaffold_37417 |
| IMAGE:30348971 | CB961988 | 1 | 718 | 99.3 | 28 | 761 | chr19 | 45976046 | 45984723 | 9 | 5 | 0 | 4 | 0 | scaffold_37165 |
| IMAGE:30351998 | CB956007 | 1 | 283 | 100 | 3 | 286 | chr4 | 71182219 | 71183708 | 2 | 2 | 0 | 0 | 0 | scaffold_37623 |
| IMAGE:30366289 | CD108265 | 1 | 790 | 98.3 | 1 | 831 | chr17 | 62467930 | 62469561 | 16 | 12 | 4 | 0 | 0 | scaffold_37678 |
|  |  | 2 | 706 | 93.9 | 1 | 831 | chr17 | 62430942 | 62432586 | 15 | 3 | 1 | 11 | 0 | scaffold_37678 |
|  |  | 3 | 678 | 92.4 | 1 | 820 | chr17 | 62422754 | 62447296 | 15 | 4 | 2 | 9 | 0 | scaffold_37678 |
|  |  | 4 | 606 | 92.6 | 1 | 717 | chr17 | 62422850 | 62424399 | 6 | 0 | 0 | 6 | 0 |  |
| IMAGE:30371344 | CD103346 | 1 | 666 | 97.3 | 3 | 694 | chr5 | 64030181 | 64030858 | 11 | 10 | 1 | 0 | 0 | scaffold_33689 |
| IMAGE:30377669 | CD244628 | 1 | 503 | 99 | 27 | 554 | chr12 | 109182199 | 109197203 | 10 | 9 | 1 | 0 | 0 | scaffold_37306 |
| IMAGE:30384985 | CD242644 | 1 | 757 | 97.1 | 27 | 817 | chr18 | 12296997 | 12297779 | 10 | 8 | 2 | 0 | 0 | scaffold_28456 |
| IMAGE:30404579 | CD356197 | 1 | 558 | 98.8 | 3 | 577 | chr19 | 54160378 | 54161473 | 5 | 5 | 0 | 0 | 0 | scaffold_37466 |
|  |  | 2 | 551 | 95.1 | 2 | 610 | chr20 | 3999364 | 3999966 | 6 | 6 | 0 | 0 | 0 | scaffold_37176 |
|  |  | 3 | 522 | 93.5 | 8 | 610 | chrX | 30009645 | 30010243 | 10 | 9 | 1 | 0 | 0 | scaffold_32663 |
| IMAGE:30413669 | CD521907 | 1 | 551 | 97.6 | 4 | 593 | chr11 | 5211051 | 5212610 | 10 | 10 | 0 | 0 | 0 | scaffold_37629 |
|  |  | 2 | 275 | 94.5 | 150 | 482 | chr11 | 5218519 | 5219748 | 2 | 2 | 0 | 0 | 0 | scaffold_37629 |
| IMAGE:30420399 | CD657572 | 1 | 763 | 96.6 | 17 | 859 | chr19 | 977644 | 988798 | 16 | 12 | 4 | 0 | 0 | scaffold_37281 scaffold_37643 scaffold_11230 |
|  |  | 2 | 559 | 91.8 | 30 | 699 | chr6 | 111225012 | 111225682 | 2 | 1 | 1 | 0 | 0 | scaffold_37579 |
| IMAGE:30425053 | CD654820 | 1 | 757 | 99.2 | 12 | 791 | chr15 | 47142301 | 47163817 | 10 | 10 | 0 | 0 | 0 | scaffold_37412 scaffold_23708 |
| IMAGE:30425415 | CD644164 | 1 | 609 | 94.1 | 1 | 712 | chr12 | 67154042 | 67154758 | 5 | 5 | 0 | 0 | 0 | scaffold_37580 |
|  |  | 2 | 681 | 99.7 | 1 | 712 | chr14 | 51167856 | 51177941 | 12 | 12 | 0 | 0 | 0 | scaffold_32092 |
|  |  | 3 | 519 | 92 | 1 | 656 | chr5 | 34016725 | 34017980 | 9 | 8 | 1 | 0 | 0 | scaffold_37626 |
|  |  | 4 | 689 | 98.6 | 1 | 734 | chr8 | 57012726 | 57013470 | 9 | 6 | 1 | 2 | 0 | scaffold_36547 |
| IMAGE:30523865 | CF780747 | 1 | 645 | 99.8 | 7 | 657 | chr21 | 46530508 | 46537004 | 5 | 5 | 0 | 0 | 0 | scaffold_33342 |
| IMAGE:30527609 | CF995219 | 1 | 405 | 100 | 28 | 432 | chr19 | 50886635 | 50887039 | 1 | 0 | 1 | 0 | 0 | scaffold_37439 |
| IMAGE:30528536 | CF994917 | 1 | 763 | 99.6 | 28 | 801 | chrX | 101703372 | 101713484 | 6 | 6 | 0 | 0 | 0 | scaffold_37494 |
| IMAGE:30563382 | CF126905 | 1 | 786 | 99.5 | 1 | 812 | chr1 | 109529186 | 109534772 | 18 | 17 | 1 | 0 | 0 | scaffold_37402 |
|  |  | 2 | 666 | 95 | 4 | 812 | chr1 | 109497705 | 109516313 | 18 | 18 | 0 | 0 | 0 | scaffold_36156 scaffold_37402 |
|  |  | 3 | 479 | 93 | 4 | 605 | chr1 | 109509431 | 109556590 | 7 | 5 | 2 | 0 | 0 | scaffold_36156 scaffold_37402 |
|  |  | 4 | 316 | 97.1 | 6 | 346 | chr1 | 109553630 | 109555059 | 6 | 6 | 0 | 0 | 0 | scaffold_37402 |
|  |  | 5 | 512 | 90.5 | 10 | 641 | chr3 | 12274350 | 12274981 | 1 | 0 | 1 | 0 | 0 | scaffold_32934 |
| IMAGE:30572328 | CF122825 | 1 | 583 | 97.9 | 8 | 615 | chr15 | 32498067 | 32498673 | 3 | 1 | 1 | 1 | 0 | scaffold_36674 |
|  |  | 2 | 593 | 100 | 39 | 636 | chr19 | 44022939 | 44028573 | 5 | 5 | 0 | 0 | 0 | scaffold_37497 |
|  |  | 3 | 543 | 94.5 | 9 | 615 | chr6 | 7427138 | 7427741 | 4 | 4 | 0 | 0 | 0 | scaffold_37460 |
| IMAGE:3069048 | BF512470 | 1 | 418 | 100 | 1 | 421 | chr16 | 88266957 | 88327857 | 4 | 4 | 0 | 0 | 0 | scaffold_25725 scaffold_30581 |
| IMAGE:3071427 | BF507391 | 1 | 547 | 99.6 | 8 | 558 | chr18 | 3000119 | 3000669 | 1 | 1 | 0 | 0 | 0 | scaffold_37707 |
| IMAGE:30719616 | CK003892 | 1 | 621 | 99.5 | 3 | 630 | chrX | 69257112 | 69257739 | 3 | 2 | 1 | 0 | 0 | scaffold_37530 |
| IMAGE:307309 | W21235 | 1 | 255 | 99.2 | 15 | 279 | chr16 | 67200309 | 67200689 | 4 | 0 | 0 | 4 | 0 |  |
| IMAGE:309916 | N94495 | 1 | 437 | 99.8 | 10 | 449 | chr1 | 5954639 | 5955078 | 1 | 1 | 0 | 0 | 0 | scaffold_31944 |
|  |  | 2 | 253 | 92.3 | 88 | 449 | chr11 | 32681357 | 32681738 | 9 | 2 | 0 | 7 | 0 | scaffold_11461 |
|  |  | 3 | 359 | 93 | 1 | 449 | chr2 | 110229367 | 110229819 | 9 | 8 | 1 | 0 | 0 | scaffold_33226 |
|  |  | 4 | 357 | 92.8 | 1 | 445 | chr2 | 106487084 | 106487541 | 10 | 0 | 0 | 10 | 0 |  |
|  |  | 5 | 359 | 93 | 1 | 449 | chr2 | 111206990 | 111207442 | 9 | 0 | 0 | 0 | 9 |  |
|  |  | 6 | 345 | 92.4 | 1 | 445 | chr2 | 108154627 | 108155084 | 9 | 0 | 0 | 9 | 0 |  |
| IMAGE:309916 | W23945 | 1 | 430 | 98.4 | 1 | 439 | chr1 | 5955481 | 5955917 | 3 | 3 | 0 | 0 | 0 | scaffold_31944 |
|  |  | 2 | 328 | 94.1 | 2 | 382 | chr2 | 106485975 | 106486659 | 8 | 2 | 0 | 6 | 0 | scaffold_37331 |
|  |  | 3 | 321 | 93.5 | 2 | 382 | chr2 | 108153527 | 108154202 | 7 | 0 | 0 | 7 | 0 |  |
|  |  | 4 | 312 | 92.6 | 2 | 382 | chr2 | 111206171 | 111206567 | 8 | 0 | 0 | 0 | 8 |  |
|  |  | 5 | 312 | 92.6 | 2 | 382 | chr2 | 110230242 | 110230638 | 8 | 7 | 1 | 0 | 0 | scaffold_33226 |
|  |  | 6 | 218 | 91.7 | 9 | 296 | chrX | 80517087 | 80517386 | 5 | 4 | 1 | 0 | 0 | scaffold_33279 |
| IMAGE:3110624 | BM988524 | 1 | 659 | 95.5 | 14 | 730 | chr10 | 94093345 | 94094058 | 2 | 1 | 1 | 0 | 0 | scaffold_37705 |
|  |  | 2 | 594 | 94 | 35 | 730 | chr14 | 62191439 | 62192420 | 9 | 6 | 3 | 0 | 0 | scaffold_32092 |
|  |  | 3 | 690 | 98 | 14 | 730 | chr2 | 171810878 | 171811576 | 2 | 0 | 0 | 2 | 0 |  |
|  |  | 4 | 712 | 100 | 14 | 730 | chr20 | 33397161 | 33409023 | 6 | 6 | 0 | 0 | 0 | scaffold_37132 |
|  |  | 5 | 622 | 93.8 | 14 | 730 | chr3 | 186284421 | 186285123 | 7 | 6 | 1 | 0 | 0 | scaffold_7792 |
| IMAGE:3162538 | BE313990 | 1 | 543 | 91.4 | 1 | 696 | chr1 | 223600914 | 223601606 | 10 | 10 | 0 | 0 | 0 | scaffold_37457 |
|  |  | 2 | 529 | 91.8 | 1 | 660 | chr1 | 119015659 | 119016274 | 10 | 0 | 1 | 9 | 0 | scaffold_37569 |
|  |  | 3 | 641 | 97.8 | 1 | 696 | chr10 | 85984797 | 85985520 | 12 | 12 | 0 | 0 | 0 | scaffold_34649 |
|  |  | 4 | 591 | 93.3 | 1 | 696 | chr10 | 97018957 | 97019647 | 12 | 10 | 2 | 0 | 0 | scaffold_37705 |
|  |  | 5 | 523 | 90.9 | 2 | 653 | chr12 | 8403032 | 8403675 | 10 | 7 | 3 | 0 | 0 | scaffold_20622 |
|  |  | 6 | 592 | 93.5 | 1 | 696 | chr13 | 24949044 | 24949739 | 13 | 12 | 1 | 0 | 0 | scaffold_25860 |
|  |  | 7 | 665 | 98.4 | 1 | 696 | chr15 | 57776628 | 57777325 | 11 | 6 | 2 | 3 | 0 | scaffold_37184 |
|  |  | 8 | 636 | 96.4 | 2 | 696 | chr15 | 41124029 | 41124725 | 11 | 10 | 1 | 0 | 0 | scaffold_37412 |
|  |  | 9 | 529 | 90.6 | 2 | 696 | chr16 | 5320490 | 5321172 | 12 | 11 | 1 | 0 | 0 | scaffold_37151 |
|  |  | 10 | 626 | 95.2 | 1 | 696 | chr18 | 55965798 | 55966490 | 13 | 13 | 0 | 0 | 0 | scaffold_37621 |
|  |  | 11 | 547 | 91.7 | 22 | 696 | chr2 | 160748346 | 160749334 | 14 | 14 | 0 | 0 | 0 | scaffold_37694 |
|  |  | 12 | 556 | 92.1 | 1 | 696 | chr22 | 25142664 | 25143692 | 12 | 11 | 1 | 0 | 0 | scaffold_37586 |
|  |  | 13 | 516 | 90.2 | 2 | 642 | chr3 | 15157913 | 15158543 | 7 | 6 | 1 | 0 | 0 | scaffold_28429 scaffold_37683 |
|  |  | 14 | 519 | 90.1 | 2 | 653 | chr3 | 126834880 | 126835523 | 8 | 0 | 0 | 0 | 8 |  |
|  |  | 15 | 522 | 91.2 | 2 | 653 | chr3 | 75512316 | 75512959 | 7 | 5 | 2 | 0 | 0 | scaffold_20493 |
|  |  | 16 | 668 | 99 | 1 | 696 | chr4 | 152598453 | 152603296 | 14 | 13 | 1 | 0 | 0 | scaffold_37633 |
|  |  | 17 | 516 | 91 | 2 | 653 | chr4 | 9315696 | 9316338 | 11 | 10 | 1 | 0 | 0 | scaffold_10445 |
|  |  | 18 | 550 | 91.5 | 2 | 696 | chr4 | 154050132 | 154051131 | 14 | 14 | 0 | 0 | 0 | scaffold_37633 |
|  |  | 19 | 495 | 90.6 | 19 | 642 | chr4 | 4143108 | 4143720 | 10 | 0 | 0 | 0 | 10 |  |
|  |  | 20 | 644 | 97.1 | 2 | 695 | chr5 | 110604217 | 110604934 | 12 | 12 | 0 | 0 | 0 | scaffold_37656 |
|  |  | 21 | 632 | 95.6 | 1 | 696 | chr5 | 78664104 | 78664793 | 12 | 12 | 0 | 0 | 0 | scaffold_31458 |
|  |  | 22 | 505 | 91.5 | 43 | 696 | chr5 | 78088700 | 78089653 | 17 | 14 | 3 | 0 | 0 | scaffold_37690 |
|  |  | 23 | 224 | 90.7 | 41 | 304 | chr5 | 157012599 | 157012856 | 3 | 3 | 0 | 0 | 0 | scaffold_37615 |
|  |  | 24 | 658 | 97.7 | 1 | 696 | chr7 | 97626579 | 97627272 | 12 | 12 | 0 | 0 | 0 | scaffold_37686 |
|  |  | 25 | 616 | 95.7 | 9 | 696 | chr7 | 93936442 | 93937129 | 13 | 11 | 2 | 0 | 0 | scaffold_37515 |
|  |  | 26 | 517 | 91 | 2 | 642 | chr7 | 97139177 | 97139807 | 8 | 6 | 1 | 0 | 1 | scaffold_13730 |
|  |  | 27 | 512 | 90 | 2 | 642 | chr8 | 7990900 | 7991530 | 7 | 0 | 0 | 0 | 7 |  |
|  |  | 28 | 511 | 90.3 | 2 | 642 | chr8 | 6967826 | 6968456 | 10 | 0 | 0 | 0 | 10 |  |
|  |  | 29 | 512 | 90.3 | 2 | 653 | chr8 | 12496466 | 12497109 | 7 | 0 | 0 | 0 | 7 |  |
|  |  | 30 | 513 | 90.8 | 2 | 653 | chr8 | 12438340 | 12438983 | 11 | 11 | 0 | 0 | 0 | scaffold_18142 |
| IMAGE:3270673 | CB048398 | 1 | 292 | 99.3 | 30 | 326 | chr22 | 48838151 | 48838537 | 2 | 2 | 0 | 0 | 0 | scaffold_9936 |
| IMAGE:3270673 | CB048399 | 1 | 296 | 100 | 10 | 306 | chr22 | 48838151 | 48838537 | 2 | 2 | 0 | 0 | 0 | scaffold_9936 |
| IMAGE:3291161 | CB054059 | 1 | 613 | 99.7 | 24 | 646 | chr5 | 137568792 | 137571992 | 5 | 4 | 1 | 0 | 0 | scaffold_37656 |
| IMAGE:3291161 | CB054060 | 1 | 541 | 100 | 10 | 552 | chr5 | 137576927 | 137578130 | 2 | 1 | 1 | 0 | 0 | scaffold_37656 |
| IMAGE:3345261 | BE268682 | 1 | 493 | 99.8 | 25 | 524 | chr19 | 54830707 | 54835072 | 6 | 6 | 0 | 0 | 0 | scaffold_37466 |
| IMAGE:3357734 | BE253347 | 1 | 561 | 99.8 | 1 | 568 | chr11 | 44892262 | 44905798 | 6 | 6 | 0 | 0 | 0 | scaffold_37695 |
| IMAGE:33917 | R23526 | 1 | 389 | 95 | 1 | 426 | chr10 | 23429840 | 23430262 | 6 | 5 | 1 | 0 | 0 | scaffold_37564 |
|  |  | 2 | 385 | 94.7 | 1 | 426 | chr10 | 107110648 | 107111070 | 6 | 6 | 0 | 0 | 0 | scaffold_32851 |
|  |  | 3 | 344 | 91.9 | 2 | 421 | chr15 | 43061016 | 43061408 | 7 | 6 | 1 | 0 | 0 | scaffold_37412 |
|  |  | 4 | 380 | 94.9 | 1 | 418 | chr2 | 127408616 | 127409030 | 6 | 5 | 1 | 0 | 0 | scaffold_37685 |
|  |  | 5 | 351 | 92.9 | 5 | 417 | chr6 | 127657456 | 127657863 | 8 | 8 | 0 | 0 | 0 | scaffold_37501 |
|  |  | 6 | 397 | 97.6 | 6 | 426 | chr8 | 101893741 | 101917714 | 7 | 7 | 0 | 0 | 0 | scaffold_37544 |
|  |  | 7 | 362 | 92.7 | 8 | 426 | chr9 | 34912515 | 34912930 | 8 | 7 | 1 | 0 | 0 | scaffold_37419 |
|  |  | 8 | 383 | 95.1 | 8 | 426 | chrX | 40566282 | 40566697 | 6 | 6 | 0 | 0 | 0 | scaffold_37163 |
|  |  | 9 | 292 | 91 | 39 | 420 | chrX | 62699588 | 62699964 | 9 | 8 | 1 | 0 | 0 | scaffold_37513 |
| IMAGE:345774 | W78179 | 1 | 408 | 97.4 | 1 | 425 | chr12 | 116994791 | 116995211 | 4 | 3 | 1 | 0 | 0 | scaffold_37378 |
|  |  | 2 | 377 | 93.4 | 1 | 425 | chr2 | 85542298 | 85542708 | 7 | 5 | 2 | 0 | 0 | scaffold_36190 |
| IMAGE:3503912 | BE281089 | 1 | 670 | 99.7 | 32 | 714 | chr1 | 158388966 | 158396495 | 11 | 11 | 0 | 0 | 0 | scaffold_33696 scaffold_30731 |
| IMAGE:3531003 | BE297709 | 1 | 666 | 99.1 | 5 | 710 | chr17 | 5044223 | 5046320 | 9 | 9 | 0 | 0 | 0 | scaffold_37555 |
| IMAGE:3542171 | BE269008 | 1 | 494 | 97.8 | 1 | 528 | chr16 | 11405778 | 11411171 | 13 | 6 | 0 | 7 | 0 | scaffold_34722 |
| IMAGE:3563735 | BF196788 | 1 | 507 | 99.8 | 1 | 513 | chr9 | 134337710 | 134338690 | 5 | 1 | 4 | 0 | 0 | scaffold_36948 |
| IMAGE:363827 | AA021020 | 1 | 434 | 99.3 | 1 | 436 | chr20 | 4828123 | 4828557 | 2 | 2 | 0 | 0 | 0 | scaffold_11682 |
| IMAGE:3677388 | BE561430 | 1 | 747 | 98 | 1 | 771 | chr6 | 52316878 | 52317646 | 3 | 2 | 1 | 0 | 0 | scaffold_37490 |
| IMAGE:37961 | R60588 | 1 | 370 | 98.7 | 7 | 383 | chr7 | 115755361 | 115755736 | 2 | 2 | 0 | 0 | 0 | scaffold_37671 |
| IMAGE:3835278 | BE747476 | 1 | 602 | 98.1 | 53 | 704 | chr19 | 59396554 | 59403265 | 19 | 19 | 0 | 0 | 0 | scaffold_37207 |
|  |  | 2 | 524 | 95.1 | 69 | 655 | chr19 | 54914589 | 54915175 | 12 | 11 | 1 | 0 | 0 | scaffold_36551 |
|  |  | 3 | 547 | 94.4 | 62 | 666 | chr22 | 39703064 | 39703659 | 13 | 13 | 0 | 0 | 0 | scaffold_37547 |
| IMAGE:3836704 | BE742175 | 1 | 432 | 99.8 | 1 | 438 | chr17 | 62381921 | 62382750 | 5 | 5 | 0 | 0 | 0 | scaffold_37678 |
| IMAGE:3899766 | BE907705 | 1 | 591 | 99.7 | 19 | 619 | chr17 | 9482460 | 9672354 | 7 | 6 | 1 | 0 | 0 | scaffold_25922 scaffold_37659 |
| IMAGE:3902925 | BE910610 | 1 | 656 | 99.1 | 1 | 675 | chr2 | 220606658 | 220607338 | 8 | 8 | 0 | 0 | 0 | scaffold_37336 |
| IMAGE:3923335 | BE897768 | 1 | 672 | 99.7 | 1 | 681 | chr8 | 75312626 | 75326264 | 7 | 7 | 0 | 0 | 0 | scaffold_37639 |
| IMAGE:3925426 | BE898119 | 1 | 678 | 99.4 | 16 | 705 | chr17 | 80505517 | 80506641 | 9 | 9 | 0 | 0 | 0 | scaffold_34348 |
|  |  | 2 | 671 | 99.4 | 16 | 703 | chr17_random | 2226816 | 2227948 | 13 | 0 | 0 | 0 | 13 |  |
| IMAGE:3949097 | BE740209 | 1 | 256 | 97.3 | 16 | 279 | chr17 | 42905374 | 42905634 | 4 | 3 | 1 | 0 | 0 | scaffold_37116 |
|  |  | 2 | 235 | 95.8 | 568 | 837 | chr17 | 27187415 | 27188089 | 13 | 13 | 0 | 0 | 0 | scaffold_28792 |
| IMAGE:4041177 | BF129988 | 1 | 524 | 92.4 | 37 | 669 | chr1 | 148092938 | 148093576 | 13 | 11 | 2 | 0 | 0 | scaffold_36950 |
|  |  | 2 | 534 | 92.4 | 2 | 669 | chr11 | 31266217 | 31266888 | 15 | 15 | 0 | 0 | 0 | scaffold_11461 |
|  |  | 3 | 428 | 90.9 | 40 | 578 | chr11 | 108230495 | 108231039 | 8 | 8 | 0 | 0 | 0 | scaffold_37627 |
|  |  | 4 | 500 | 91.7 | 25 | 670 | chr13 | 39235413 | 39236070 | 19 | 17 | 2 | 0 | 0 | scaffold_36177 |
|  |  | 5 | 511 | 91.5 | 19 | 667 | chr14 | 75090064 | 75090712 | 14 | 14 | 0 | 0 | 0 | scaffold_37670 |
|  |  | 6 | 200 | 93.9 | 380 | 675 | chr14 | 34351764 | 34352065 | 8 | 8 | 0 | 0 | 0 | scaffold_32092 |
|  |  | 7 | 519 | 92.9 | 37 | 669 | chr16 | 25431904 | 25432535 | 13 | 1 | 1 | 11 | 0 | scaffold_34603 |
|  |  | 8 | 512 | 92.3 | 37 | 669 | chr2 | 78614140 | 78614773 | 14 | 14 | 0 | 0 | 0 | scaffold_36190 |
|  |  | 9 | 531 | 91.9 | 13 | 669 | chr3 | 10074352 | 10075017 | 14 | 11 | 3 | 0 | 0 | scaffold_32934 |
|  |  | 10 | 502 | 90.1 | 28 | 669 | chr3 | 10017029 | 10017656 | 16 | 2 | 2 | 12 | 0 | scaffold_4376 |
|  |  | 11 | 440 | 91.4 | 27 | 578 | chr3 | 11895083 | 11895620 | 13 | 12 | 1 | 0 | 0 | scaffold_32934 |
|  |  | 12 | 233 | 91.5 | 277 | 578 | chr3 | 33203276 | 33203582 | 7 | 6 | 1 | 0 | 0 | scaffold_37683 |
|  |  | 13 | 609 | 96.2 | 1 | 669 | chr6 | 34234021 | 34235011 | 14 | 14 | 0 | 0 | 0 | scaffold_37673 |
|  |  | 14 | 495 | 91.4 | 37 | 666 | chr7 | 120592994 | 120593637 | 15 | 15 | 0 | 0 | 0 | scaffold_37671 |
|  |  | 15 | 644 | 98.9 | 1 | 669 | chr7 | 24905285 | 24907134 | 14 | 14 | 0 | 0 | 0 | scaffold_34502 |
|  |  | 16 | 587 | 96.2 | 27 | 670 | chr8 | 33884439 | 33885090 | 12 | 12 | 0 | 0 | 0 | scaffold_37182 |
|  |  | 17 | 529 | 91.3 | 1 | 669 | chr9 | 92141671 | 92142341 | 14 | 14 | 0 | 0 | 0 | scaffold_37575 |
|  |  | 18 | 583 | 94.3 | 2 | 669 | chrX | 151574559 | 151575234 | 12 | 12 | 0 | 0 | 0 | scaffold_22502 |
|  |  | 19 | 430 | 94.5 | 169 | 669 | chrY | 15998315 | 15998818 | 13 | 13 | 0 | 0 | 0 | scaffold_34733 |
| IMAGE:4042664 | BF106086 | 1 | 368 | 97.1 | 1 | 420 | chr3 | 36988889 | 36989305 | 6 | 5 | 1 | 0 | 0 | scaffold_9043 |
| IMAGE:4073246 | BF102949 | 1 | 531 | 98.5 | 1 | 554 | chr19 | 54160397 | 54161472 | 14 | 14 | 0 | 0 | 0 | scaffold_37466 |
|  |  | 2 | 519 | 95.9 | 1 | 588 | chr20 | 3999384 | 3999972 | 16 | 16 | 0 | 0 | 0 | scaffold_37176 |
|  |  | 3 | 494 | 94 | 1 | 588 | chrX | 30009659 | 30010249 | 18 | 17 | 1 | 0 | 0 | scaffold_32663 |
| IMAGE:4274977 | BF664617 | 1 | 578 | 99.5 | 1 | 595 | chrX | 104800189 | 104810879 | 14 | 10 | 0 | 4 | 0 | scaffold_1932 |
| IMAGE:427693 | AA002166 | 1 | 439 | 97.8 | 5 | 464 | chr2 | 219889335 | 219889788 | 5 | 5 | 0 | 0 | 0 | scaffold_37336 |
| IMAGE:427693 | BX106920 | 1 | 646 | 100 | 1 | 648 | chr2 | 219889141 | 219889788 | 1 | 1 | 0 | 0 | 0 | scaffold_37336 |
| IMAGE:4281569 | BF696660 | 1 | 570 | 94.7 | 3 | 643 | chr1 | 27138014 | 27138643 | 8 | 7 | 1 | 0 | 0 | scaffold_37489 |
|  |  | 2 | 413 | 91.3 | 119 | 639 | chr1 | 200513126 | 200513632 | 8 | 7 | 1 | 0 | 0 | scaffold_37677 |
|  |  | 3 | 480 | 93.6 | 49 | 600 | chr10 | 97984481 | 97985030 | 6 | 6 | 0 | 0 | 0 | scaffold_37705 |
|  |  | 4 | 470 | 93 | 49 | 597 | chr10 | 126130432 | 126130983 | 7 | 7 | 0 | 0 | 0 | scaffold_34590 |
|  |  | 5 | 514 | 93.2 | 49 | 643 | chr10 | 97614029 | 97614610 | 9 | 8 | 1 | 0 | 0 | scaffold_37705 |
|  |  | 6 | 486 | 94.2 | 49 | 600 | chr10 | 74022391 | 74022943 | 6 | 6 | 0 | 0 | 0 | scaffold_34674 |
|  |  | 7 | 498 | 94.3 | 49 | 639 | chr11 | 61873732 | 61874324 | 12 | 11 | 1 | 0 | 0 | scaffold_36755 |
|  |  | 8 | 538 | 93.1 | 3 | 632 | chr12 | 9739152 | 9739755 | 8 | 7 | 1 | 0 | 0 | scaffold_37674 |
|  |  | 9 | 480 | 91.1 | 49 | 654 | chr13 | 66204336 | 66204927 | 7 | 6 | 1 | 0 | 0 | scaffold_30014 |
|  |  | 10 | 513 | 93.1 | 7 | 625 | chr15 | 90558735 | 90559345 | 8 | 7 | 1 | 0 | 0 | scaffold_34672 |
|  |  | 11 | 484 | 90.6 | 3 | 643 | chr15 | 70907886 | 70908516 | 7 | 7 | 0 | 0 | 0 | scaffold_37637 |
|  |  | 12 | 494 | 91.5 | 3 | 641 | chr16 | 5415995 | 5416634 | 12 | 12 | 0 | 0 | 0 | scaffold_36026 |
|  |  | 13 | 398 | 91.8 | 32 | 539 | chr18 | 22036754 | 22037345 | 9 | 7 | 2 | 0 | 0 | scaffold_3084 scaffold_37707 |
|  |  | 14 | 555 | 94.5 | 3 | 660 | chr2 | 198447282 | 198448254 | 12 | 7 | 4 | 1 | 0 | scaffold_37634 |
|  |  | 15 | 480 | 93.6 | 76 | 643 | chr2 | 204840223 | 204840789 | 7 | 5 | 2 | 0 | 0 | scaffold_37505 |
|  |  | 16 | 459 | 90.3 | 49 | 643 | chr2 | 4126632 | 4127204 | 8 | 7 | 1 | 0 | 0 | scaffold_37131 |
|  |  | 17 | 344 | 91.3 | 3 | 600 | chr2 | 122561460 | 122561871 | 6 | 4 | 1 | 1 | 0 | scaffold_37685 |
|  |  | 18 | 495 | 90.4 | 6 | 643 | chr20 | 38291984 | 38292586 | 7 | 7 | 0 | 0 | 0 | scaffold_37443 |
|  |  | 19 | 513 | 91.5 | 3 | 654 | chr3 | 150977938 | 150978588 | 11 | 11 | 0 | 0 | 0 | scaffold_37635 |
|  |  | 20 | 596 | 99.3 | 3 | 625 | chr5 | 170795780 | 170800901 | 11 | 9 | 0 | 2 | 0 | scaffold_37615 |
|  |  | 21 | 588 | 95.8 | 2 | 641 | chr5 | 93092962 | 93093592 | 8 | 4 | 4 | 0 | 0 | scaffold_37517 |
|  |  | 22 | 479 | 92.2 | 49 | 625 | chr6 | 41681451 | 41682025 | 7 | 6 | 1 | 0 | 0 | scaffold_37583 |
|  |  | 23 | 461 | 92.3 | 49 | 598 | chr6 | 70411149 | 70411698 | 5 | 4 | 1 | 0 | 0 | scaffold_7679 |
|  |  | 24 | 491 | 92.3 | 1 | 605 | chr7 | 36599614 | 36600221 | 8 | 8 | 0 | 0 | 0 | scaffold_37393 |
|  |  | 25 | 475 | 93.8 | 49 | 600 | chr7 | 148422797 | 148423355 | 7 | 6 | 1 | 0 | 0 | scaffold_33336 |
|  |  | 26 | 555 | 93.8 | 3 | 641 | chr8 | 62164776 | 62165400 | 8 | 6 | 1 | 1 | 0 | scaffold_37639 |
|  |  | 27 | 492 | 91.6 | 2 | 625 | chr8 | 68147360 | 68147962 | 10 | 10 | 0 | 0 | 0 | scaffold_37639 |
|  |  | 28 | 549 | 94.8 | 4 | 645 | chr8 | 57063932 | 57064881 | 8 | 7 | 1 | 0 | 0 | scaffold_36547 |
|  |  | 29 | 474 | 91.4 | 62 | 660 | chrX | 31585403 | 31585974 | 9 | 9 | 0 | 0 | 0 | scaffold_37356 |
|  |  | 30 | 448 | 92.7 | 74 | 600 | chrX | 122112112 | 122112654 | 7 | 6 | 1 | 0 | 0 | scaffold_37648 |
|  |  | 31 | 486 | 91.9 | 49 | 643 | chrX | 14214350 | 14214917 | 7 | 6 | 1 | 0 | 0 | scaffold_34445 |
| IMAGE:4293235 | BF672597 | 1 | 543 | 97.9 | 2 | 579 | chr14 | 50446283 | 50456553 | 16 | 16 | 0 | 0 | 0 | scaffold_32092 |
| IMAGE:4301223 | BF683777 | 1 | 681 | 96.6 | 17 | 780 | chr8 | 37662023 | 37662807 | 17 | 17 | 0 | 0 | 0 | scaffold_37182 |
|  |  | 2 | 733 | 99.5 | 7 | 775 | chrX | 117427682 | 117453430 | 24 | 17 | 1 | 6 | 0 | scaffold_29870 |
| IMAGE:4301483 | BF685670 | 1 | 685 | 97.7 | 2 | 740 | chr1 | 92769439 | 92774991 | 25 | 24 | 1 | 0 | 0 | scaffold_37509 |
|  |  | 2 | 555 | 92 | 19 | 703 | chr1 | 90956258 | 90956945 | 19 | 18 | 1 | 0 | 0 | scaffold_37509 |
|  |  | 3 | 527 | 93 | 31 | 666 | chr1 | 35243980 | 35244614 | 16 | 15 | 1 | 0 | 0 | scaffold_37598 |
|  |  | 4 | 533 | 92.5 | 13 | 654 | chr1 | 182435589 | 182436236 | 16 | 13 | 2 | 1 | 0 | scaffold_37227 |
|  |  | 5 | 525 | 92 | 19 | 666 | chr10 | 13104050 | 13104699 | 16 | 16 | 0 | 0 | 0 | scaffold_31942 |
|  |  | 6 | 568 | 93.5 | 2 | 666 | chr11 | 117969023 | 117969687 | 16 | 15 | 1 | 0 | 0 | scaffold_37627 |
|  |  | 7 | 552 | 93.2 | 42 | 703 | chr11 | 55900165 | 55900835 | 17 | 15 | 2 | 0 | 0 | scaffold_37107 |
|  |  | 8 | 566 | 95.5 | 12 | 641 | chr15 | 22701720 | 22702357 | 12 | 11 | 1 | 0 | 0 | scaffold_36937 |
|  |  | 9 | 554 | 93.1 | 5 | 666 | chr15 | 69071516 | 69072180 | 14 | 14 | 0 | 0 | 0 | scaffold_37693 |
|  |  | 10 | 550 | 92.6 | 19 | 703 | chr2 | 111820516 | 111821228 | 18 | 18 | 0 | 0 | 0 | scaffold_33337 |
|  |  | 11 | 634 | 93.1 | 2 | 761 | chr22 | 41403517 | 41404277 | 23 | 23 | 0 | 0 | 0 | scaffold_37547 |
|  |  | 12 | 484 | 90.9 | 19 | 624 | chr22 | 48371372 | 48371981 | 11 | 10 | 1 | 0 | 0 | scaffold_30607 |
|  |  | 13 | 501 | 93.3 | 42 | 647 | chr4 | 99404937 | 99405545 | 13 | 12 | 1 | 0 | 0 | scaffold_25950 |
|  |  | 14 | 528 | 94.2 | 19 | 761 | chr4 | 175036453 | 175037195 | 19 | 19 | 0 | 0 | 0 | scaffold_31924 |
|  |  | 15 | 563 | 93 | 19 | 704 | chr6 | 127664211 | 127664898 | 18 | 18 | 0 | 0 | 0 | scaffold_37501 |
|  |  | 16 | 587 | 91.2 | 22 | 778 | chr8 | 28179098 | 28179850 | 20 | 19 | 1 | 0 | 0 | scaffold_37701 |
|  |  | 17 | 561 | 91.1 | 5 | 764 | chr8 | 103892342 | 103893097 | 17 | 17 | 0 | 0 | 0 | scaffold_37544 |
|  |  | 18 | 291 | 92.4 | 282 | 623 | chr8 | 42216940 | 42217272 | 8 | 6 | 1 | 1 | 0 | scaffold_36987 |
| IMAGE:4307027 | BF978121 | 1 | 494 | 91.3 | 1 | 601 | chr10 | 65929876 | 65930478 | 5 | 5 | 0 | 0 | 0 | scaffold_37597 |
|  |  | 2 | 570 | 99.8 | 2 | 582 | chr15 | 58364173 | 58406241 | 10 | 8 | 0 | 2 | 0 | scaffold_37693 |
|  |  | 3 | 513 | 93.9 | 2 | 601 | chr4 | 154806996 | 154807596 | 5 | 3 | 2 | 0 | 0 | scaffold_37633 |
|  |  | 4 | 581 | 98.8 | 4 | 601 | chr9 | 33614219 | 33614819 | 4 | 3 | 1 | 0 | 0 | scaffold_37419 |
| IMAGE:4339901 | BF794781 | 1 | 646 | 98.3 | 1 | 688 | chrX | 1153598 | 1156448 | 11 | 0 | 0 | 0 | 11 |  |
|  |  | 2 | 646 | 98.3 | 1 | 688 | chrY | 1153598 | 1156448 | 11 | 0 | 0 | 0 | 11 |  |
| IMAGE:434133 | AA693897 | 1 | 391 | 100 | 1 | 392 | chr19 | 11137266 | 11137658 | 2 | 2 | 0 | 0 | 0 | scaffold_37160 |
| IMAGE:4368457 | BG109676 | 1 | 776 | 98 | 1 | 841 | chr19 | 57411108 | 57421072 | 22 | 13 | 1 | 8 | 0 | scaffold_37604 |
| IMAGE:4396729 | BF981742 | 1 | 405 | 95.7 | 264 | 723 | chr1 | 201050161 | 201050617 | 8 | 8 | 0 | 0 | 0 | scaffold_34717 |
|  |  | 2 | 372 | 94.3 | 276 | 722 | chr1 | 39182874 | 39183885 | 8 | 7 | 1 | 0 | 0 | scaffold_37598 |
|  |  | 3 | 402 | 94.6 | 265 | 734 | chr12 | 100769437 | 100769901 | 7 | 6 | 1 | 0 | 0 | scaffold_37435 |
|  |  | 4 | 420 | 96.7 | 259 | 723 | chr14 | 62058230 | 62058694 | 8 | 8 | 0 | 0 | 0 | scaffold_32092 |
|  |  | 5 | 436 | 94.2 | 243 | 787 | chr15 | 88907671 | 88908232 | 9 | 8 | 1 | 0 | 0 | scaffold_34672 |
|  |  | 6 | 475 | 92.7 | 93 | 722 | chr16 | 68496298 | 68496899 | 11 | 11 | 0 | 0 | 0 | scaffold_37667 |
|  |  | 7 | 377 | 95.5 | 269 | 705 | chr16 | 74425572 | 74426710 | 6 | 0 | 0 | 6 | 0 |  |
|  |  | 8 | 718 | 99.6 | 1 | 734 | chr2 | 198567407 | 198570661 | 11 | 11 | 0 | 0 | 0 | scaffold_37634 |
|  |  | 9 | 348 | 92.3 | 297 | 722 | chr2 | 235650429 | 235651177 | 7 | 6 | 1 | 0 | 0 | scaffold_37338 |
|  |  | 10 | 208 | 91.6 | 297 | 586 | chr5 | 139018992 | 139019611 | 5 | 5 | 0 | 0 | 0 | scaffold_14778 |
|  |  | 11 | 359 | 91.7 | 244 | 700 | chr5 | 126148912 | 126149992 | 10 | 0 | 0 | 10 | 0 |  |
| IMAGE:4403381 | BG032175 | 1 | 753 | 98 | 1 | 811 | chr1 | 145955433 | 145957568 | 15 | 0 | 0 | 15 | 0 |  |
|  |  | 2 | 723 | 97.9 | 1 | 773 | chr1 | 146117477 | 146119569 | 12 | 0 | 0 | 12 | 0 |  |
|  |  | 3 | 691 | 96.1 | 1 | 773 | chr1 | 142579870 | 142581964 | 13 | 9 | 2 | 0 | 2 | scaffold_23067 scaffold_6388 |
|  |  | 4 | 690 | 96.1 | 1 | 773 | chr1 | 143112481 | 143117729 | 13 | 0 | 0 | 0 | 13 |  |
|  |  | 5 | 636 | 94.9 | 2 | 718 | chr1 | 143971450 | 143973502 | 11 | 0 | 0 | 0 | 11 |  |
|  |  | 6 | 656 | 95.9 | 57 | 811 | chr1 | 145701994 | 145703360 | 15 | 0 | 0 | 15 | 0 |  |
|  |  | 7 | 610 | 94.4 | 57 | 773 | chr1 | 143762999 | 143764341 | 12 | 11 | 1 | 0 | 0 | scaffold_34605 |
|  |  | 8 | 592 | 94.7 | 57 | 773 | chr1 | 16271161 | 16272496 | 11 | 9 | 2 | 0 | 0 | scaffold_14405 |
|  |  | 9 | 527 | 94.3 | 175 | 773 | chr1_random | 1078991 | 1079582 | 10 | 0 | 0 | 0 | 10 |  |
| IMAGE:4477986 | BG255454 | 1 | 577 | 97.1 | 1 | 613 | chr1 | 96384249 | 96384861 | 1 | 1 | 0 | 0 | 0 | scaffold_37180 |
|  |  | 2 | 520 | 92.3 | 1 | 613 | chr1 | 191446318 | 191446922 | 3 | 2 | 1 | 0 | 0 | scaffold_37227 |
|  |  | 3 | 515 | 93.1 | 16 | 612 | chr12 | 17034848 | 17035444 | 1 | 1 | 0 | 0 | 0 | scaffold_37674 |
|  |  | 4 | 365 | 93.3 | 195 | 613 | chr12 | 42339941 | 42340358 | 2 | 2 | 0 | 0 | 0 | scaffold_37551 |
|  |  | 5 | 554 | 95.3 | 1 | 613 | chr12 | 19501261 | 19501887 | 2 | 0 | 2 | 0 | 0 | scaffold_37674 |
|  |  | 6 | 502 | 91.9 | 1 | 613 | chr13 | 26586617 | 26587226 | 4 | 4 | 0 | 0 | 0 | scaffold_37433 |
|  |  | 7 | 535 | 93.3 | 1 | 613 | chr15 | 50946198 | 50946809 | 2 | 1 | 1 | 0 | 0 | scaffold_37399 |
|  |  | 8 | 514 | 92.1 | 1 | 613 | chr16 | 27112170 | 27113087 | 4 | 4 | 0 | 0 | 0 | scaffold_37487 |
|  |  | 9 | 526 | 93 | 1 | 613 | chr19 | 40564876 | 40565989 | 3 | 3 | 0 | 0 | 0 | scaffold_37497 |
|  |  | 10 | 578 | 97.2 | 1 | 613 | chr2 | 106935957 | 106936570 | 2 | 0 | 2 | 0 | 0 | scaffold_31970 |
|  |  | 11 | 536 | 94.5 | 1 | 613 | chr3 | 39360590 | 39361201 | 3 | 2 | 1 | 0 | 0 | scaffold_37683 |
|  |  | 12 | 368 | 90.8 | 157 | 613 | chr3 | 139864814 | 139865269 | 4 | 3 | 1 | 0 | 0 | scaffold_36172 |
|  |  | 13 | 534 | 92.9 | 1 | 613 | chr3 | 185065884 | 185066489 | 6 | 6 | 0 | 0 | 0 | scaffold_37140 |
|  |  | 14 | 564 | 96.1 | 1 | 613 | chr4 | 106864627 | 106865243 | 2 | 2 | 0 | 0 | 0 | scaffold_36289 |
|  |  | 15 | 521 | 92 | 1 | 613 | chr4 | 136425402 | 136426012 | 3 | 1 | 2 | 0 | 0 | scaffold_37448 |
|  |  | 16 | 589 | 98 | 1 | 613 | chr5 | 14705599 | 14706211 | 1 | 1 | 0 | 0 | 0 | scaffold_37681 |
|  |  | 17 | 572 | 96.1 | 1 | 613 | chr5 | 43541487 | 43542095 | 2 | 2 | 0 | 0 | 0 | scaffold_37592 |
|  |  | 18 | 606 | 100 | 6 | 613 | chr6 | 74224299 | 74225380 | 3 | 2 | 0 | 1 | 0 | scaffold_37647 |
|  |  | 19 | 474 | 91.5 | 14 | 613 | chr7 | 87880333 | 87880930 | 5 | 5 | 0 | 0 | 0 | scaffold_37515 |
|  |  | 20 | 605 | 99 | 1 | 613 | chr7 | 22293317 | 22293928 | 2 | 0 | 1 | 1 | 0 | scaffold_37670 |
|  |  | 21 | 546 | 94.6 | 1 | 613 | chr7 | 143736834 | 143737447 | 2 | 2 | 0 | 0 | 0 | scaffold_37657 |
|  |  | 22 | 611 | 99.8 | 1 | 613 | chr9 | 131170880 | 131171492 | 1 | 0 | 1 | 0 | 0 | scaffold_37580 |
|  |  | 23 | 476 | 91.5 | 2 | 613 | chrX | 118099792 | 118100703 | 7 | 6 | 1 | 0 | 0 | scaffold_7558 |
|  |  | 24 | 255 | 97 | 343 | 613 | chrX | 84187940 | 84188210 | 1 | 1 | 0 | 0 | 0 | scaffold_37388 |
| IMAGE:4500491 | BG286219 | 1 | 608 | 96.9 | 1 | 649 | chr1 | 96384777 | 96385425 | 3 | 1 | 2 | 0 | 0 | scaffold_37180 |
|  |  | 2 | 543 | 91.9 | 1 | 649 | chr1 | 191446845 | 191447486 | 4 | 3 | 1 | 0 | 0 | scaffold_37227 |
|  |  | 3 | 538 | 92.1 | 1 | 637 | chr12 | 42340275 | 42340910 | 4 | 4 | 0 | 0 | 0 | scaffold_37551 |
|  |  | 4 | 536 | 90.9 | 1 | 649 | chr12 | 17035361 | 17036000 | 5 | 4 | 1 | 0 | 0 | scaffold_37674 |
|  |  | 5 | 567 | 94.5 | 1 | 639 | chr12 | 19500705 | 19501345 | 3 | 2 | 1 | 0 | 0 | scaffold_37674 |
|  |  | 6 | 536 | 91.6 | 1 | 637 | chr15 | 50945652 | 50946282 | 3 | 2 | 1 | 0 | 0 | scaffold_37399 |
|  |  | 7 | 529 | 91 | 1 | 649 | chr16 | 27111604 | 27112561 | 5 | 5 | 0 | 0 | 0 | scaffold_37487 |
|  |  | 8 | 555 | 92.3 | 1 | 649 | chr19 | 40565906 | 40566547 | 6 | 6 | 0 | 0 | 0 | scaffold_37497 |
|  |  | 9 | 576 | 95.9 | 1 | 649 | chr2 | 106935437 | 106936042 | 5 | 4 | 1 | 0 | 0 | scaffold_31970 |
|  |  | 10 | 409 | 90.1 | 132 | 649 | chr21 | 23684453 | 23684938 | 5 | 5 | 0 | 0 | 0 | scaffold_37624 |
|  |  | 11 | 549 | 92.3 | 1 | 649 | chr3 | 39361117 | 39361756 | 5 | 5 | 0 | 0 | 0 | scaffold_37683 |
|  |  | 12 | 513 | 91.2 | 6 | 639 | chr3 | 139865190 | 139865822 | 3 | 2 | 1 | 0 | 0 | scaffold_36172 |
|  |  | 13 | 542 | 93.4 | 1 | 625 | chr3 | 185065343 | 185065967 | 3 | 3 | 0 | 0 | 0 | scaffold_37140 |
|  |  | 14 | 606 | 97.5 | 1 | 639 | chr4 | 106865159 | 106865798 | 2 | 2 | 0 | 0 | 0 | scaffold_36289 |
|  |  | 15 | 546 | 91.8 | 1 | 649 | chr4 | 136425929 | 136426576 | 4 | 2 | 2 | 0 | 0 | scaffold_37448 |
|  |  | 16 | 624 | 98.1 | 1 | 649 | chr5 | 14705035 | 14705683 | 3 | 3 | 0 | 0 | 0 | scaffold_37681 |
|  |  | 17 | 598 | 96.1 | 1 | 649 | chr5 | 43540923 | 43541571 | 3 | 2 | 1 | 0 | 0 | scaffold_37592 |
|  |  | 18 | 641 | 99.7 | 1 | 649 | chr6 | 74223476 | 74224383 | 6 | 5 | 1 | 0 | 0 | scaffold_37647 |
|  |  | 19 | 634 | 98.6 | 1 | 649 | chr7 | 22292753 | 22293400 | 4 | 0 | 0 | 4 | 0 |  |
|  |  | 20 | 546 | 92.8 | 1 | 639 | chr7 | 143736279 | 143736918 | 2 | 0 | 2 | 0 | 0 | scaffold_37657 |
|  |  | 21 | 638 | 99.2 | 1 | 649 | chr9 | 131171408 | 131172056 | 3 | 2 | 1 | 0 | 0 | scaffold_37662 |
|  |  | 22 | 540 | 91.9 | 1 | 649 | chrX | 84187342 | 84188024 | 4 | 3 | 1 | 0 | 0 | scaffold_37388 |
|  |  | 23 | 446 | 90.8 | 24 | 588 | chrX | 118098817 | 118099853 | 2 | 2 | 0 | 0 | 0 | scaffold_7558 |
| IMAGE:4591511 | BG419839 | 1 | 512 | 91.1 | 2 | 619 | chr10 | 65929897 | 65930513 | 2 | 2 | 0 | 0 | 0 | scaffold_37597 |
|  |  | 2 | 590 | 99.8 | 22 | 619 | chr15 | 58362414 | 58394345 | 7 | 7 | 0 | 0 | 0 | scaffold_37693 |
|  |  | 3 | 526 | 94.2 | 20 | 619 | chr4 | 154806961 | 154807558 | 2 | 0 | 2 | 0 | 0 | scaffold_37633 |
|  |  | 4 | 602 | 98.7 | 2 | 619 | chr9 | 33614237 | 33614854 | 1 | 0 | 1 | 0 | 0 | scaffold_37419 |
| IMAGE:4594591 | BG401835 | 1 | 637 | 93.4 | 2 | 765 | chr1 | 96384739 | 96385557 | 11 | 9 | 2 | 0 | 0 | scaffold_37180 |
|  |  | 2 | 551 | 90.8 | 1 | 698 | chr1 | 191446806 | 191447546 | 10 | 9 | 1 | 0 | 0 | scaffold_37227 |
|  |  | 3 | 561 | 93.9 | 1 | 673 | chr12 | 19500711 | 19501384 | 10 | 9 | 1 | 0 | 0 | scaffold_37674 |
|  |  | 4 | 458 | 91.3 | 1 | 673 | chr13 | 26587103 | 26587769 | 5 | 5 | 0 | 0 | 0 | scaffold_37433 |
|  |  | 5 | 467 | 92.4 | 1 | 594 | chr16 | 27111699 | 27113079 | 9 | 9 | 0 | 0 | 0 | scaffold_37487 |
|  |  | 6 | 529 | 90.3 | 1 | 673 | chr19 | 40565867 | 40566532 | 10 | 10 | 0 | 0 | 0 | scaffold_37497 |
|  |  | 7 | 592 | 96.2 | 1 | 738 | chr2 | 106935333 | 106936081 | 13 | 12 | 1 | 0 | 0 | scaffold_31970 |
|  |  | 8 | 379 | 92.6 | 171 | 700 | chr21 | 23684453 | 23685138 | 12 | 12 | 0 | 0 | 0 | scaffold_37624 |
|  |  | 9 | 572 | 92.8 | 1 | 742 | chr3 | 39361078 | 39361865 | 12 | 12 | 0 | 0 | 0 | scaffold_37683 |
|  |  | 10 | 523 | 92.5 | 1 | 700 | chr3 | 139865146 | 139866030 | 10 | 9 | 1 | 0 | 0 | scaffold_36172 |
|  |  | 11 | 535 | 93 | 1 | 698 | chr3 | 185065260 | 185066005 | 11 | 11 | 0 | 0 | 0 | scaffold_37140 |
|  |  | 12 | 633 | 95.8 | 1 | 698 | chr4 | 106865120 | 106865867 | 11 | 11 | 0 | 0 | 0 | scaffold_36289 |
|  |  | 13 | 570 | 92.2 | 1 | 742 | chr4 | 136425890 | 136426685 | 16 | 14 | 2 | 0 | 0 | scaffold_37448 |
|  |  | 14 | 635 | 97 | 1 | 698 | chr5 | 14704975 | 14705722 | 11 | 11 | 0 | 0 | 0 | scaffold_37681 |
|  |  | 15 | 613 | 95.1 | 1 | 698 | chr5 | 43540863 | 43541610 | 11 | 10 | 1 | 0 | 0 | scaffold_37592 |
|  |  | 16 | 686 | 97.3 | 1 | 759 | chr6 | 74223350 | 74224422 | 15 | 14 | 1 | 0 | 0 | scaffold_37647 |
|  |  | 17 | 655 | 97.4 | 1 | 698 | chr7 | 22292693 | 22293439 | 12 | 1 | 1 | 10 | 0 | scaffold_37334 |
|  |  | 18 | 577 | 92.9 | 1 | 742 | chr7 | 143736161 | 143736957 | 12 | 10 | 2 | 0 | 0 | scaffold_37657 |
|  |  | 19 | 681 | 97.1 | 1 | 765 | chr9 | 131171369 | 131172188 | 13 | 12 | 1 | 0 | 0 | scaffold_37662 |
| IMAGE:4617167 | BG485167 | 1 | 240 | 98 | 1 | 254 | chr1 | 143189192 | 143189660 | 7 | 7 | 0 | 0 | 0 | scaffold_32121 |
| IMAGE:4640128 | BG477557 | 1 | 708 | 99.4 | 2 | 723 | chr12 | 107541704 | 107542431 | 7 | 6 | 1 | 0 | 0 | scaffold_26015 |
| IMAGE:4661406 | BG469590 | 1 | 519 | 96.9 | 1 | 565 | chr16 | 2166484 | 2167041 | 8 | 6 | 2 | 0 | 0 | scaffold_37104 |
| IMAGE:4661406 | BM083746 | 1 | 770 | 98.6 | 1 | 795 | chr16 | 2166484 | 2167272 | 5 | 2 | 3 | 0 | 0 | scaffold_37104 |
| IMAGE:4687737 | BG535214 | 1 | 455 | 90.1 | 2 | 682 | chr11 | 72377815 | 72378471 | 10 | 10 | 0 | 0 | 0 | scaffold_37360 |
|  |  | 2 | 550 | 92.3 | 5 | 682 | chr13 | 98500730 | 98501383 | 21 | 21 | 0 | 0 | 0 | scaffold_37611 |
|  |  | 3 | 520 | 94.3 | 9 | 681 | chr22 | 25607417 | 25608094 | 17 | 15 | 2 | 0 | 0 | scaffold_37586 |
|  |  | 4 | 581 | 96.1 | 25 | 682 | chr3 | 23934303 | 23935963 | 21 | 21 | 0 | 0 | 0 | scaffold_37683 |
|  |  | 5 | 573 | 93 | 1 | 678 | chr4 | 47623894 | 47624548 | 18 | 18 | 0 | 0 | 0 | scaffold_37561 |
|  |  | 6 | 601 | 94.5 | 2 | 682 | chr6 | 12622292 | 12622948 | 19 | 0 | 0 | 19 | 0 |  |
|  |  | 7 | 418 | 91.3 | 2 | 510 | chr7 | 134760850 | 134761353 | 5 | 4 | 1 | 0 | 0 | scaffold_37657 |
| IMAGE:469994 | AA028907 | 1 | 352 | 98.1 | 1 | 391 | chr5 | 9599170 | 9602014 | 8 | 8 | 0 | 0 | 0 | scaffold_37681 |
| IMAGE:4700537 | BG545172 | 1 | 638 | 99.2 | 1 | 652 | chr16 | 71864796 | 71870639 | 7 | 7 | 0 | 0 | 0 | scaffold_34584 |
|  |  | 2 | 595 | 96 | 1 | 652 | chr16 | 71873399 | 71886830 | 8 | 8 | 0 | 0 | 0 | scaffold_34584 |
| IMAGE:4763586 | BG684692 | 1 | 311 | 100 | 2 | 314 | chr11 | 57097270 | 57110447 | 3 | 3 | 0 | 0 | 0 | scaffold_37107 |
| IMAGE:4764621 | BG686596 | 1 | 430 | 97.5 | 4 | 468 | chr15 | 67461000 | 67463667 | 13 | 13 | 0 | 0 | 0 | scaffold_37693 |
|  |  | 2 | 241 | 91.4 | 9 | 299 | chr17 | 41695071 | 41695361 | 1 | 1 | 0 | 0 | 0 | scaffold_37479 |
|  |  | 3 | 400 | 94.2 | 4 | 443 | chr2 | 177268198 | 177268630 | 8 | 7 | 1 | 0 | 0 | scaffold_37634 |
| IMAGE:4765240 | BG685420 | 1 | 655 | 98.7 | 13 | 701 | chr1 | 44654999 | 44657706 | 12 | 12 | 0 | 0 | 0 | scaffold_34667 |
|  |  | 2 | 525 | 90.6 | 2 | 642 | chr10 | 121062547 | 121063186 | 2 | 1 | 1 | 0 | 0 | scaffold_37641 |
|  |  | 3 | 595 | 92.9 | 2 | 690 | chr14 | 104416236 | 104416917 | 7 | 6 | 1 | 0 | 0 | scaffold_37328 |
|  |  | 4 | 599 | 93.2 | 2 | 690 | chr15 | 19937805 | 19938486 | 7 | 0 | 0 | 0 | 7 |  |
|  |  | 5 | 421 | 91.3 | 9 | 568 | chr19 | 20051626 | 20052349 | 6 | 6 | 0 | 0 | 0 | scaffold_37664 |
|  |  | 6 | 633 | 96 | 2 | 690 | chr5 | 33207778 | 33208461 | 9 | 6 | 3 | 0 | 0 | scaffold_37614 scaffold_37626 |
|  |  | 7 | 527 | 91.8 | 2 | 642 | chr9 | 34213977 | 34214634 | 6 | 5 | 1 | 0 | 0 | scaffold_37419 |
| IMAGE:4772466 | BG614036 | 1 | 743 | 98.6 | 2 | 777 | chr14 | 100281216 | 100349476 | 15 | 15 | 0 | 0 | 0 | scaffold_37603 |
|  |  | 2 | 619 | 95.2 | 86 | 777 | chr3 | 52366138 | 52366832 | 12 | 12 | 0 | 0 | 0 | scaffold_37645 |
| IMAGE:4774660 | BG615353 | 1 | 742 | 99.7 | 2 | 751 | chr15 | 42719771 | 42726205 | 7 | 6 | 1 | 0 | 0 | scaffold_37412 |
| IMAGE:4795442 | BG706543 | 1 | 634 | 100 | 6 | 641 | chr2 | 241714995 | 241722641 | 3 | 1 | 1 | 1 | 0 | scaffold_36181 |
| IMAGE:4801427 | BG698380 | 1 | 630 | 99.8 | 1 | 633 | chr11 | 65260834 | 65261467 | 2 | 2 | 0 | 0 | 0 | scaffold_37417 |
| IMAGE:4801843 | BG696541 | 1 | 490 | 92.3 | 3 | 578 | chr1 | 232942823 | 232943389 | 6 | 6 | 0 | 0 | 0 | scaffold_37427 |
|  |  | 2 | 504 | 93.6 | 7 | 578 | chr10 | 120356817 | 120357384 | 4 | 4 | 0 | 0 | 0 | scaffold_37641 |
|  |  | 3 | 566 | 99.5 | 3 | 578 | chr11 | 18386824 | 18393088 | 6 | 6 | 0 | 0 | 0 | scaffold_37629 |
|  |  | 4 | 506 | 94.1 | 3 | 577 | chr2 | 42021796 | 42022382 | 4 | 4 | 0 | 0 | 0 | scaffold_37688 |
|  |  | 5 | 492 | 92.5 | 3 | 578 | chr2 | 84978972 | 84979537 | 3 | 3 | 0 | 0 | 0 | scaffold_36190 |
|  |  | 6 | 470 | 90.3 | 3 | 578 | chr4 | 4960498 | 4961067 | 5 | 5 | 0 | 0 | 0 | scaffold_32361 |
|  |  | 7 | 521 | 95.2 | 3 | 578 | chr9 | 14911384 | 14911957 | 3 | 1 | 2 | 0 | 0 | scaffold_37419 |
| IMAGE:4831331 | BG719019 | 1 | 524 | 91.7 | 40 | 673 | chr1 | 37675559 | 37676185 | 6 | 6 | 0 | 0 | 0 | scaffold_37598 |
|  |  | 2 | 662 | 99.6 | 1 | 673 | chr14 | 48569378 | 48573396 | 7 | 7 | 0 | 0 | 0 | scaffold_32092 |
| IMAGE:486584 | AA042933 | 1 | 385 | 98.2 | 1 | 406 | chr3 | 11618307 | 11737007 | 10 | 10 | 0 | 0 | 0 | scaffold_32934 |
| IMAGE:486584 | AA042980 | 1 | 436 | 97.6 | 1 | 468 | chr3 | 11574963 | 11575933 | 7 | 7 | 0 | 0 | 0 | scaffold_32934 |
| IMAGE:4891958 | BI195901 | 1 | 795 | 98.7 | 23 | 845 | chr15 | 58394283 | 58405595 | 10 | 9 | 1 | 0 | 0 | scaffold_37693 |
| IMAGE:4995837 | BI089608 | 1 | 811 | 98.6 | 1 | 854 | chr10 | 7834175 | 7848776 | 20 | 19 | 1 | 0 | 0 | scaffold_37507 |
|  |  | 2 | 703 | 90.8 | 1 | 854 | chr14 | 52447910 | 52448753 | 15 | 15 | 0 | 0 | 0 | scaffold_32092 |
| IMAGE:504537 | AA150035 | 1 | 440 | 99.8 | 1 | 442 | chr14 | 79929309 | 79929750 | 1 | 0 | 1 | 0 | 0 | scaffold_37670 |
| IMAGE:504537 | AA150050 | 1 | 414 | 96 | 1 | 440 | chr14 | 79930197 | 79930628 | 8 | 7 | 1 | 0 | 0 | scaffold_37670 |
| IMAGE:504602 | AA150129 | 1 | 294 | 98 | 2 | 311 | chr6 | 139482202 | 139482509 | 2 | 1 | 1 | 0 | 0 | scaffold_37467 |
| IMAGE:504602 | AA152136 | 1 | 412 | 95.3 | 2 | 443 | chr6 | 139481775 | 139482206 | 11 | 11 | 0 | 0 | 0 | scaffold_37467 |
| IMAGE:504789 | AA148403 | 1 | 304 | 99.7 | 3 | 309 | chr8 | 30593246 | 30593718 | 2 | 2 | 0 | 0 | 0 | scaffold_3527 |
| IMAGE:504789 | AA148404 | 1 | 222 | 99.6 | 1 | 226 | chr6 | 111925714 | 111925939 | 1 | 1 | 0 | 0 | 0 | scaffold_37579 |
| IMAGE:5086652 | BI712430 | 1 | 549 | 98.1 | 1 | 573 | chr1 | 96385394 | 96385987 | 3 | 2 | 1 | 0 | 0 | scaffold_37180 |
|  |  | 2 | 457 | 92.4 | 18 | 573 | chr1 | 191447455 | 191448407 | 4 | 2 | 2 | 0 | 0 | scaffold_37227 |
|  |  | 3 | 233 | 91.8 | 1 | 287 | chr1 | 194355043 | 194355339 | 4 | 4 | 0 | 0 | 0 | scaffold_37677 |
|  |  | 4 | 479 | 96.5 | 10 | 547 | chr10 | 113972671 | 113973220 | 5 | 5 | 0 | 0 | 0 | scaffold_32851 |
|  |  | 5 | 298 | 95.4 | 1 | 328 | chr10 | 128797200 | 128797527 | 1 | 1 | 0 | 0 | 0 | scaffold_27098 |
|  |  | 6 | 502 | 93.3 | 1 | 573 | chr12 | 19500159 | 19500727 | 2 | 2 | 0 | 0 | 0 | scaffold_37674 |
|  |  | 7 | 463 | 91.1 | 1 | 573 | chr12 | 95796630 | 95797567 | 7 | 5 | 2 | 0 | 0 | scaffold_37435 |
|  |  | 8 | 323 | 97.4 | 1 | 348 | chr12 | 8124897 | 8125281 | 3 | 1 | 2 | 0 | 0 | scaffold_37591 |
|  |  | 9 | 498 | 94.2 | 7 | 573 | chr12 | 42340890 | 42341494 | 4 | 4 | 0 | 0 | 0 | scaffold_37551 |
|  |  | 10 | 466 | 93.7 | 7 | 573 | chr12 | 17035969 | 17036858 | 6 | 6 | 0 | 0 | 0 | scaffold_37674 |
|  |  | 11 | 456 | 92.2 | 7 | 568 | chr13 | 26587758 | 26588329 | 8 | 7 | 1 | 0 | 0 | scaffold_37433 |
|  |  | 12 | 343 | 97.8 | 7 | 365 | chr13 | 93969577 | 93969935 | 1 | 0 | 1 | 0 | 0 | scaffold_37396 |
|  |  | 13 | 507 | 94.9 | 1 | 573 | chr15 | 50945100 | 50945672 | 3 | 3 | 0 | 0 | 0 | scaffold_37399 |
|  |  | 14 | 491 | 93.9 | 12 | 573 | chr16 | 27111066 | 27111635 | 3 | 2 | 1 | 0 | 0 | scaffold_37487 |
|  |  | 15 | 337 | 96.9 | 10 | 369 | chr17 | 17689348 | 17690009 | 4 | 3 | 1 | 0 | 0 | scaffold_34420 |
|  |  | 16 | 501 | 93.7 | 1 | 573 | chr19 | 40566516 | 40567088 | 1 | 1 | 0 | 0 | 0 | scaffold_37497 |
|  |  | 17 | 532 | 96.1 | 1 | 573 | chr2 | 106934898 | 106935468 | 2 | 1 | 1 | 0 | 0 | scaffold_31970 |
|  |  | 18 | 505 | 94.1 | 1 | 573 | chr21 | 23684907 | 23685479 | 1 | 1 | 0 | 0 | 0 | scaffold_37624 |
|  |  | 19 | 483 | 93.4 | 1 | 573 | chr3 | 185064774 | 185065351 | 6 | 6 | 0 | 0 | 0 | scaffold_37140 |
|  |  | 20 | 510 | 95 | 11 | 573 | chr3 | 39361725 | 39362285 | 3 | 2 | 1 | 0 | 0 | scaffold_37683 |
|  |  | 21 | 464 | 91.3 | 1 | 573 | chr3 | 139865800 | 139866385 | 9 | 9 | 0 | 0 | 0 | scaffold_36172 |
|  |  | 22 | 297 | 92.6 | 1 | 374 | chr4 | 96639671 | 96640046 | 5 | 5 | 0 | 0 | 0 | scaffold_37147 |
|  |  | 23 | 542 | 97.4 | 1 | 573 | chr4 | 106865776 | 106866349 | 2 | 2 | 0 | 0 | 0 | scaffold_36289 |
|  |  | 24 | 496 | 94 | 1 | 573 | chr4 | 136426545 | 136427472 | 4 | 4 | 0 | 0 | 0 | scaffold_37448 |
|  |  | 25 | 559 | 98.8 | 1 | 573 | chr5 | 14704494 | 14705066 | 1 | 1 | 0 | 0 | 0 | scaffold_37681 |
|  |  | 26 | 541 | 96.7 | 1 | 573 | chr5 | 43540385 | 43540954 | 2 | 1 | 1 | 0 | 0 | scaffold_37592 |
|  |  | 27 | 343 | 91.3 | 12 | 433 | chr5 | 126591375 | 126591792 | 3 | 3 | 0 | 0 | 0 | scaffold_37656 |
|  |  | 28 | 572 | 100 | 1 | 573 | chr6 | 74222840 | 74223507 | 2 | 2 | 0 | 0 | 0 | scaffold_37647 |
|  |  | 29 | 569 | 99.7 | 1 | 573 | chr7 | 22292212 | 22292784 | 1 | 0 | 1 | 0 | 0 | scaffold_37334 |
|  |  | 30 | 511 | 94.8 | 1 | 572 | chr7 | 143735421 | 143736300 | 2 | 2 | 0 | 0 | 0 | scaffold_37657 |
|  |  | 31 | 457 | 92.2 | 7 | 573 | chr7 | 87881463 | 87882579 | 8 | 4 | 2 | 2 | 0 | scaffold_37515 |
|  |  | 32 | 571 | 99.8 | 1 | 573 | chr9 | 131172025 | 131172597 | 1 | 1 | 0 | 0 | 0 | scaffold_37662 |
|  |  | 33 | 475 | 92.5 | 12 | 573 | chrX | 84186804 | 84187373 | 6 | 6 | 0 | 0 | 0 | scaffold_37388 |
|  |  | 34 | 368 | 92.4 | 16 | 535 | chrX | 118097815 | 118098753 | 8 | 8 | 0 | 0 | 0 | scaffold_7558 |
| IMAGE:5086652 | BI712675 | 1 | 567 | 97.5 | 11 | 607 | chr1 | 96384957 | 96385553 | 1 | 0 | 1 | 0 | 0 | scaffold_37180 |
|  |  | 2 | 512 | 93 | 11 | 606 | chr1 | 191447018 | 191447613 | 1 | 1 | 0 | 0 | 0 | scaffold_37227 |
|  |  | 3 | 513 | 92.5 | 11 | 607 | chr12 | 17035541 | 17036128 | 3 | 2 | 1 | 0 | 0 | scaffold_37674 |
|  |  | 4 | 489 | 91.9 | 11 | 589 | chr12 | 42340454 | 42341031 | 2 | 2 | 0 | 0 | 0 | scaffold_37551 |
|  |  | 5 | 526 | 94.1 | 11 | 607 | chr12 | 19500568 | 19501165 | 2 | 1 | 1 | 0 | 0 | scaffold_37674 |
|  |  | 6 | 510 | 93 | 19 | 600 | chr15 | 50945521 | 50946094 | 3 | 2 | 1 | 0 | 0 | scaffold_37399 |
|  |  | 7 | 481 | 90.4 | 11 | 607 | chr16 | 27111476 | 27112074 | 2 | 2 | 0 | 0 | 0 | scaffold_37487 |
|  |  | 8 | 501 | 91.5 | 11 | 606 | chr19 | 40566085 | 40566674 | 3 | 3 | 0 | 0 | 0 | scaffold_37497 |
|  |  | 9 | 567 | 97.6 | 12 | 606 | chr2 | 106935310 | 106935904 | 1 | 1 | 0 | 0 | 0 | scaffold_31970 |
|  |  | 10 | 481 | 90.6 | 11 | 607 | chr21 | 23684501 | 23685066 | 2 | 2 | 0 | 0 | 0 | scaffold_37624 |
|  |  | 11 | 484 | 91.4 | 11 | 593 | chr3 | 39361297 | 39361870 | 3 | 2 | 1 | 0 | 0 | scaffold_37683 |
|  |  | 12 | 460 | 90.7 | 31 | 596 | chr3 | 139865385 | 139865949 | 3 | 3 | 0 | 0 | 0 | scaffold_36172 |
|  |  | 13 | 513 | 93 | 11 | 607 | chr3 | 185065192 | 185065788 | 1 | 1 | 0 | 0 | 0 | scaffold_37140 |
|  |  | 14 | 567 | 97.5 | 11 | 607 | chr4 | 106865339 | 106865935 | 1 | 1 | 0 | 0 | 0 | scaffold_36289 |
|  |  | 15 | 506 | 92.9 | 11 | 600 | chr4 | 136426108 | 136426697 | 1 | 0 | 1 | 0 | 0 | scaffold_37448 |
|  |  | 16 | 575 | 98.2 | 11 | 607 | chr5 | 14704907 | 14705503 | 1 | 1 | 0 | 0 | 0 | scaffold_37681 |
|  |  | 17 | 560 | 96.8 | 16 | 607 | chr5 | 43540798 | 43541386 | 2 | 1 | 1 | 0 | 0 | scaffold_37592 |
|  |  | 18 | 594 | 100 | 11 | 606 | chr6 | 74223349 | 74224120 | 3 | 2 | 1 | 0 | 0 | scaffold_37647 |
|  |  | 19 | 589 | 99.3 | 11 | 607 | chr7 | 22292625 | 22293221 | 1 | 0 | 1 | 0 | 0 | scaffold_37334 |
|  |  | 20 | 493 | 91.3 | 11 | 607 | chr7 | 143736142 | 143736738 | 1 | 0 | 1 | 0 | 0 | scaffold_37657 |
|  |  | 21 | 591 | 99.5 | 11 | 607 | chr9 | 131171588 | 131172184 | 1 | 0 | 1 | 0 | 0 | scaffold_37662 |
|  |  | 22 | 482 | 91.4 | 11 | 596 | chrX | 84187225 | 84187844 | 2 | 1 | 1 | 0 | 0 | scaffold_37388 |
| IMAGE:5087516 | BI261376 | 1 | 490 | 91 | 5 | 643 | chr1 | 163903957 | 163904582 | 7 | 6 | 1 | 0 | 0 | scaffold_37675 |
|  |  | 2 | 488 | 90.8 | 9 | 643 | chr1 | 168892360 | 168892984 | 11 | 11 | 0 | 0 | 0 | scaffold_37122 |
|  |  | 3 | 396 | 90.7 | 80 | 562 | chr14 | 20208720 | 20209197 | 6 | 5 | 1 | 0 | 0 | scaffold_37444 |
|  |  | 4 | 578 | 97.8 | 2 | 643 | chr15 | 64509614 | 64512985 | 13 | 13 | 0 | 0 | 0 | scaffold_37693 |
|  |  | 5 | 574 | 95 | 2 | 643 | chr3 | 186456887 | 186457518 | 11 | 11 | 0 | 0 | 0 | scaffold_37548 |
|  |  | 6 | 538 | 93 | 2 | 643 | chr9 | 7467728 | 7468359 | 9 | 9 | 0 | 0 | 0 | scaffold_37582 |
| IMAGE:5087516 | BI261993 | 1 | 482 | 93.7 | 20 | 585 | chr1 | 168891658 | 168892224 | 7 | 7 | 0 | 0 | 0 | scaffold_37122 |
|  |  | 2 | 473 | 94.2 | 20 | 585 | chr1 | 163904718 | 163905287 | 9 | 8 | 1 | 0 | 0 | scaffold_37675 |
|  |  | 3 | 558 | 99.6 | 20 | 585 | chr15 | 64507552 | 64509209 | 5 | 3 | 1 | 1 | 0 | scaffold_37693 |
|  |  | 4 | 543 | 98.1 | 20 | 585 | chr3 | 186456185 | 186456751 | 2 | 2 | 0 | 0 | 0 | scaffold_37548 |
|  |  | 5 | 524 | 96.5 | 20 | 585 | chr9 | 7467029 | 7467991 | 3 | 2 | 1 | 0 | 0 | scaffold_37582 |
| IMAGE:5163490 | BI520286 | 1 | 814 | 98.6 | 8 | 891 | chr11 | 6589718 | 6595103 | 20 | 20 | 0 | 0 | 0 | scaffold_37629 |
| IMAGE:5163490 | BI520987 | 1 | 794 | 97.1 | 2 | 854 | chr11 | 6595052 | 6596357 | 22 | 22 | 0 | 0 | 0 | scaffold_37629 |
| IMAGE:5207904 | BI772441 | 1 | 605 | 100 | 1 | 606 | chr7 | 142452993 | 142454093 | 2 | 2 | 0 | 0 | 0 | scaffold_37657 |
| IMAGE:5210029 | BI767840 | 1 | 584 | 96.7 | 2 | 625 | chr6 | 123090772 | 123091392 | 6 | 6 | 0 | 0 | 0 | scaffold_37501 |
| IMAGE:5226869 | BI835070 | 1 | 675 | 96.1 | 4 | 763 | chr7 | 84180702 | 84181583 | 9 | 6 | 3 | 0 | 0 | scaffold_37515 |
|  |  | 2 | 734 | 99.5 | 3 | 751 | chr9 | 134976401 | 134979890 | 8 | 7 | 1 | 0 | 0 | scaffold_27091 |
| IMAGE:5242323 | BI916293 | 1 | 718 | 99 | 1 | 735 | chr17 | 80491858 | 80497294 | 8 | 8 | 0 | 0 | 0 | scaffold_34348 |
|  |  | 2 | 716 | 99 | 1 | 735 | chr17_random | 2213089 | 2218550 | 10 | 0 | 0 | 0 | 10 |  |
| IMAGE:5261412 | BI546852 | 1 | 478 | 100 | 10 | 490 | chr7 | 781681 | 812338 | 4 | 1 | 2 | 1 | 0 | scaffold_36945 scaffold_14598 scaffold_36211 |
| IMAGE:5301515 | BI603198 | 1 | 526 | 97.5 | 5 | 558 | chr1 | 189973533 | 189974086 | 1 | 0 | 1 | 0 | 0 | scaffold_37099 |
|  |  | 2 | 523 | 98.1 | 20 | 558 | chr16 | 60864509 | 60865046 | 2 | 1 | 1 | 0 | 0 | scaffold_37666 |
|  |  | 3 | 489 | 94.2 | 6 | 558 | chr17 | 10362607 | 10363159 | 1 | 0 | 1 | 0 | 0 | scaffold_37659 |
|  |  | 4 | 432 | 92.5 | 6 | 535 | chr19 | 7636565 | 7637387 | 5 | 4 | 1 | 0 | 0 | scaffold_37480 |
|  |  | 5 | 529 | 99.8 | 6 | 544 | chr2 | 55434358 | 55437277 | 6 | 6 | 0 | 0 | 0 | scaffold_37688 |
|  |  | 6 | 462 | 93.1 | 17 | 544 | chr20 | 17492461 | 17492985 | 2 | 1 | 1 | 0 | 0 | scaffold_37176 |
|  |  | 7 | 440 | 92.4 | 12 | 549 | chr20 | 36363070 | 36363583 | 7 | 7 | 0 | 0 | 0 | scaffold_37418 |
|  |  | 8 | 423 | 90.2 | 17 | 544 | chr5 | 79878390 | 79878899 | 6 | 6 | 0 | 0 | 0 | scaffold_37690 |
|  |  | 9 | 492 | 94.8 | 5 | 544 | chr6 | 113948234 | 113948769 | 3 | 2 | 1 | 0 | 0 | scaffold_37579 |
|  |  | 10 | 490 | 93.9 | 6 | 557 | chr7 | 156401879 | 156402426 | 5 | 3 | 2 | 0 | 0 | scaffold_34623 |
|  |  | 11 | 445 | 91.6 | 17 | 547 | chrX | 3230293 | 3230806 | 4 | 3 | 1 | 0 | 0 | scaffold_36354 |
| IMAGE:5395639 | BI860223 | 1 | 701 | 100 | 1 | 707 | chr16 | 11933304 | 11945506 | 7 | 6 | 1 | 0 | 0 | scaffold_34722 scaffold_37297 scaffold_14297 |
|  |  | 2 | 538 | 90.4 | 11 | 692 | chrX | 50404825 | 50405501 | 4 | 0 | 0 | 4 | 0 |  |
| IMAGE:545004 | AA075486 | 1 | 475 | 92.9 | 1 | 566 | chr16 | 71815079 | 71815642 | 7 | 7 | 0 | 0 | 0 | scaffold_34584 |
|  |  | 2 | 552 | 99.5 | 1 | 566 | chr18 | 41916102 | 41918393 | 7 | 7 | 0 | 0 | 0 | scaffold_37259 |
|  |  | 3 | 369 | 95.6 | 3 | 408 | chr2 | 156328688 | 156329094 | 2 | 1 | 1 | 0 | 0 | scaffold_30774 |
|  |  | 4 | 446 | 90.9 | 3 | 566 | chr9 | 41983552 | 41984082 | 7 | 6 | 1 | 0 | 0 | scaffold_37074 |
|  |  | 5 | 448 | 91.1 | 3 | 566 | chr9 | 40690756 | 40691285 | 8 | 6 | 2 | 0 | 0 | scaffold_37074 |
|  |  | 6 | 446 | 90.9 | 3 | 566 | chr9 | 40075752 | 40076282 | 7 | 0 | 0 | 7 | 0 |  |
|  |  | 7 | 472 | 93.5 | 3 | 561 | chr9 | 41102072 | 41102630 | 6 | 0 | 0 | 0 | 6 |  |
|  |  | 8 | 446 | 90.9 | 3 | 566 | chr9_random | 1176876 | 1177406 | 7 | 0 | 0 | 0 | 7 |  |
| IMAGE:5455785 | BM047177 | 1 | 644 | 99.1 | 1 | 662 | chr4 | 77299457 | 77319611 | 8 | 8 | 0 | 0 | 0 | scaffold_37596 |
| IMAGE:5504759 | BM464712 | 1 | 898 | 95.4 | 34 | 1054 | chr1 | 96384672 | 96385688 | 8 | 7 | 1 | 0 | 0 | scaffold_37180 |
|  |  | 2 | 775 | 92.2 | 35 | 1054 | chr1 | 191446742 | 191447744 | 9 | 8 | 1 | 0 | 0 | scaffold_37227 |
|  |  | 3 | 749 | 92 | 34 | 925 | chr12 | 17035256 | 17036139 | 6 | 5 | 1 | 0 | 0 | scaffold_37674 |
|  |  | 4 | 745 | 92.3 | 34 | 935 | chr12 | 42340170 | 42341070 | 6 | 6 | 0 | 0 | 0 | scaffold_37551 |
|  |  | 5 | 775 | 94.1 | 54 | 935 | chr12 | 19500547 | 19501429 | 4 | 3 | 1 | 0 | 0 | scaffold_37674 |
|  |  | 6 | 757 | 92 | 34 | 935 | chr15 | 50945493 | 50946387 | 6 | 5 | 1 | 0 | 0 | scaffold_37399 |
|  |  | 7 | 779 | 91.1 | 40 | 1001 | chr19 | 40565807 | 40566760 | 9 | 9 | 0 | 0 | 0 | scaffold_37497 |
|  |  | 8 | 811 | 96.1 | 34 | 935 | chr2 | 106935288 | 106936147 | 6 | 4 | 2 | 0 | 0 | scaffold_31970 |
|  |  | 9 | 795 | 91.6 | 34 | 1001 | chr3 | 39361012 | 39361969 | 8 | 6 | 2 | 0 | 0 | scaffold_37683 |
|  |  | 10 | 726 | 90.5 | 36 | 965 | chr3 | 139865084 | 139866008 | 10 | 9 | 1 | 0 | 0 | scaffold_36172 |
|  |  | 11 | 779 | 93.3 | 34 | 931 | chr3 | 185065175 | 185066071 | 6 | 6 | 0 | 0 | 0 | scaffold_37140 |
|  |  | 12 | 851 | 97.5 | 34 | 933 | chr4 | 106865054 | 106865954 | 5 | 5 | 0 | 0 | 0 | scaffold_36289 |
|  |  | 13 | 765 | 92.4 | 36 | 935 | chr4 | 136425827 | 136426725 | 6 | 4 | 2 | 0 | 0 | scaffold_37448 |
|  |  | 14 | 871 | 98.4 | 34 | 935 | chr5 | 14704886 | 14705788 | 4 | 4 | 0 | 0 | 0 | scaffold_37681 |
|  |  | 15 | 838 | 96.4 | 34 | 935 | chr5 | 43540777 | 43541676 | 4 | 3 | 1 | 0 | 0 | scaffold_37592 |
|  |  | 16 | 929 | 98.3 | 34 | 1001 | chr6 | 74223168 | 74224488 | 10 | 9 | 1 | 0 | 0 | scaffold_37647 |
|  |  | 17 | 887 | 99.3 | 34 | 935 | chr7 | 22292604 | 22293505 | 5 | 2 | 1 | 2 | 0 | scaffold_37334 |
|  |  | 18 | 788 | 92.7 | 34 | 965 | chr7 | 143736092 | 143737023 | 5 | 4 | 1 | 0 | 0 | scaffold_37657 |
|  |  | 19 | 891 | 99.6 | 34 | 935 | chr9 | 131171303 | 131172205 | 4 | 3 | 1 | 0 | 0 | scaffold_37662 |
|  |  | 20 | 775 | 91.5 | 34 | 1001 | chrX | 84187129 | 84188129 | 7 | 6 | 1 | 0 | 0 | scaffold_37388 |
|  |  | 21 | 605 | 90.7 | 34 | 895 | chrX | 118098652 | 118099969 | 6 | 6 | 0 | 0 | 0 | scaffold_7558 |
| IMAGE:5517176 | BM423933 | 1 | 957 | 98.3 | 1 | 1014 | chr22 | 29146320 | 29149474 | 13 | 12 | 1 | 0 | 0 | scaffold_37586 |
| IMAGE:5550707 | BM560783 | 1 | 974 | 97.8 | 2 | 1027 | chr12 | 118945579 | 118946602 | 13 | 12 | 1 | 0 | 0 | scaffold_37378 |
| IMAGE:5561593 | BM479264 | 1 | 392 | 99 | 6 | 406 | chr19 | 17527495 | 17531232 | 2 | 2 | 0 | 0 | 0 | scaffold_8856 |
| IMAGE:5580683 | BM465832 | 1 | 626 | 90.5 | 51 | 831 | chr15 | 56700697 | 56701467 | 5 | 5 | 0 | 0 | 0 | scaffold_37693 |
|  |  | 2 | 772 | 96 | 51 | 903 | chr4 | 89271375 | 89272213 | 8 | 8 | 0 | 0 | 0 | scaffold_37561 |
|  |  | 3 | 713 | 92.9 | 51 | 903 | chr4 | 13088974 | 13089809 | 9 | 9 | 0 | 0 | 0 | scaffold_37099 |
|  |  | 4 | 853 | 99.1 | 1 | 903 | chr6 | 44261723 | 44265059 | 12 | 12 | 0 | 0 | 0 | scaffold_37583 scaffold_22744 |
| IMAGE:5633807 | BM511082 | 1 | 418 | 96.6 | 8 | 459 | chr1 | 609649 | 610099 | 2 | 0 | 0 | 0 | 2 |  |
|  |  | 2 | 287 | 92.7 | 109 | 451 | chr4 | 12392801 | 12393142 | 4 | 1 | 2 | 1 | 0 | scaffold_37099 scaffold_37548 |
|  |  | 3 | 432 | 98.2 | 8 | 459 | chrM | 9238 | 9688 | 2 | 0 | 0 | 0 | 2 |  |
| IMAGE:5671550 | BI963035 | 1 | 355 | 100 | 1 | 355 | chr9_random | 2341357 | 2341711 | 1 | 1 | 0 | 0 | 0 | scaffold_5580 |
| IMAGE:5671550 | BI966839 | 1 | 324 | 100 | 1 | 324 | chr9_random | 2341386 | 2341709 | 1 | 1 | 0 | 0 | 0 | scaffold_5580 |
| IMAGE:5673379 | BM023307 | 1 | 314 | 99.4 | 14 | 331 | chr3 | 15068719 | 15069036 | 1 | 1 | 0 | 0 | 0 | scaffold_37683 |
| IMAGE:5673379 | BM023613 | 1 | 417 | 99.1 | 1 | 425 | chr3 | 15068719 | 15069143 | 1 | 1 | 0 | 0 | 0 | scaffold_37683 |
| IMAGE:5676602 | BM128763 | 1 | 569 | 99.8 | 4 | 574 | chr17 | 43512228 | 43512798 | 1 | 1 | 0 | 0 | 0 | scaffold_37116 |
| IMAGE:5676602 | BM128991 | 1 | 490 | 100 | 1 | 491 | chr17 | 43512542 | 43520116 | 2 | 2 | 0 | 0 | 0 | scaffold_37116 |
| IMAGE:5722059 | BM903645 | 1 | 777 | 98.4 | 1 | 817 | chr20 | 43511286 | 43524817 | 12 | 12 | 0 | 0 | 0 | scaffold_37443 |
| IMAGE:5736258 | BM907829 | 1 | 745 | 92.5 | 24 | 902 | chr1 | 203785943 | 203786807 | 7 | 7 | 0 | 0 | 0 | scaffold_37640 |
|  |  | 2 | 633 | 90.6 | 24 | 815 | chr1 | 45530006 | 45530796 | 5 | 2 | 3 | 0 | 0 | scaffold_34667 |
|  |  | 3 | 622 | 91.2 | 55 | 815 | chr1 | 7826859 | 7828045 | 7 | 7 | 0 | 0 | 0 | scaffold_37442 |
|  |  | 4 | 791 | 95.5 | 24 | 901 | chr10 | 65006739 | 65007612 | 9 | 6 | 3 | 0 | 0 | scaffold_37597 |
|  |  | 5 | 729 | 93.3 | 24 | 875 | chr10 | 33350500 | 33351347 | 4 | 4 | 0 | 0 | 0 | scaffold_37564 |
|  |  | 6 | 727 | 92.3 | 24 | 889 | chr10 | 88055101 | 88055945 | 7 | 5 | 2 | 0 | 0 | scaffold_36993 |
|  |  | 7 | 310 | 92.5 | 521 | 904 | chr13 | 21175433 | 21175823 | 7 | 6 | 1 | 0 | 0 | scaffold_32325 |
|  |  | 8 | 834 | 97.1 | 24 | 908 | chr14 | 68342024 | 68342902 | 7 | 5 | 2 | 0 | 0 | scaffold_37460 |
|  |  | 9 | 706 | 91 | 24 | 907 | chr14 | 66795412 | 66796290 | 13 | 4 | 1 | 8 | 0 | scaffold_37703 |
|  |  | 10 | 446 | 90.5 | 349 | 898 | chr14 | 33694354 | 33694898 | 7 | 7 | 0 | 0 | 0 | scaffold_37441 |
|  |  | 11 | 784 | 95 | 24 | 896 | chr15 | 45738332 | 45739192 | 10 | 9 | 1 | 0 | 0 | scaffold_37412 |
|  |  | 12 | 720 | 91.4 | 24 | 901 | chr16 | 86340539 | 86341409 | 7 | 0 | 0 | 7 | 0 |  |
|  |  | 13 | 816 | 97 | 24 | 909 | chr18 | 35166564 | 35167627 | 7 | 6 | 1 | 0 | 0 | scaffold_37707 |
|  |  | 14 | 688 | 90.6 | 24 | 900 | chr19 | 21124224 | 21125085 | 11 | 9 | 2 | 0 | 0 | scaffold_37664 |
|  |  | 15 | 710 | 92.5 | 24 | 868 | chr20 | 36758527 | 36759369 | 6 | 6 | 0 | 0 | 0 | scaffold_37418 |
|  |  | 16 | 799 | 95.5 | 24 | 902 | chr22 | 18154367 | 18155240 | 7 | 5 | 2 | 0 | 0 | scaffold_37421 |
|  |  | 17 | 786 | 94.9 | 24 | 908 | chr3 | 122533854 | 122534727 | 7 | 6 | 1 | 0 | 0 | scaffold_37456 |
|  |  | 18 | 737 | 92.6 | 24 | 897 | chr4 | 57137839 | 57138703 | 11 | 11 | 0 | 0 | 0 | scaffold_37623 |
|  |  | 19 | 791 | 95.8 | 24 | 889 | chr4 | 114168001 | 114168858 | 7 | 5 | 1 | 1 | 0 | scaffold_32345 |
|  |  | 20 | 683 | 91.7 | 31 | 863 | chr4 | 83870339 | 83871180 | 6 | 6 | 0 | 0 | 0 | scaffold_37596 |
|  |  | 21 | 612 | 91.1 | 24 | 897 | chr4 | 189966357 | 189967072 | 8 | 8 | 0 | 0 | 0 | scaffold_34255 |
|  |  | 22 | 525 | 90.1 | 237 | 903 | chr5 | 172633692 | 172634354 | 6 | 5 | 1 | 0 | 0 | scaffold_37615 |
|  |  | 23 | 729 | 91.8 | 24 | 898 | chr6 | 10226667 | 10227536 | 7 | 6 | 1 | 0 | 0 | scaffold_35233 |
|  |  | 24 | 722 | 92.3 | 29 | 889 | chr6 | 64255391 | 64256248 | 8 | 2 | 1 | 5 | 0 | scaffold_37200 |
|  |  | 25 | 617 | 92.7 | 24 | 757 | chr6 | 29879051 | 29879783 | 4 | 4 | 0 | 0 | 0 | scaffold_37619 |
|  |  | 26 | 683 | 91 | 24 | 875 | chr7 | 101956119 | 101956959 | 5 | 0 | 0 | 0 | 5 |  |
|  |  | 27 | 685 | 91.2 | 24 | 875 | chr7 | 76356762 | 76357602 | 5 | 0 | 0 | 0 | 5 |  |
|  |  | 28 | 618 | 92.2 | 24 | 772 | chr7 | 24994096 | 24994858 | 6 | 0 | 0 | 6 | 0 |  |
|  |  | 29 | 833 | 99.3 | 30 | 889 | chr9 | 131491821 | 131494324 | 12 | 12 | 0 | 0 | 0 | scaffold_34597 |
|  |  | 30 | 686 | 91.2 | 24 | 867 | chr9 | 14194243 | 14195079 | 7 | 2 | 1 | 4 | 0 | scaffold_37582 |
|  |  | 31 | 833 | 99.3 | 30 | 889 | chr9_random | 2156779 | 2159282 | 12 | 0 | 0 | 0 | 12 |  |
| IMAGE:5785295 | BQ219355 | 1 | 889 | 99.2 | 1 | 908 | chr3 | 150779948 | 150791175 | 9 | 9 | 0 | 0 | 0 | scaffold_19089 |
| IMAGE:5809347 | BQ056901 | 1 | 640 | 91.4 | 24 | 850 | chr1 | 39841502 | 39842302 | 17 | 16 | 1 | 0 | 0 | scaffold_37598 |
|  |  | 2 | 574 | 93 | 24 | 713 | chr1 | 158592639 | 158593460 | 10 | 9 | 1 | 0 | 0 | scaffold_33696 |
|  |  | 3 | 584 | 94.3 | 24 | 693 | chr1 | 51086415 | 51087073 | 8 | 6 | 2 | 0 | 0 | scaffold_37644 |
|  |  | 4 | 615 | 92.9 | 24 | 750 | chr11 | 17214117 | 17214830 | 13 | 13 | 0 | 0 | 0 | scaffold_37629 |
|  |  | 5 | 688 | 95.3 | 24 | 787 | chr12 | 117095831 | 117096578 | 16 | 16 | 0 | 0 | 0 | scaffold_37378 |
|  |  | 6 | 625 | 93.1 | 24 | 777 | chr14 | 103274160 | 103274908 | 14 | 13 | 1 | 0 | 0 | scaffold_37324 |
|  |  | 7 | 671 | 97.6 | 31 | 750 | chr16 | 1952503 | 1954630 | 15 | 15 | 0 | 0 | 0 | scaffold_34712 |
|  |  | 8 | 729 | 94.3 | 25 | 885 | chr17 | 19511375 | 19512204 | 24 | 22 | 2 | 0 | 0 | scaffold_37151 |
|  |  | 9 | 613 | 92.4 | 24 | 759 | chr17 | 52309158 | 52309888 | 15 | 15 | 0 | 0 | 0 | scaffold_37659 |
|  |  | 10 | 510 | 91.7 | 24 | 653 | chr18 | 66246425 | 66247061 | 9 | 9 | 0 | 0 | 0 | scaffold_37478 |
|  |  | 11 | 650 | 93.7 | 24 | 777 | chr2 | 85117439 | 85118176 | 16 | 14 | 2 | 0 | 0 | scaffold_36190 |
|  |  | 12 | 662 | 93.6 | 24 | 787 | chr20 | 44954702 | 44955449 | 16 | 16 | 0 | 0 | 0 | scaffold_37443 |
|  |  | 13 | 592 | 94.6 | 24 | 712 | chr4 | 144848265 | 144848908 | 9 | 4 | 1 | 4 | 0 | scaffold_36997 |
|  |  | 14 | 530 | 93 | 24 | 661 | chr4 | 43327858 | 43328507 | 6 | 6 | 0 | 0 | 0 | scaffold_37596 |
|  |  | 15 | 650 | 92.3 | 24 | 885 | chr6 | 43553477 | 43554306 | 16 | 16 | 0 | 0 | 0 | scaffold_37583 |
|  |  | 16 | 491 | 91.4 | 24 | 653 | chr7 | 23272291 | 23272985 | 11 | 11 | 0 | 0 | 0 | scaffold_34502 |
|  |  | 17 | 459 | 91.8 | 24 | 587 | chr7 | 123675070 | 123675627 | 7 | 7 | 0 | 0 | 0 | scaffold_37671 |
|  |  | 18 | 534 | 93.5 | 24 | 643 | chr9 | 98259146 | 98259782 | 5 | 5 | 0 | 0 | 0 | scaffold_37692 |
|  |  | 19 | 665 | 94.2 | 24 | 787 | chrX | 39825318 | 39826066 | 17 | 2 | 1 | 14 | 0 | scaffold_37496 |
| IMAGE:5823554 | BQ001115 | 1 | 479 | 100 | 12 | 490 | chr12 | 2782891 | 2783369 | 1 | 1 | 0 | 0 | 0 | scaffold_30586 |
|  |  | 2 | 410 | 92.1 | 1 | 490 | chr4 | 119572688 | 119573173 | 6 | 6 | 0 | 0 | 0 | scaffold_28535 |
| IMAGE:5825774 | BM994966 | 1 | 627 | 99.4 | 16 | 650 | chr5 | 135475089 | 135475723 | 1 | 0 | 1 | 0 | 0 | scaffold_37656 |
| IMAGE:5827347 | BQ019651 | 1 | 673 | 100 | 18 | 691 | chr22 | 19677206 | 19677880 | 2 | 1 | 1 | 0 | 0 | scaffold_37083 |
| IMAGE:5846417 | BQ006986 | 1 | 636 | 99.7 | 13 | 652 | chr6 | 158274215 | 158274854 | 1 | 1 | 0 | 0 | 0 | scaffold_33331 |
| IMAGE:5851036 | BQ183583 | 1 | 560 | 99.8 | 18 | 582 | chr19 | 54160844 | 54161948 | 3 | 2 | 1 | 0 | 0 | scaffold_37466 |
|  |  | 2 | 549 | 97.2 | 1 | 582 | chr20 | 3999671 | 4000252 | 1 | 1 | 0 | 0 | 0 | scaffold_37176 |
|  |  | 3 | 499 | 93.8 | 12 | 582 | chrX | 30009948 | 30010518 | 3 | 2 | 1 | 0 | 0 | scaffold_32663 |
| IMAGE:5921279 | BQ061784 | 1 | 874 | 99 | 1 | 901 | chr1 | 6415398 | 6471414 | 10 | 9 | 1 | 0 | 0 | scaffold_31944 scaffold_30472 |
| IMAGE:5930660 | BQ066045 | 1 | 745 | 98.3 | 1 | 831 | chr9_random | 1823614 | 1827186 | 16 | 13 | 0 | 3 | 0 | scaffold_34748 |
| IMAGE:594535 | AA169811 | 1 | 398 | 98 | 1 | 414 | chr10 | 89709297 | 89709708 | 3 | 2 | 1 | 0 | 0 | scaffold_37705 |
| IMAGE:594535 | AA171649 | 1 | 243 | 98.4 | 138 | 391 | chr10 | 89997288 | 90006044 | 4 | 4 | 0 | 0 | 0 | scaffold_37705 |
| IMAGE:6028109 | BQ548626 | 1 | 333 | 100 | 1 | 334 | chr11 | 2145352 | 2146472 | 2 | 1 | 1 | 0 | 0 | scaffold_34651 |
| IMAGE:6028109 | BQ548920 | 1 | 329 | 100 | 19 | 348 | chr11 | 2145352 | 2146468 | 2 | 1 | 1 | 0 | 0 | scaffold_34651 |
| IMAGE:6036618 | BU070994 | 1 | 631 | 99.7 | 1 | 635 | chr17 | 28023524 | 28024158 | 1 | 0 | 1 | 0 | 0 | scaffold_28792 |
| IMAGE:6036618 | BU073007 | 1 | 541 | 100 | 20 | 560 | chr17 | 28023940 | 28024480 | 1 | 1 | 0 | 0 | 0 | scaffold_28792 |
| IMAGE:6069988 | BQ223525 | 1 | 280 | 96.3 | 1 | 312 | chr12 | 55353056 | 55368298 | 3 | 3 | 0 | 0 | 0 | scaffold_36205 |
| IMAGE:6106631 | BU189043 | 1 | 425 | 95.2 | 357 | 821 | chr1 | 225784378 | 225784837 | 6 | 6 | 0 | 0 | 0 | scaffold_11898 |
|  |  | 2 | 768 | 98.7 | 32 | 833 | chr11 | 61507498 | 61510439 | 13 | 13 | 0 | 0 | 0 | scaffold_36755 |
|  |  | 3 | 705 | 93.6 | 32 | 833 | chr11 | 77171768 | 77172561 | 10 | 10 | 0 | 0 | 0 | scaffold_37428 |
|  |  | 4 | 728 | 95.5 | 32 | 833 | chr13 | 21068101 | 21068898 | 11 | 9 | 2 | 0 | 0 | scaffold_32325 |
|  |  | 5 | 670 | 94.9 | 93 | 833 | chr2 | 181940076 | 181940804 | 10 | 9 | 1 | 0 | 0 | scaffold_37634 |
|  |  | 6 | 674 | 95.1 | 32 | 803 | chr2 | 27590151 | 27590954 | 9 | 7 | 2 | 0 | 0 | scaffold_37688 |
|  |  | 7 | 700 | 94 | 32 | 833 | chr3 | 72898922 | 72899710 | 14 | 14 | 0 | 0 | 0 | scaffold_37430 |
|  |  | 8 | 675 | 92.8 | 32 | 833 | chr3 | 129804157 | 129804945 | 9 | 8 | 1 | 0 | 0 | scaffold_37698 |
|  |  | 9 | 716 | 94.6 | 32 | 833 | chr5 | 17406659 | 17407455 | 12 | 12 | 0 | 0 | 0 | scaffold_33904 |
|  |  | 10 | 679 | 92.3 | 32 | 833 | chr6 | 50927200 | 50927987 | 13 | 11 | 2 | 0 | 0 | scaffold_37490 |
|  |  | 11 | 690 | 94.4 | 32 | 828 | chr8 | 82483820 | 82484600 | 13 | 13 | 0 | 0 | 0 | scaffold_37639 |
|  |  | 12 | 702 | 93.8 | 32 | 833 | chr9 | 15517070 | 15517860 | 14 | 13 | 1 | 0 | 0 | scaffold_37419 |
|  |  | 13 | 719 | 95.2 | 34 | 833 | chrX | 145801245 | 145802030 | 10 | 6 | 1 | 3 | 0 | scaffold_37343 |
| IMAGE:6127384 | BU948557 | 1 | 578 | 99.7 | 1 | 582 | chr9 | 126259017 | 126259598 | 1 | 1 | 0 | 0 | 0 | scaffold_32871 |
| IMAGE:6127384 | BU948802 | 1 | 553 | 99.8 | 18 | 575 | chr9 | 126259508 | 126262174 | 4 | 3 | 1 | 0 | 0 | scaffold_32871 |
| IMAGE:6135750 | BQ100857 | 1 | 575 | 100 | 5 | 579 | chr12 | 50188668 | 50189242 | 1 | 1 | 0 | 0 | 0 | scaffold_37077 |
| IMAGE:6135750 | BQ101159 | 1 | 501 | 99.8 | 1 | 503 | chr12 | 50188428 | 50188930 | 1 | 0 | 1 | 0 | 0 | scaffold_37077 |
| IMAGE:6144678 | BU154255 | 1 | 752 | 99.5 | 1 | 779 | chr12 | 6897380 | 6901825 | 8 | 6 | 2 | 0 | 0 | scaffold_37590 |
| IMAGE:6151944 | BQ423891 | 1 | 843 | 99.1 | 1 | 865 | chr1 | 151396774 | 151409824 | 14 | 14 | 0 | 0 | 0 | scaffold_36950 |
| IMAGE:6154547 | BQ425762 | 1 | 778 | 98.6 | 10 | 837 | chr1 | 9377159 | 9382657 | 16 | 16 | 0 | 0 | 0 | scaffold_37442 |
| IMAGE:6190617 | BQ716150 | 1 | 737 | 98.8 | 1 | 761 | chr1 | 143187405 | 143189055 | 7 | 6 | 1 | 0 | 0 | scaffold_32121 |
| IMAGE:6206716 | BQ690149 | 1 | 841 | 98.4 | 1 | 914 | chr6 | 32208079 | 32209783 | 13 | 3 | 1 | 9 | 0 | scaffold_37619 |
| IMAGE:6248869 | BQ689517 | 1 | 824 | 98.9 | 1 | 865 | chr19 | 44663330 | 44670697 | 16 | 15 | 1 | 0 | 0 | scaffold_37165 |
| IMAGE:6262938 | BQ684162 | 1 | 773 | 99.2 | 12 | 819 | chr16 | 58476461 | 58485650 | 10 | 10 | 0 | 0 | 0 | scaffold_37666 |
| IMAGE:628623 | AA192191 | 1 | 437 | 99.8 | 1 | 443 | chr1 | 148359073 | 148360554 | 4 | 4 | 0 | 0 | 0 | scaffold_36950 |
| IMAGE:628623 | AA194560 | 1 | 315 | 97.3 | 1 | 350 | chr1 | 148363448 | 148365008 | 8 | 4 | 0 | 4 | 0 | scaffold_36950 |
| IMAGE:6294443 | BQ677162 | 1 | 525 | 92.9 | 1 | 604 | chr11 | 89704647 | 89705237 | 7 | 7 | 0 | 0 | 0 | scaffold_37405 |
|  |  | 2 | 547 | 94.5 | 1 | 603 | chr12 | 47952599 | 47953195 | 7 | 7 | 0 | 0 | 0 | scaffold_37077 |
|  |  | 3 | 588 | 95.5 | 1 | 633 | chr12 | 47808070 | 47808692 | 10 | 9 | 1 | 0 | 0 | scaffold_1310 |
|  |  | 4 | 516 | 96 | 29 | 580 | chr12 | 47865170 | 47865716 | 7 | 7 | 0 | 0 | 0 | scaffold_37077 |
| IMAGE:6301617 | BQ710896 | 1 | 743 | 97 | 1 | 784 | chr17 | 529520 | 530297 | 6 | 6 | 0 | 0 | 0 | scaffold_37555 |
| IMAGE:6379190 | BU501962 | 1 | 864 | 97.1 | 1 | 925 | chr7 | 73230732 | 73231639 | 10 | 9 | 1 | 0 | 0 | scaffold_34601 |
| IMAGE:6537798 | BU527920 | 1 | 800 | 99.4 | 36 | 854 | chr17 | 7326685 | 7328769 | 13 | 12 | 1 | 0 | 0 | scaffold_37549 |
| IMAGE:6547599 | CA866149 | 1 | 585 | 99.2 | 1 | 596 | chr6 | 53009399 | 53010006 | 2 | 1 | 1 | 0 | 0 | scaffold_37490 |
| IMAGE:6547599 | CA941121 | 1 | 592 | 99.8 | 1 | 601 | chr6 | 52990442 | 53005154 | 7 | 7 | 0 | 0 | 0 | scaffold_37490 |
| IMAGE:6648216 | BU858257 | 1 | 721 | 94.5 | 1 | 801 | chr1 | 154259793 | 154260588 | 4 | 3 | 1 | 0 | 0 | scaffold_36950 |
|  |  | 2 | 609 | 90.2 | 113 | 858 | chr1 | 43982936 | 43983671 | 8 | 8 | 0 | 0 | 0 | scaffold_37599 |
|  |  | 3 | 656 | 91.6 | 4 | 801 | chr10 | 8560356 | 8561145 | 8 | 8 | 0 | 0 | 0 | scaffold_37507 |
|  |  | 4 | 667 | 91.2 | 4 | 847 | chr11 | 9080281 | 9081132 | 13 | 13 | 0 | 0 | 0 | scaffold_37629 |
|  |  | 5 | 727 | 92.5 | 4 | 858 | chr11 | 119011866 | 119012713 | 7 | 7 | 0 | 0 | 0 | scaffold_37627 |
|  |  | 6 | 675 | 91.9 | 11 | 847 | chr11 | 4708930 | 4709755 | 12 | 12 | 0 | 0 | 0 | scaffold_37629 |
|  |  | 7 | 661 | 92.3 | 4 | 793 | chr12 | 60559320 | 60560113 | 7 | 7 | 0 | 0 | 0 | scaffold_36205 |
|  |  | 8 | 634 | 90.3 | 4 | 793 | chr12 | 67385636 | 67386424 | 4 | 4 | 0 | 0 | 0 | scaffold_37580 |
|  |  | 9 | 836 | 98.9 | 1 | 858 | chr12 | 51580003 | 51585080 | 11 | 10 | 0 | 1 | 0 | scaffold_37077 |
|  |  | 10 | 636 | 90.1 | 4 | 801 | chr14 | 37186238 | 37187030 | 7 | 4 | 3 | 0 | 0 | scaffold_32092 |
|  |  | 11 | 672 | 92.9 | 4 | 801 | chr15 | 68866825 | 68867620 | 6 | 3 | 3 | 0 | 0 | scaffold_37693 |
|  |  | 12 | 615 | 91.4 | 4 | 830 | chr15 | 46980009 | 46980927 | 10 | 10 | 0 | 0 | 0 | scaffold_37412 |
|  |  | 13 | 619 | 90.8 | 11 | 765 | chr15 | 74987896 | 74988649 | 2 | 0 | 1 | 1 | 0 | scaffold_37637 |
|  |  | 14 | 670 | 91 | 24 | 841 | chr16 | 77862534 | 77863345 | 9 | 9 | 0 | 0 | 0 | scaffold_37614 |
|  |  | 15 | 691 | 91.5 | 6 | 841 | chr2 | 184138970 | 184139800 | 6 | 5 | 1 | 0 | 0 | scaffold_37634 |
|  |  | 16 | 683 | 91.1 | 4 | 851 | chr3 | 171534098 | 171534931 | 7 | 5 | 2 | 0 | 0 | scaffold_37927 |
|  |  | 17 | 590 | 90.1 | 25 | 754 | chr3 | 87293295 | 87294023 | 2 | 1 | 1 | 0 | 0 | scaffold_32945 |
|  |  | 18 | 711 | 91.9 | 4 | 851 | chr3 | 139141870 | 139142713 | 9 | 8 | 1 | 0 | 0 | scaffold_36172 |
|  |  | 19 | 706 | 91 | 2 | 858 | chr4 | 104108941 | 104109790 | 11 | 11 | 0 | 0 | 0 | scaffold_20635 |
|  |  | 20 | 733 | 94.5 | 1 | 816 | chr5 | 122813488 | 122814300 | 6 | 4 | 2 | 0 | 0 | scaffold_37656 |
|  |  | 21 | 708 | 93.5 | 2 | 847 | chr5 | 97804655 | 97805501 | 9 | 9 | 0 | 0 | 0 | scaffold_37626 |
|  |  | 22 | 703 | 91.8 | 4 | 858 | chr5 | 146114886 | 146115716 | 10 | 9 | 1 | 0 | 0 | scaffold_37144 |
|  |  | 23 | 627 | 91.1 | 4 | 816 | chr5 | 60055950 | 60056725 | 11 | 9 | 2 | 0 | 0 | scaffold_37612 |
|  |  | 24 | 697 | 91.1 | 4 | 858 | chr6 | 134599628 | 134600449 | 9 | 9 | 0 | 0 | 0 | scaffold_34646 |
|  |  | 25 | 658 | 90.9 | 30 | 847 | chr7 | 38435539 | 38436367 | 9 | 9 | 0 | 0 | 0 | scaffold_33340 |
|  |  | 26 | 388 | 95.1 | 71 | 501 | chr7 | 136039066 | 136039500 | 2 | 2 | 0 | 0 | 0 | scaffold_37657 |
|  |  | 27 | 792 | 95.4 | 1 | 858 | chr8 | 62540692 | 62541543 | 7 | 7 | 0 | 0 | 0 | scaffold_27056 |
|  |  | 28 | 660 | 90.8 | 24 | 846 | chr9 | 97447266 | 97448072 | 8 | 8 | 0 | 0 | 0 | scaffold_37692 |
|  |  | 29 | 678 | 92.4 | 4 | 801 | chrX | 150265617 | 150266409 | 4 | 3 | 1 | 0 | 0 | scaffold_37326 |
|  |  | 30 | 658 | 90.7 | 4 | 801 | chrX | 56977866 | 56978658 | 4 | 4 | 0 | 0 | 0 | scaffold_37519 |
|  |  | 31 | 689 | 91.8 | 4 | 829 | chrX | 44538181 | 44539005 | 7 | 7 | 0 | 0 | 0 | scaffold_37620 |
| IMAGE:6648254 | BU858290 | 1 | 717 | 98.4 | 1 | 786 | chr15 | 85849940 | 85851683 | 15 | 14 | 1 | 0 | 0 | scaffold_31000 |
| IMAGE:665231 | AA195662 | 1 | 381 | 99.2 | 1 | 384 | chr15 | 29040286 | 29040668 | 2 | 1 | 1 | 0 | 0 | scaffold_37002 |
| IMAGE:665231 | AA195775 | 1 | 432 | 96.2 | 1 | 469 | chr15 | 29041053 | 29041511 | 9 | 9 | 0 | 0 | 0 | scaffold_37002 |
| IMAGE:67076 | T70347 | 1 | 276 | 99.6 | 1 | 287 | chr4 | 100442716 | 100443002 | 1 | 1 | 0 | 0 | 0 | scaffold_36289 |
| IMAGE:67076 | T70432 | 1 | 397 | 96.6 | 1 | 422 | chr4 | 100442333 | 100442745 | 8 | 7 | 1 | 0 | 0 | scaffold_36289 |
| IMAGE:6971771 | CD559533 | 1 | 452 | 100 | 33 | 488 | chr5 | 131953690 | 131955409 | 5 | 5 | 0 | 0 | 0 | scaffold_37656 |
| IMAGE:6971771 | CD559687 | 1 | 445 | 99.8 | 15 | 469 | chr5 | 131953690 | 131955409 | 5 | 5 | 0 | 0 | 0 | scaffold_37656 |
| IMAGE:724703 | AA291791 | 1 | 365 | 99.5 | 13 | 381 | chr11 | 47557470 | 47557838 | 1 | 1 | 0 | 0 | 0 | scaffold_37695 |
| IMAGE:726657 | AA398244 | 1 | 332 | 99.1 | 1 | 334 | chr16 | 27046601 | 27046933 | 2 | 2 | 0 | 0 | 0 | scaffold_17005 |
| IMAGE:726657 | AA399310 | 1 | 430 | 100 | 1 | 430 | chr16 | 27046608 | 27047037 | 1 | 1 | 0 | 0 | 0 | scaffold_17005 |
| IMAGE:726657 | BX091593 | 1 | 449 | 100 | 8 | 456 | chr16 | 27046589 | 27047037 | 1 | 0 | 1 | 0 | 0 | scaffold_17005 |
| IMAGE:730015 | AA416859 | 1 | 387 | 100 | 3 | 389 | chr9 | 125361104 | 125361490 | 1 | 1 | 0 | 0 | 0 | scaffold_32871 |
| IMAGE:730015 | AA469916 | 1 | 403 | 100 | 1 | 403 | chr9 | 125360604 | 125361006 | 1 | 0 | 1 | 0 | 0 | scaffold_32871 |
| IMAGE:74849 | T47148 | 1 | 357 | 100 | 1 | 359 | chr14 | 57243734 | 57244178 | 3 | 3 | 0 | 0 | 0 | scaffold_32092 |
| IMAGE:74849 | T47149 | 1 | 379 | 95.9 | 1 | 403 | chr14 | 57245586 | 57245979 | 8 | 8 | 0 | 0 | 0 | scaffold_32092 |
| IMAGE:755736 | AA496445 | 1 | 380 | 91 | 1 | 465 | chr13 | 28668656 | 28669121 | 2 | 1 | 1 | 0 | 0 | scaffold_37433 |
|  |  | 2 | 451 | 98.7 | 1 | 465 | chr19 | 12768646 | 12771803 | 3 | 2 | 1 | 0 | 0 | scaffold_37371 |
|  |  | 3 | 368 | 92.2 | 1 | 447 | chr6 | 100629940 | 100630389 | 4 | 1 | 1 | 2 | 0 | scaffold_37679 |
| IMAGE:755736 | AA496505 | 1 | 391 | 90.8 | 11 | 497 | chr13 | 28668263 | 28668744 | 4 | 4 | 0 | 0 | 0 | scaffold_37433 |
|  |  | 2 | 469 | 99 | 18 | 500 | chr19 | 12771712 | 12773087 | 5 | 4 | 1 | 0 | 0 | scaffold_37371 |
|  |  | 3 | 389 | 91.5 | 11 | 500 | chr6 | 100629519 | 100630012 | 4 | 4 | 0 | 0 | 0 | scaffold_37679 |
| IMAGE:756783 | AA425329 | 1 | 349 | 91.1 | 1 | 445 | chr15 | 42830301 | 42830750 | 6 | 4 | 2 | 0 | 0 | scaffold_37412 |
|  |  | 2 | 334 | 90.5 | 1 | 445 | chr15 | 43086374 | 43086812 | 7 | 7 | 0 | 0 | 0 | scaffold_37412 |
|  |  | 3 | 444 | 100 | 1 | 445 | chr20 | 35906899 | 35907347 | 2 | 0 | 2 | 0 | 0 | scaffold_37418 |
| IMAGE:756783 | AA426444 | 1 | 245 | 91.6 | 115 | 419 | chr15 | 43086031 | 43086342 | 6 | 6 | 0 | 0 | 0 | scaffold_37412 |
|  |  | 2 | 242 | 92 | 115 | 419 | chr15 | 42830782 | 42831091 | 6 | 5 | 1 | 0 | 0 | scaffold_37412 |
|  |  | 3 | 403 | 100 | 17 | 419 | chr20 | 35907380 | 35907782 | 1 | 0 | 1 | 0 | 0 | scaffold_37418 |
| IMAGE:769838 | AA430440 | 1 | 236 | 99.6 | 1 | 238 | chr11 | 63519121 | 63519358 | 1 | 1 | 0 | 0 | 0 | scaffold_37276 |
| IMAGE:782944 | AA468038 | 1 | 362 | 98.9 | 9 | 378 | chr15 | 41211857 | 41212226 | 1 | 0 | 1 | 0 | 0 | scaffold_37412 |
| IMAGE:824308 | AA490827 | 1 | 397 | 100 | 10 | 407 | chr9 | 67326841 | 67327239 | 2 | 2 | 0 | 0 | 0 | scaffold_34695 |
| IMAGE:852604 | AA663100 | 1 | 317 | 99.7 | 1 | 320 | chr5 | 15729960 | 15730280 | 2 | 2 | 0 | 0 | 0 | scaffold_37681 |
| IMAGE:923948 | AA514235 | 1 | 440 | 100 | 1 | 441 | chr14 | 43575144 | 43577359 | 2 | 1 | 1 | 0 | 0 | scaffold_32092 |
| IMAGE:924830 | AA515494 | 1 | 477 | 99.6 | 1 | 483 | chr7 | 30414138 | 30415918 | 3 | 1 | 2 | 0 | 0 | scaffold_34502 |
| IMAGE:980394 | AA524812 | 1 | 539 | 100 | 1 | 545 | chr1 | 158396121 | 158399062 | 7 | 7 | 0 | 0 | 0 | scaffold_30731 |
| **Sum** | | | | | | | | | | 6183 | 5040 | 539 | 382 | 222 |  |
| **Average** | | | | | | | | | |  | 81.51% | 8.72% | 6.18% | 3.59% |  |
